# Author Correction: Ago2/CAV1 interaction potentiates metastasis via controlling Ago2 localization and miRNA action

**DOI:** 10.1038/s44319-024-00162-1

**Published:** 2024-06-05

**Authors:** Meng-Chieh Lin, Wen-Hung Kuo, Shih-Yin Chen, Jing-Ya Hsu, Li-Yu Lu, Chen-Chi Wang, Yi-Ju Chen, Jia-Shiuan Tsai, Hua-Jung Li

**Affiliations:** 1 Institute of Cellular and System Medicine, National Health Research Institutes, Miaoli, 35053 Taiwan; 2 Department of Surgery, National Taiwan University Hospital, Taipei, 100229 Taiwan; 3 Institute of Biotechnology, National Tsing Hua University, Hsinchu, 30013 Taiwan; 4 Program in Tissue Engineering and Regenerative Medicine, National Chung Hsing University, Taichung City, 402 Taiwan

## Abstract

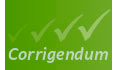

**Correction to:** *EMBO Reports* (2024) 25:2441–2478. 10.1038/s44319-024-00132-7 | Published online 22 April 2024

**Author’s name is corrected**.

The 4th Authors name is spelled incorrectly.

Jin-Ya Hsu

Is corrected to:

Jing-Ya Hsu

