# Supplementary material for: Ago2/CAV1 interaction potentiates metastasis via controlling Ago2 localization and miRNA action
Source: EMBO Rep. 2024 Apr 22;25(5):20. doi: 10.1038/s44319-024-00132-7 (PMC11094075; doi:10.1038/s44319-024-00132-7)
Supplement: Supplementary file 1 — Appendix [file 44319_2024_132_MOESM1_ESM.pdf]

APPENDIX for

Ago2/CAV1 interaction potentiates metastasis via controlling Ago2 localization and miRNA action

Table of Contents

Appendix Figure S1 Blockage of Ago2/CAV1 interaction interferes with the expression of miRNAs and mRNAs in cancer cells., related to Fig. 3 2

Appendix Figure S2. Ago2/CAV1 interaction in tumor progression, related to Fig. 5 4

Appendix Figure S3. Quantitation of Western blots in Figure 6., related to Fig. 6 5

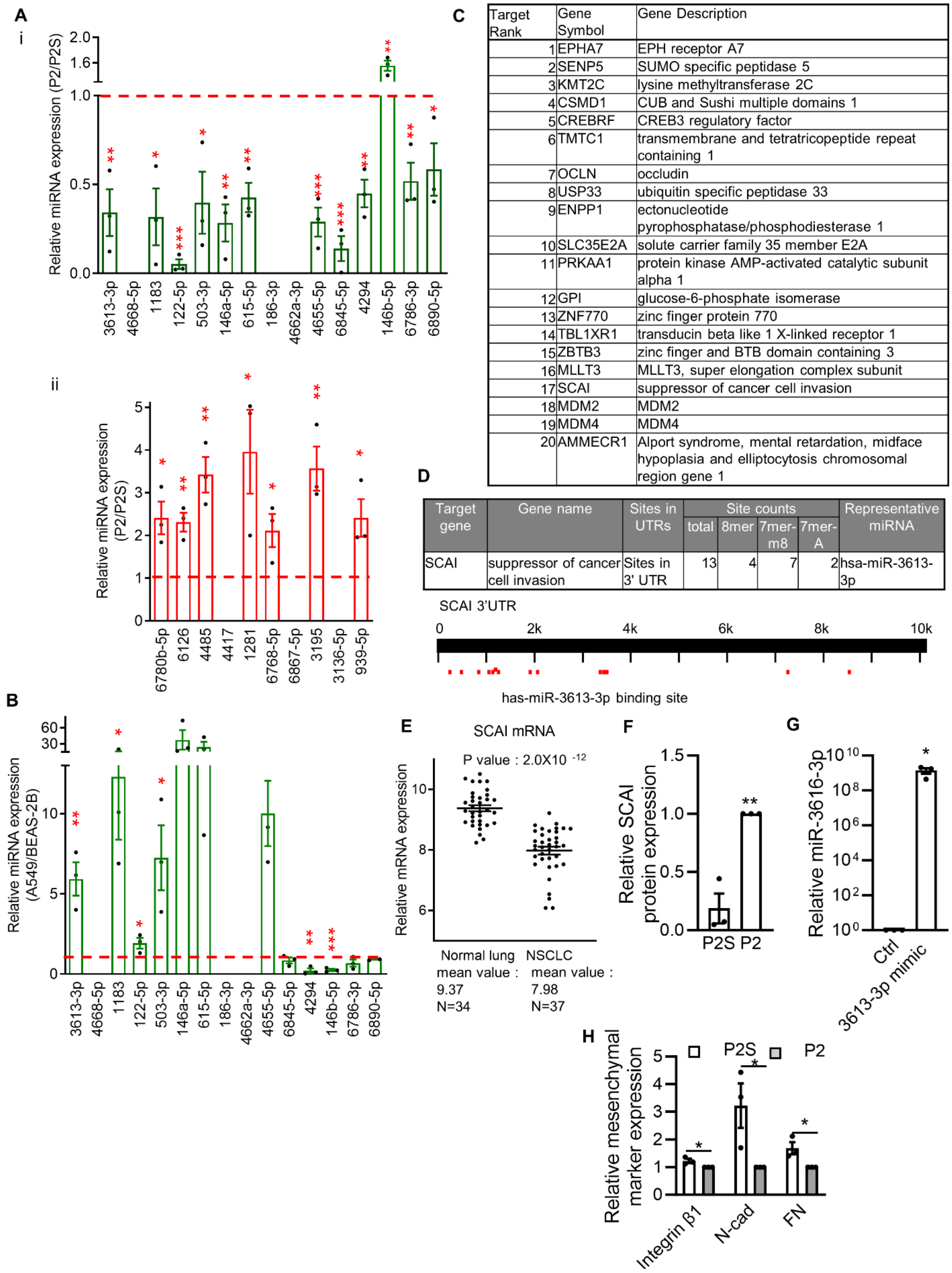

Target Rank	Gene Symbol	Gene Description
1	EPHA7	EPH receptor A7
2	SENP5	SUMO specific peptidase 5
3	KMT2C	lysine methyltransferase 2C
4	CSMD1	CUB and Sushi multiple domains 1
5	CREBRF	CREB3 regulatory factor
6	TMTC1	transmembrane and tetratricopeptide repeat containing 1
7	OCLN	occludin
8	USP33	ubiquitin specific peptidase 33
9	ENPP1	ectonucleotide pyrophosphatase/phosphodiesterase 1
10	SLC35E2A	solute carrier family 35 member E2A
11	PRKAA1	protein kinase AMP-activated catalytic subunit alpha 1
12	GPI	glucose-6-phosphate isomerase
13	ZNF770	zinc finger protein 770
14	TBL1XR1	transducin beta like 1 X-linked receptor 1
15	ZBTB3	zinc finger and BTB domain containing 3
16	MLLT3	MLLT3, super elongation complex subunit
17	SCAI	suppressor of cancer cell invasion
18	MDM2	MDM2
19	MDM4	MDM4
20	AMMECR1	Alport syndrome, mental retardation, midface hypoplasia and elliptocytosis chromosomal region gene 1

Target gene	Gene name	Sites in UTRs	Site counts				Representative miRNA
			total	8mer	7mer-m8	7mer-A	
SCAI	suppressor of cancer cell invasion	Sites in 3' UTR	13	4	7	2	hsa-miR-3613-3p

Appendix Figure S1. Blockage of Ago2/CAV1 interaction interferes with the expression of miRNAs

and mRNAs in cancer cells. (A) Top 15 downregulated miRNAs (panel i) and top 10 upregulated miRNAs (panel ii) with P2 peptides in the miRNA array assays in Fig. 3A, verified using qPCR. Each spot describes the relative miRNA expression level in P2-treated A549 cancer cells to that in P2S-treated cells. Bars are means ± SEM (n = 3, biological replicates). Student's t test, $^{*}P \leq 0.05$; $^{**}P \leq 0.01$; $^{***}P \leq 0.001$. (B) Differentially expressed miRNAs in BEAS-2B normal epithelial cells and A549 cancer cells. Each spot describes the relative miRNA expression level in A549 cells to that in BEAS-2B cells. Bars are means ± SEM (n = 3, biological replicates). Student's t test, $^{*}P \leq 0.05$; $^{**}P \leq 0.01$; $^{***}P \leq 0.001$. (C) Top 20 gene targets of miR-3613-3p indicated by miRDB. (D) Putative miR-3613-3p binding sites on SCAI mRNAs. (E) Differentially expressed SCAI miRNAs in normal lung tissues and non–small cell lung cancer tissues of patients. Each spot describes the mRNA level of a sample from the GEO data set GSE102287. Bars are means ± SEM (n = 34 and 37, patient number). Student's t test, $P = 2 \times 10^{-12}$. (F) Expression of SCAI proteins in P2- and P2S-treated A549 cancer cells. The quantitation of Western blots of experiments in figure 3F. Each spot describes the level of SCAI proteins in nuclear fraction of the sample normalized by HDAC1, relative to that of P2-treated sample in each replicated experiment. Bars are means ± SEM (n = 3, biological replicates). Student's t test, $^{**}P \leq 0.01$. (G) Levels of miR-3613-3p in A549 and miR-3613-3p mimic overexpressed A549 cancer cells. The bar chart describes the relative expression level of miR-3613-3p in a sample to that of A549 cancer cells. Bars are means ± SEM (n = 3, biological replicates). Student's t test, $^{*}P \leq 0.05$. (H) Expression of mesenchymal markers integrin β1, E-cadherin, and fibronectin in P2- and P2S-treated A549 cancer cells. The quantitation of Western blots of experiments in figure 3F. Each spot describes the level of the mesenchymal marker in membrane fraction of the sample normalized by CAV1, relative to that of P2-treated sample in each replicated experiment. Bars are means ± SEM (n = 3, biological replicates). Student's t test, $^{*}P \leq 0.05$.

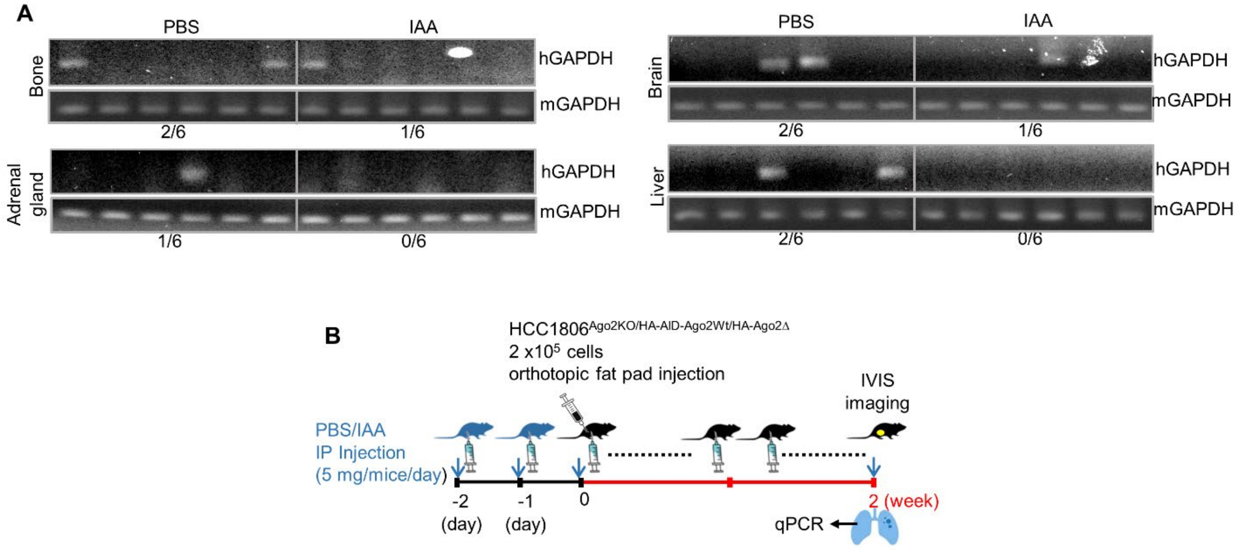

Appendix Figure S2. Ago2/CAV1 interaction in tumor progression. (A) PCR detection of A549$^{Ago2KO/HA\text{-}AID\text{-}Ago2Wt/HA\text{-}Ago2\Delta}$ cell genomic DNA (gDNA) in the bones, adrenal glands, brains, and livers of mice in Fig. 5B. (B) Scheme of animal experiments in Fig. 5F, indicating the time points of intraperitoneal injection of PBS/IAA, IV injection of HCC1806$^{Ago2KO/HA\text{-}AID\text{-}Ago2Wt/HA\text{-}Ago2\Delta}$, and IVIS imaging and tissue collection.

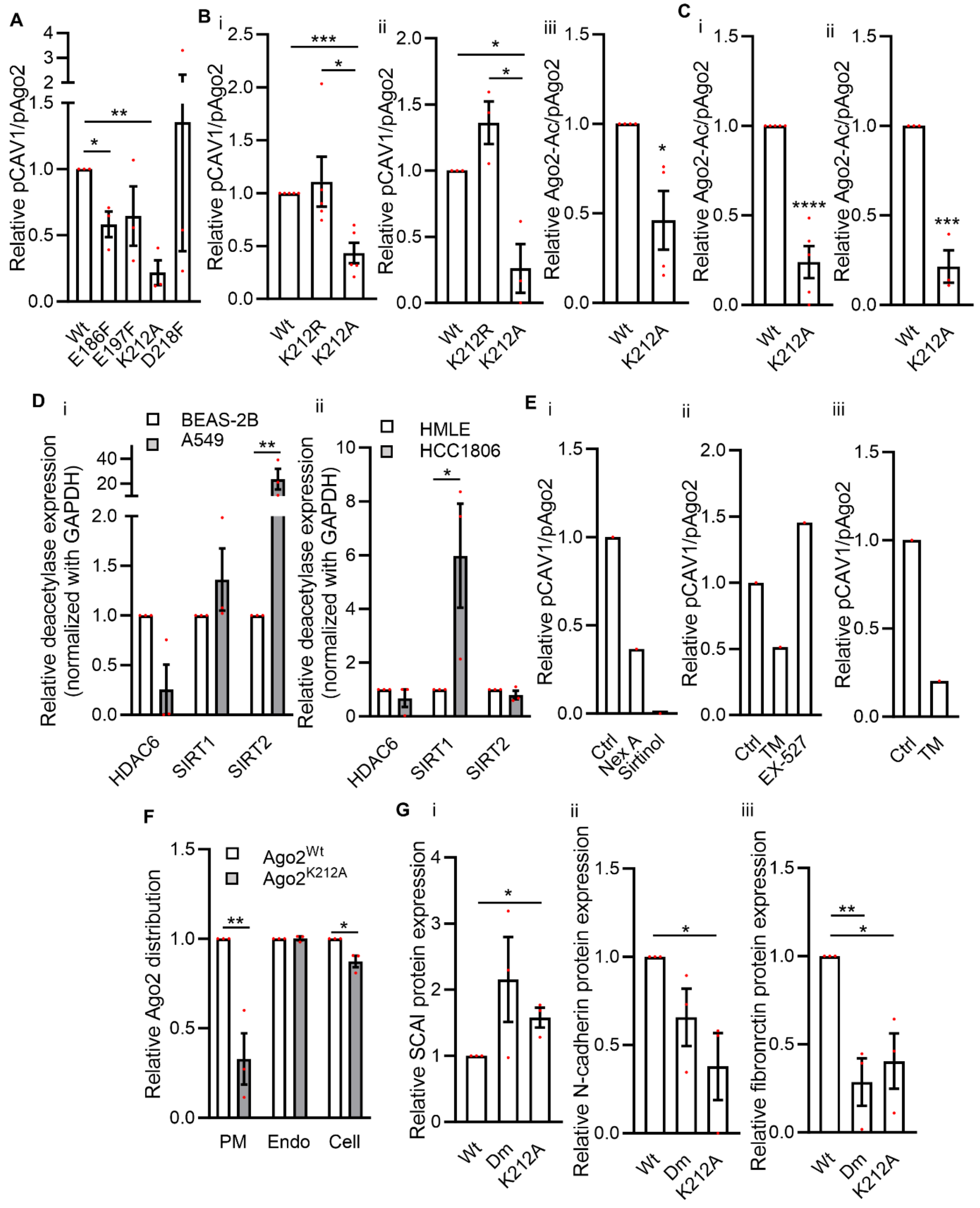

Appendix Figure S3. Quantitation of Western blots in Figure 6. (A) The quantitation of Western blots of experiments in figure 6A. Each spot describes the ratio of coprecipitated CAV1 (pCAV1) to precipitated Ago2 (pAgo2), relative to that of the mixture with wild-type HA-Ago2 (Wt) in each replicated experiment. Bars are means ± SEM (*n* = 3, biological replicates). Student's t test, $^{*}P \leq 0.05$; $^{**}P \leq 0.01$. (B) The quantitation of Western blots of experiments in figure 6Bi (panel i), 6Bii (panel ii), and 6Biii (panel iii).

Each spot describes the ratio of coprecipitated CAV1 (pCAV1) to precipitated Ago2 (pAgo2), relative to that of the mixture with wild-type HA-Ago2 (Wt) in each replicated experiment. Bars are means ± SEM (*n* = 3-5, biological replicates). Student's t test, $^{*}P \leq 0.05$; $^{***}P \leq 0.001$. (C) The quantitation of Western blots of experiments in figure 6Ci (panel i) and 6Cii (panel ii). Each spot describes the level of acetylated lysine (Ago2-Ac) of the precipitated Ago2 (pAgo2), relative to that of the wild-type HA-Ago2 (Wt) in each replicated experiment. Bars are means ± SEM (*n* = 5 and 3, biological replicates). Student's t test, $^{***}P \leq 0.001$; $^{****}P \leq 0.0001$. (D) The quantitation of Western blots of experiments in figure 6D, panel i for lung cells and panel ii for breast cells. Each spot describes the level of the deacetylase of the sample normalized by GAPDH, relative to that of normal epithelial cell sample (BEAS-2B or HMLE) in each replicated experiment. Bars are means ± SEM (*n* = 3, biological replicates). Student's t test, $^{*}P \leq 0.05$; $^{**}P \leq 0.01$. (E) The quantitation of Western blots of experiments in figure 6Ei (panel i), 6Eii (panel ii), and 6Eiii (panel iii). Each spot describes the ratio of coprecipitated CAV1 (pCAV1) to precipitated Ago2 (pAgo2), relative to that of the sample treated with vehicle (Ctrl) in each replicated experiment. *n* = 1. (F) The quantitation of Western blots of experiments in figure 6G. Each spot describes the level of Ago2 in the fraction, relative to that of $A549^{Ago2KO/HA\text{-}Ago2Wt}$ in each replicated experiment. Bars are means ± SEM (*n* = 3, biological replicates). Student's t test, $^{*}P \leq 0.05$; $^{**}P \leq 0.01$. (G) Expression of SCAI, E-cadherin, and fibronectin in $A549^{Ago2KO/HA\text{-}Ago2Wt}$ (Wt), $A549^{Ago2KO/HA\text{-}Ago2\Delta}$ (Dm), and $A549^{Ago2KO/HA\text{-}Ago2K212A}$ (K212A) cells. The quantitation of Western blots of experiments in figure 6J (panel i) and 6K (panel ii and iii). Each spot describes the protein level of the sample normalized by HDAC1 (panel i) or β-actin (panel ii and iii), relative to that of $A549^{Ago2KO/HA\text{-}Ago2Wt}$ in each replicated experiment. Bars are means ± SEM (*n* = 3, biological replicates). Student's t test, $^{*}P \leq 0.05$; $^{**}P \leq 0.01$.
