# Supplementary figures and images for: Ago2/CAV1 interaction potentiates metastasis via controlling Ago2 localization and miRNA action (part 1 of 3)
Source: EMBO Rep. 2024 Apr 22;25(5):20. doi: 10.1038/s44319-024-00132-7 (PMC11094075; doi:10.1038/s44319-024-00132-7)

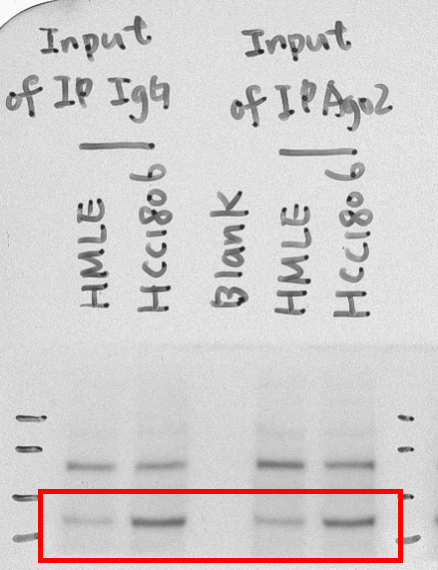

Supplement: Supplementary file 2 — Source data Fig. 1 [file 44319_2024_132_MOESM2_ESM.zip › Figure 1/1A/Breast/western Input Ago2 R.tif]

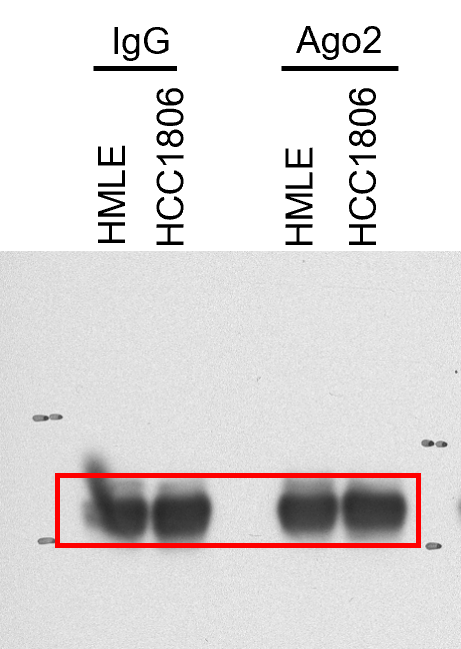

Supplement: Supplementary file 2 — Source data Fig. 1 [file 44319_2024_132_MOESM2_ESM.zip › Figure 1/1A/Breast/western Input CVA1 R.tif]

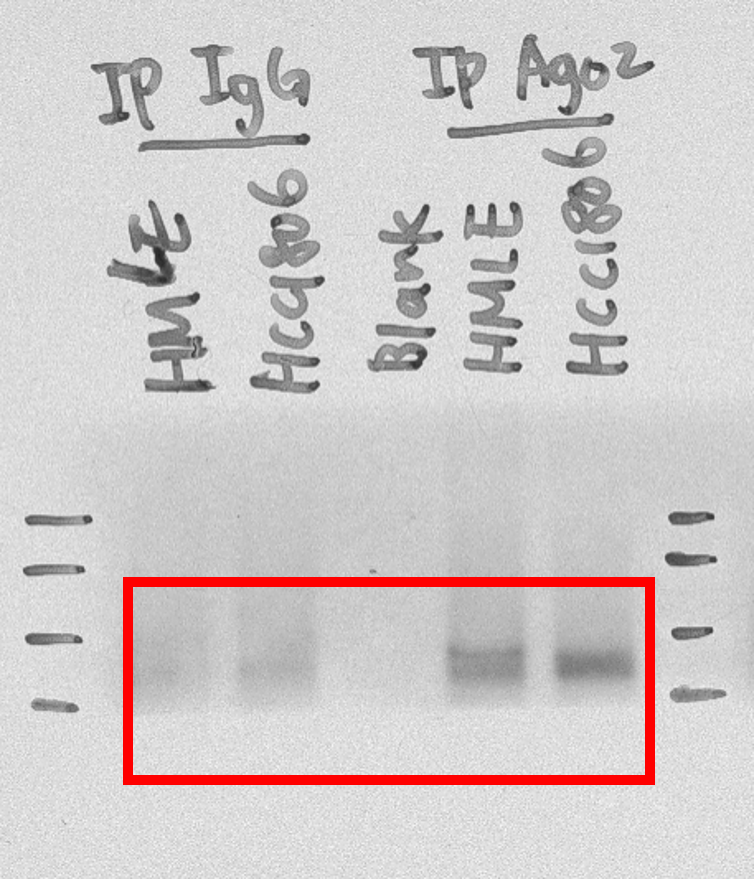

Supplement: Supplementary file 2 — Source data Fig. 1 [file 44319_2024_132_MOESM2_ESM.zip › Figure 1/1A/Breast/western IP Ago2 R.tif]

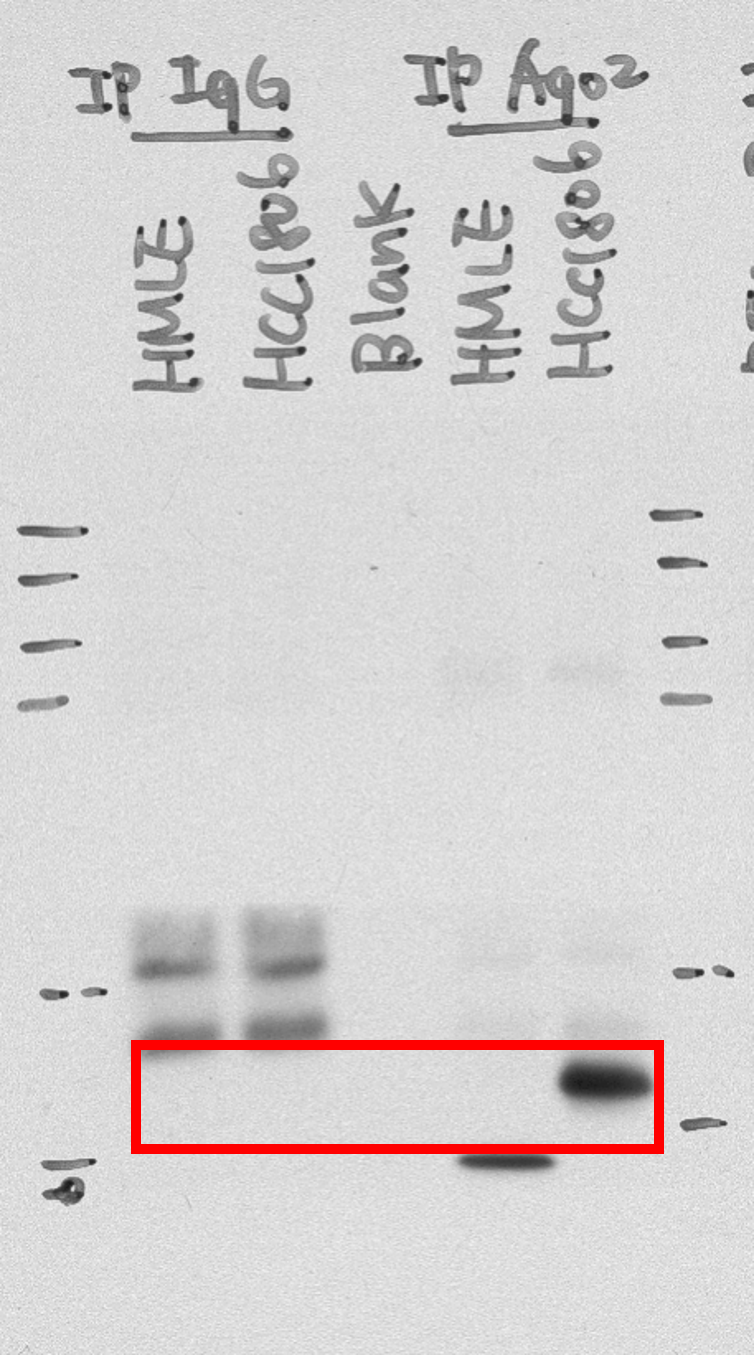

Supplement: Supplementary file 2 — Source data Fig. 1 [file 44319_2024_132_MOESM2_ESM.zip › Figure 1/1A/Breast/western IP CVA1 R.tif]

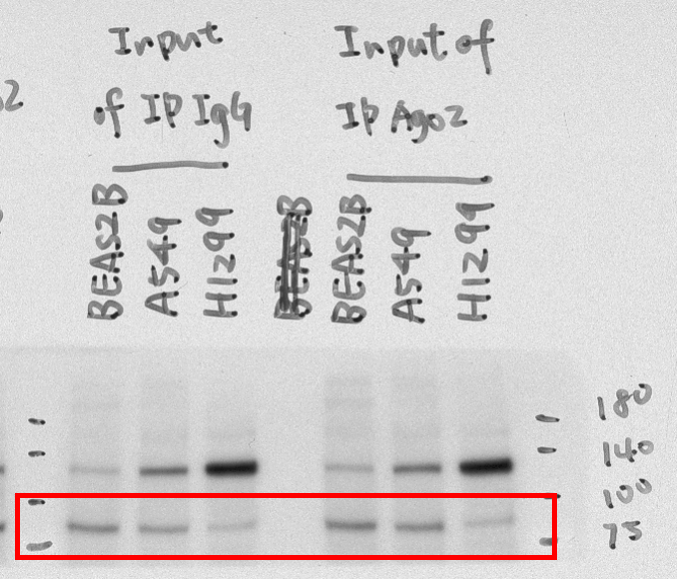

Supplement: Supplementary file 2 — Source data Fig. 1 [file 44319_2024_132_MOESM2_ESM.zip › Figure 1/1A/Lung/western Input Ago2-2.tif]

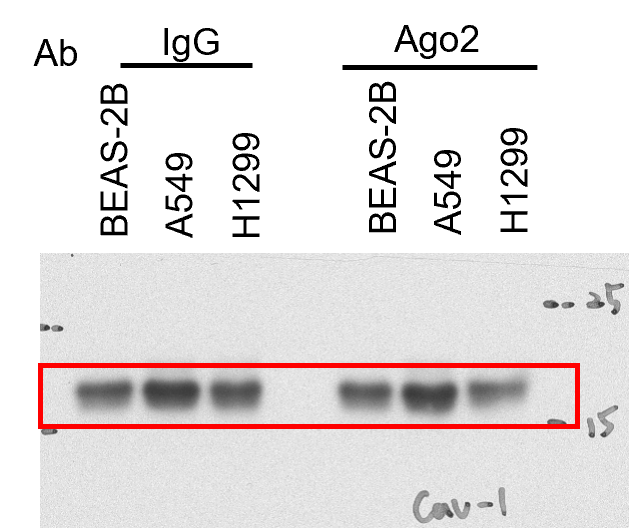

Supplement: Supplementary file 2 — Source data Fig. 1 [file 44319_2024_132_MOESM2_ESM.zip › Figure 1/1A/Lung/western Input CVA1-2.tif]

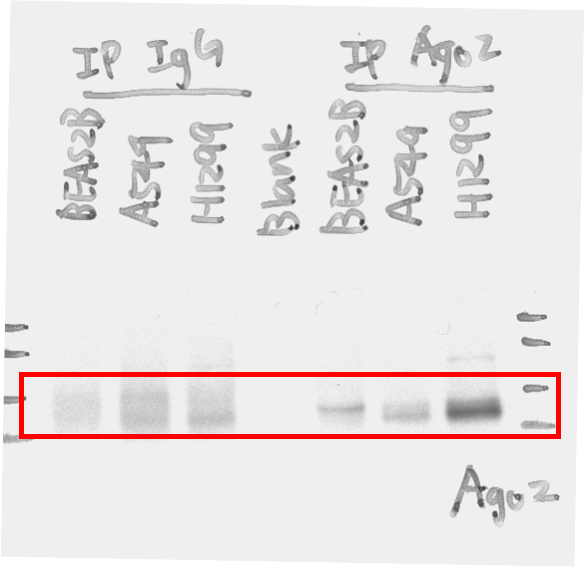

Supplement: Supplementary file 2 — Source data Fig. 1 [file 44319_2024_132_MOESM2_ESM.zip › Figure 1/1A/Lung/western IP Ago2-2.tif]

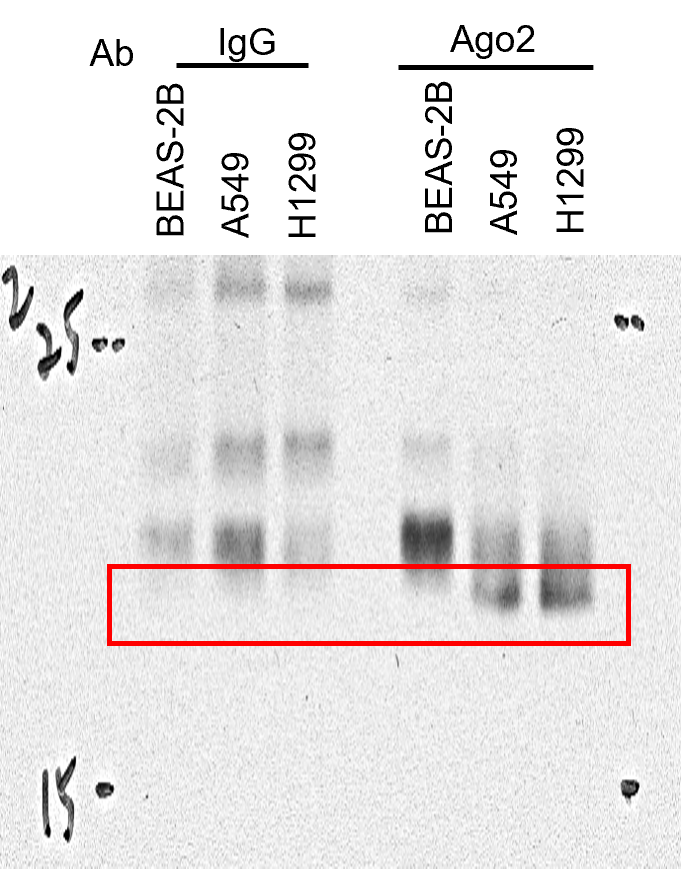

Supplement: Supplementary file 2 — Source data Fig. 1 [file 44319_2024_132_MOESM2_ESM.zip › Figure 1/1A/Lung/western IP CVA1-2.tif]

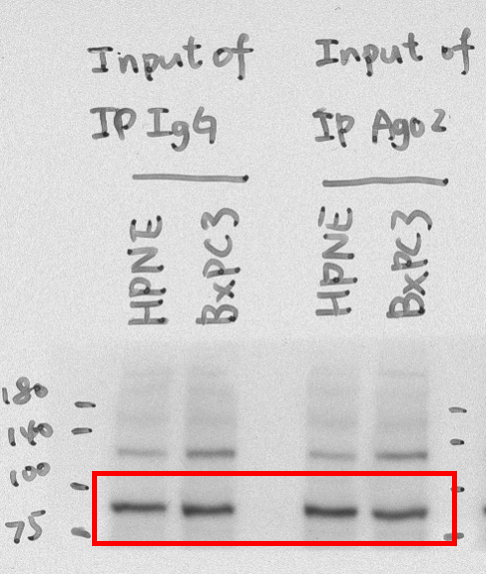

Supplement: Supplementary file 2 — Source data Fig. 1 [file 44319_2024_132_MOESM2_ESM.zip › Figure 1/1A/Pancreas/western Input Ago2-2.tif]

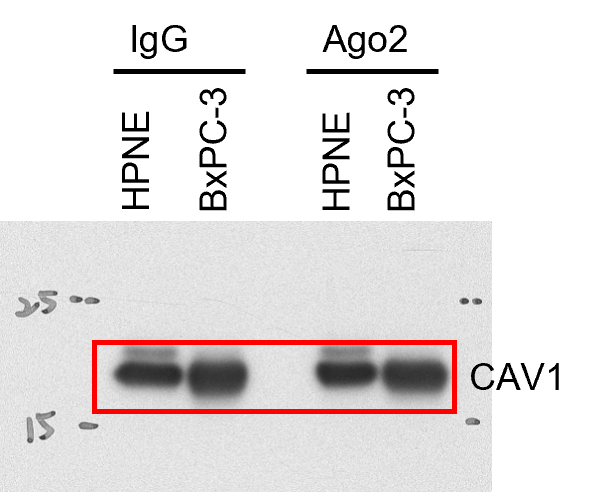

Supplement: Supplementary file 2 — Source data Fig. 1 [file 44319_2024_132_MOESM2_ESM.zip › Figure 1/1A/Pancreas/western Input CVA1-2.tif]

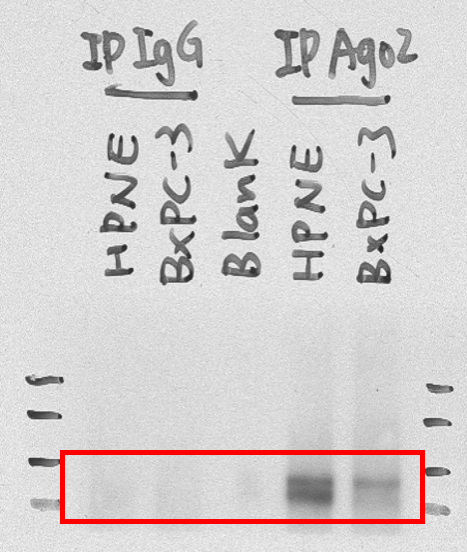

Supplement: Supplementary file 2 — Source data Fig. 1 [file 44319_2024_132_MOESM2_ESM.zip › Figure 1/1A/Pancreas/western IP Ago2-2.tif]

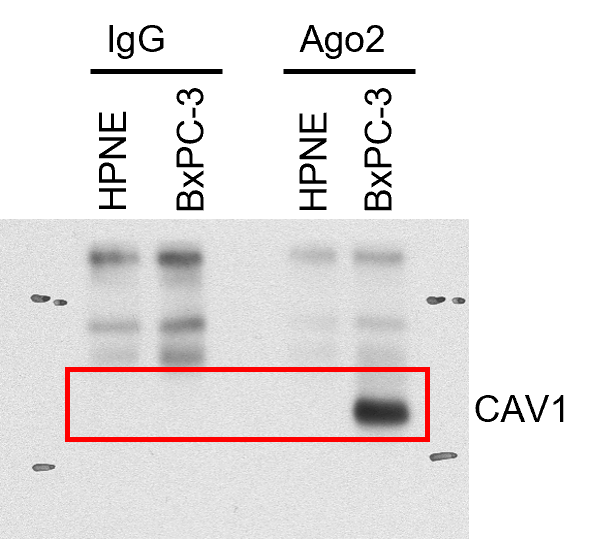

Supplement: Supplementary file 2 — Source data Fig. 1 [file 44319_2024_132_MOESM2_ESM.zip › Figure 1/1A/Pancreas/western IP CVA1-2.tif]

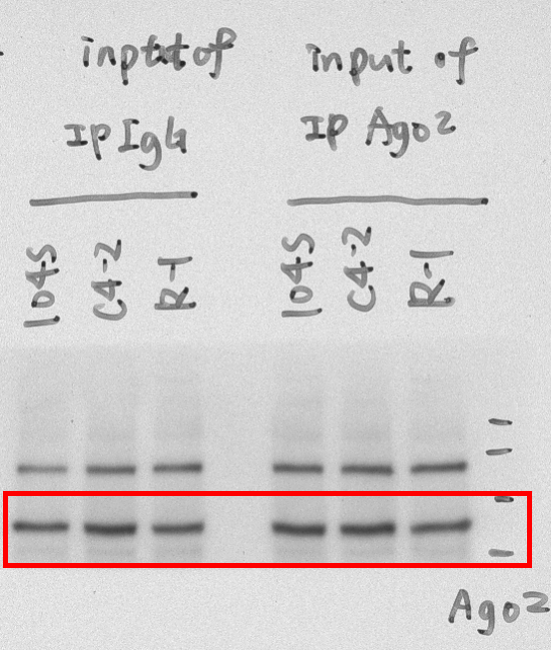

Supplement: Supplementary file 2 — Source data Fig. 1 [file 44319_2024_132_MOESM2_ESM.zip › Figure 1/1A/Prostate/western Input Ago2-2.tif]

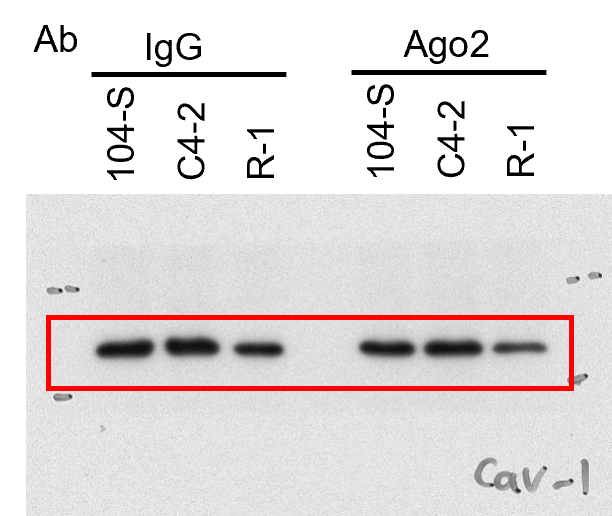

Supplement: Supplementary file 2 — Source data Fig. 1 [file 44319_2024_132_MOESM2_ESM.zip › Figure 1/1A/Prostate/western Input CVA1-2.tif]

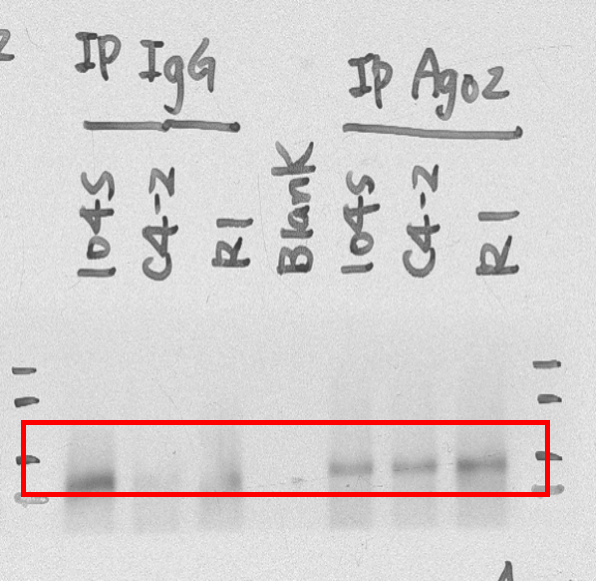

Supplement: Supplementary file 2 — Source data Fig. 1 [file 44319_2024_132_MOESM2_ESM.zip › Figure 1/1A/Prostate/western IP Ago2-2.tif]

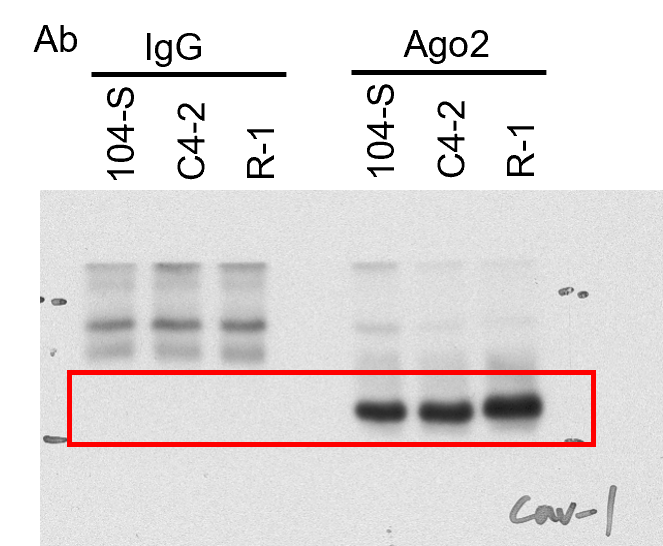

Supplement: Supplementary file 2 — Source data Fig. 1 [file 44319_2024_132_MOESM2_ESM.zip › Figure 1/1A/Prostate/western IP CVA1-2.tif]

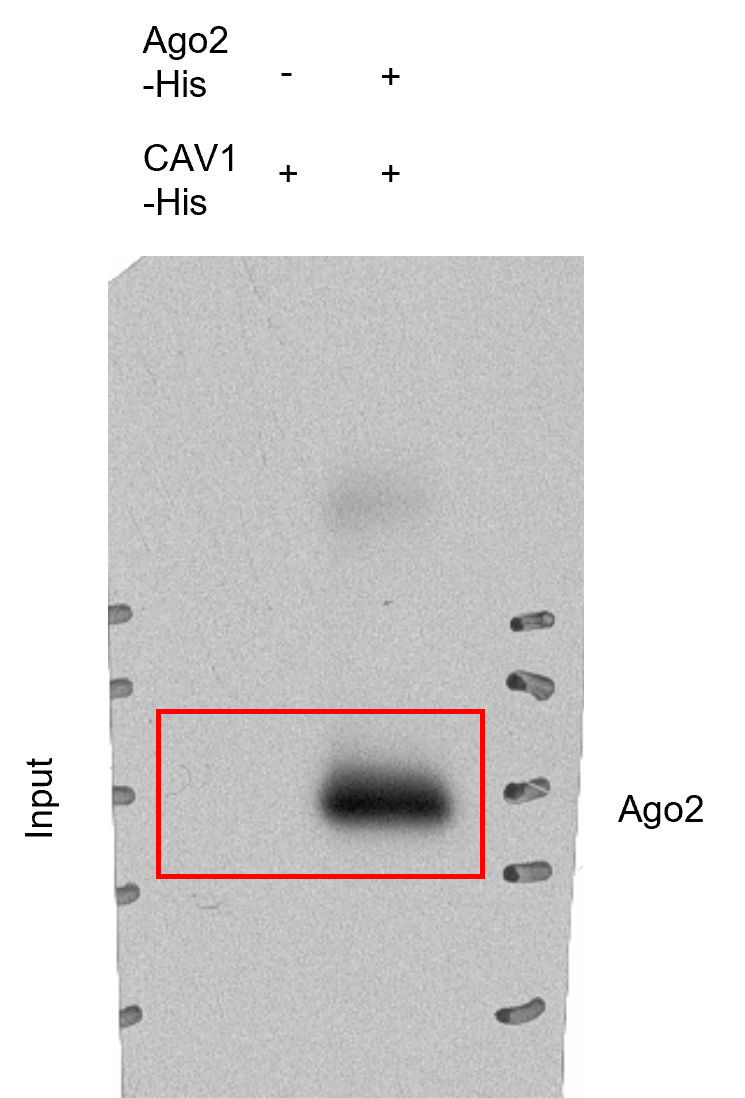

Supplement: Supplementary file 2 — Source data Fig. 1 [file 44319_2024_132_MOESM2_ESM.zip › Figure 1/1B/western Input Ago2.tif]

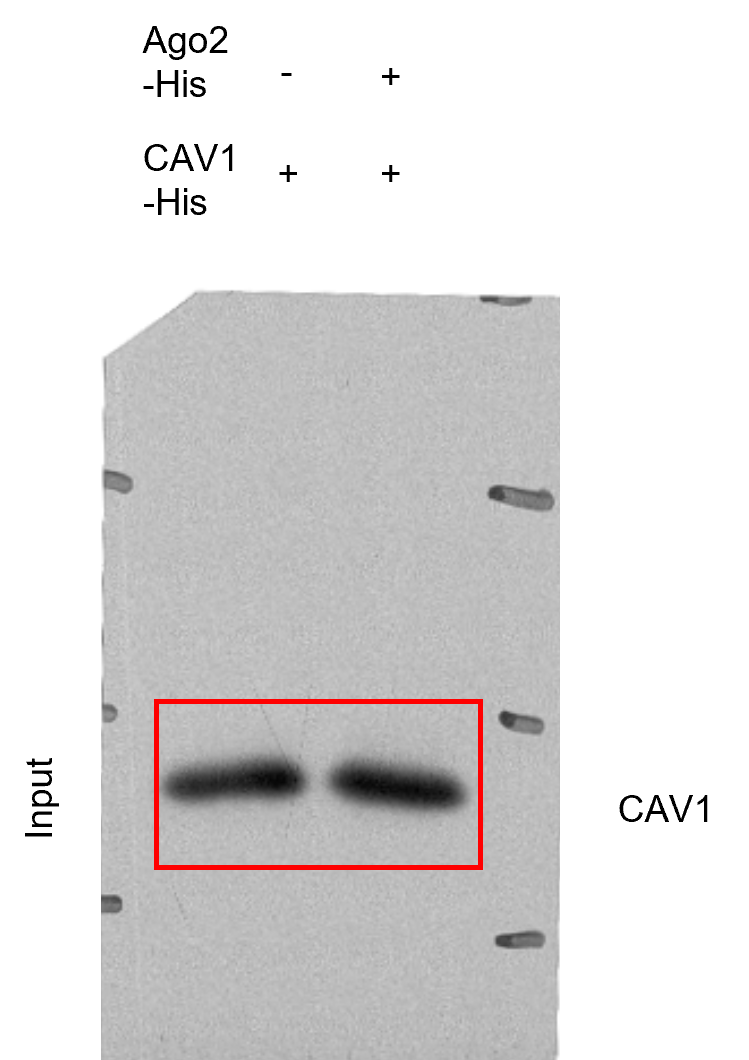

Supplement: Supplementary file 2 — Source data Fig. 1 [file 44319_2024_132_MOESM2_ESM.zip › Figure 1/1B/western Input CAV1.tif]

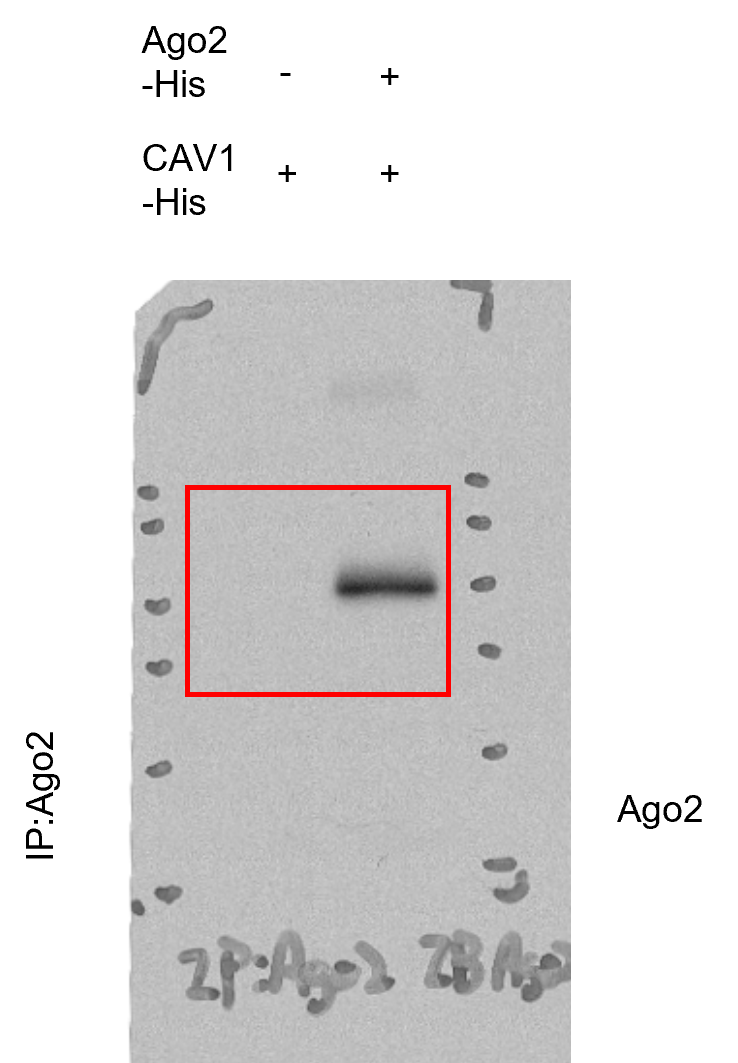

Supplement: Supplementary file 2 — Source data Fig. 1 [file 44319_2024_132_MOESM2_ESM.zip › Figure 1/1B/western IP Ago2.tif]

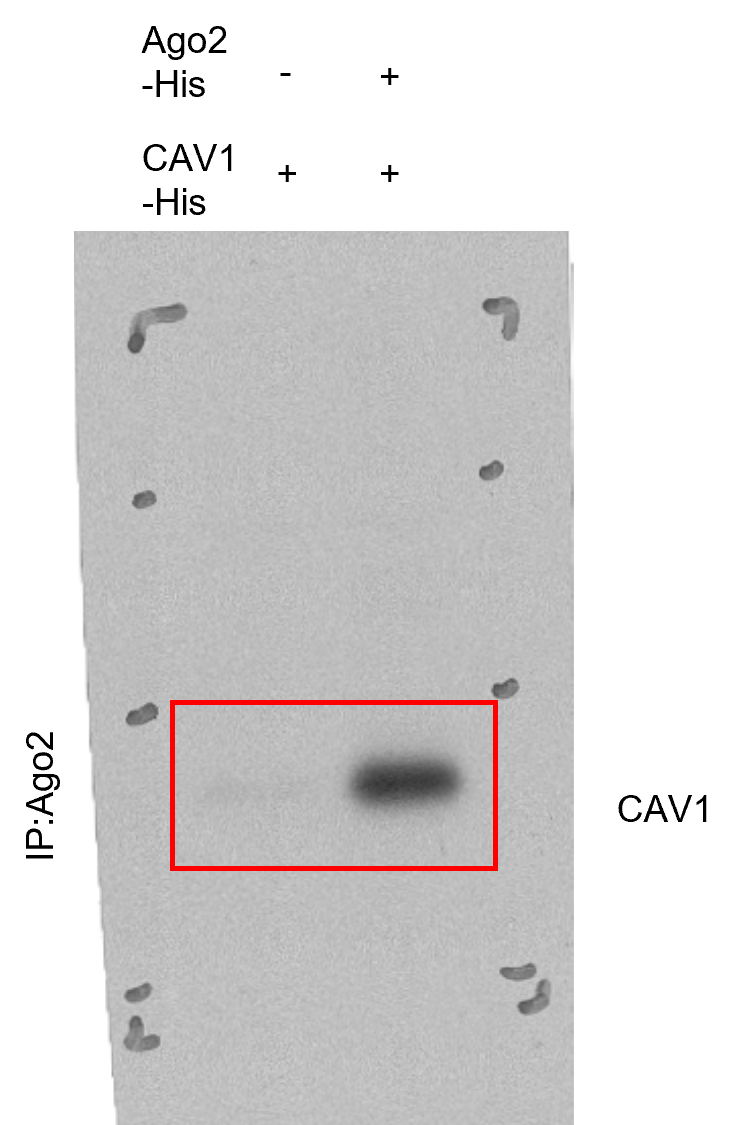

Supplement: Supplementary file 2 — Source data Fig. 1 [file 44319_2024_132_MOESM2_ESM.zip › Figure 1/1B/western IP CAV1.tif]

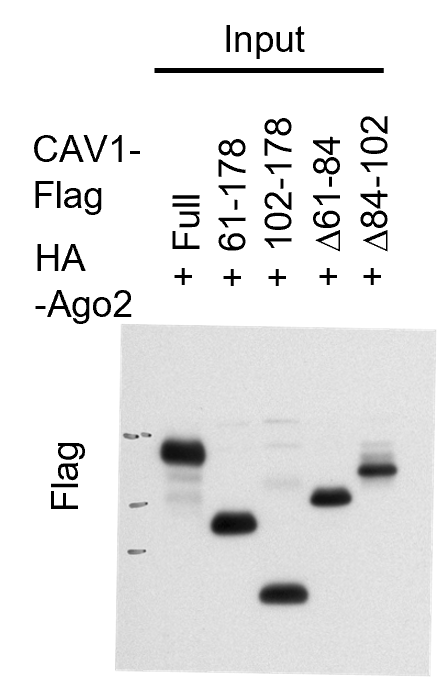

Supplement: Supplementary file 2 — Source data Fig. 1 [file 44319_2024_132_MOESM2_ESM.zip › Figure 1/1C/western Input CAV1.tif]

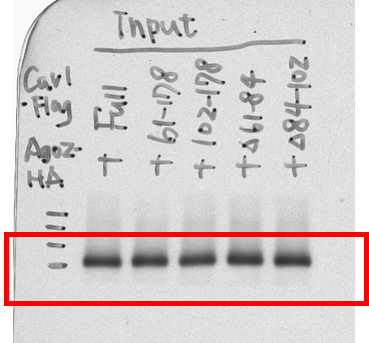

Supplement: Supplementary file 2 — Source data Fig. 1 [file 44319_2024_132_MOESM2_ESM.zip › Figure 1/1C/western Input HA.tif]

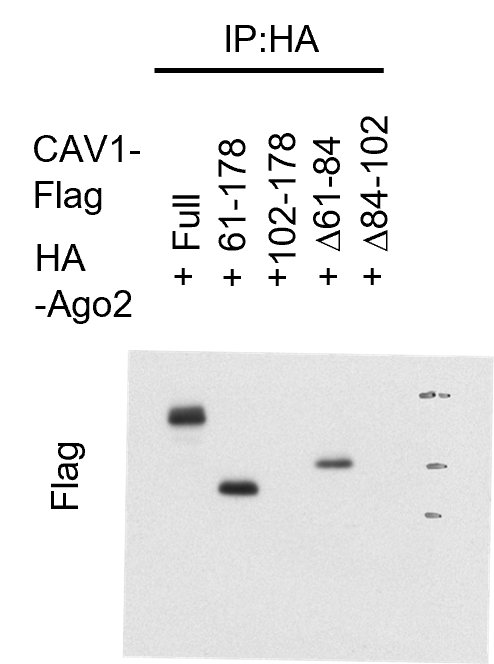

Supplement: Supplementary file 2 — Source data Fig. 1 [file 44319_2024_132_MOESM2_ESM.zip › Figure 1/1C/western IP CAV1.tif]

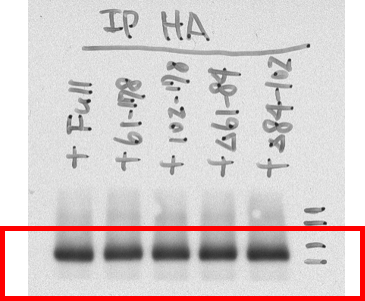

Supplement: Supplementary file 2 — Source data Fig. 1 [file 44319_2024_132_MOESM2_ESM.zip › Figure 1/1C/western IP HA.tif]

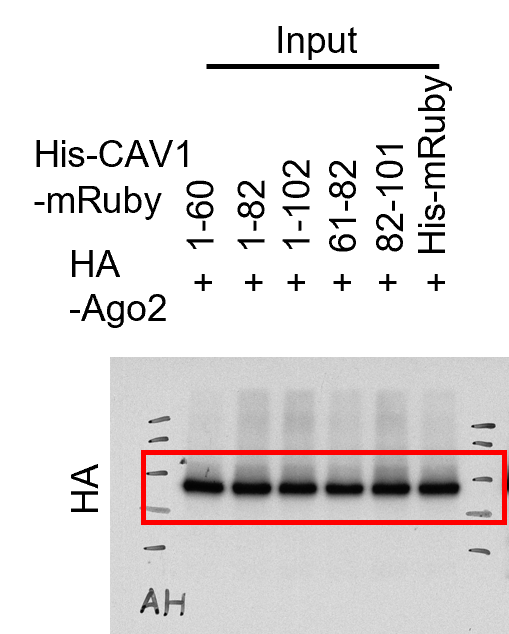

Supplement: Supplementary file 2 — Source data Fig. 1 [file 44319_2024_132_MOESM2_ESM.zip › Figure 1/1D/western Input HA.tif]

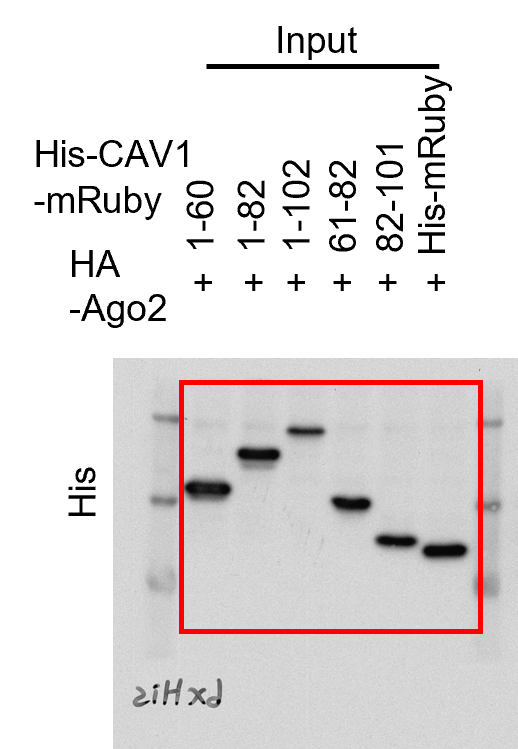

Supplement: Supplementary file 2 — Source data Fig. 1 [file 44319_2024_132_MOESM2_ESM.zip › Figure 1/1D/western Input His.tif]

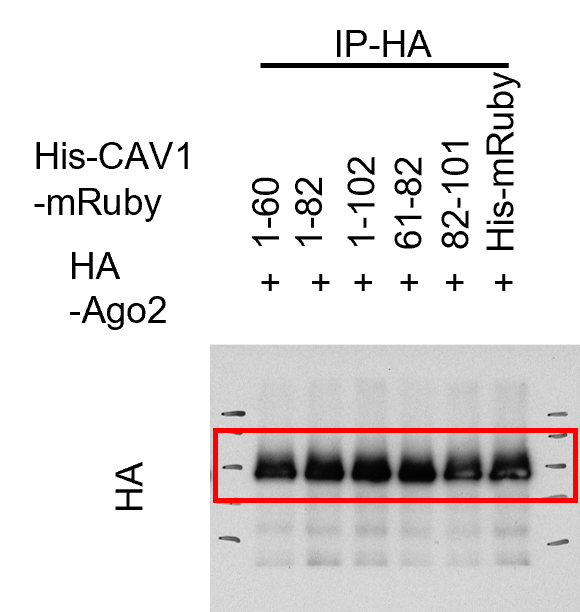

Supplement: Supplementary file 2 — Source data Fig. 1 [file 44319_2024_132_MOESM2_ESM.zip › Figure 1/1D/western IP HA.tif]

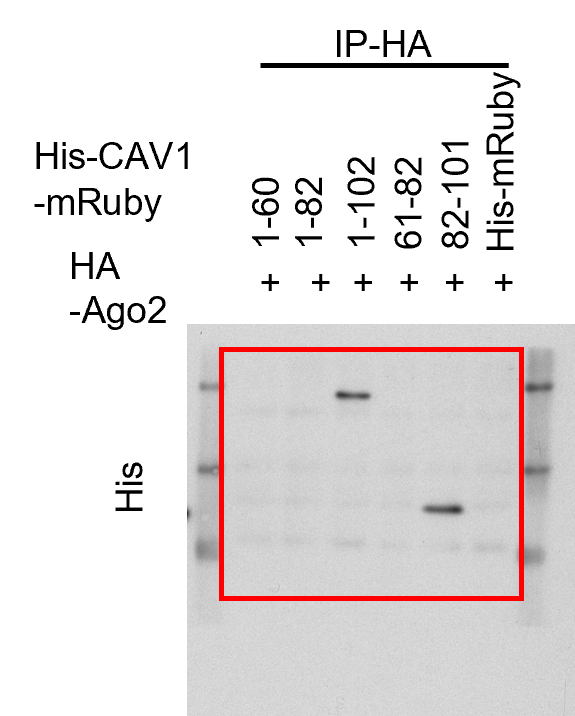

Supplement: Supplementary file 2 — Source data Fig. 1 [file 44319_2024_132_MOESM2_ESM.zip › Figure 1/1D/western IP His.tif]

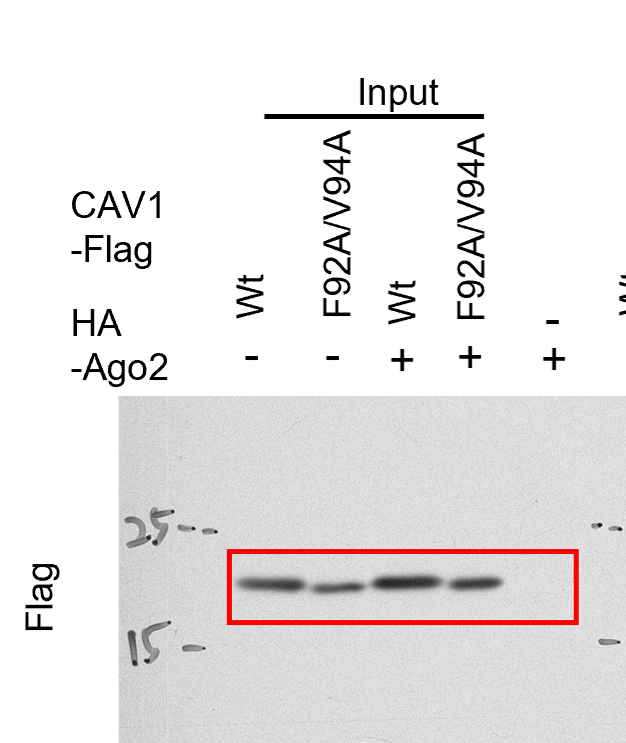

Supplement: Supplementary file 2 — Source data Fig. 1 [file 44319_2024_132_MOESM2_ESM.zip › Figure 1/1E/western Input Flag.tif]

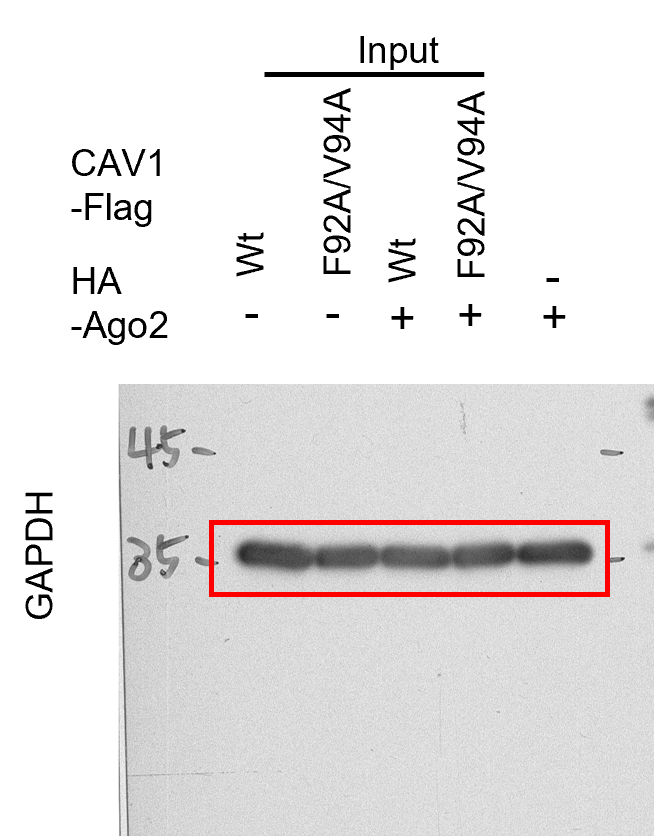

Supplement: Supplementary file 2 — Source data Fig. 1 [file 44319_2024_132_MOESM2_ESM.zip › Figure 1/1E/western Input GAPDH.tif]

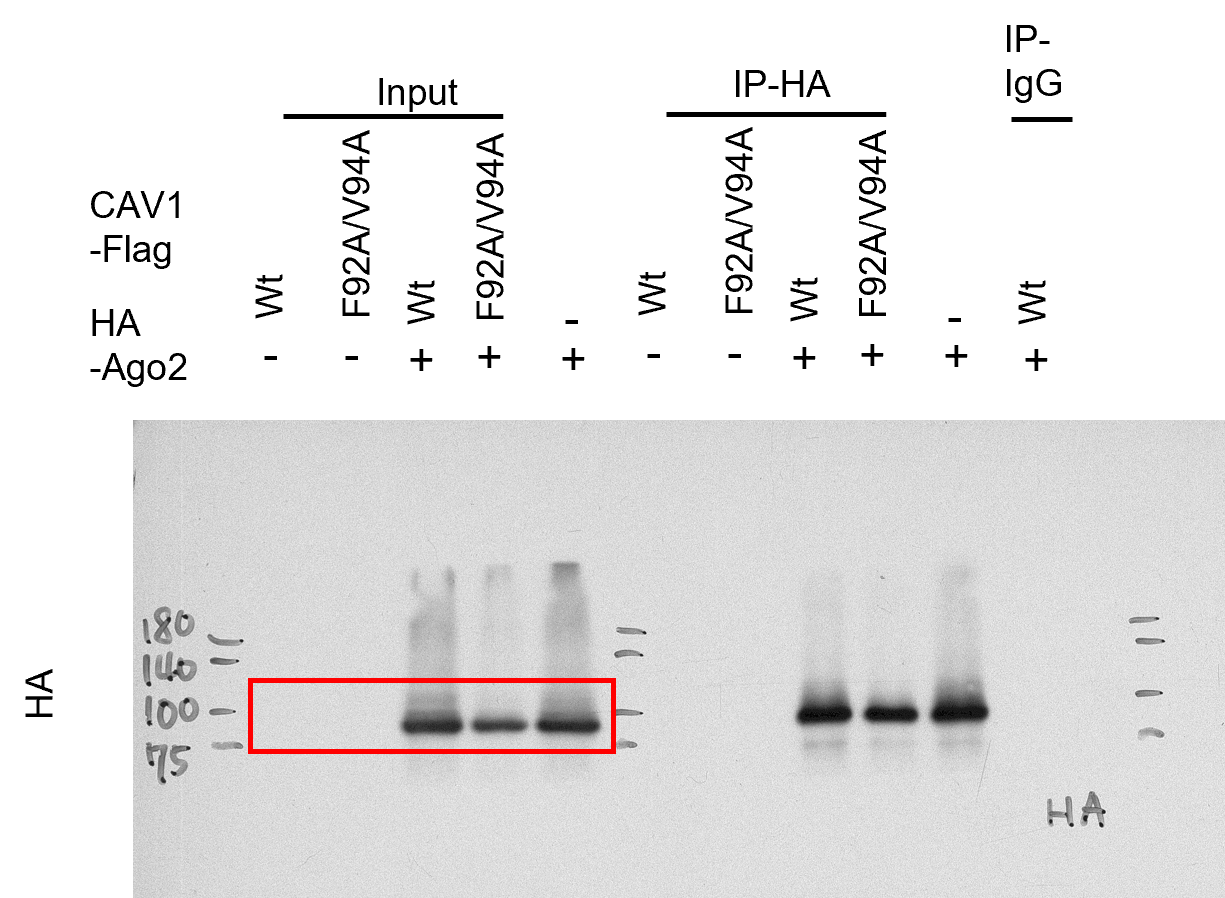

Supplement: Supplementary file 2 — Source data Fig. 1 [file 44319_2024_132_MOESM2_ESM.zip › Figure 1/1E/western Input HA.tif]

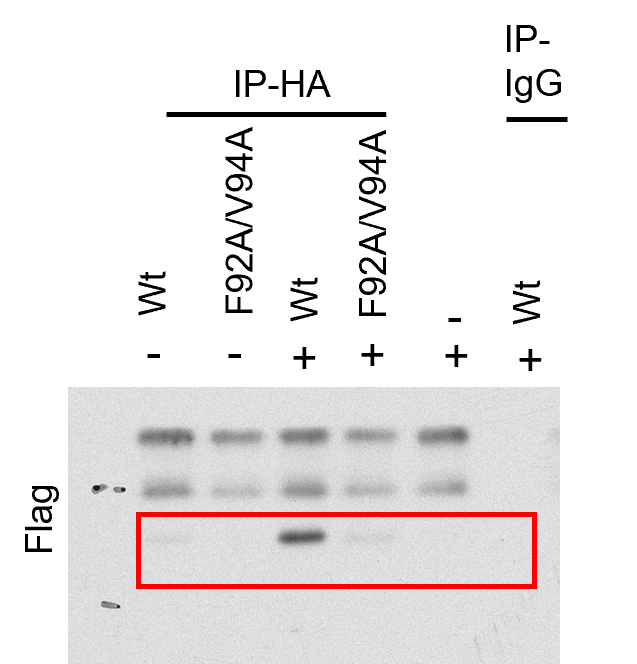

Supplement: Supplementary file 2 — Source data Fig. 1 [file 44319_2024_132_MOESM2_ESM.zip › Figure 1/1E/western IP Flag.tif]

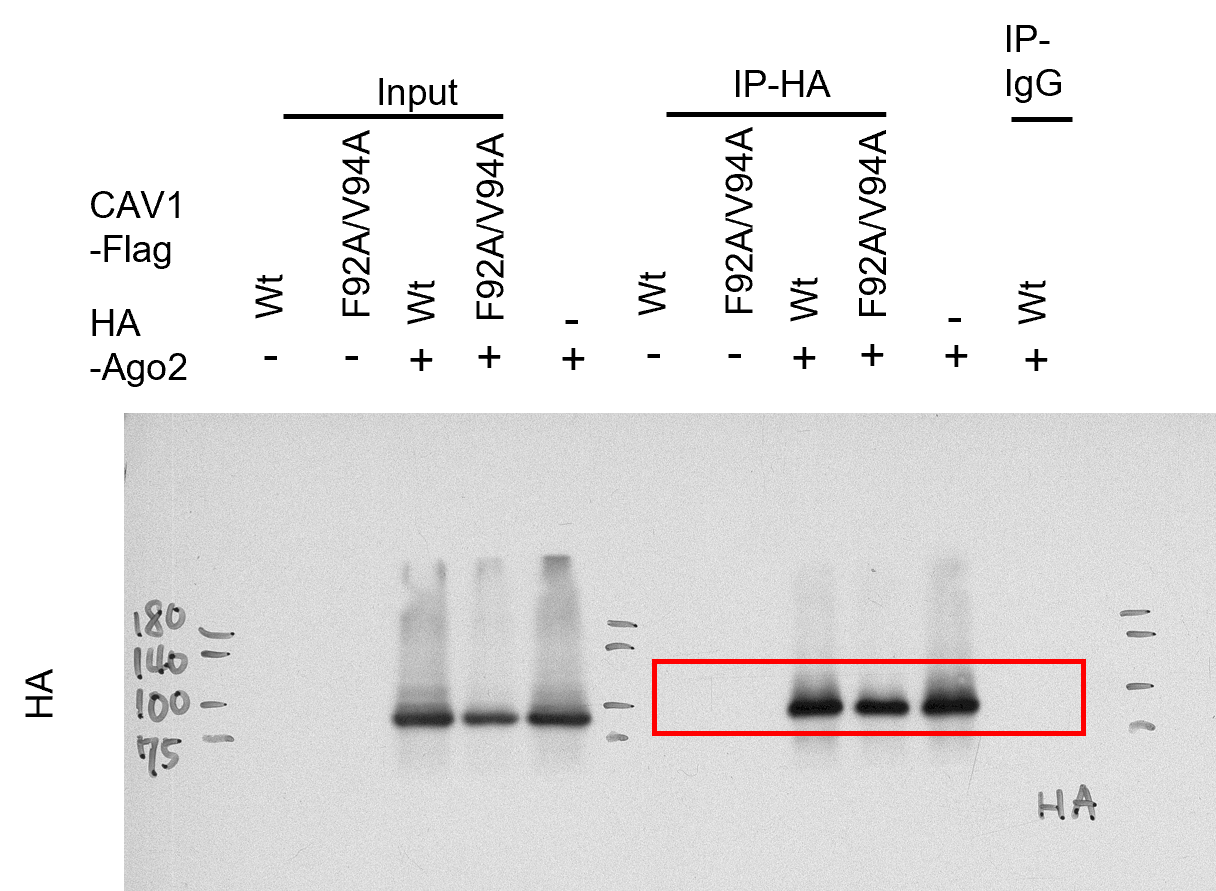

Supplement: Supplementary file 2 — Source data Fig. 1 [file 44319_2024_132_MOESM2_ESM.zip › Figure 1/1E/western IP HA.tif]

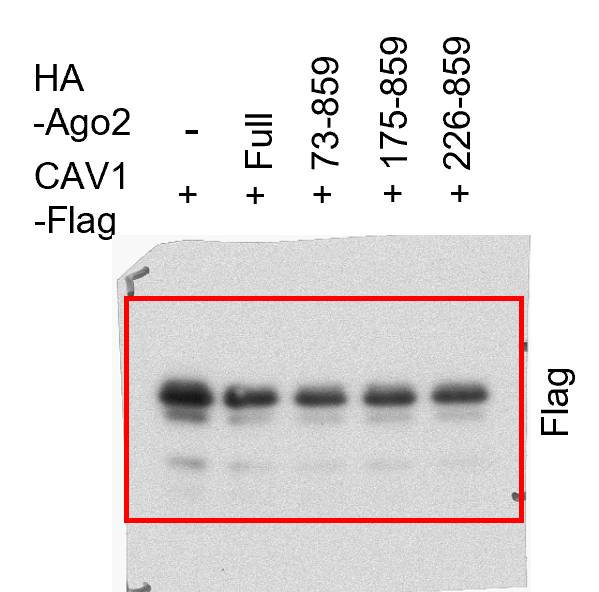

Supplement: Supplementary file 2 — Source data Fig. 1 [file 44319_2024_132_MOESM2_ESM.zip › Figure 1/1F/western Input Flag-CAV1.tif]

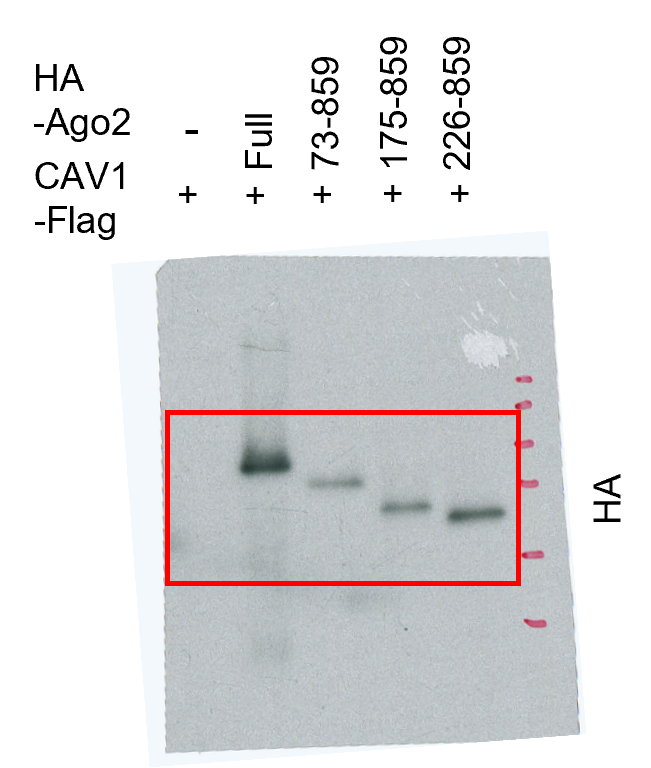

Supplement: Supplementary file 2 — Source data Fig. 1 [file 44319_2024_132_MOESM2_ESM.zip › Figure 1/1F/western Input HA-Ago2.tif]

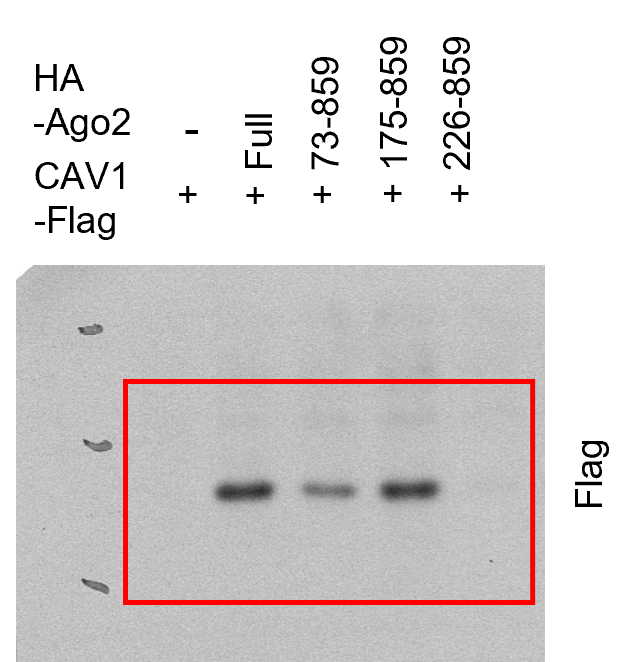

Supplement: Supplementary file 2 — Source data Fig. 1 [file 44319_2024_132_MOESM2_ESM.zip › Figure 1/1F/western IP Flag-CAV1.tif]

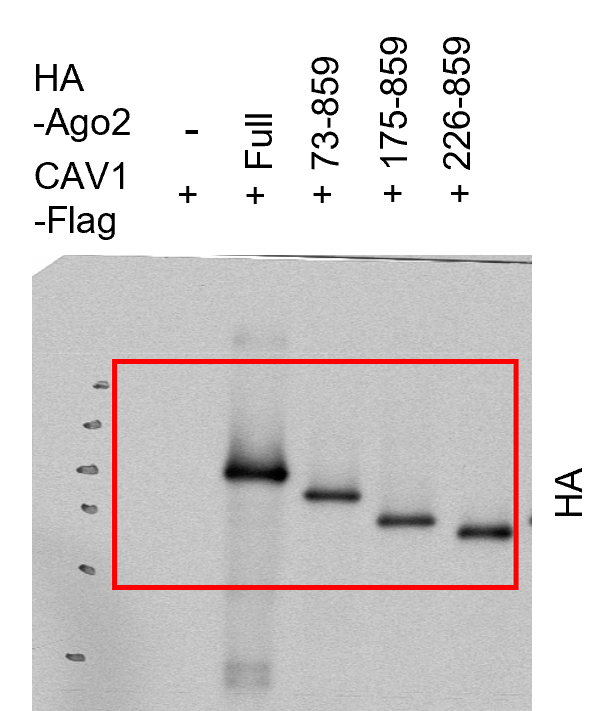

Supplement: Supplementary file 2 — Source data Fig. 1 [file 44319_2024_132_MOESM2_ESM.zip › Figure 1/1F/western IP HA-Ago2.tif]

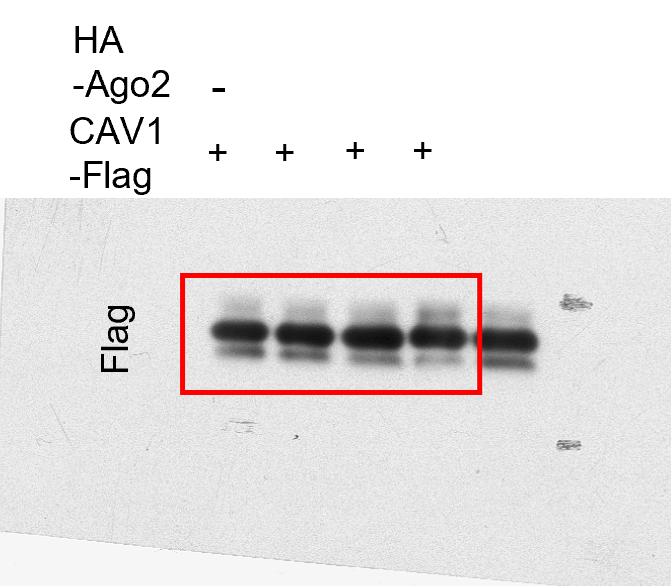

Supplement: Supplementary file 2 — Source data Fig. 1 [file 44319_2024_132_MOESM2_ESM.zip › Figure 1/1G/western Input Flag-CAV1.tif]

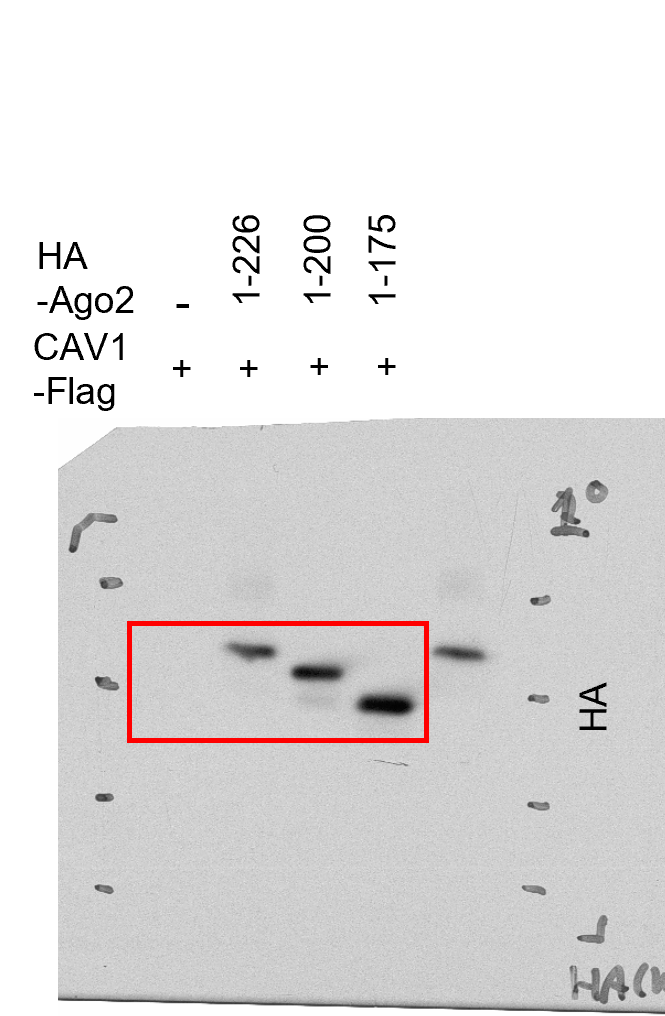

Supplement: Supplementary file 2 — Source data Fig. 1 [file 44319_2024_132_MOESM2_ESM.zip › Figure 1/1G/western Input HA-Ago2.tif]

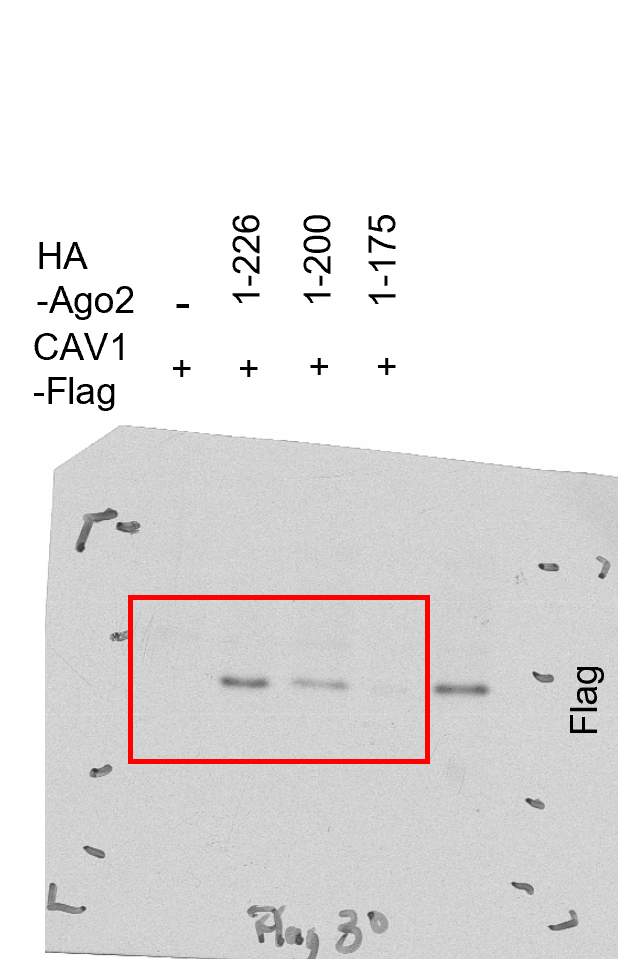

Supplement: Supplementary file 2 — Source data Fig. 1 [file 44319_2024_132_MOESM2_ESM.zip › Figure 1/1G/western IP Flag-CAV1.tif]

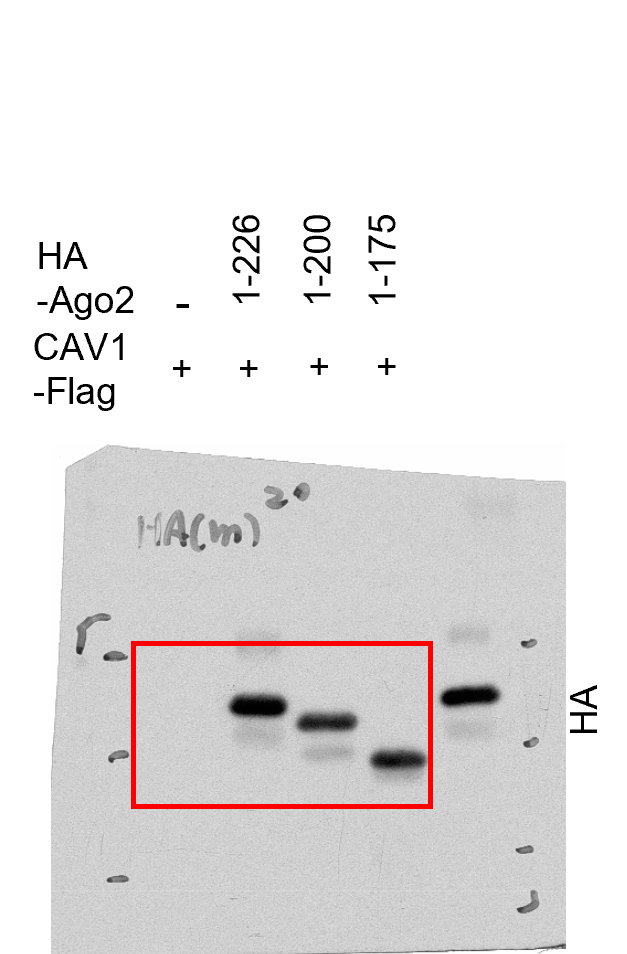

Supplement: Supplementary file 2 — Source data Fig. 1 [file 44319_2024_132_MOESM2_ESM.zip › Figure 1/1G/western IP HA-Ago2.tif]

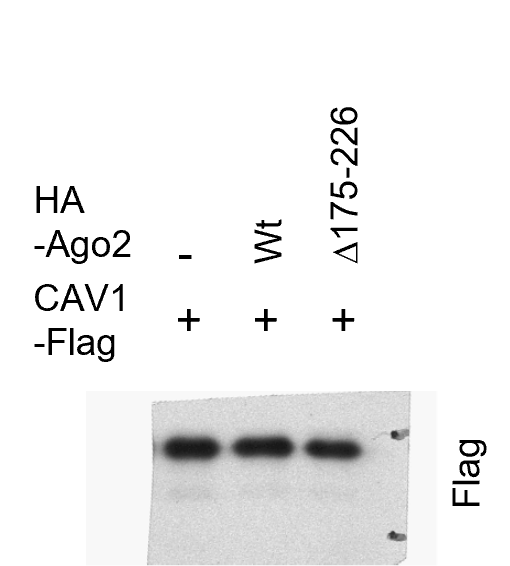

Supplement: Supplementary file 2 — Source data Fig. 1 [file 44319_2024_132_MOESM2_ESM.zip › Figure 1/1H/western input Flag-CAV1-2.tif]

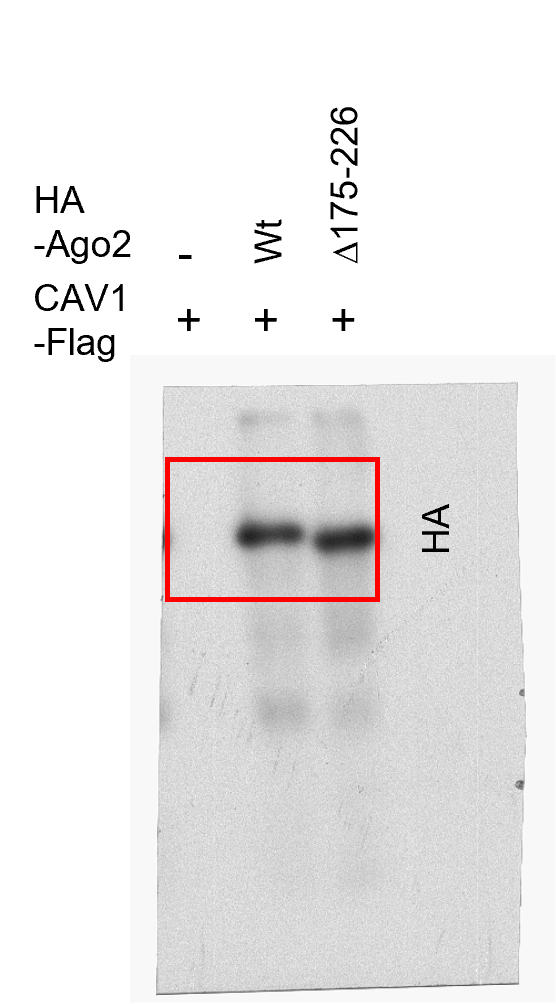

Supplement: Supplementary file 2 — Source data Fig. 1 [file 44319_2024_132_MOESM2_ESM.zip › Figure 1/1H/western input HA-Ago2.tif]

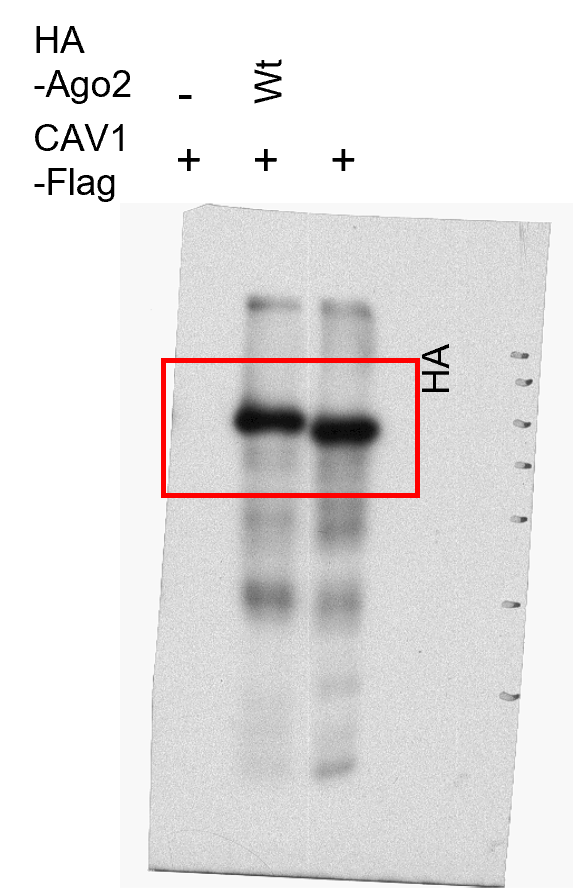

Supplement: Supplementary file 2 — Source data Fig. 1 [file 44319_2024_132_MOESM2_ESM.zip › Figure 1/1H/western input HA-Ago2-2.tif]

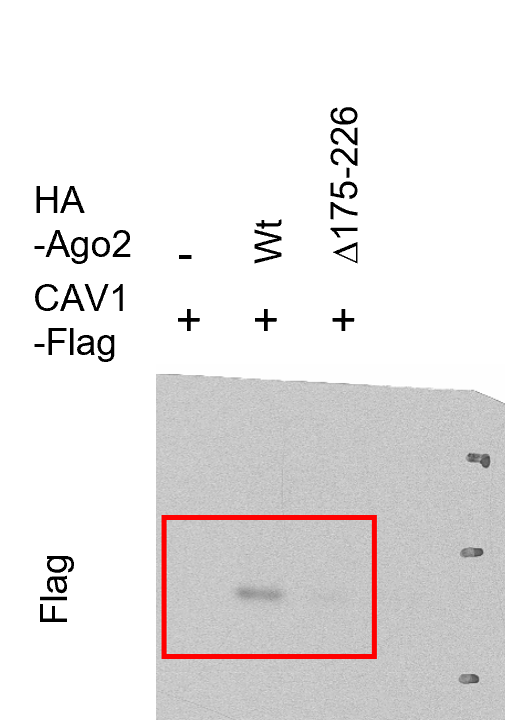

Supplement: Supplementary file 2 — Source data Fig. 1 [file 44319_2024_132_MOESM2_ESM.zip › Figure 1/1H/western IP Flag-CAV1.tif]

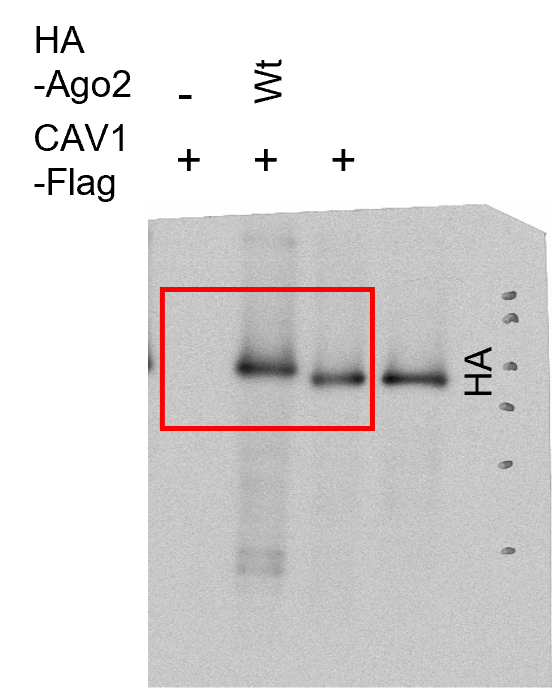

Supplement: Supplementary file 2 — Source data Fig. 1 [file 44319_2024_132_MOESM2_ESM.zip › Figure 1/1H/western IP HA-Ago2.tif]

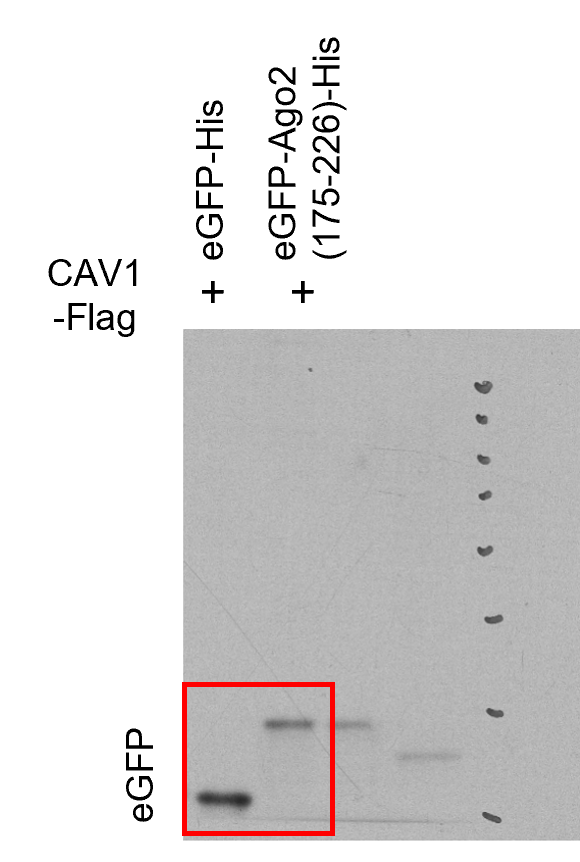

Supplement: Supplementary file 2 — Source data Fig. 1 [file 44319_2024_132_MOESM2_ESM.zip › Figure 1/1I/western input eGFP.tif]

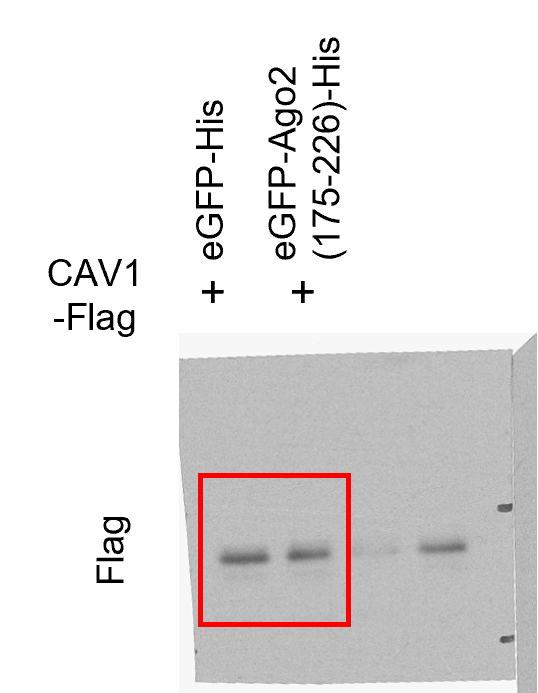

Supplement: Supplementary file 2 — Source data Fig. 1 [file 44319_2024_132_MOESM2_ESM.zip › Figure 1/1I/western input Flag-CAV1.tif]

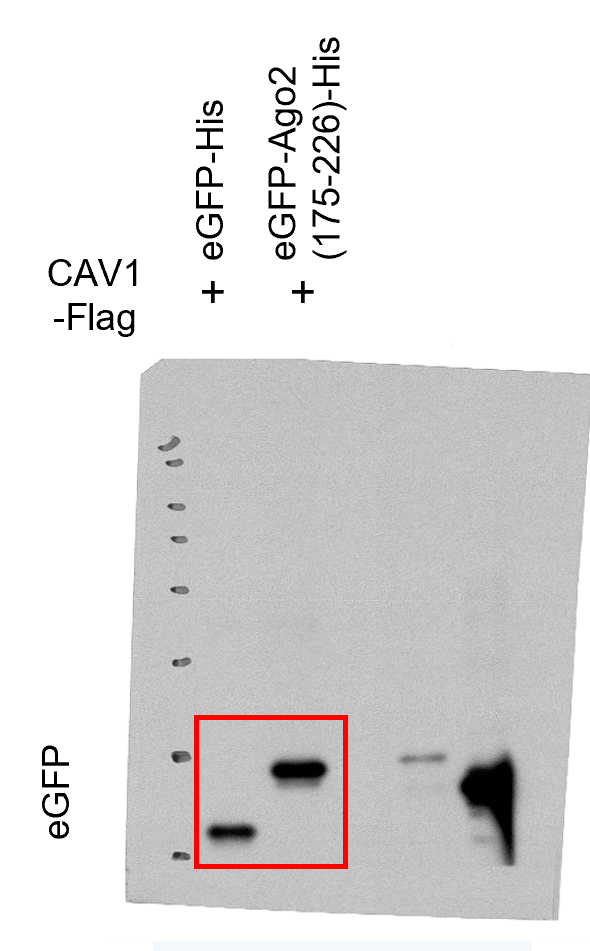

Supplement: Supplementary file 2 — Source data Fig. 1 [file 44319_2024_132_MOESM2_ESM.zip › Figure 1/1I/western IP eGFP.tif]

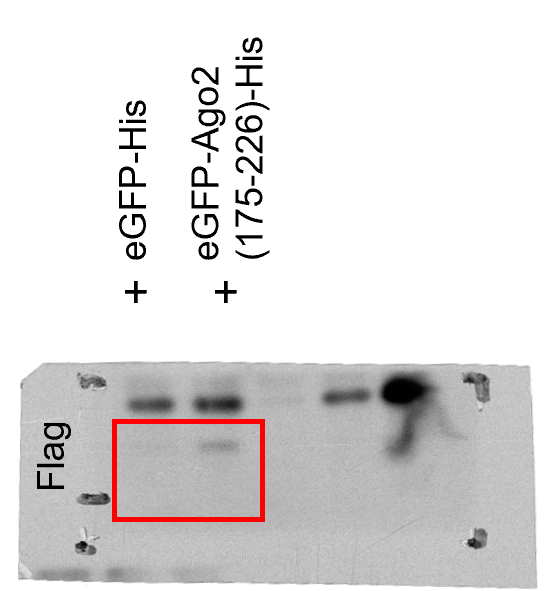

Supplement: Supplementary file 2 — Source data Fig. 1 [file 44319_2024_132_MOESM2_ESM.zip › Figure 1/1I/western IP Flag-CAV1.tif]

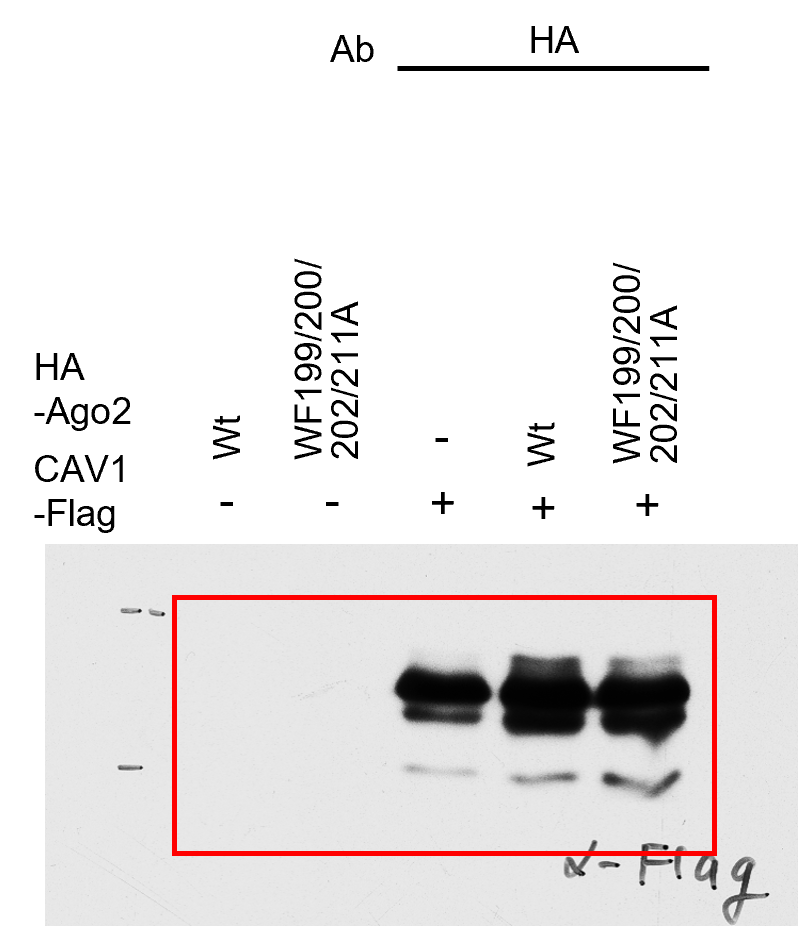

Supplement: Supplementary file 2 — Source data Fig. 1 [file 44319_2024_132_MOESM2_ESM.zip › Figure 1/1K/western input Flag-CAV1.tif]

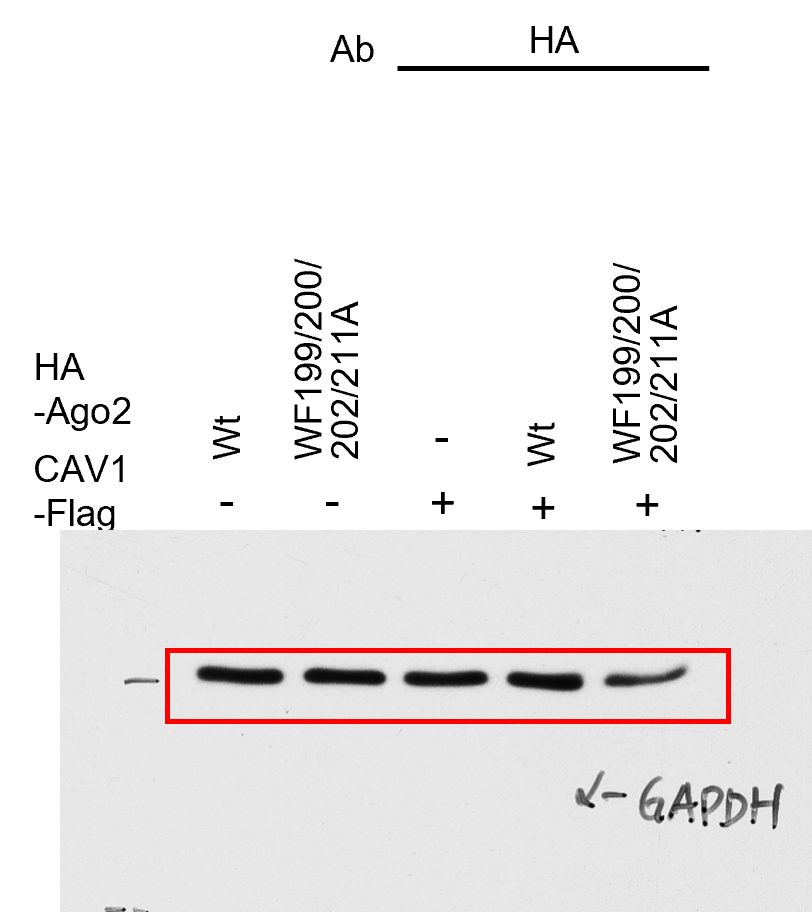

Supplement: Supplementary file 2 — Source data Fig. 1 [file 44319_2024_132_MOESM2_ESM.zip › Figure 1/1K/western input GAPDH.tif]

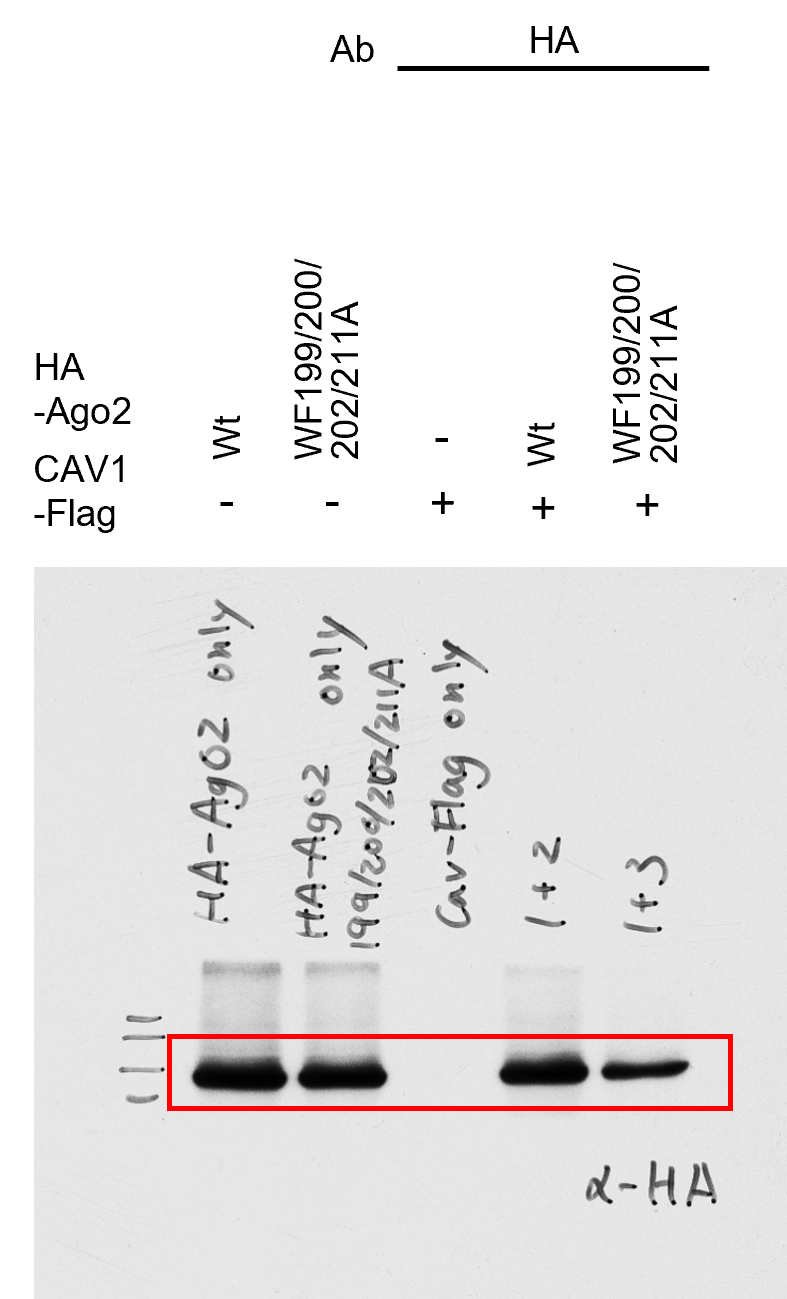

Supplement: Supplementary file 2 — Source data Fig. 1 [file 44319_2024_132_MOESM2_ESM.zip › Figure 1/1K/western input HA-Ago2.tif]

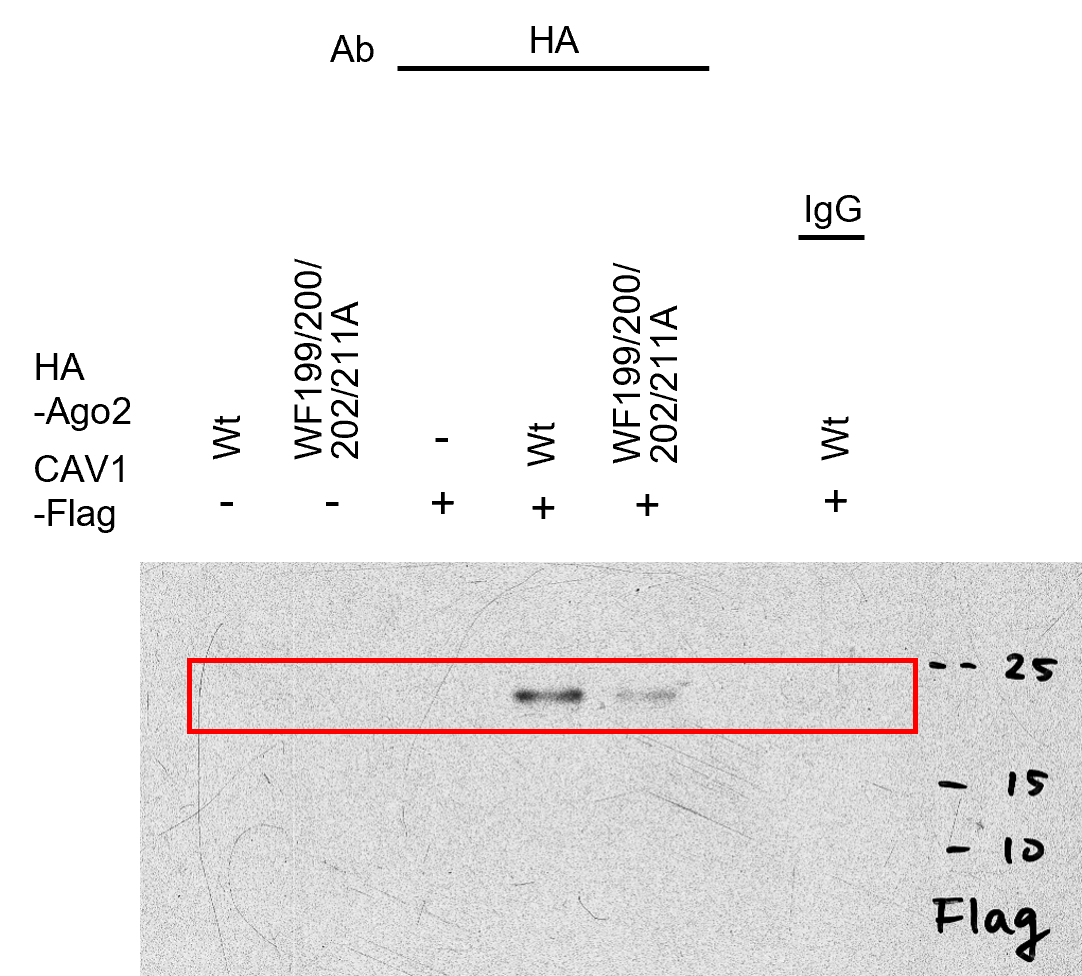

Supplement: Supplementary file 2 — Source data Fig. 1 [file 44319_2024_132_MOESM2_ESM.zip › Figure 1/1K/western IP Flag-CAV1.tif]

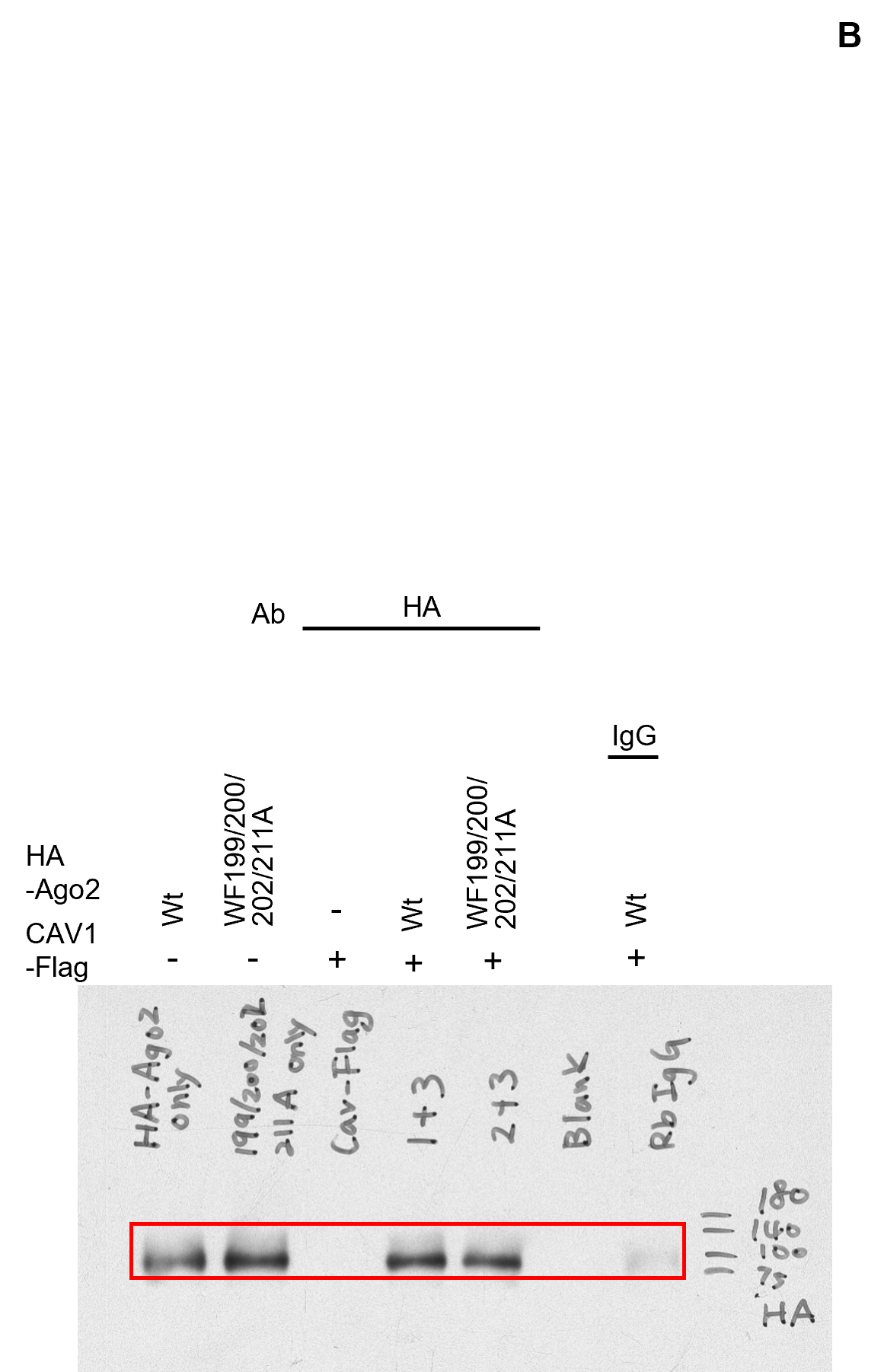

Supplement: Supplementary file 2 — Source data Fig. 1 [file 44319_2024_132_MOESM2_ESM.zip › Figure 1/1K/western IP HA-Ago2.tif]

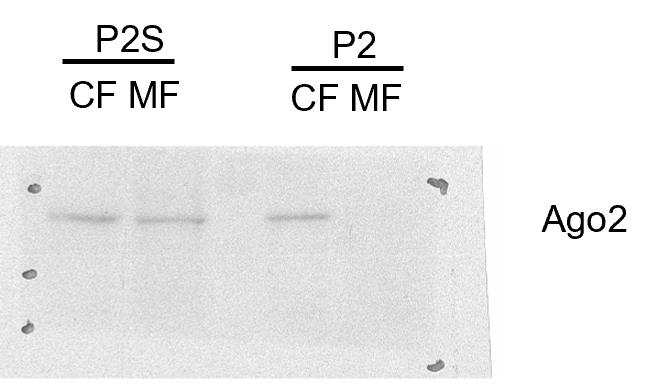

Supplement: Supplementary file 3 — Source data Fig. 2 [file 44319_2024_132_MOESM3_ESM.zip › Figure 2/2A/F2Ai/western Ago2.tif]

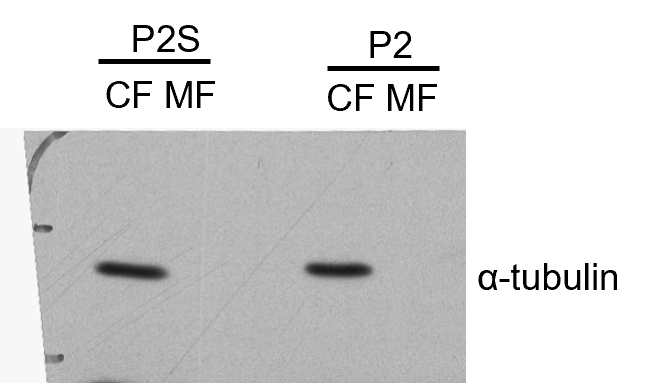

Supplement: Supplementary file 3 — Source data Fig. 2 [file 44319_2024_132_MOESM3_ESM.zip › Figure 2/2A/F2Ai/western alpha-tubulin.tif]

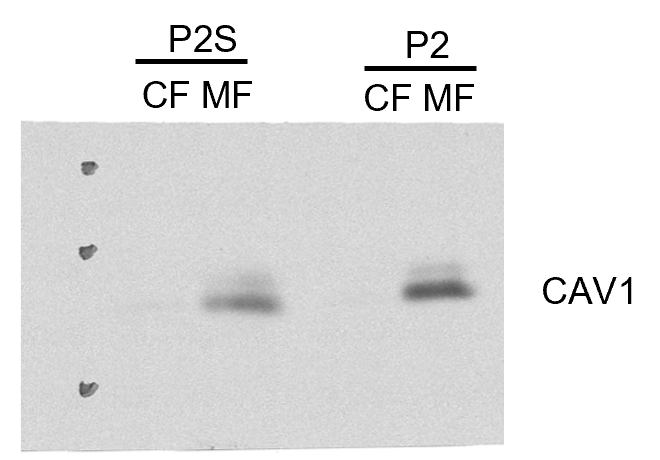

Supplement: Supplementary file 3 — Source data Fig. 2 [file 44319_2024_132_MOESM3_ESM.zip › Figure 2/2A/F2Ai/western CAV1.tif]

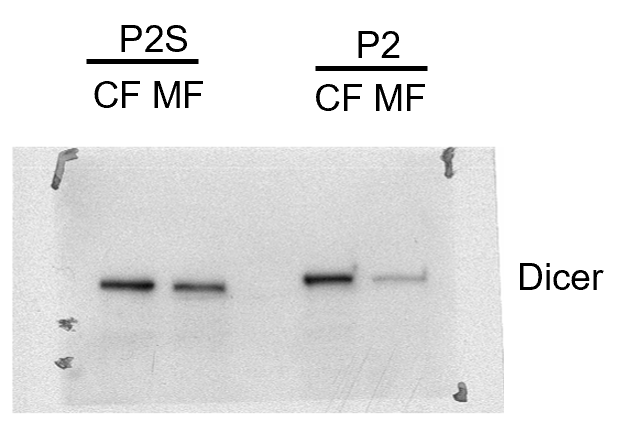

Supplement: Supplementary file 3 — Source data Fig. 2 [file 44319_2024_132_MOESM3_ESM.zip › Figure 2/2A/F2Ai/western Dicer.tif]

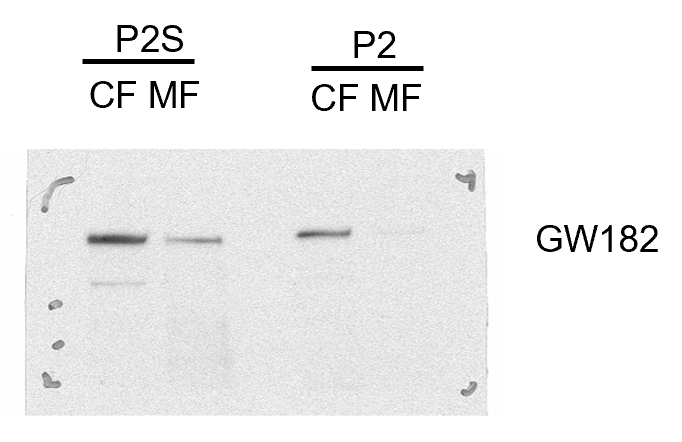

Supplement: Supplementary file 3 — Source data Fig. 2 [file 44319_2024_132_MOESM3_ESM.zip › Figure 2/2A/F2Ai/western GW182.tif]

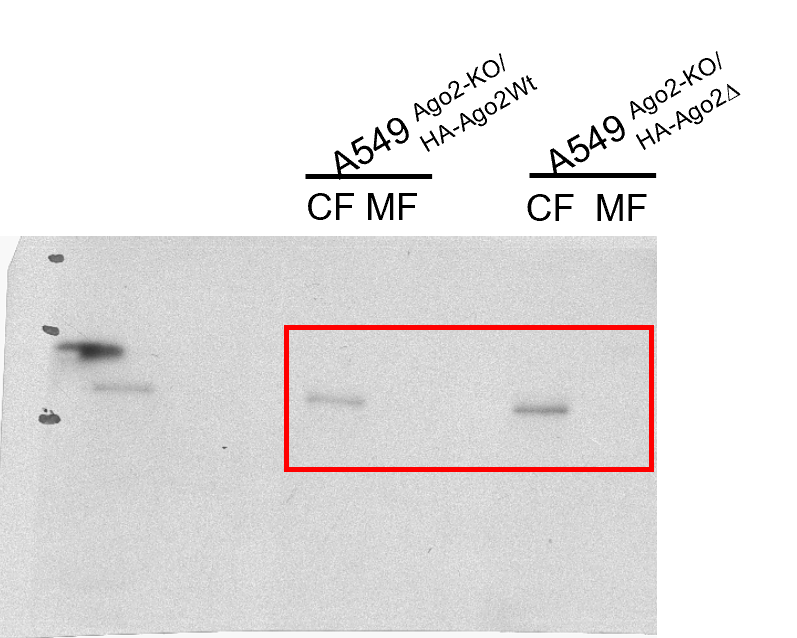

Supplement: Supplementary file 3 — Source data Fig. 2 [file 44319_2024_132_MOESM3_ESM.zip › Figure 2/2B/2Bi/western alpha-tubulin.tif]

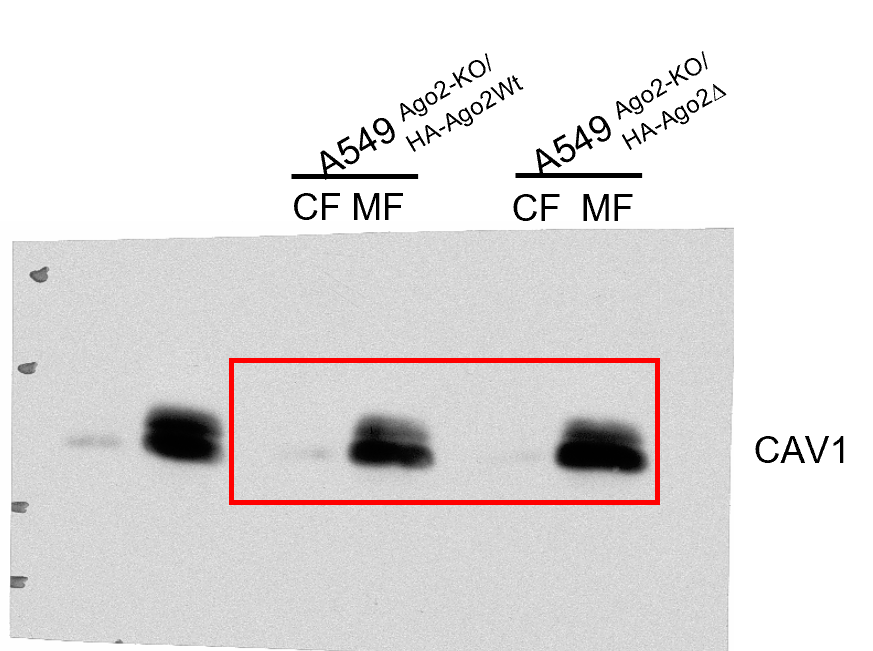

Supplement: Supplementary file 3 — Source data Fig. 2 [file 44319_2024_132_MOESM3_ESM.zip › Figure 2/2B/2Bi/western CAV1.tif]

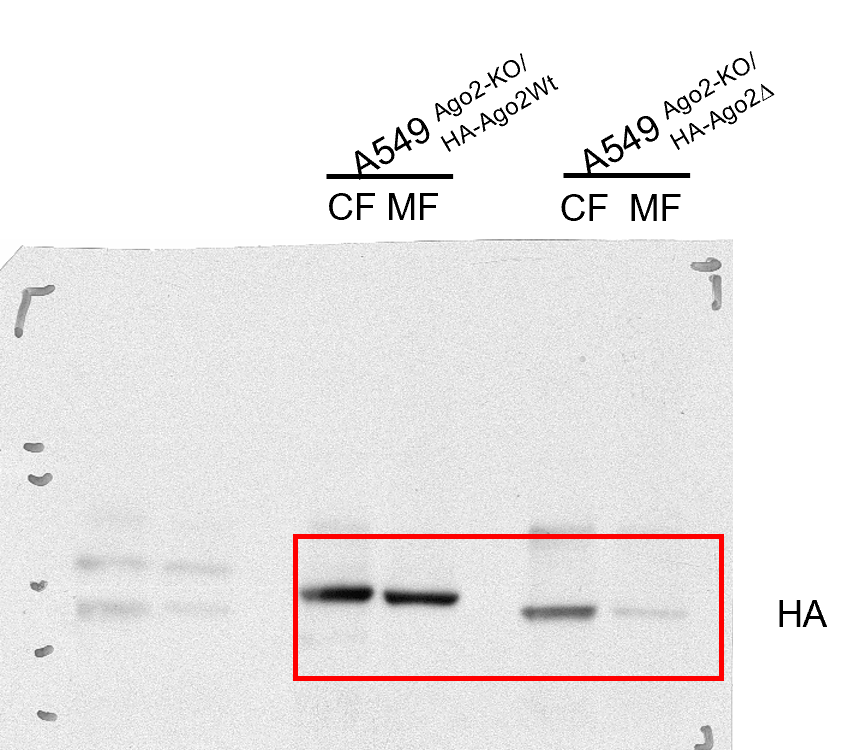

Supplement: Supplementary file 3 — Source data Fig. 2 [file 44319_2024_132_MOESM3_ESM.zip › Figure 2/2B/2Bi/western HA-Ago2.tif]

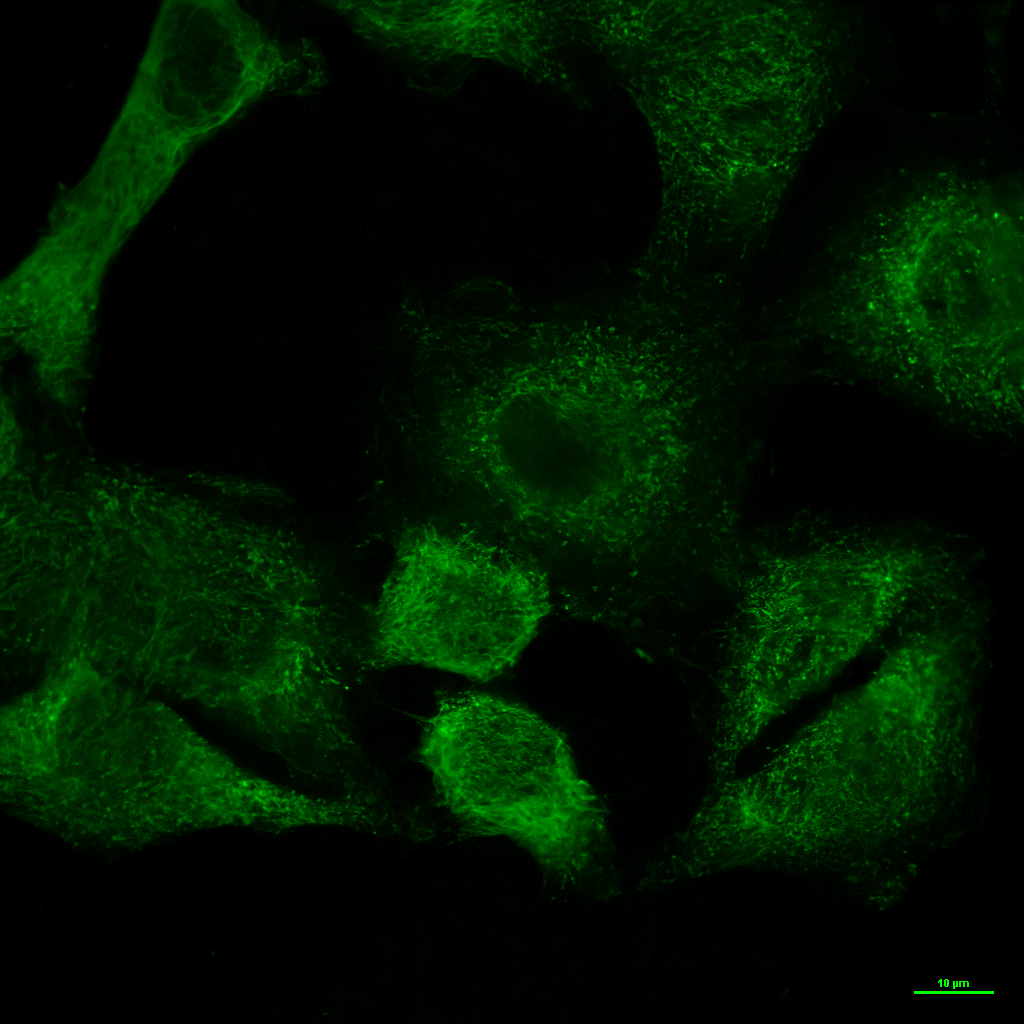

Supplement: Supplementary file 3 — Source data Fig. 2 [file 44319_2024_132_MOESM3_ESM.zip › Figure 2/2C/A549 Ago2.tif]

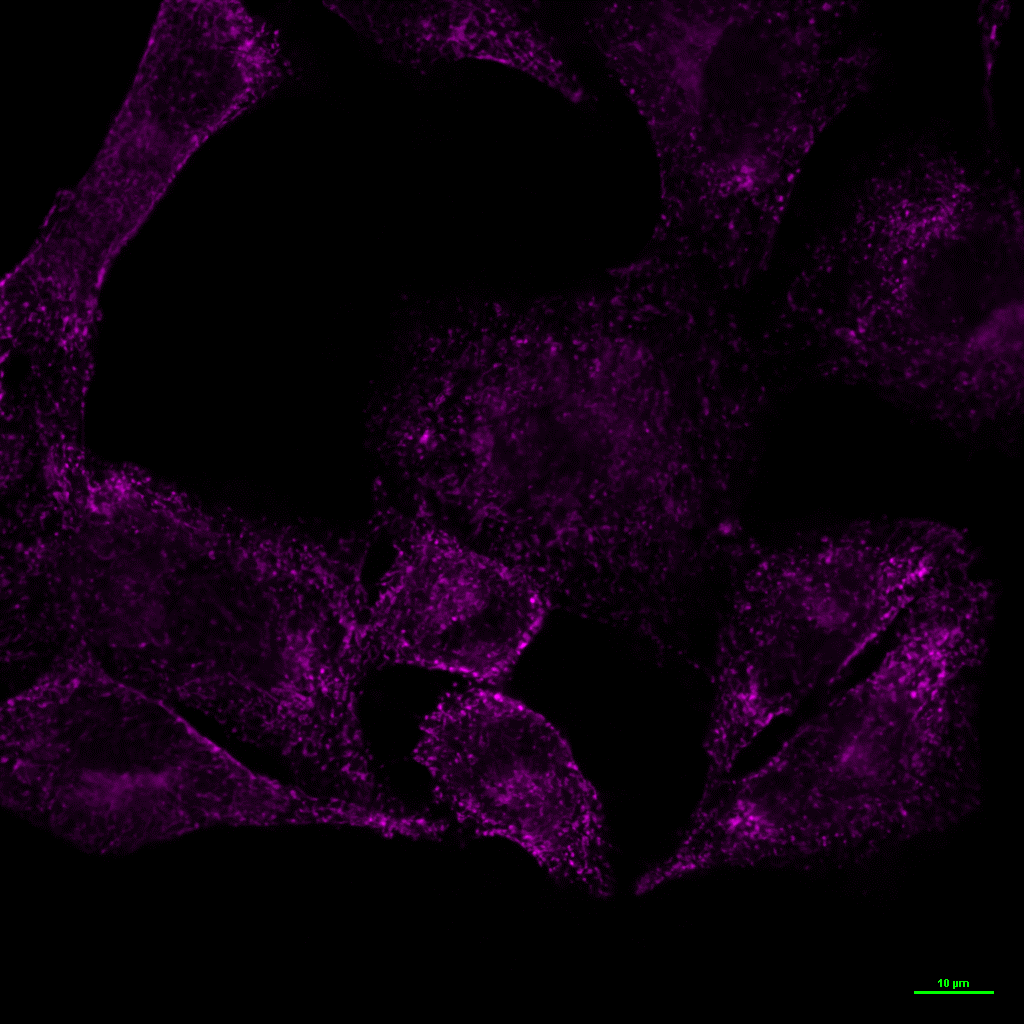

Supplement: Supplementary file 3 — Source data Fig. 2 [file 44319_2024_132_MOESM3_ESM.zip › Figure 2/2C/A549 CAV1.tif]

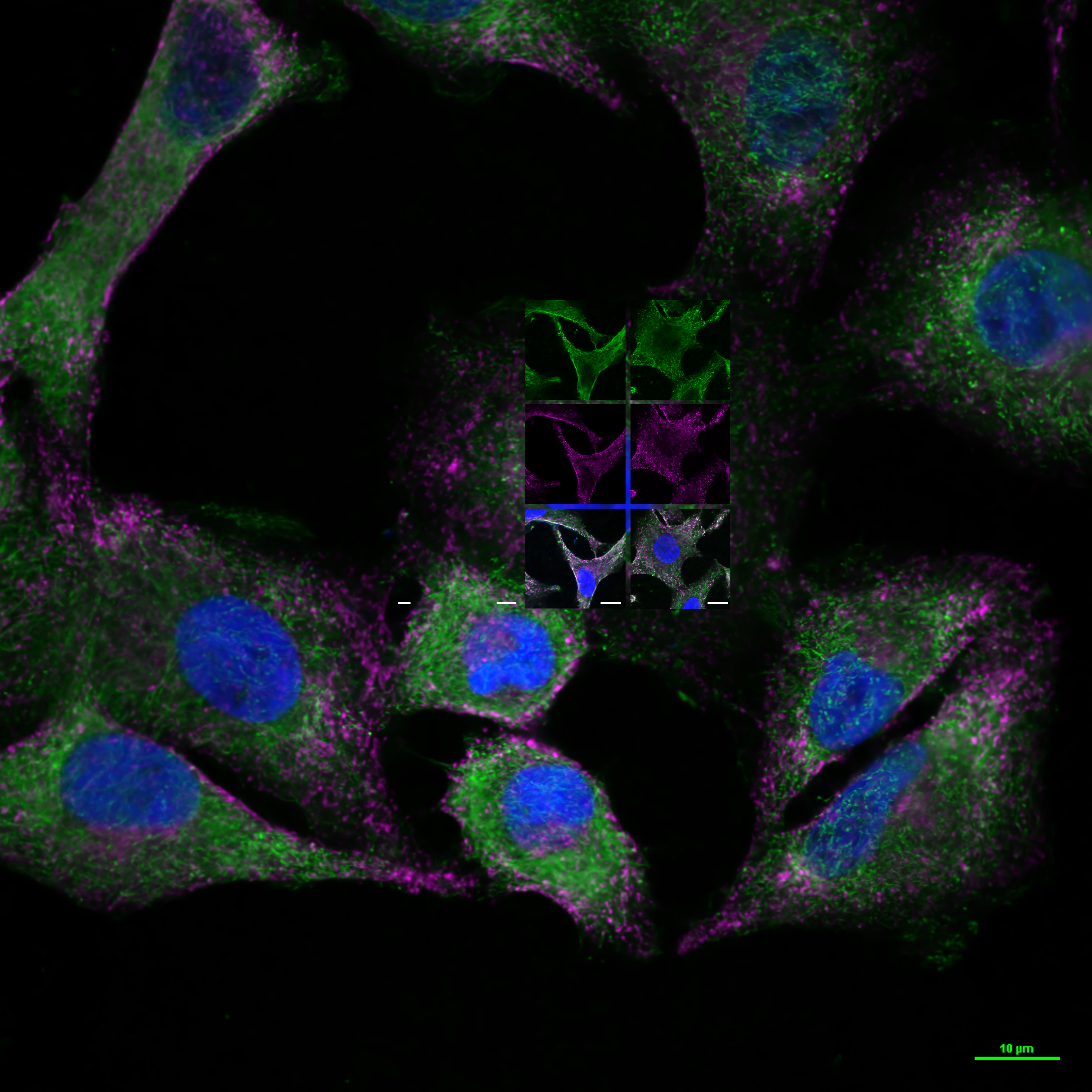

Supplement: Supplementary file 3 — Source data Fig. 2 [file 44319_2024_132_MOESM3_ESM.zip › Figure 2/2C/A549 Merged.tif]

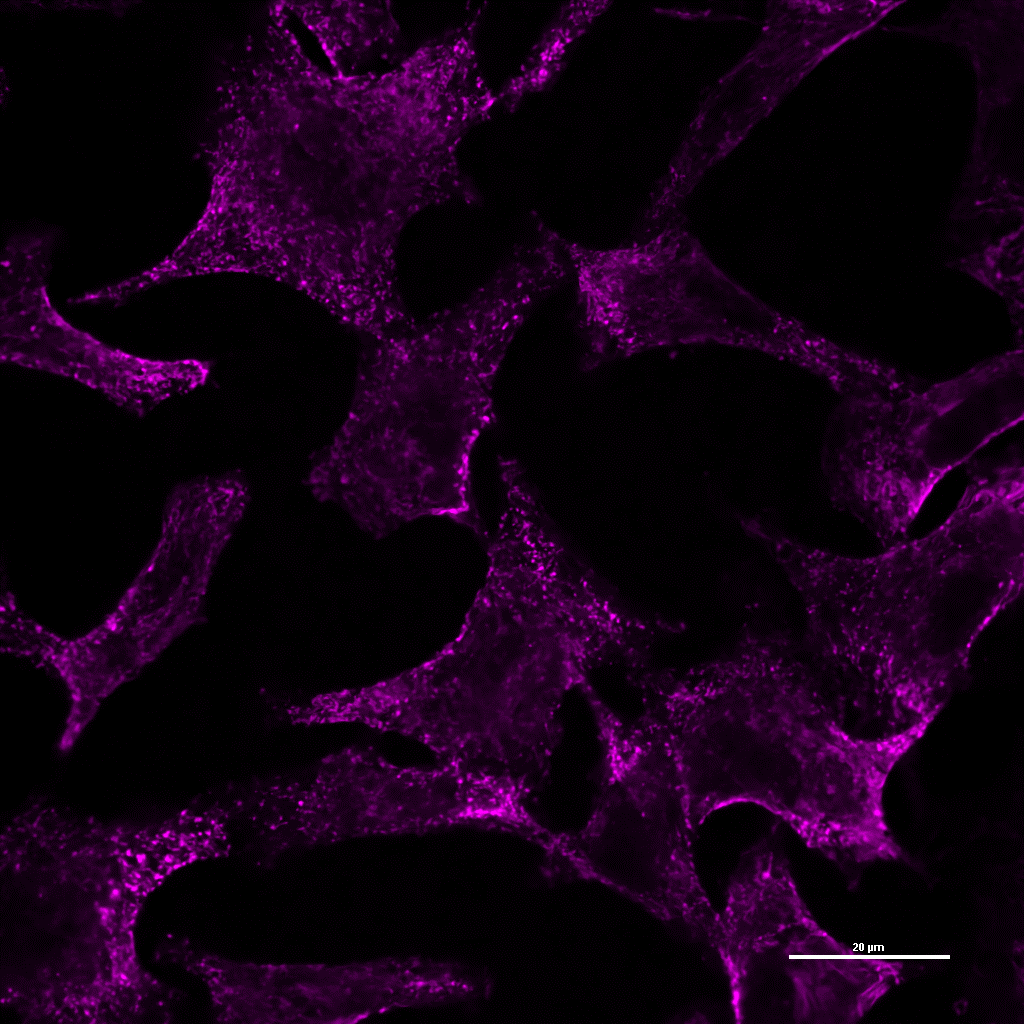

Supplement: Supplementary file 3 — Source data Fig. 2 [file 44319_2024_132_MOESM3_ESM.zip › Figure 2/2C/A549-HA-Ago2Dm CAV1.tif]

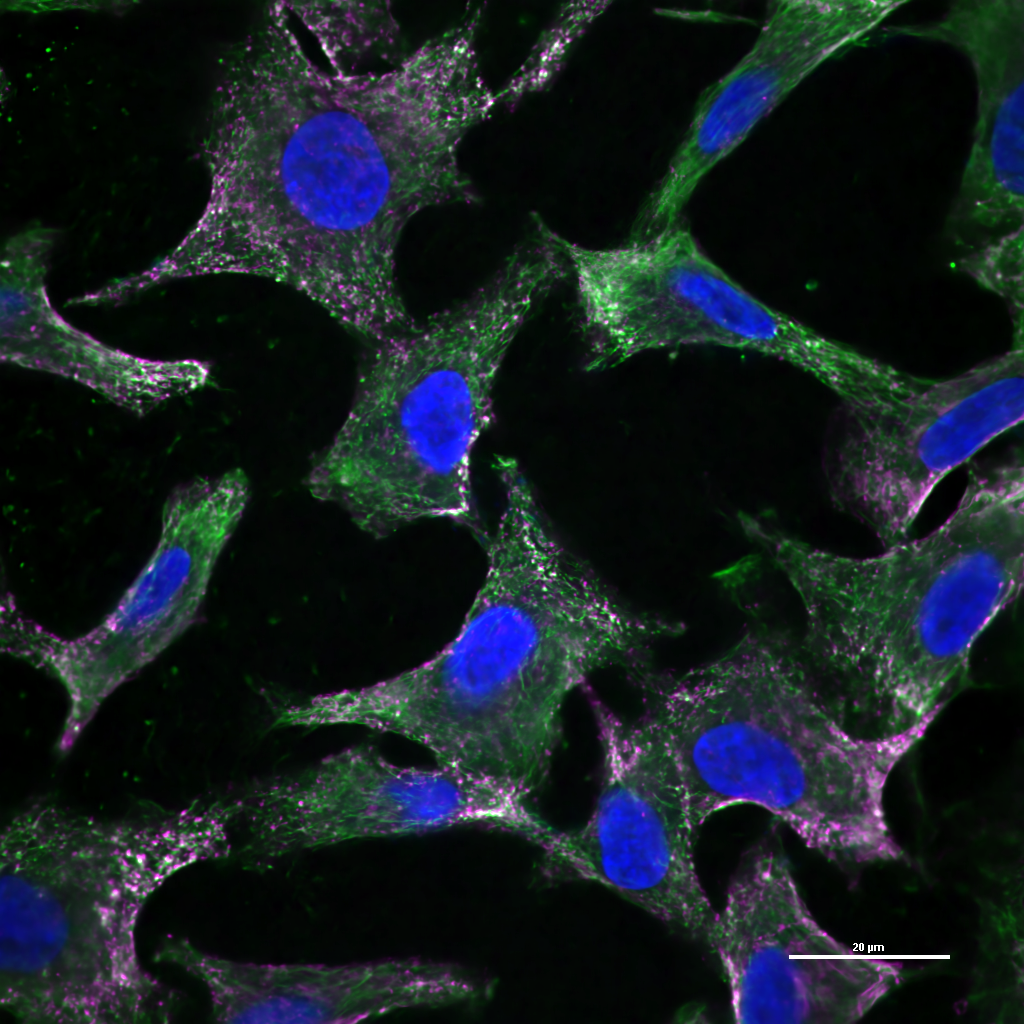

Supplement: Supplementary file 3 — Source data Fig. 2 [file 44319_2024_132_MOESM3_ESM.zip › Figure 2/2C/A549-HA-Ago2Dm Merged.tif]

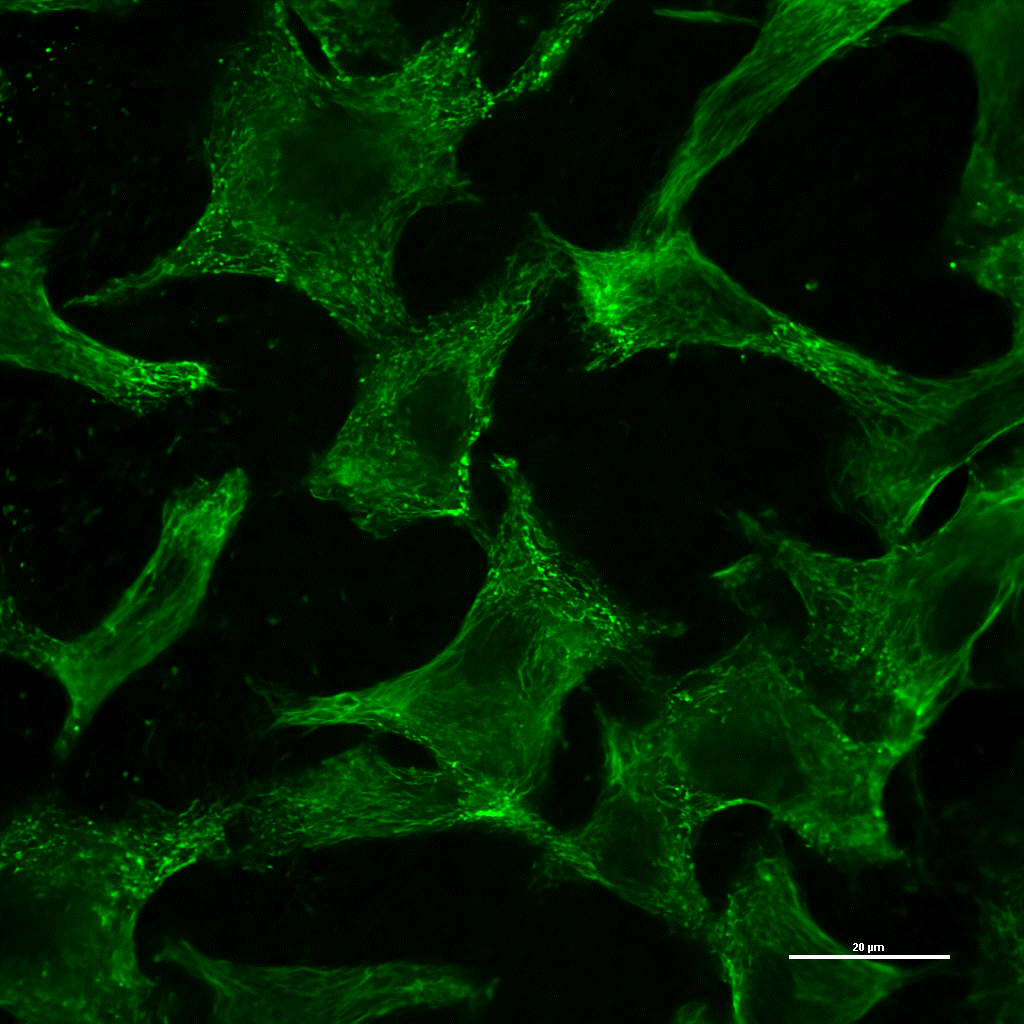

Supplement: Supplementary file 3 — Source data Fig. 2 [file 44319_2024_132_MOESM3_ESM.zip › Figure 2/2C/A549-HA-Ago2WDm Ago2.tif]

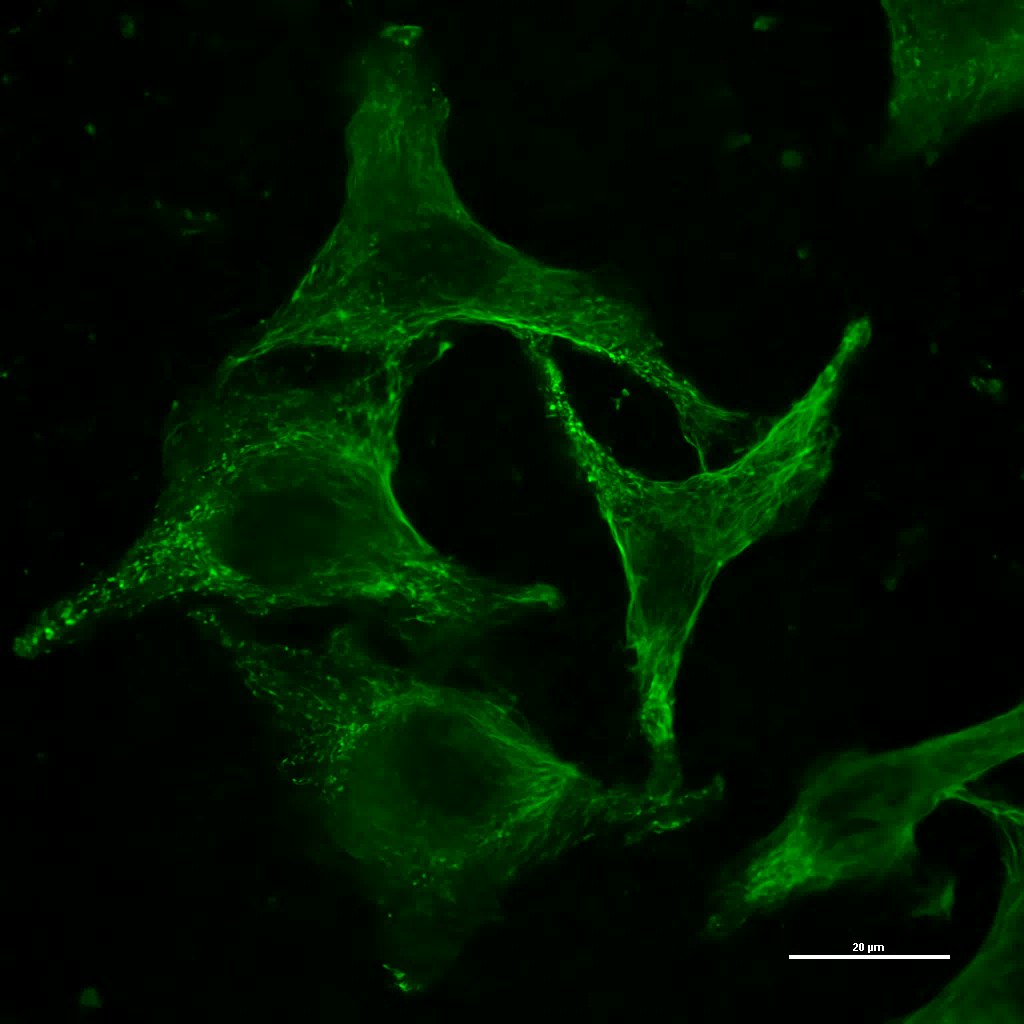

Supplement: Supplementary file 3 — Source data Fig. 2 [file 44319_2024_132_MOESM3_ESM.zip › Figure 2/2C/A549-HA-Ago2Wt Ago2.tif]

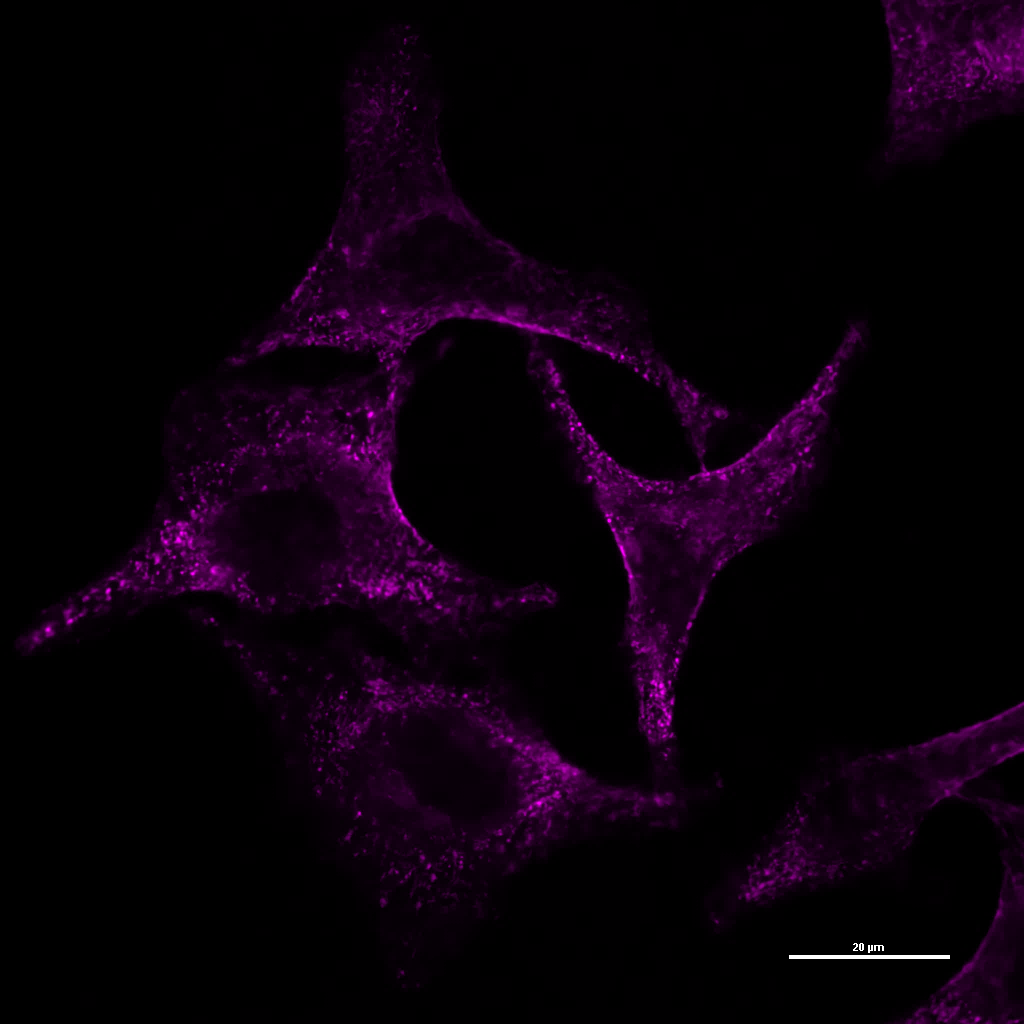

Supplement: Supplementary file 3 — Source data Fig. 2 [file 44319_2024_132_MOESM3_ESM.zip › Figure 2/2C/A549-HA-Ago2Wt CAV1.tif]

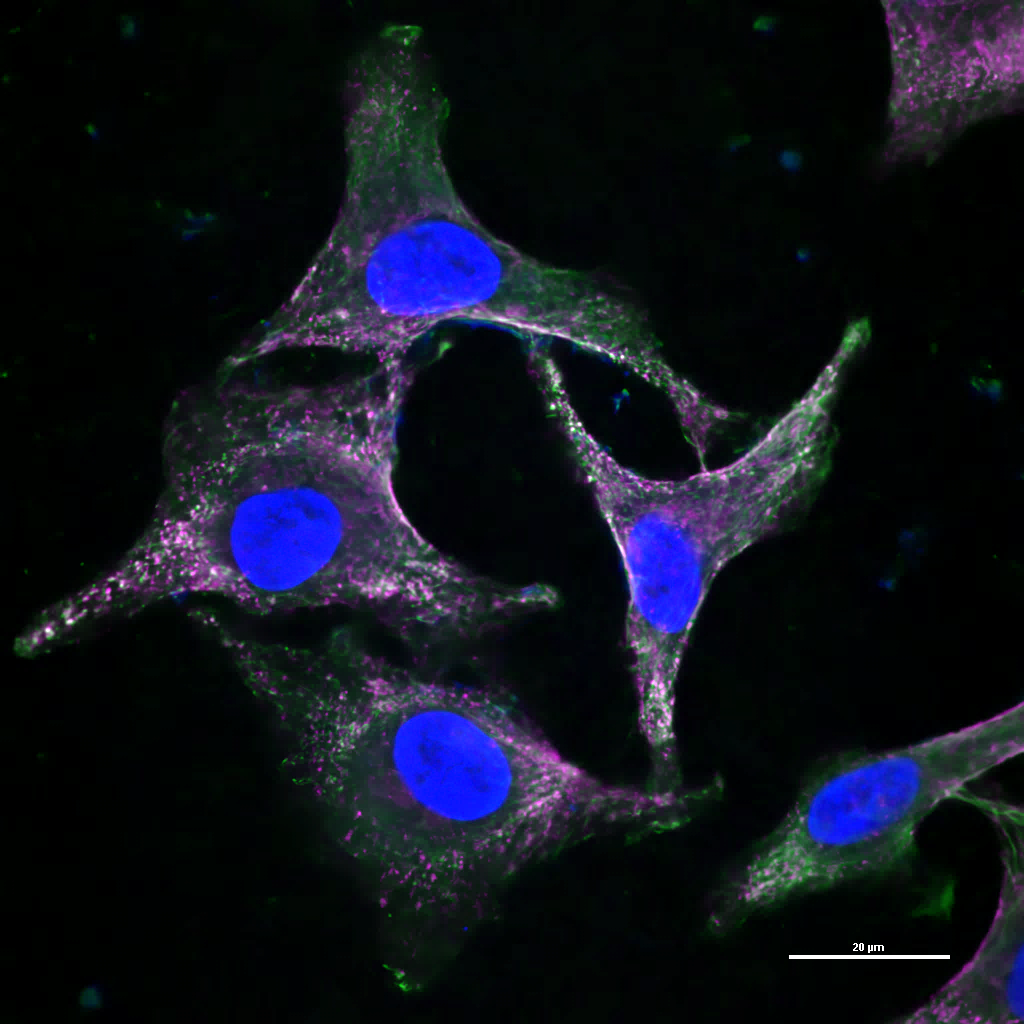

Supplement: Supplementary file 3 — Source data Fig. 2 [file 44319_2024_132_MOESM3_ESM.zip › Figure 2/2C/A549-HA-Ago2Wt Merged.tif]

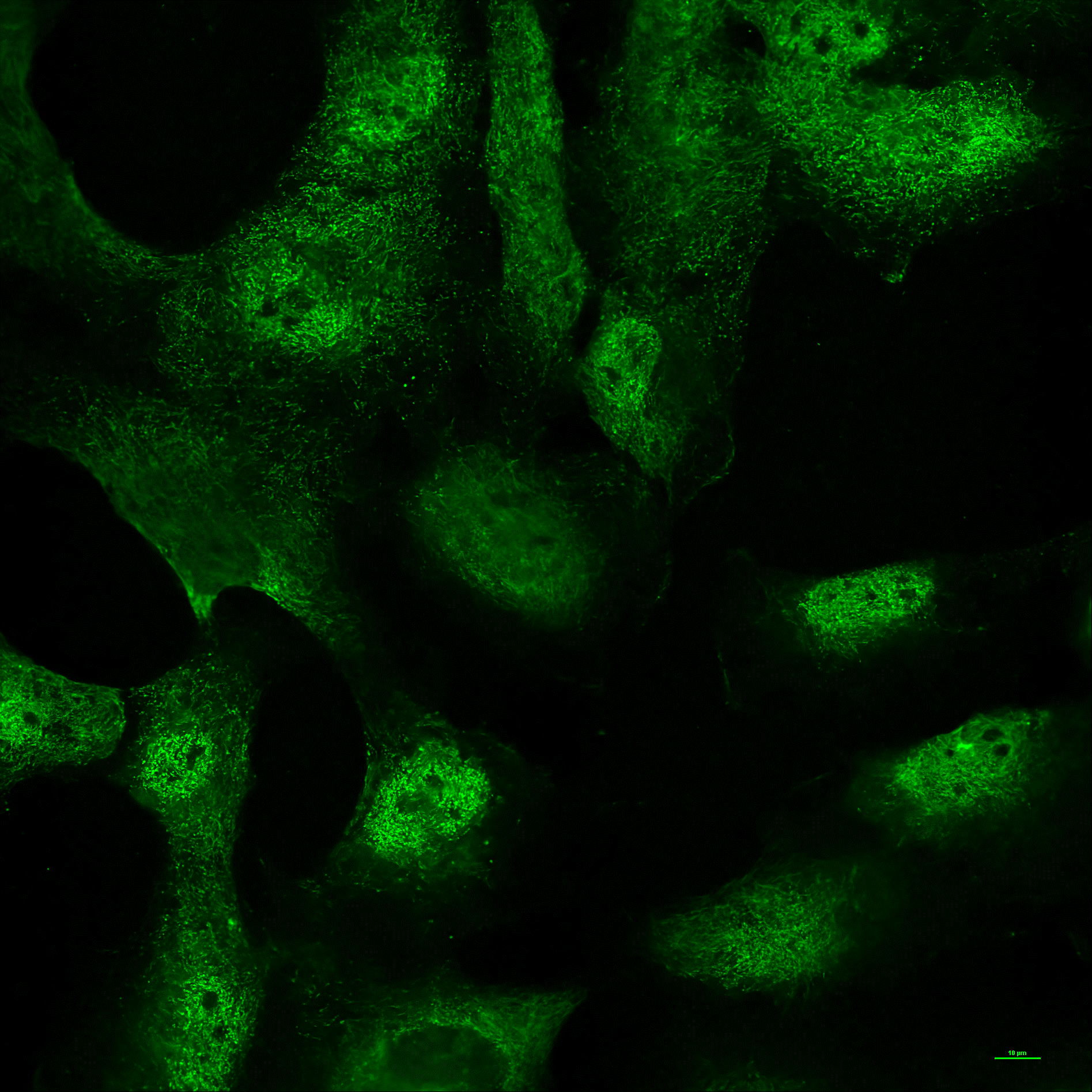

Supplement: Supplementary file 3 — Source data Fig. 2 [file 44319_2024_132_MOESM3_ESM.zip › Figure 2/2C/BEAS-2B Ago2.tif]

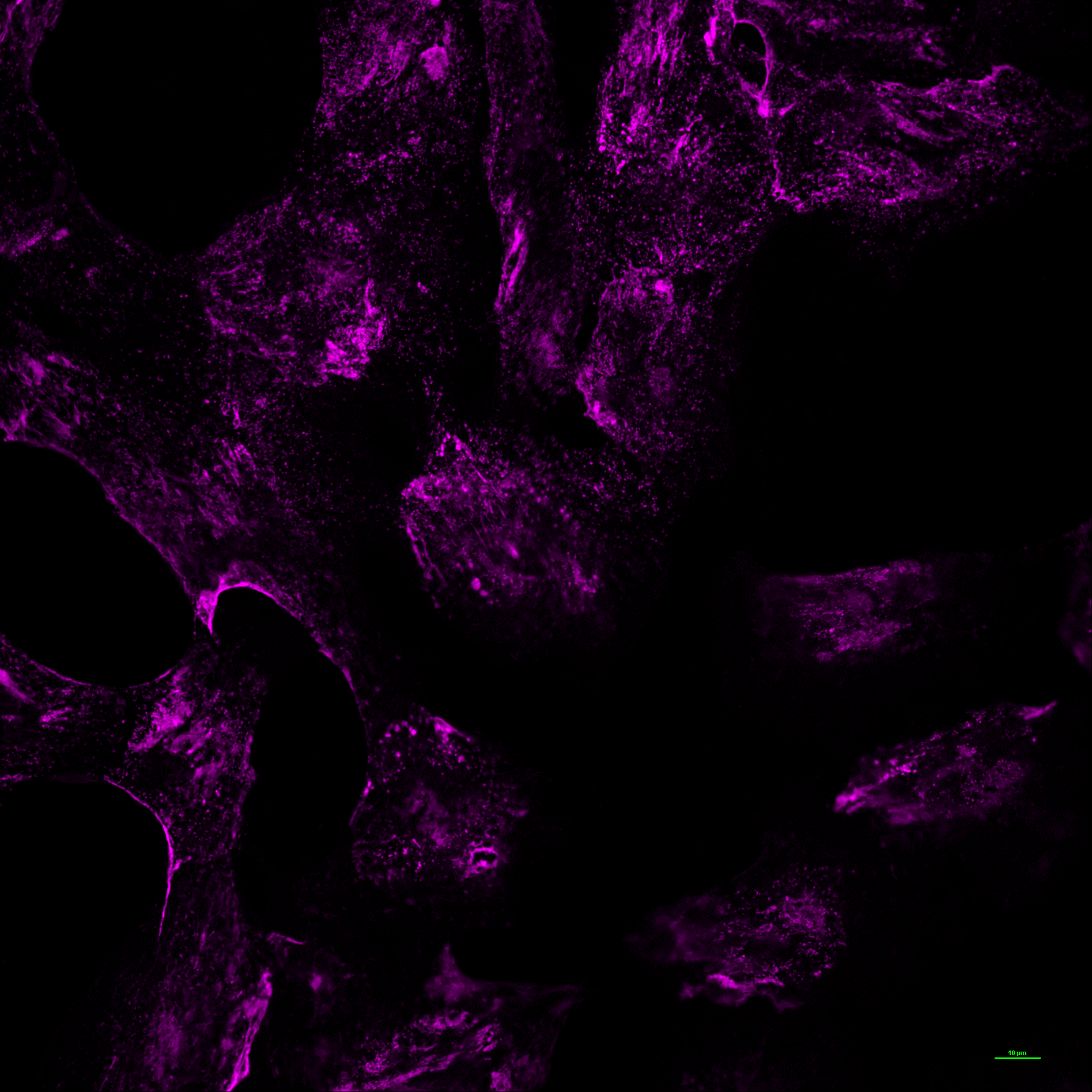

Supplement: Supplementary file 3 — Source data Fig. 2 [file 44319_2024_132_MOESM3_ESM.zip › Figure 2/2C/BEAS-2B CAV1.tif]

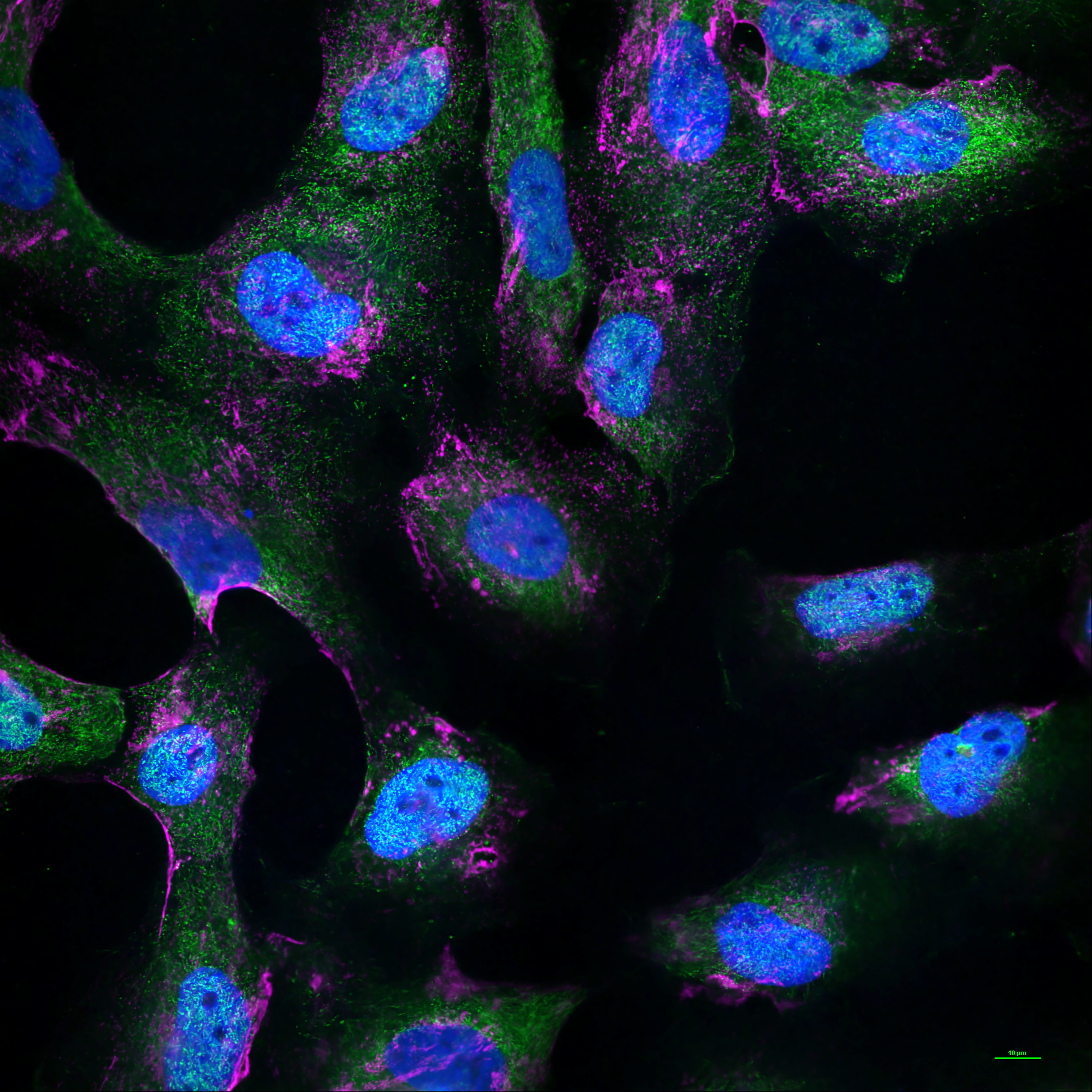

Supplement: Supplementary file 3 — Source data Fig. 2 [file 44319_2024_132_MOESM3_ESM.zip › Figure 2/2C/BEAS-2B Merged.tif]

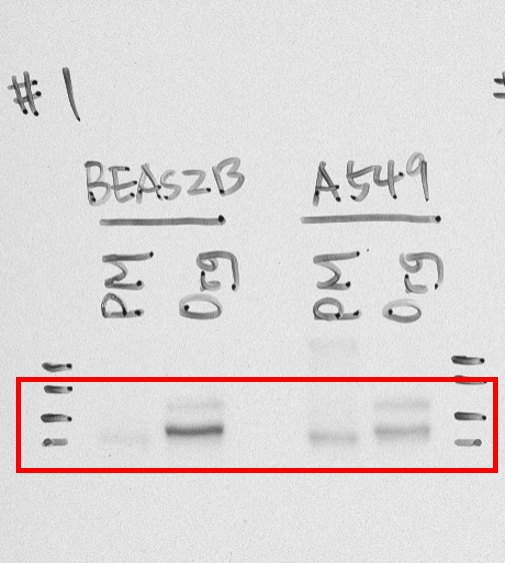

Supplement: Supplementary file 3 — Source data Fig. 2 [file 44319_2024_132_MOESM3_ESM.zip › Figure 2/2E/2Ei/western Ago2.tif]

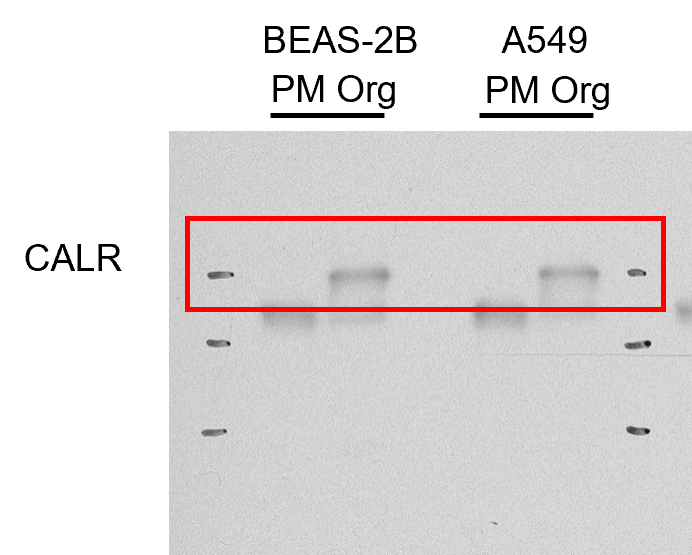

Supplement: Supplementary file 3 — Source data Fig. 2 [file 44319_2024_132_MOESM3_ESM.zip › Figure 2/2E/2Ei/western CALR.tif]

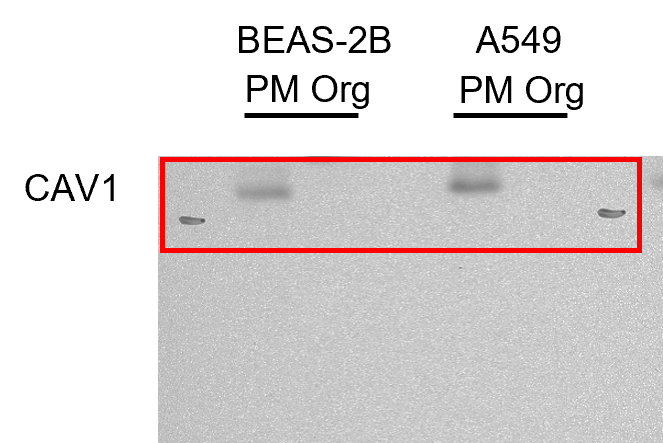

Supplement: Supplementary file 3 — Source data Fig. 2 [file 44319_2024_132_MOESM3_ESM.zip › Figure 2/2E/2Ei/western CAV1.tif]

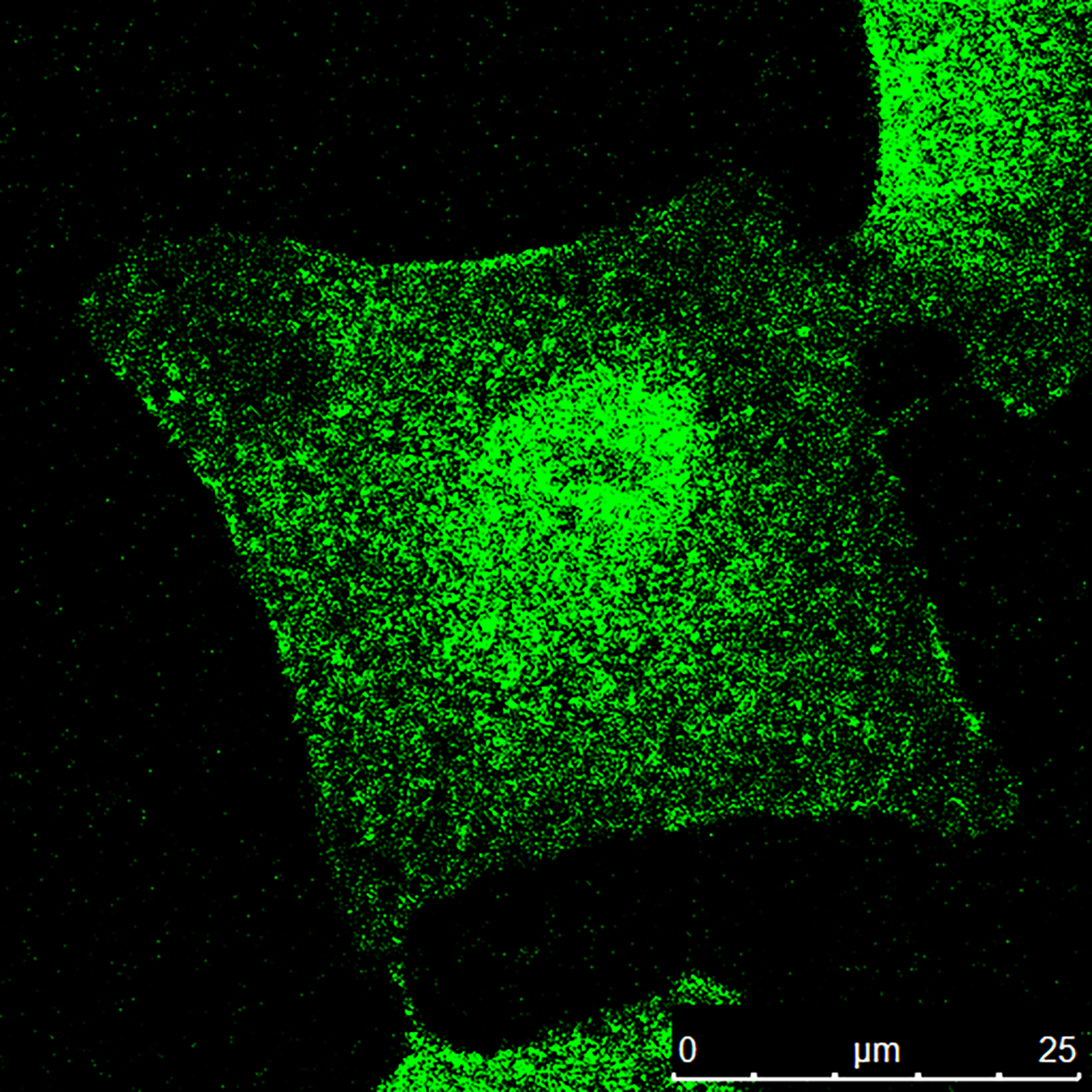

Supplement: Supplementary file 3 — Source data Fig. 2 [file 44319_2024_132_MOESM3_ESM.zip › Figure 2/2F/A549/Ago2.tif]

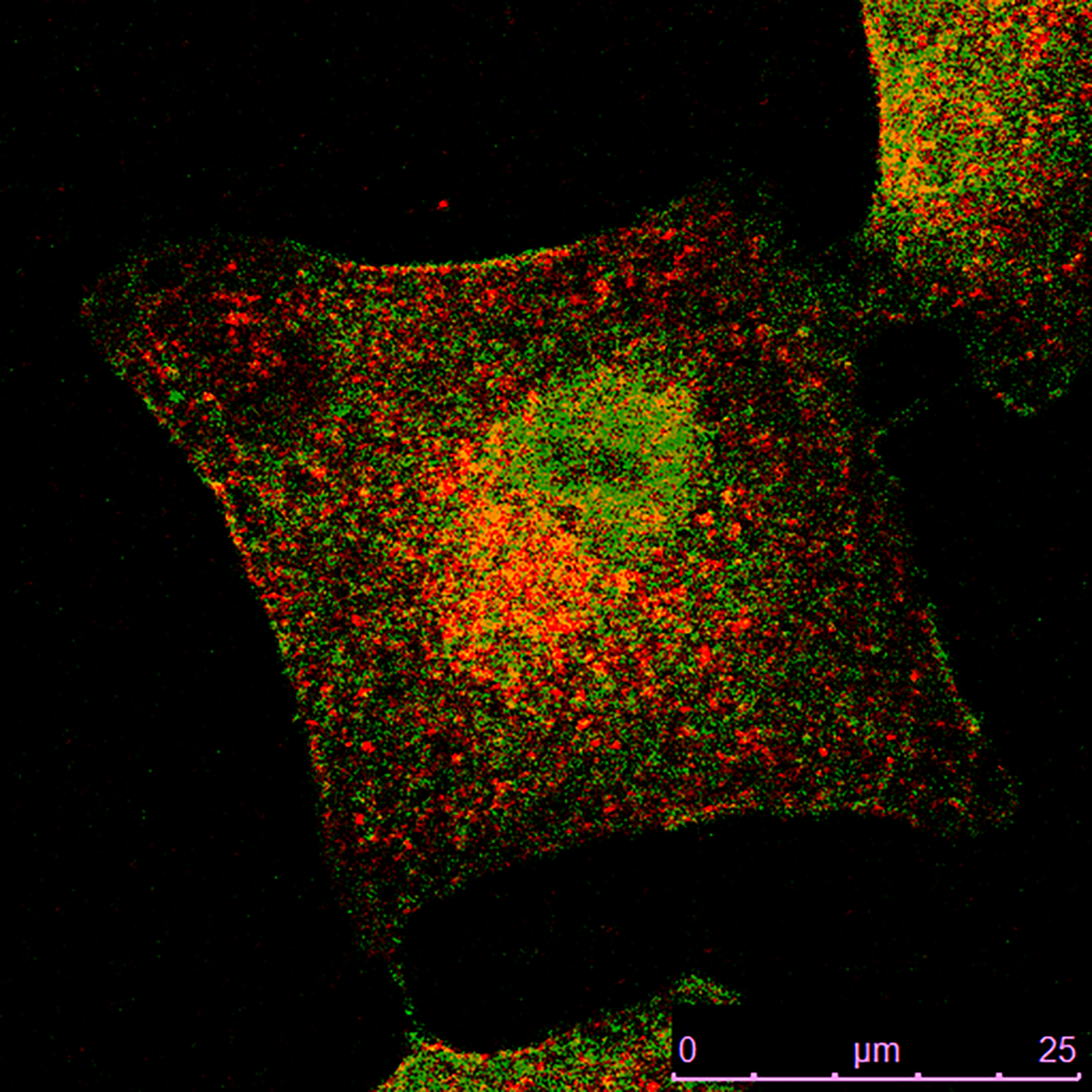

Supplement: Supplementary file 3 — Source data Fig. 2 [file 44319_2024_132_MOESM3_ESM.zip › Figure 2/2F/A549/Ago2+CAV1.tif]

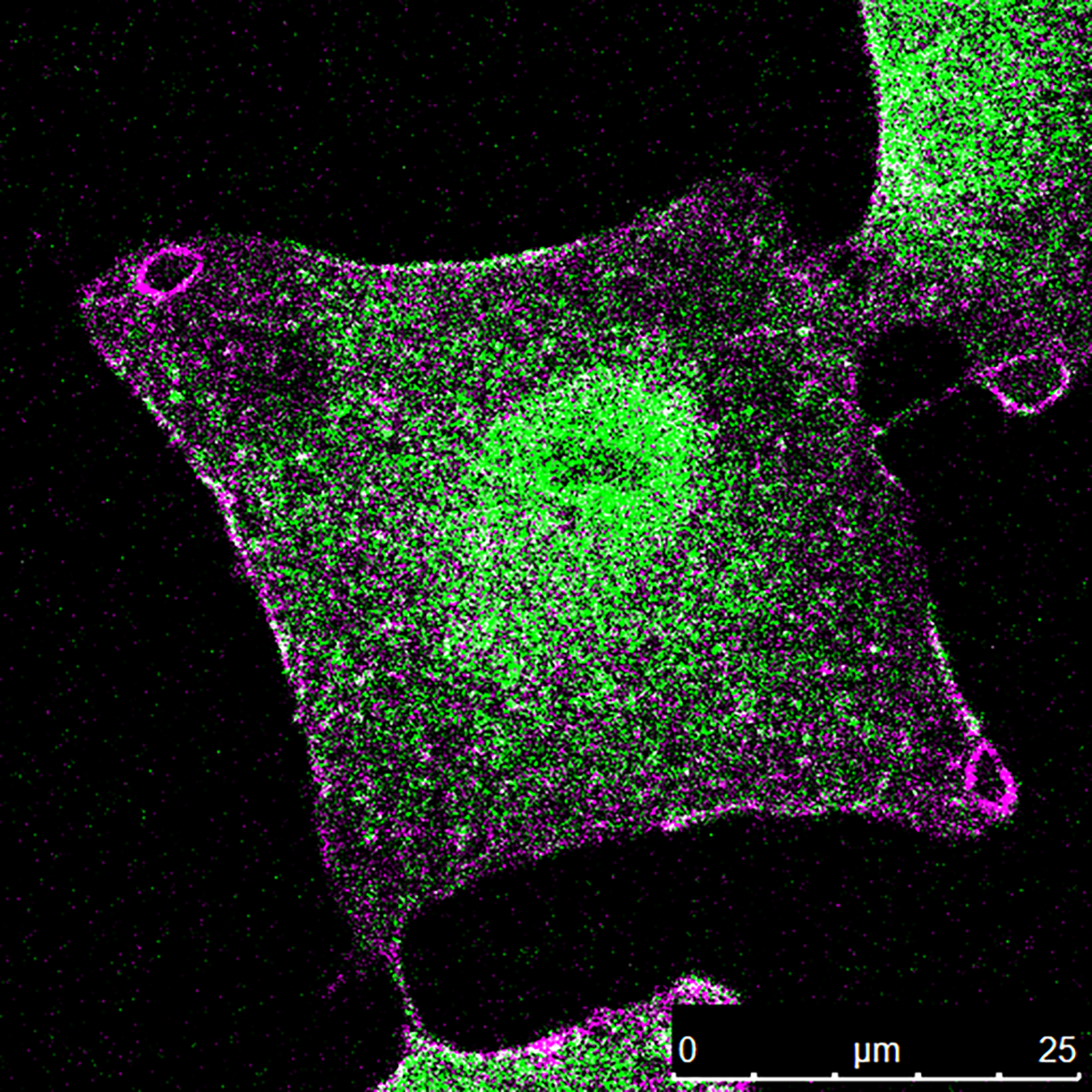

Supplement: Supplementary file 3 — Source data Fig. 2 [file 44319_2024_132_MOESM3_ESM.zip › Figure 2/2F/A549/Aho2+CTB.tif]

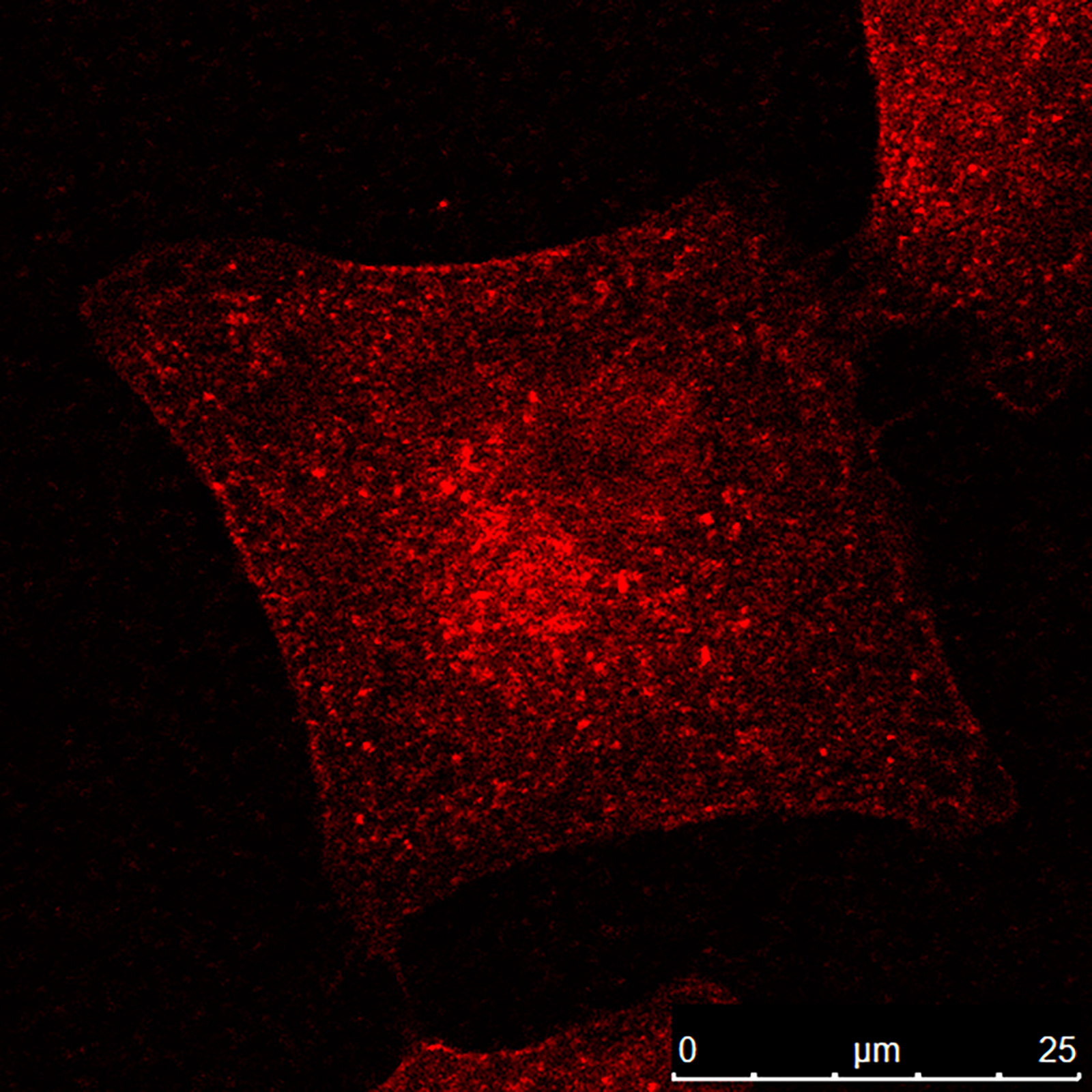

Supplement: Supplementary file 3 — Source data Fig. 2 [file 44319_2024_132_MOESM3_ESM.zip › Figure 2/2F/A549/CAV1.tif]

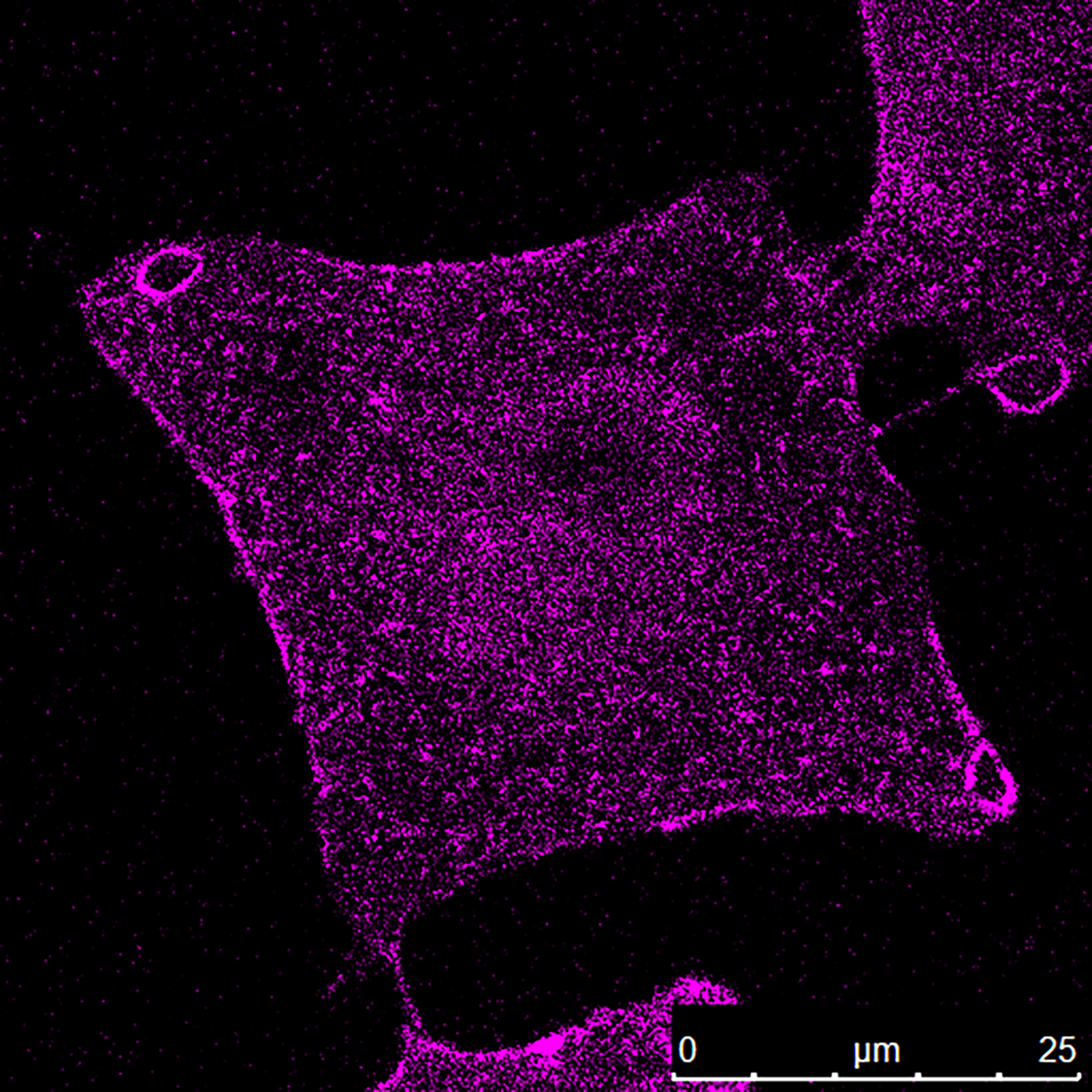

Supplement: Supplementary file 3 — Source data Fig. 2 [file 44319_2024_132_MOESM3_ESM.zip › Figure 2/2F/A549/CTB.tif]

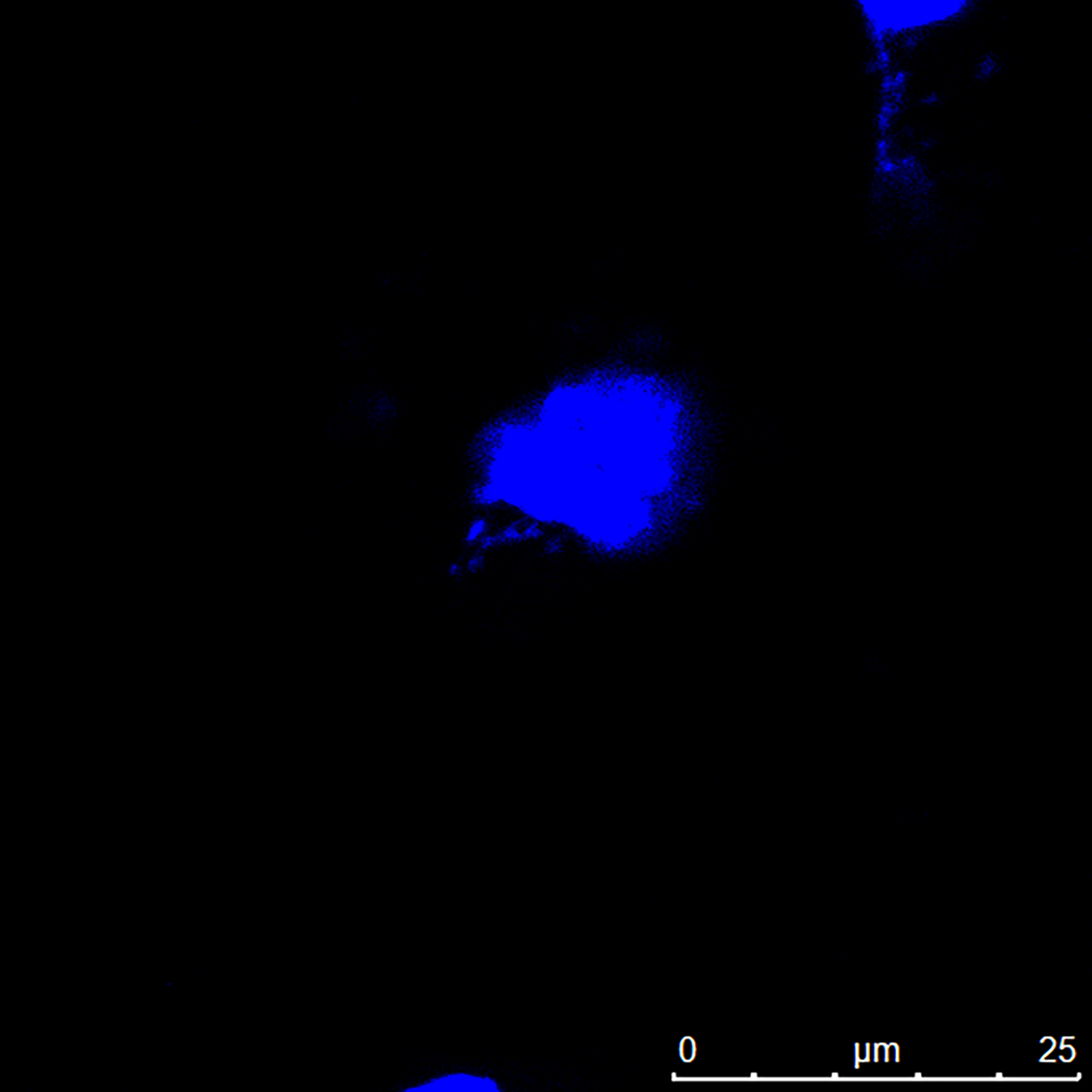

Supplement: Supplementary file 3 — Source data Fig. 2 [file 44319_2024_132_MOESM3_ESM.zip › Figure 2/2F/A549/Hoechst.tif]

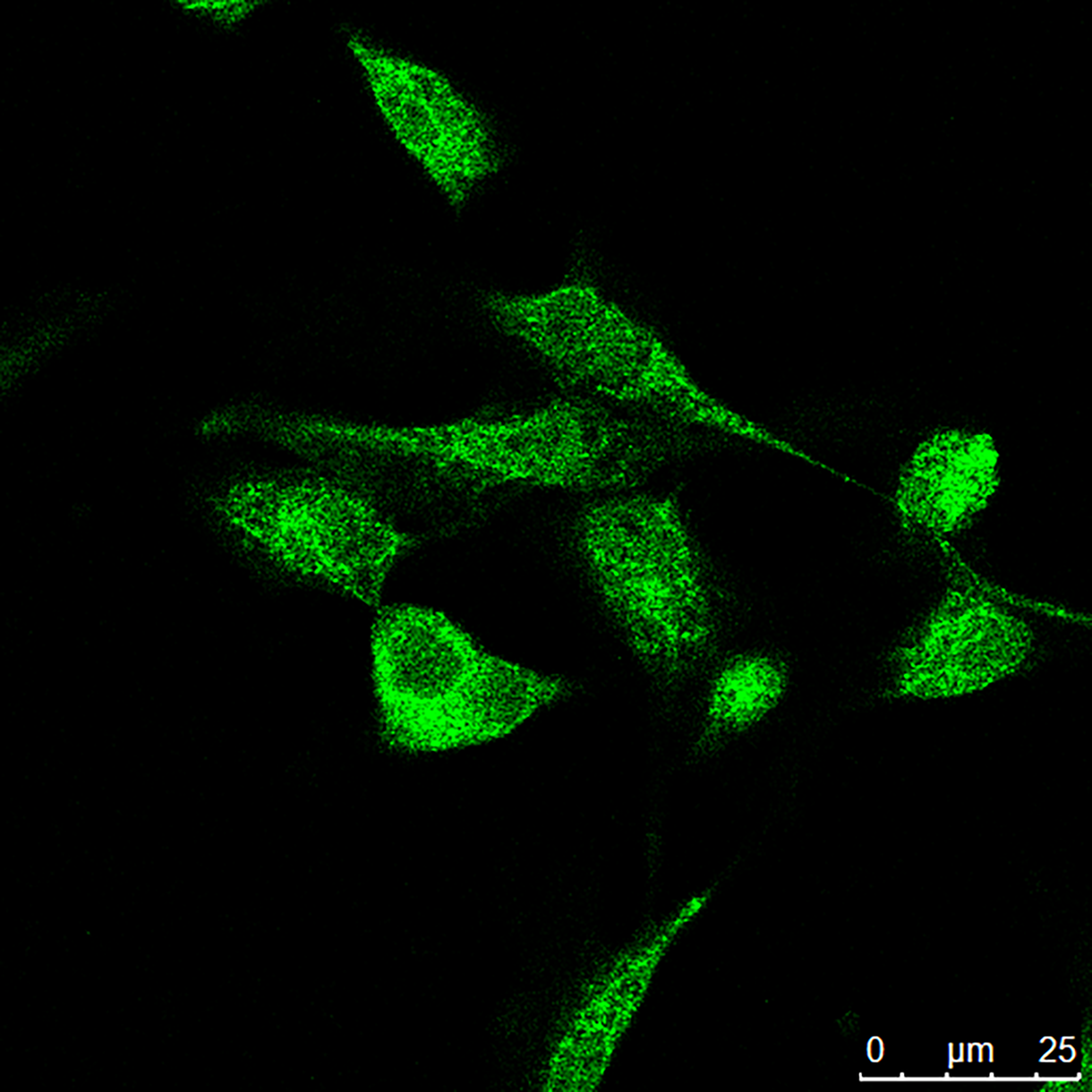

Supplement: Supplementary file 3 — Source data Fig. 2 [file 44319_2024_132_MOESM3_ESM.zip › Figure 2/2F/BEAS-2B/Ago2.tif]

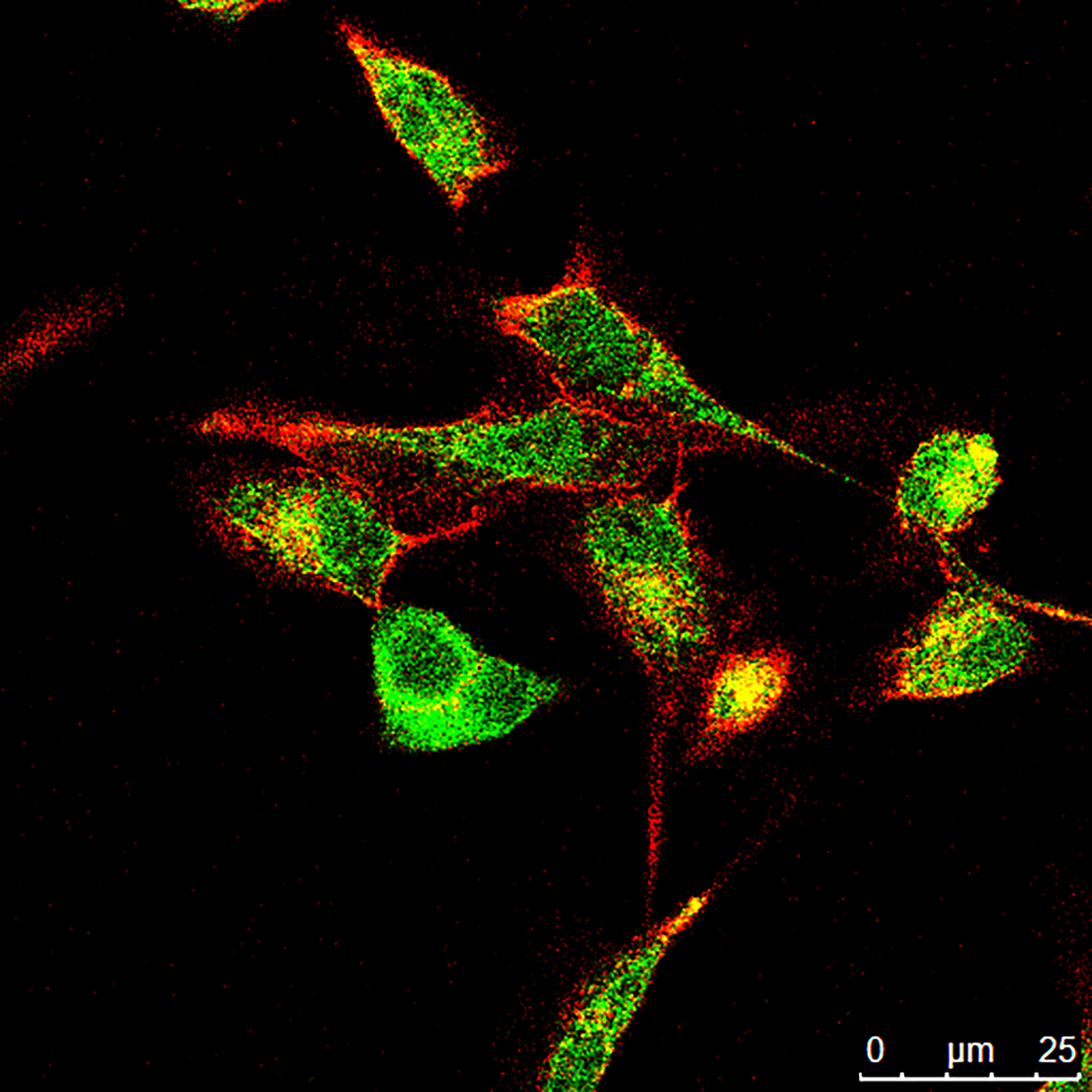

Supplement: Supplementary file 3 — Source data Fig. 2 [file 44319_2024_132_MOESM3_ESM.zip › Figure 2/2F/BEAS-2B/Ago2+CAV1.tif]

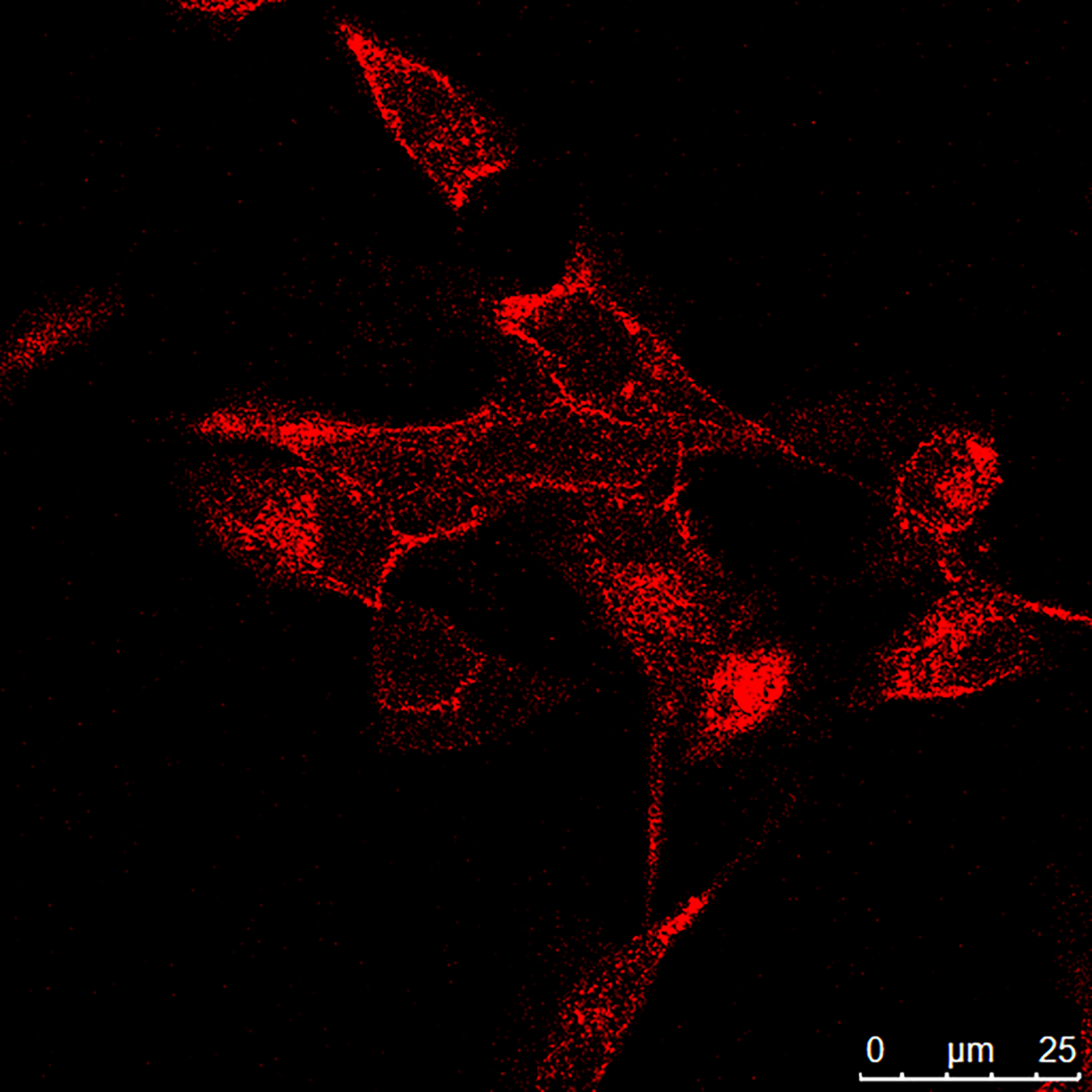

Supplement: Supplementary file 3 — Source data Fig. 2 [file 44319_2024_132_MOESM3_ESM.zip › Figure 2/2F/BEAS-2B/CAV1.tif]

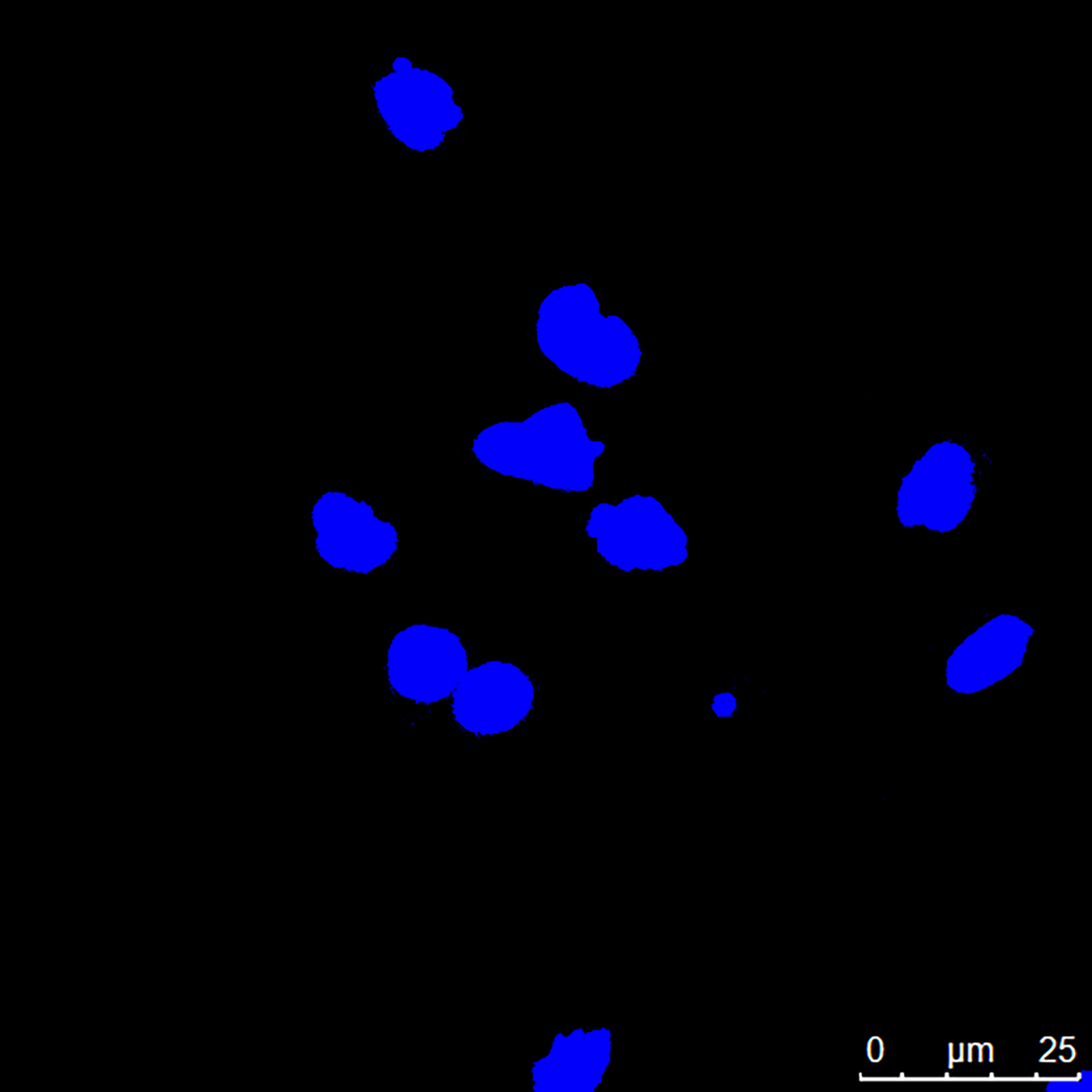

Supplement: Supplementary file 3 — Source data Fig. 2 [file 44319_2024_132_MOESM3_ESM.zip › Figure 2/2F/BEAS-2B/Hoechst.tif]

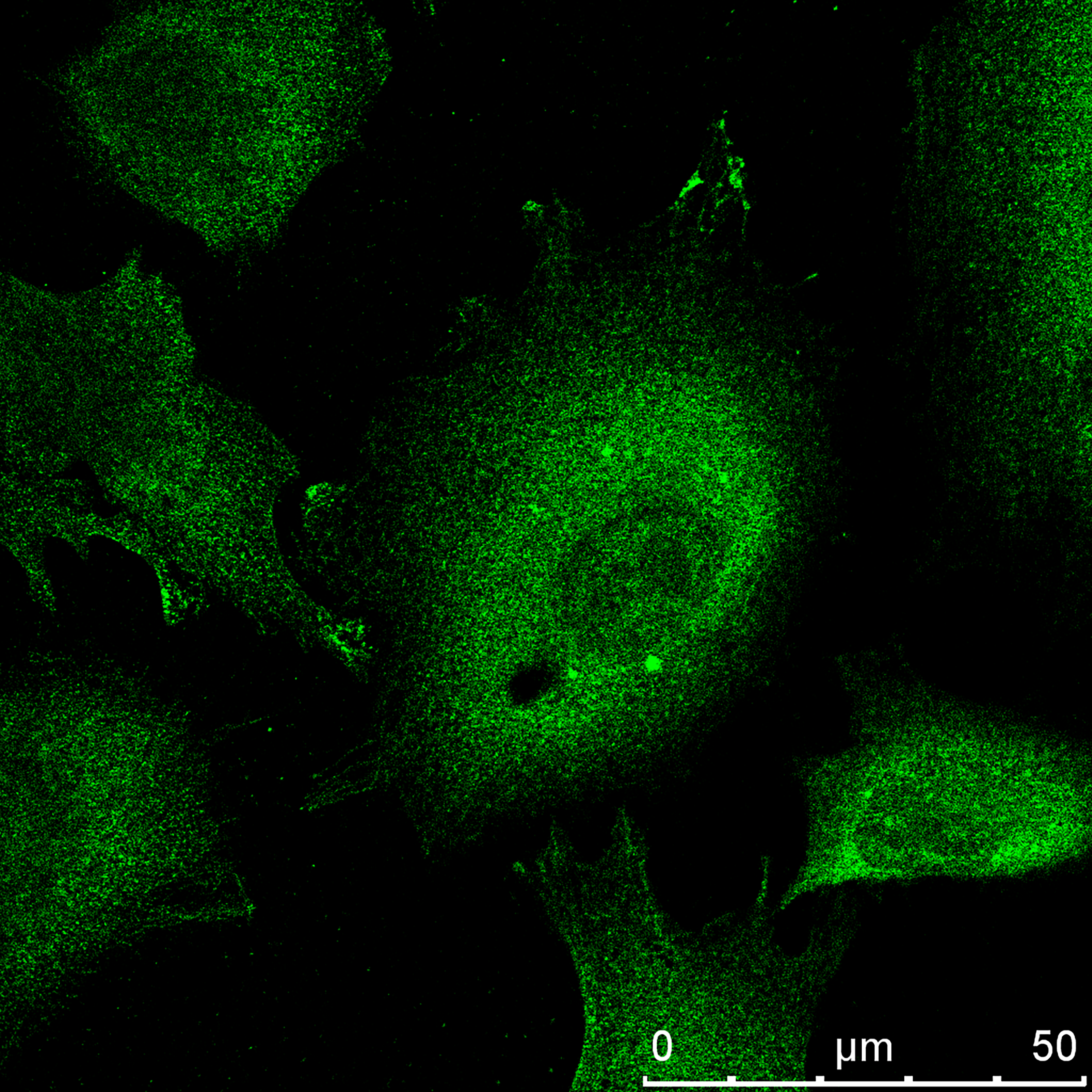

Supplement: Supplementary file 3 — Source data Fig. 2 [file 44319_2024_132_MOESM3_ESM.zip › Figure 2/2H/A549+P2/Ago2.tif]

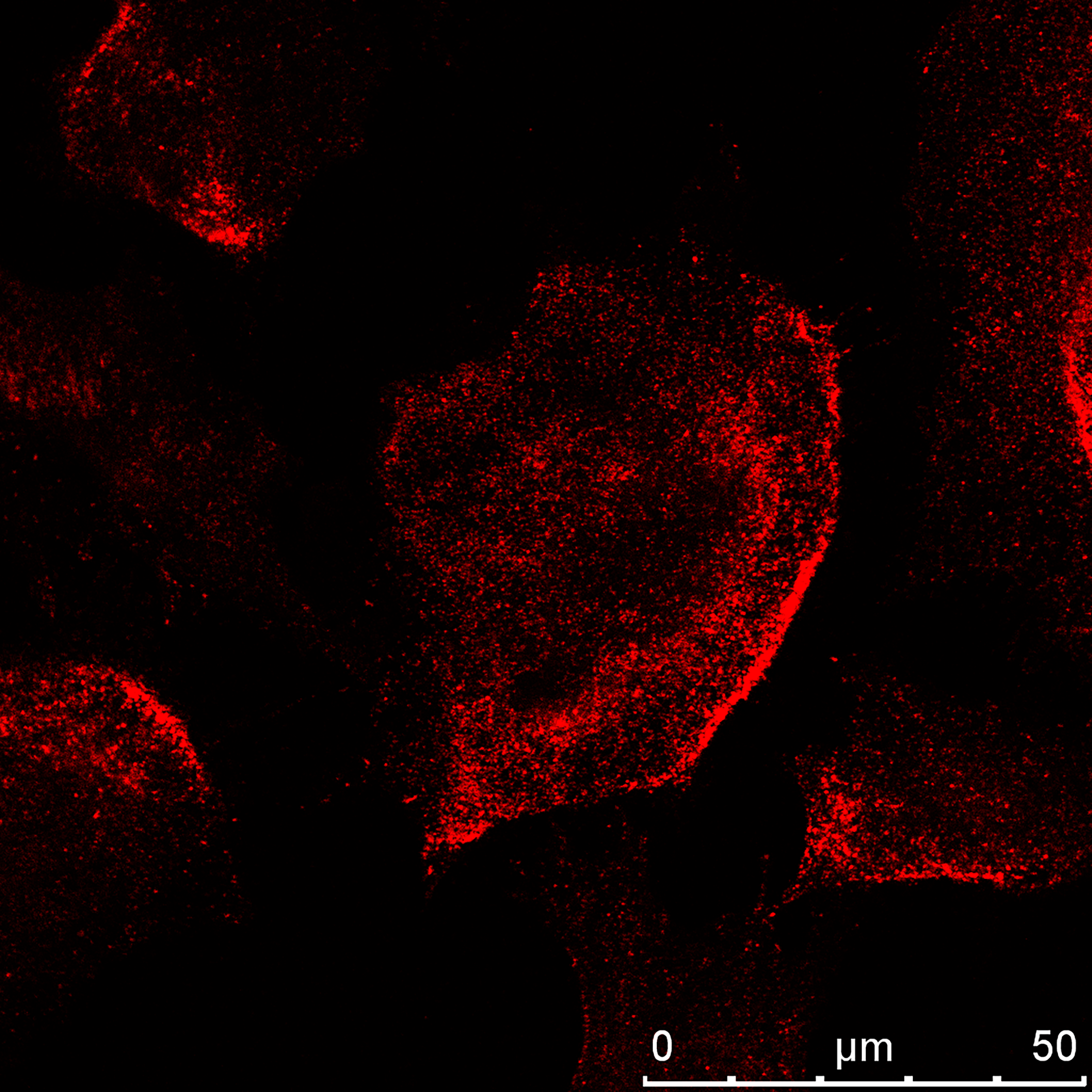

Supplement: Supplementary file 3 — Source data Fig. 2 [file 44319_2024_132_MOESM3_ESM.zip › Figure 2/2H/A549+P2/CAV1.tif]

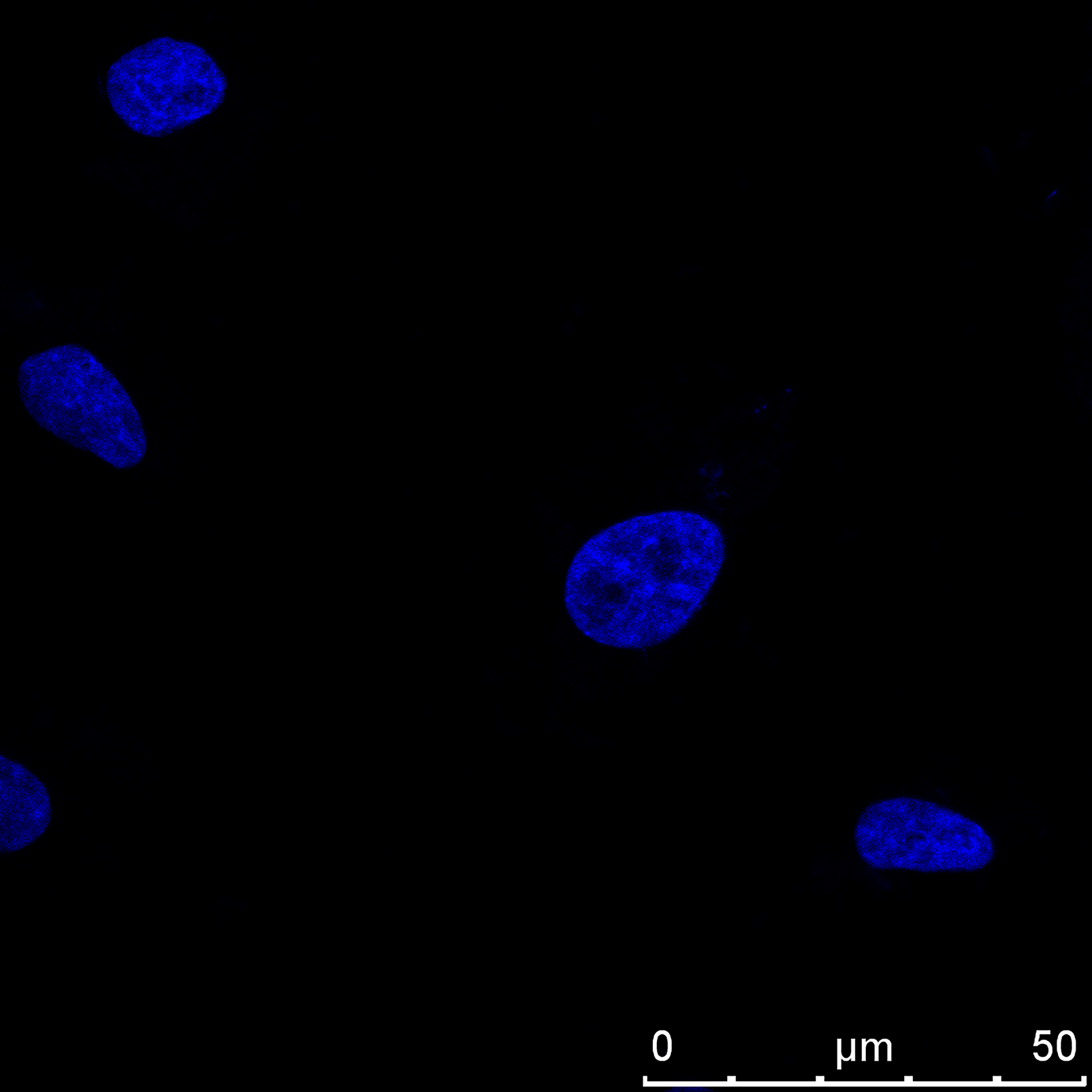

Supplement: Supplementary file 3 — Source data Fig. 2 [file 44319_2024_132_MOESM3_ESM.zip › Figure 2/2H/A549+P2/Hoechst.tif]

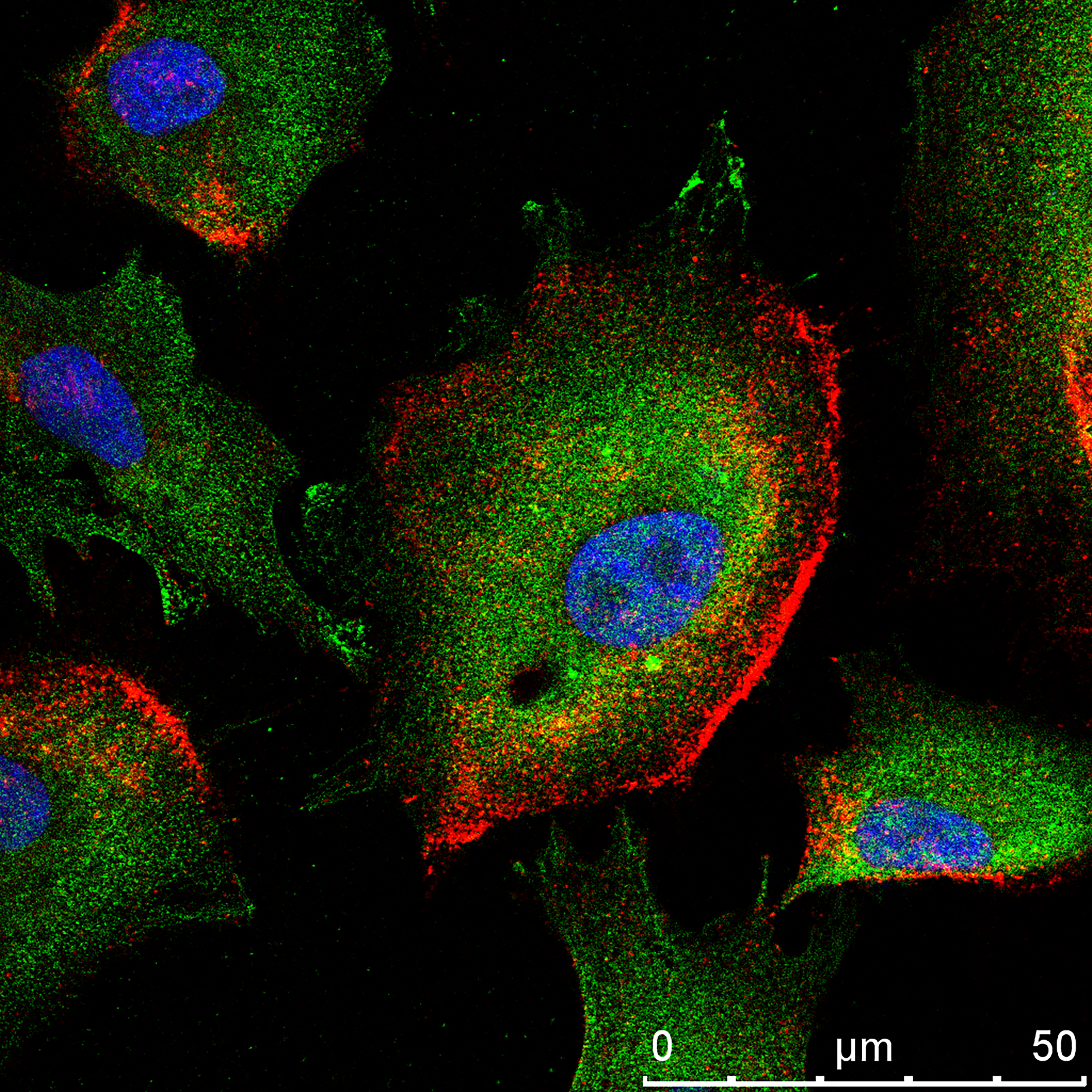

Supplement: Supplementary file 3 — Source data Fig. 2 [file 44319_2024_132_MOESM3_ESM.zip › Figure 2/2H/A549+P2/Merged.tif]

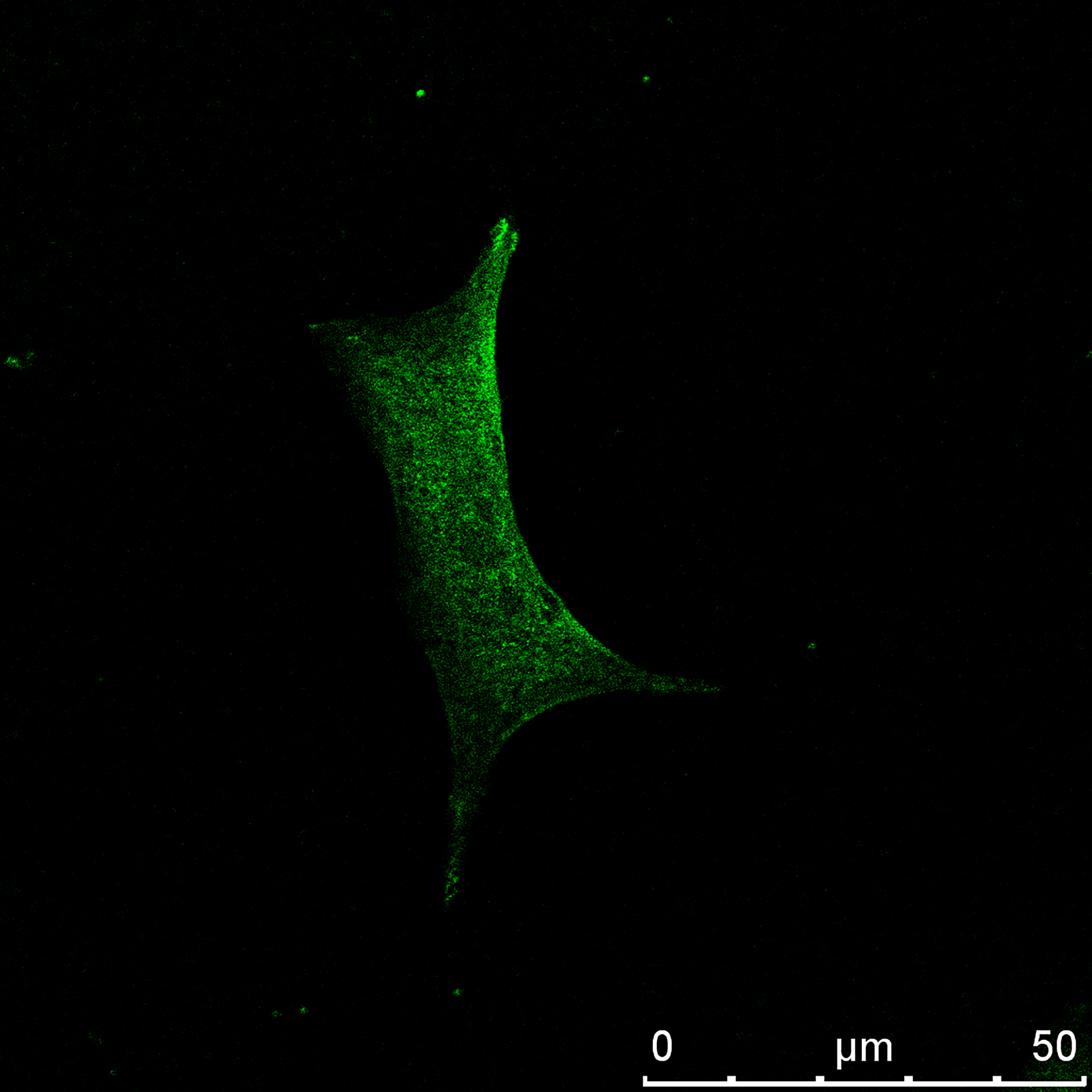

Supplement: Supplementary file 3 — Source data Fig. 2 [file 44319_2024_132_MOESM3_ESM.zip › Figure 2/2H/A549+P2S/Ago2.tif]

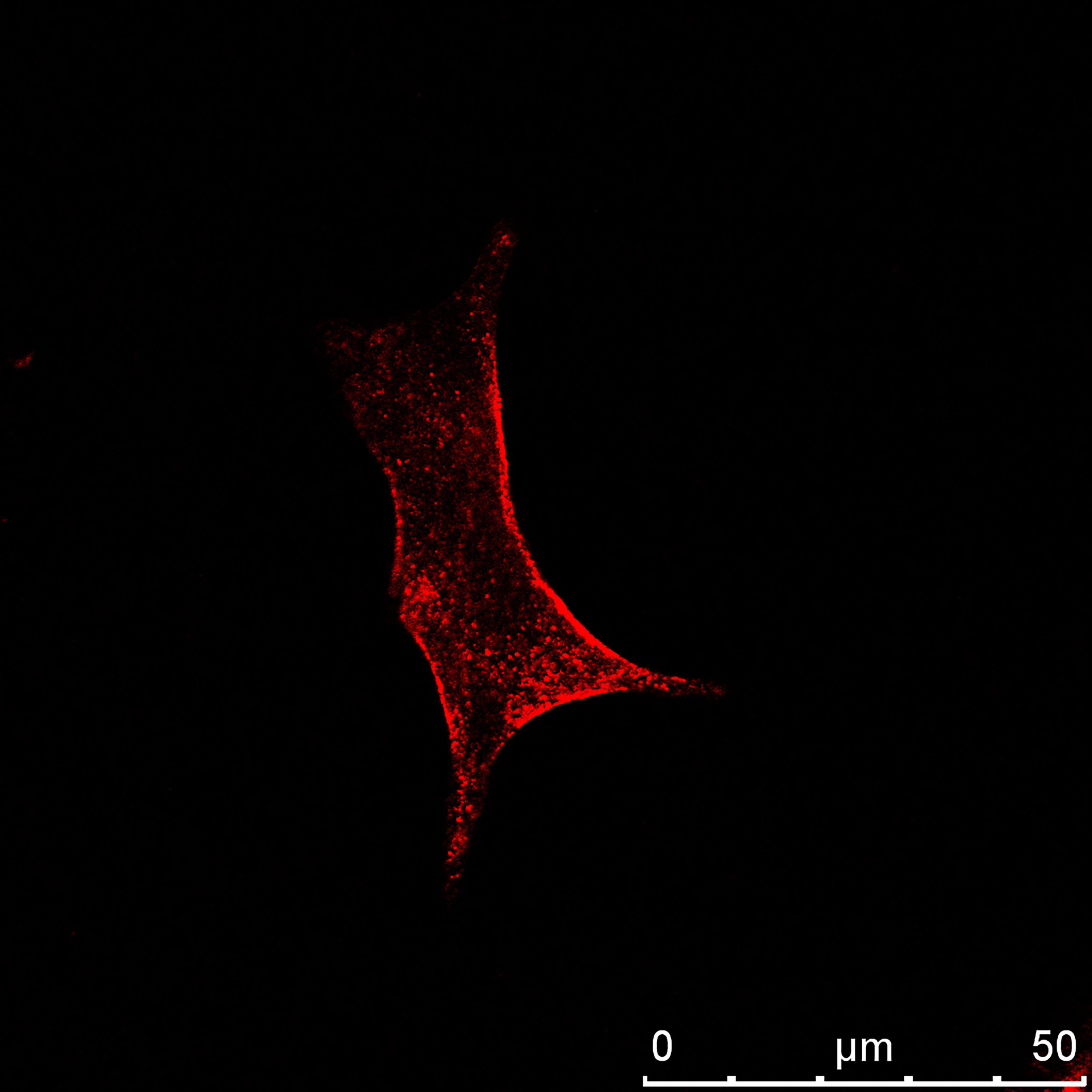

Supplement: Supplementary file 3 — Source data Fig. 2 [file 44319_2024_132_MOESM3_ESM.zip › Figure 2/2H/A549+P2S/CAV1.tif]

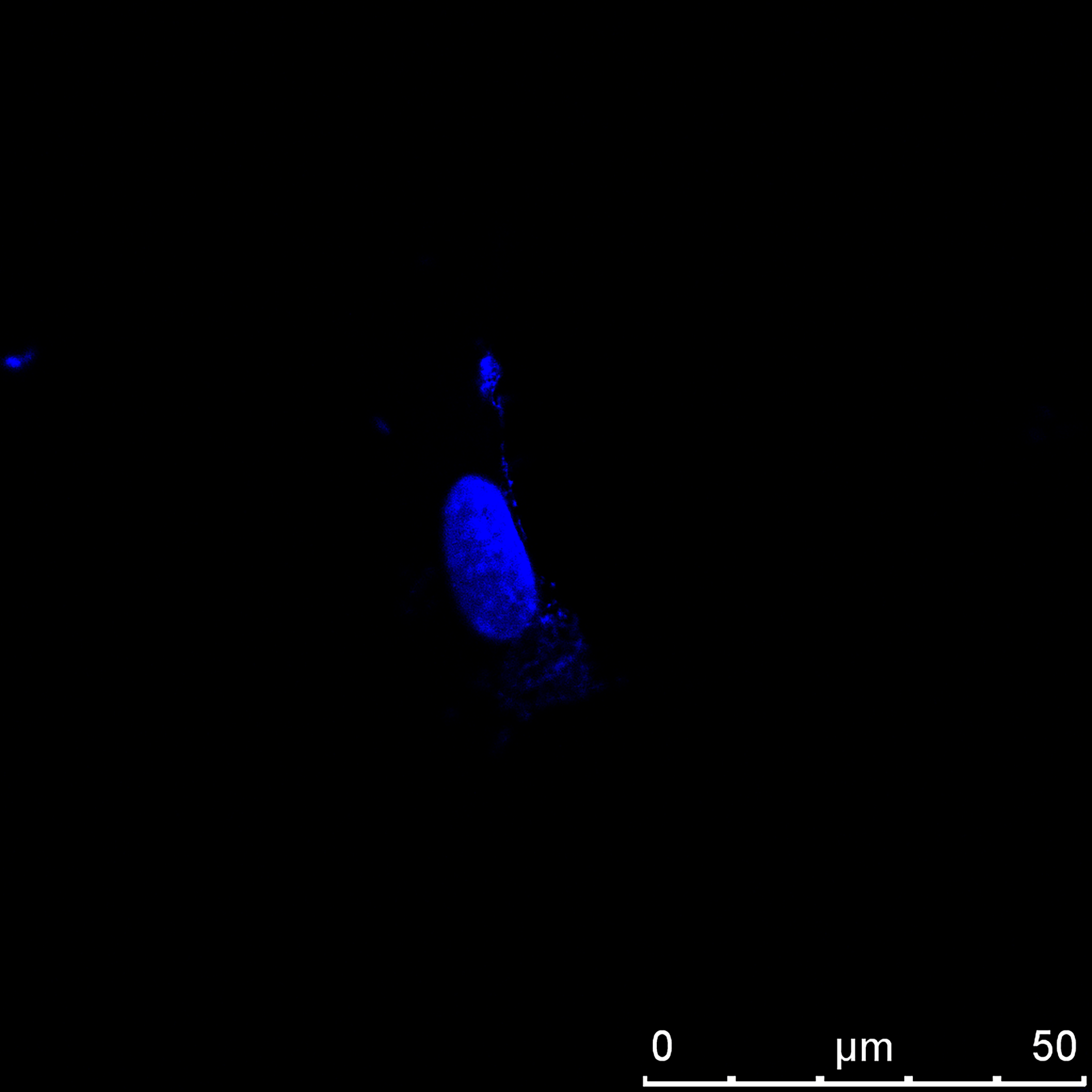

Supplement: Supplementary file 3 — Source data Fig. 2 [file 44319_2024_132_MOESM3_ESM.zip › Figure 2/2H/A549+P2S/Hoechst.tif]

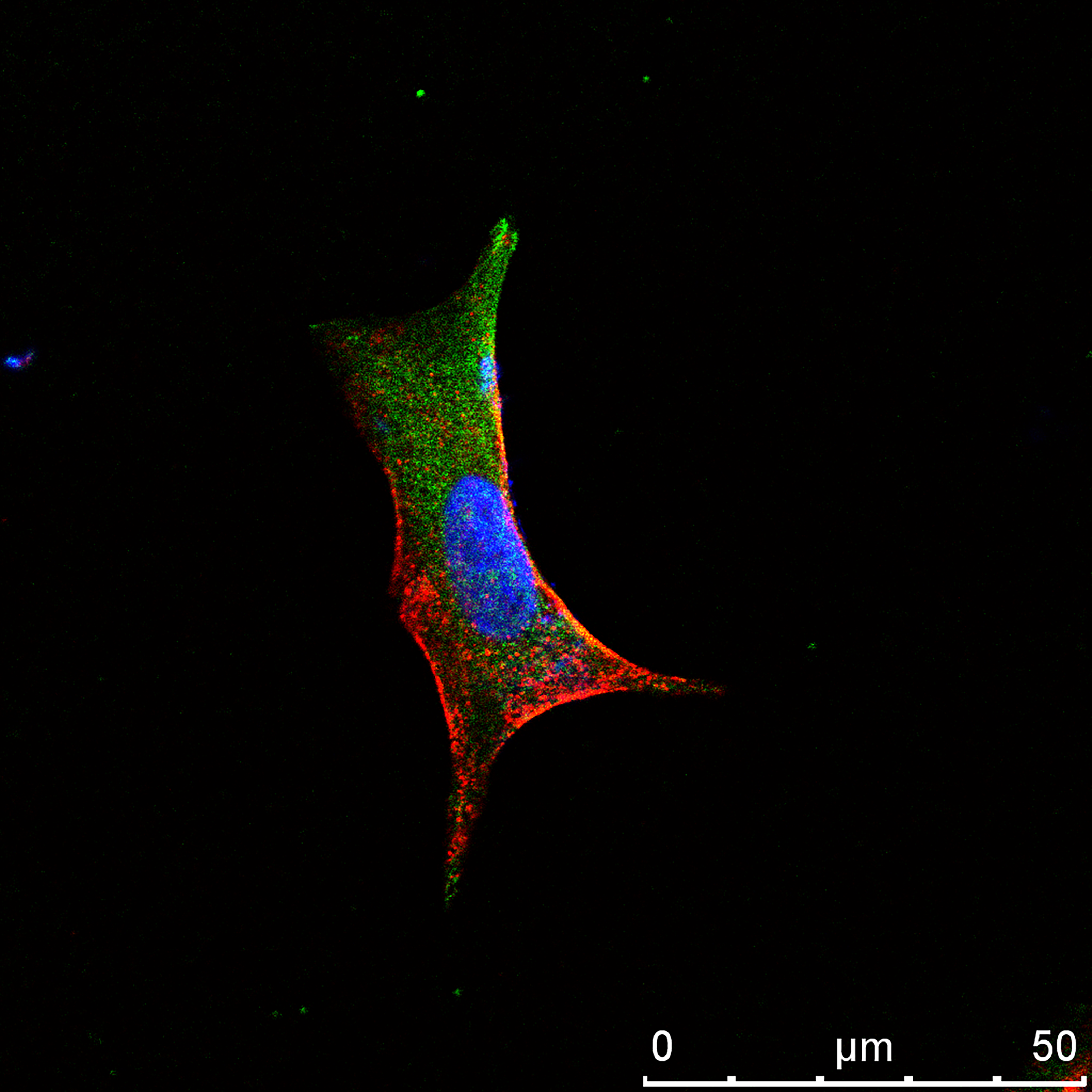

Supplement: Supplementary file 3 — Source data Fig. 2 [file 44319_2024_132_MOESM3_ESM.zip › Figure 2/2H/A549+P2S/Merged.tif]

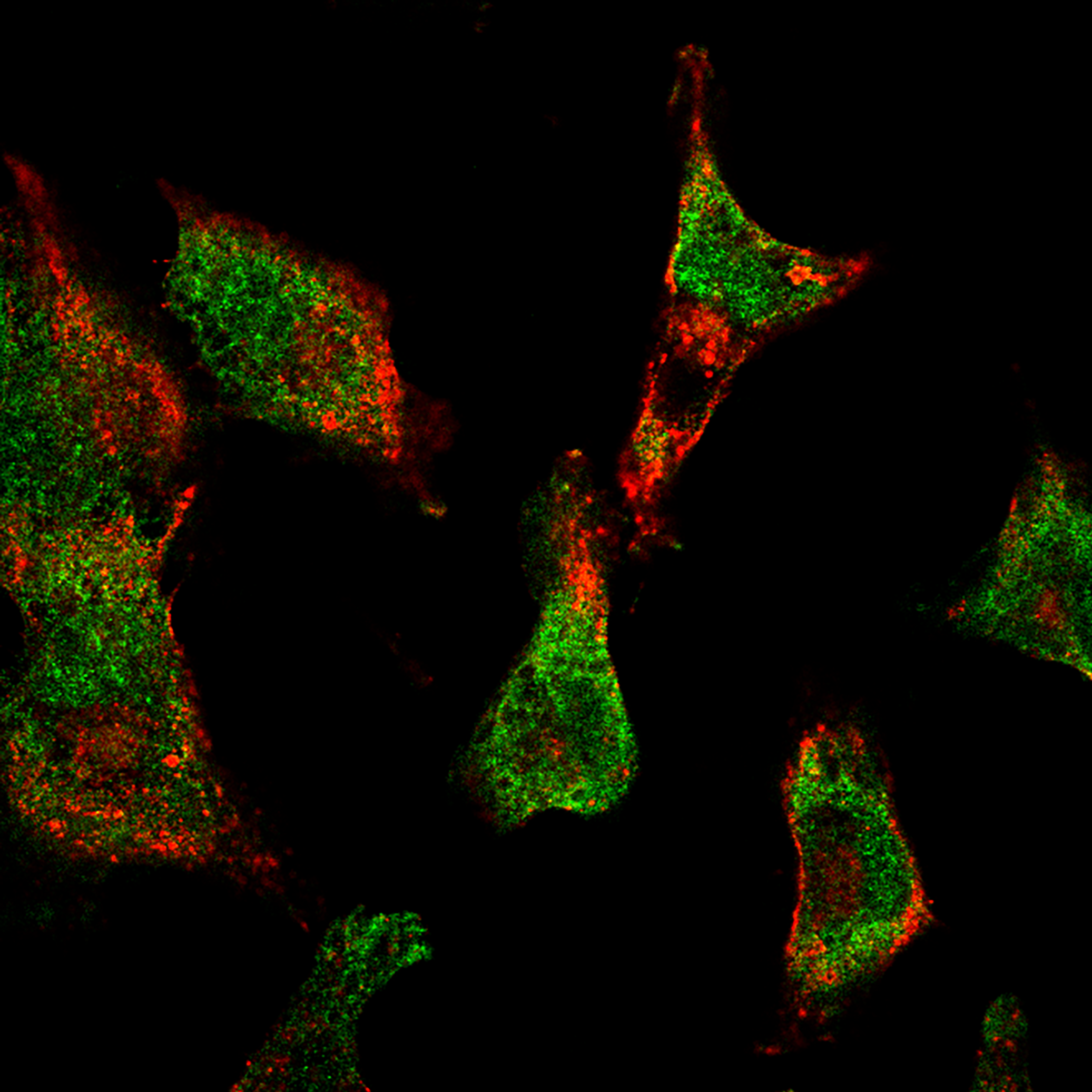

Supplement: Supplementary file 3 — Source data Fig. 2 [file 44319_2024_132_MOESM3_ESM.zip › Figure 2/2H/HCC1806+P2/HCC-P2.lif_Series017_SubVolume002.tif]

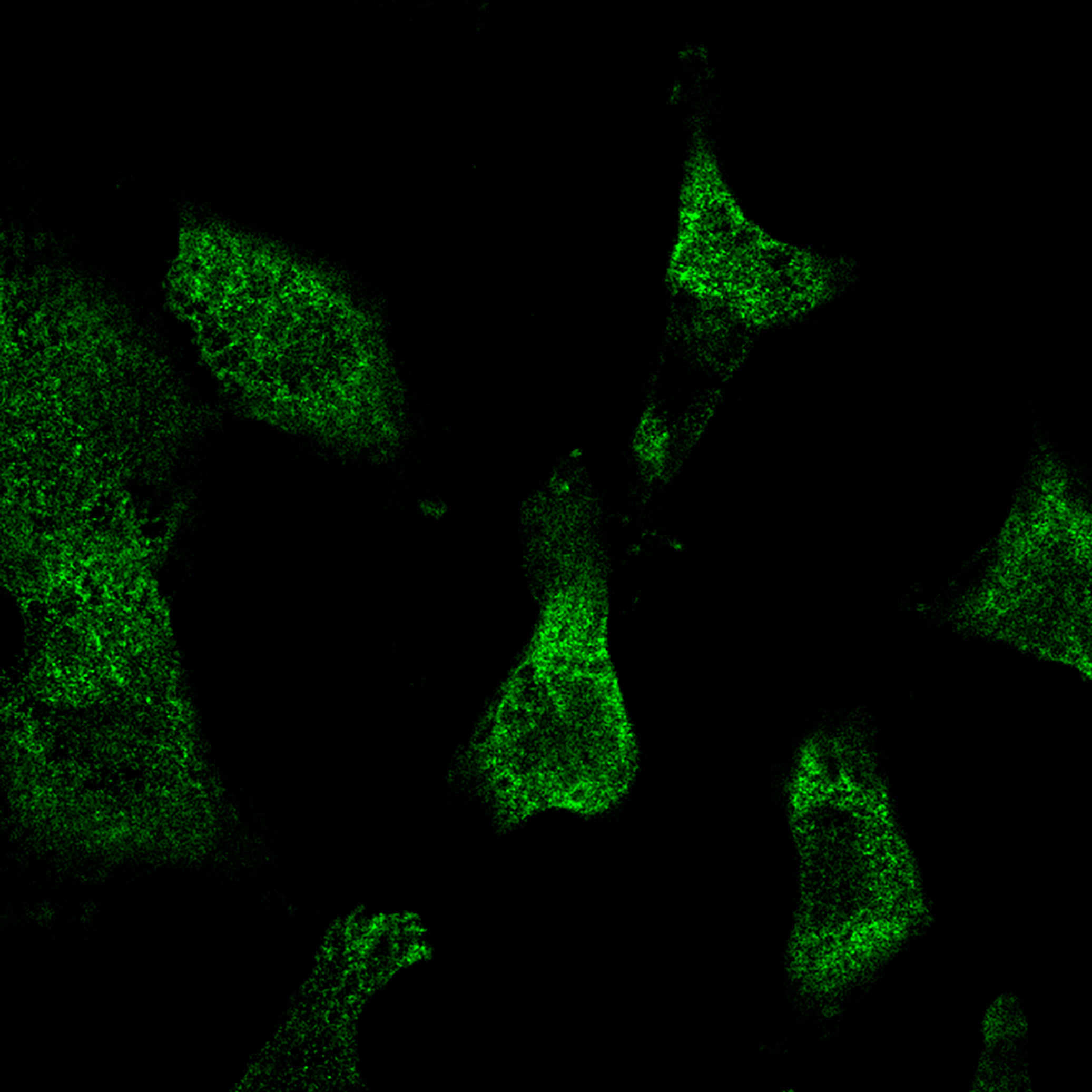

Supplement: Supplementary file 3 — Source data Fig. 2 [file 44319_2024_132_MOESM3_ESM.zip › Figure 2/2H/HCC1806+P2/HCC-P2.lif_Series017_SubVolume002_ch00.tif]

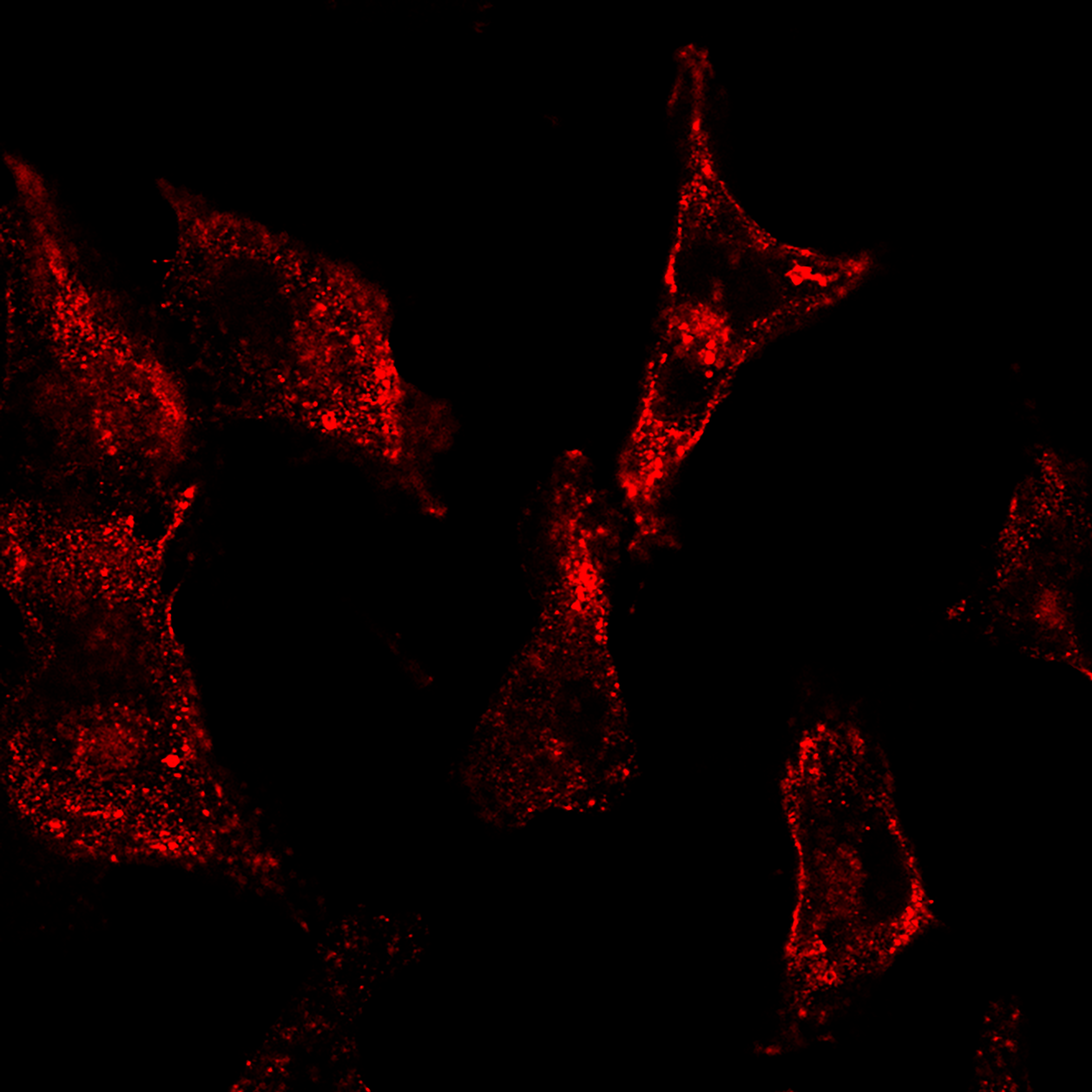

Supplement: Supplementary file 3 — Source data Fig. 2 [file 44319_2024_132_MOESM3_ESM.zip › Figure 2/2H/HCC1806+P2/HCC-P2.lif_Series017_SubVolume002_ch01.tif]

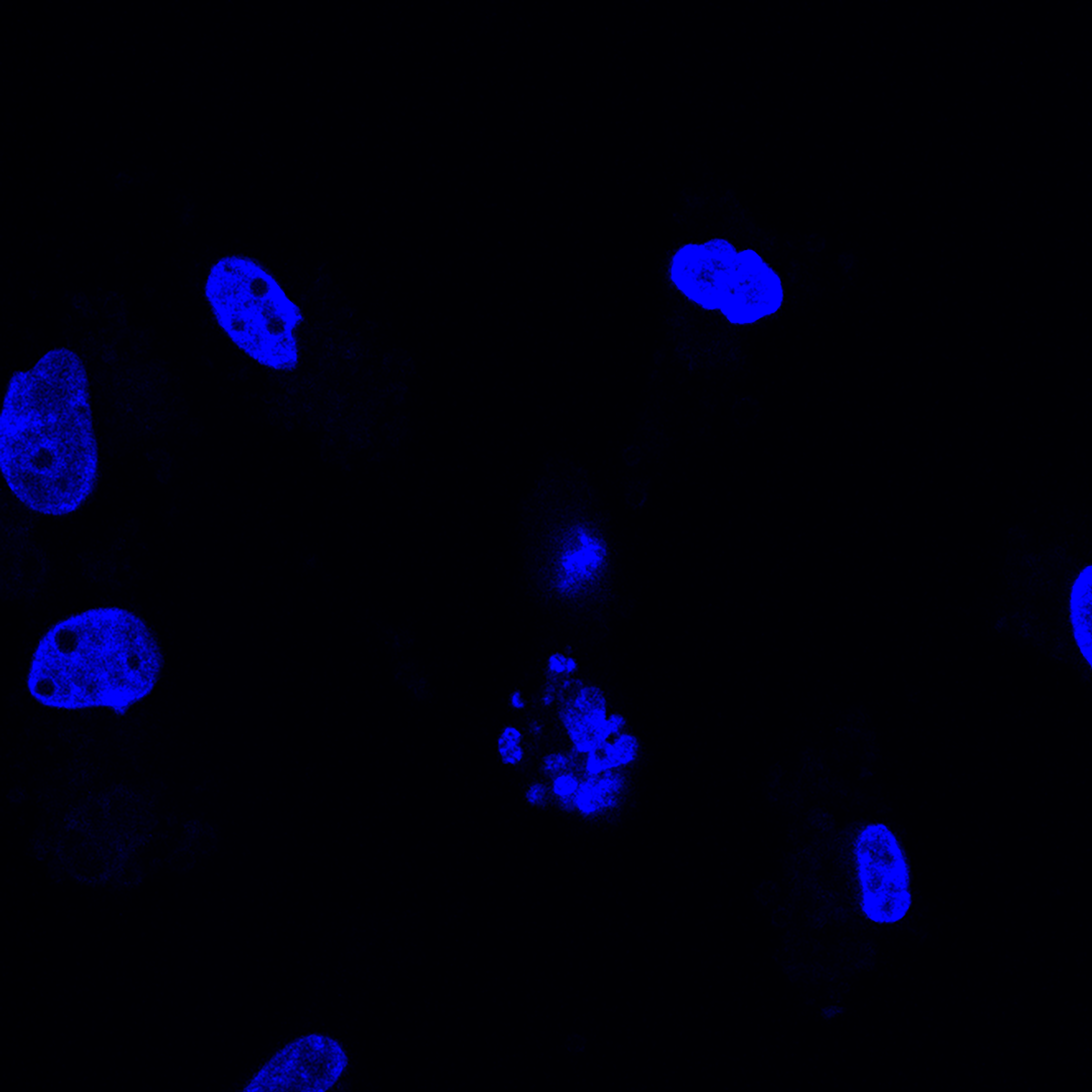

Supplement: Supplementary file 3 — Source data Fig. 2 [file 44319_2024_132_MOESM3_ESM.zip › Figure 2/2H/HCC1806+P2/HCC-P2.lif_Series017_SubVolume002_ch02.tif]
