# Supplementary figures and images for: Ago2/CAV1 interaction potentiates metastasis via controlling Ago2 localization and miRNA action (part 2 of 3)
Source: EMBO Rep. 2024 Apr 22;25(5):20. doi: 10.1038/s44319-024-00132-7 (PMC11094075; doi:10.1038/s44319-024-00132-7)

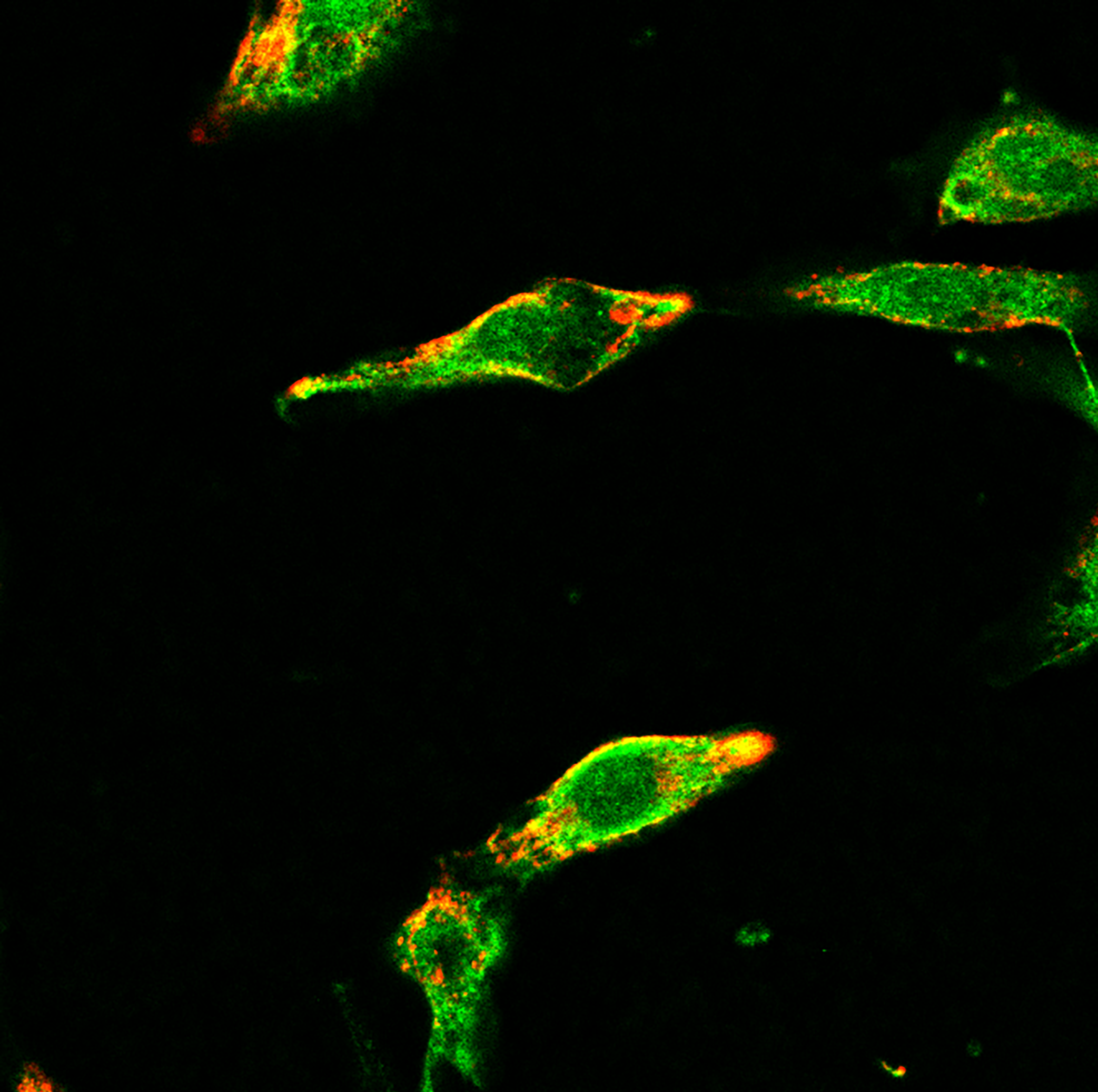

Supplement: Supplementary file 3 — Source data Fig. 2 [file 44319_2024_132_MOESM3_ESM.zip › Figure 2/2H/HCC1806+P2S/HCC18006-P2S.lif_Series002_SubVolume001.tif]

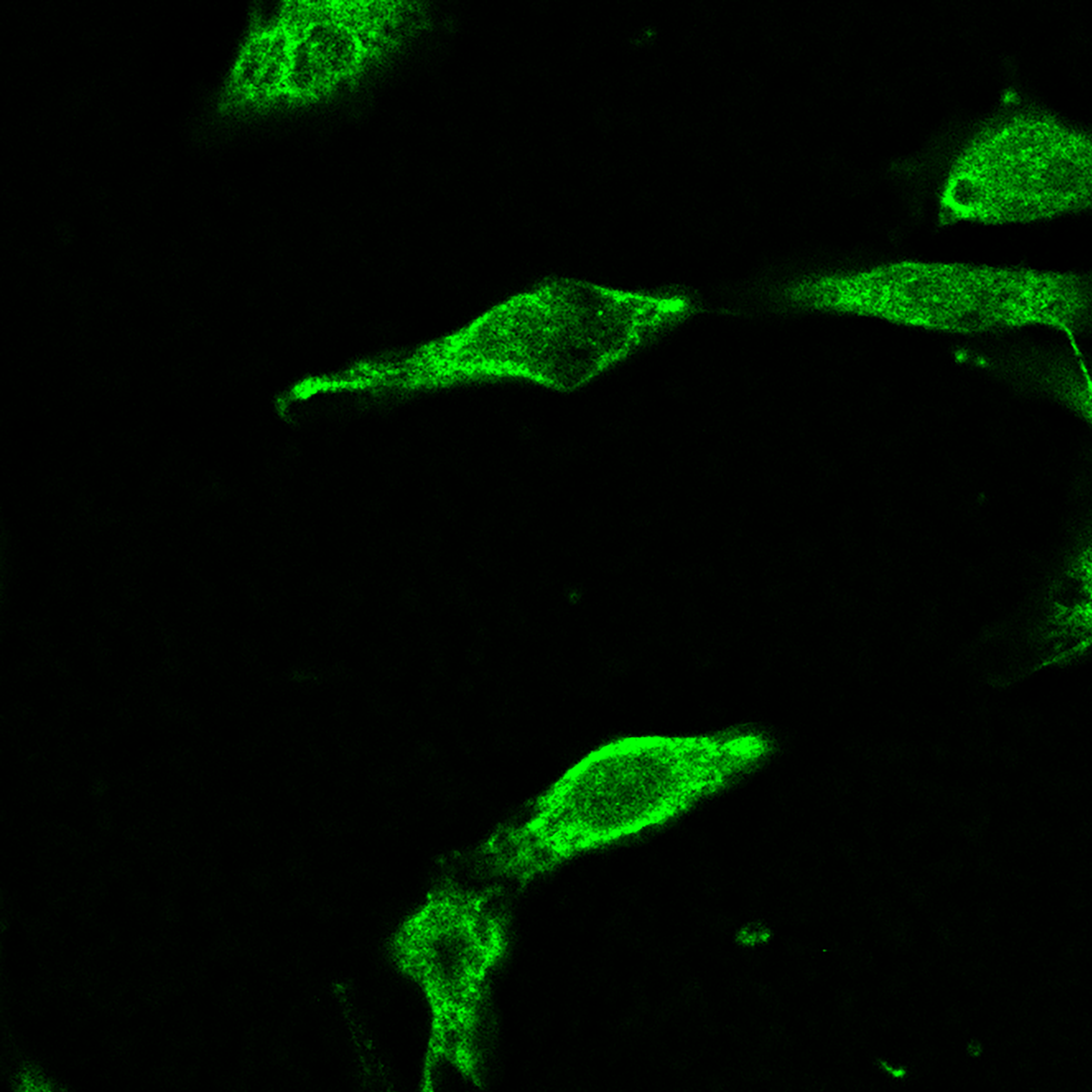

Supplement: Supplementary file 3 — Source data Fig. 2 [file 44319_2024_132_MOESM3_ESM.zip › Figure 2/2H/HCC1806+P2S/HCC18006-P2S.lif_Series002_SubVolume001_ch00.tif]

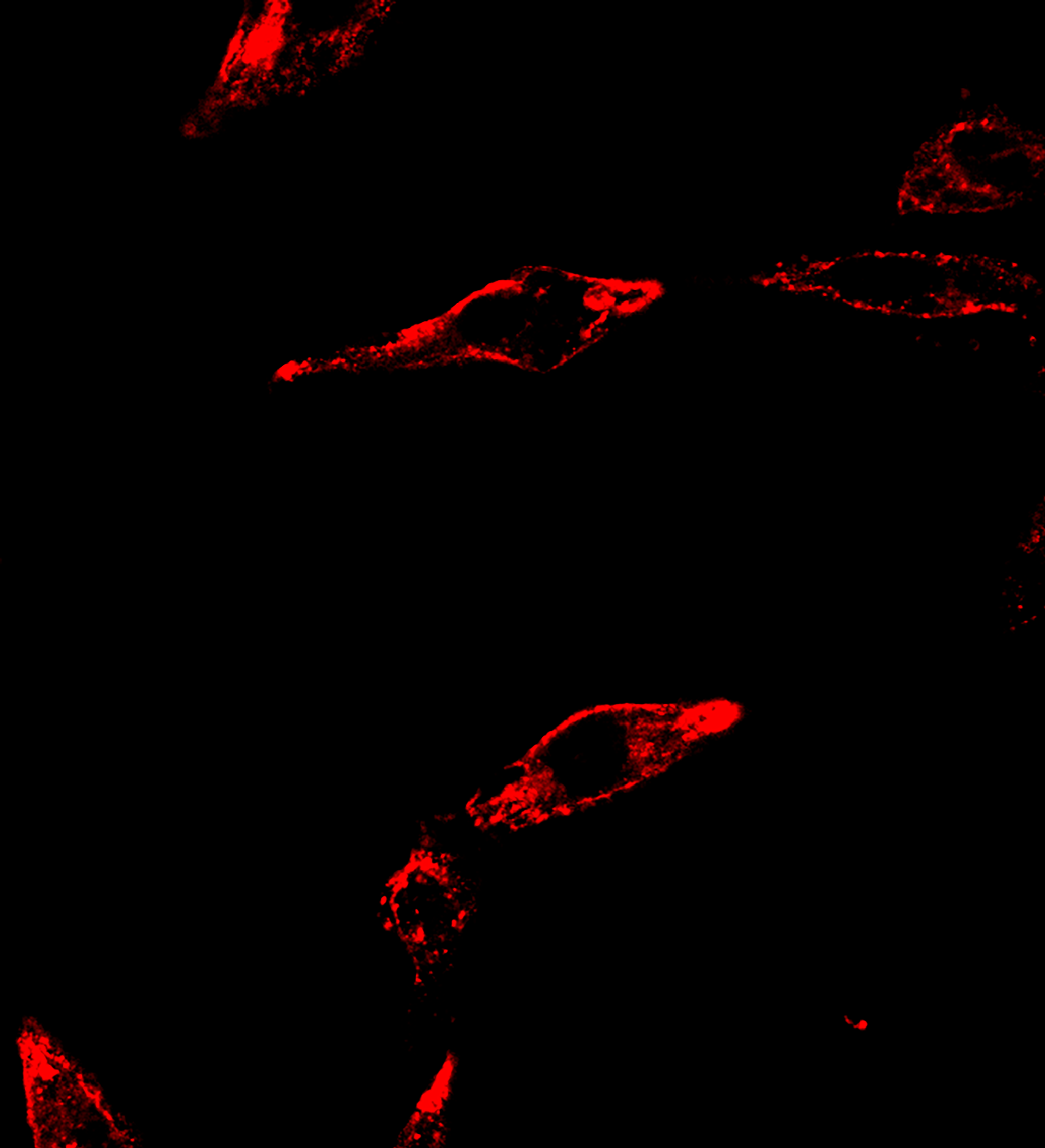

Supplement: Supplementary file 3 — Source data Fig. 2 [file 44319_2024_132_MOESM3_ESM.zip › Figure 2/2H/HCC1806+P2S/HCC18006-P2S.lif_Series002_SubVolume001_ch01.tif]

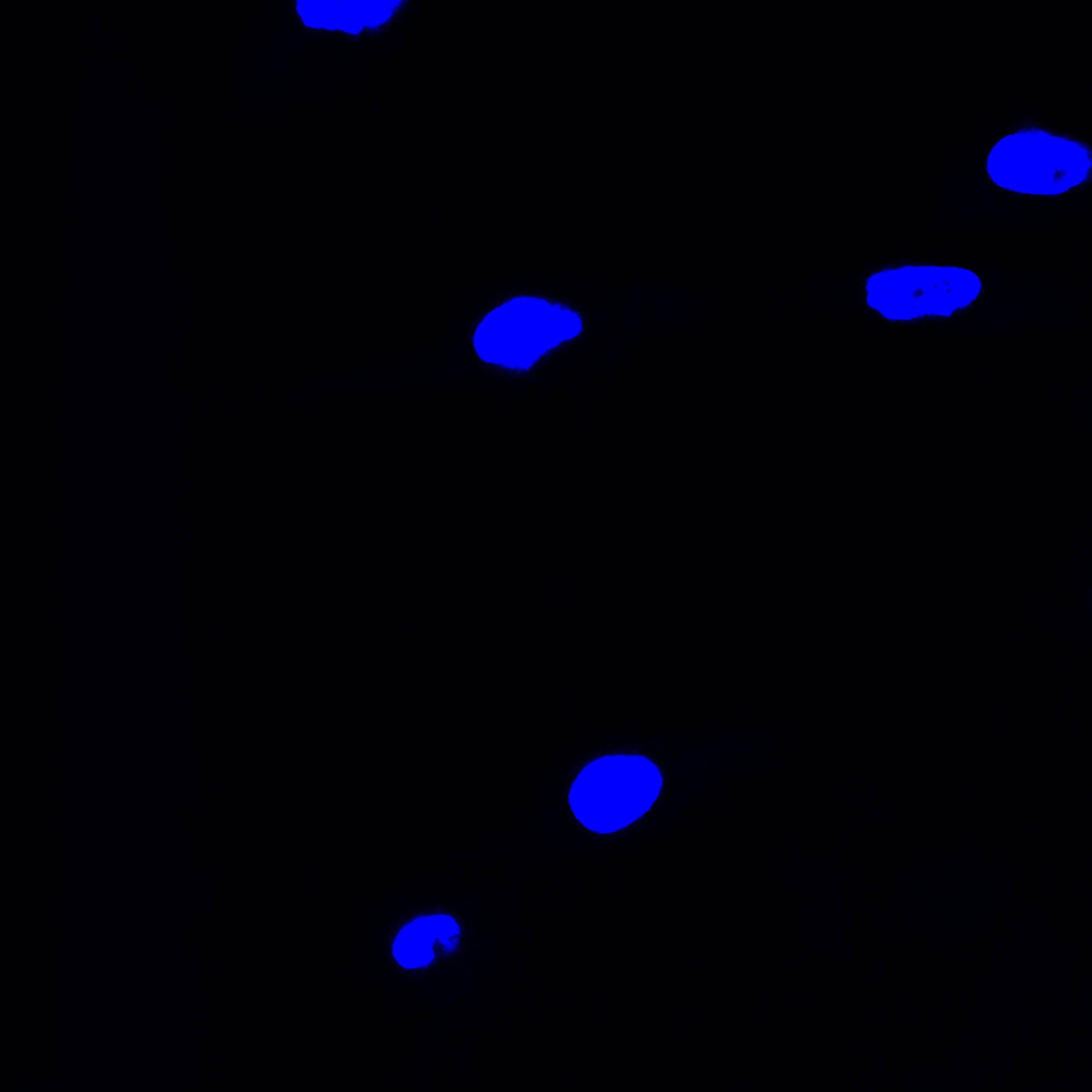

Supplement: Supplementary file 3 — Source data Fig. 2 [file 44319_2024_132_MOESM3_ESM.zip › Figure 2/2H/HCC1806+P2S/HCC18006-P2S.lif_Series002_SubVolume001_ch02.tif]

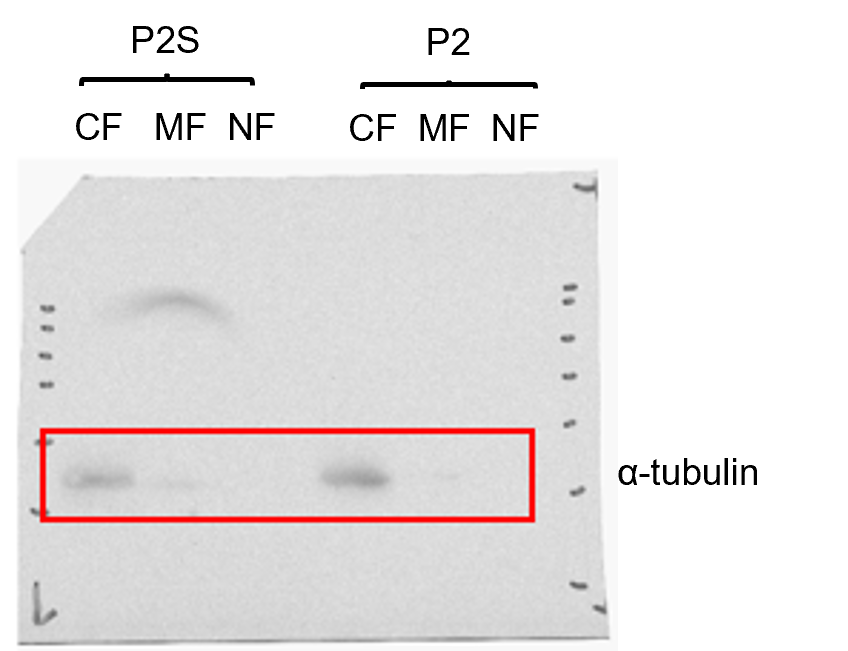

Supplement: Supplementary file 4 — Source data Fig. 3 [file 44319_2024_132_MOESM4_ESM.zip › Figure 3/3F/western alpha tubulin.tif]

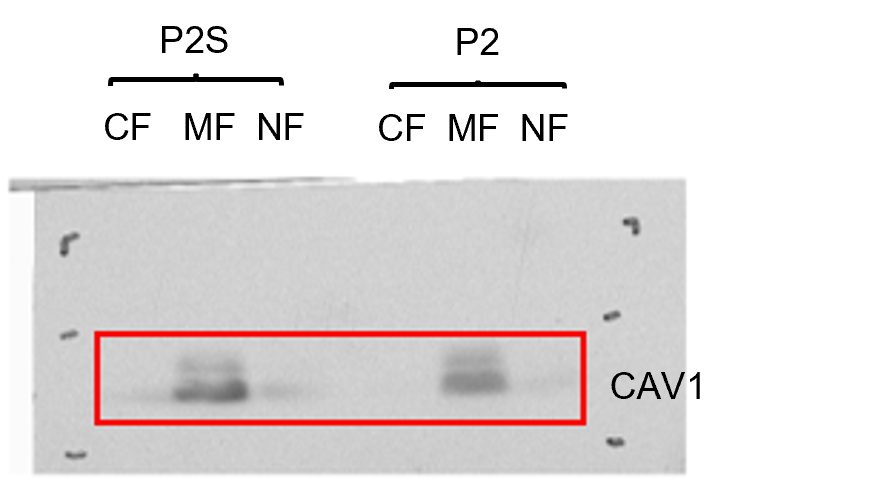

Supplement: Supplementary file 4 — Source data Fig. 3 [file 44319_2024_132_MOESM4_ESM.zip › Figure 3/3F/western CAV1.tif]

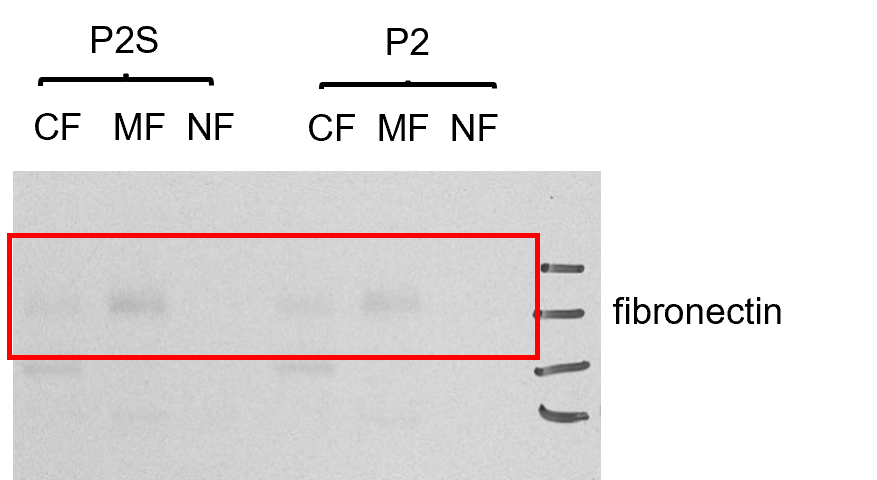

Supplement: Supplementary file 4 — Source data Fig. 3 [file 44319_2024_132_MOESM4_ESM.zip › Figure 3/3F/western Fibronectin.tif]

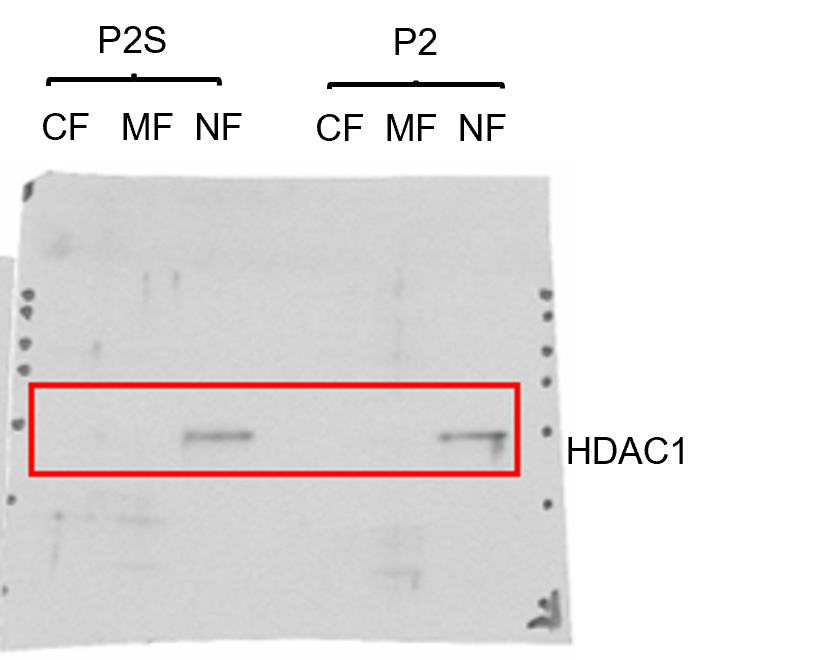

Supplement: Supplementary file 4 — Source data Fig. 3 [file 44319_2024_132_MOESM4_ESM.zip › Figure 3/3F/western HDAC1.tif]

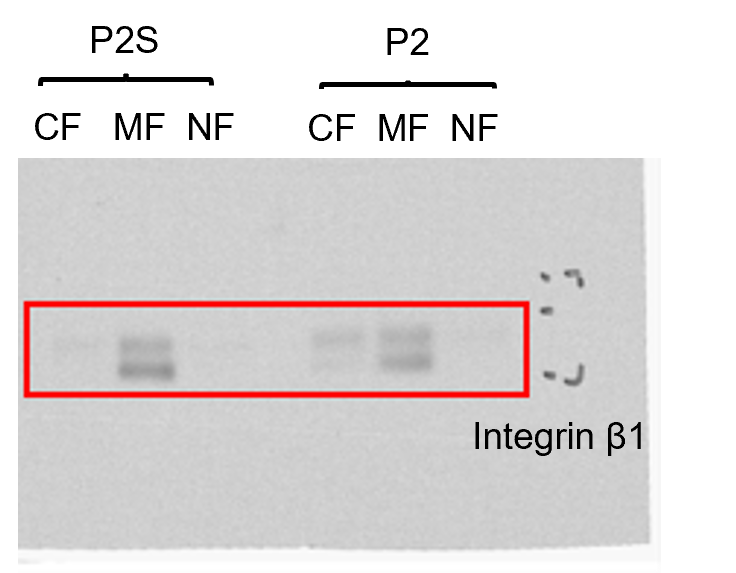

Supplement: Supplementary file 4 — Source data Fig. 3 [file 44319_2024_132_MOESM4_ESM.zip › Figure 3/3F/western Integrin beta 1.tif]

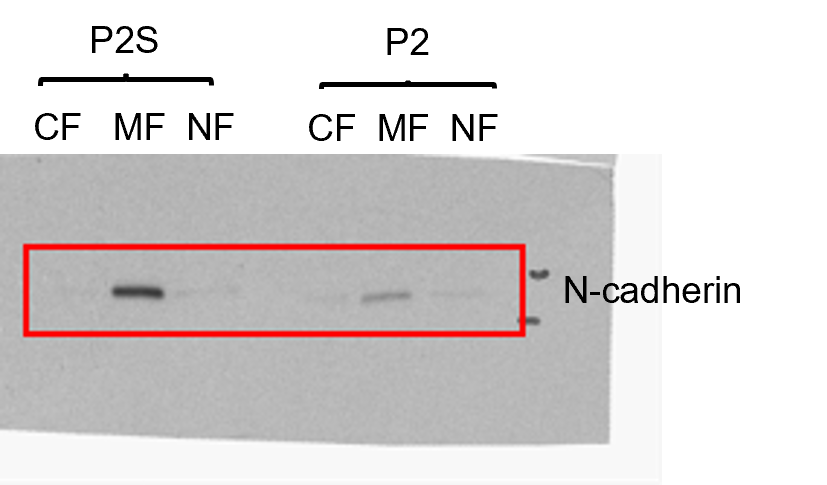

Supplement: Supplementary file 4 — Source data Fig. 3 [file 44319_2024_132_MOESM4_ESM.zip › Figure 3/3F/western N-cadherin.tif]

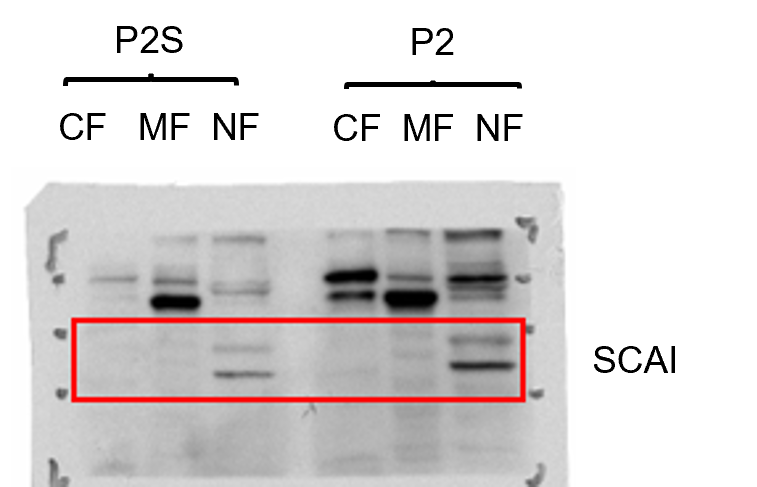

Supplement: Supplementary file 4 — Source data Fig. 3 [file 44319_2024_132_MOESM4_ESM.zip › Figure 3/3F/western SCAI.tif]

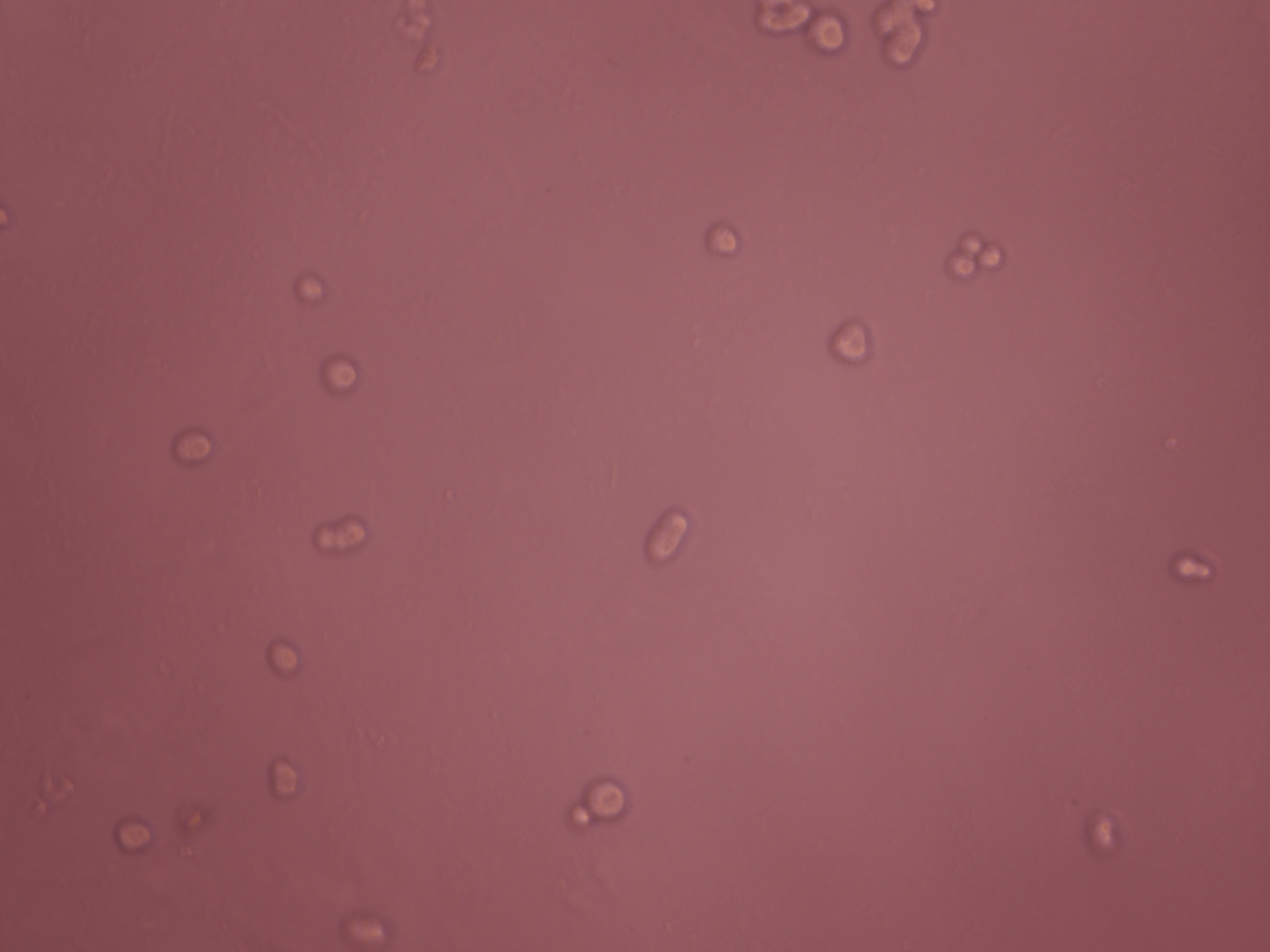

Supplement: Supplementary file 5 — Source data Fig. 4 [file 44319_2024_132_MOESM5_ESM.zip › Figure 4/4B/anoikis A549+P2.tif]

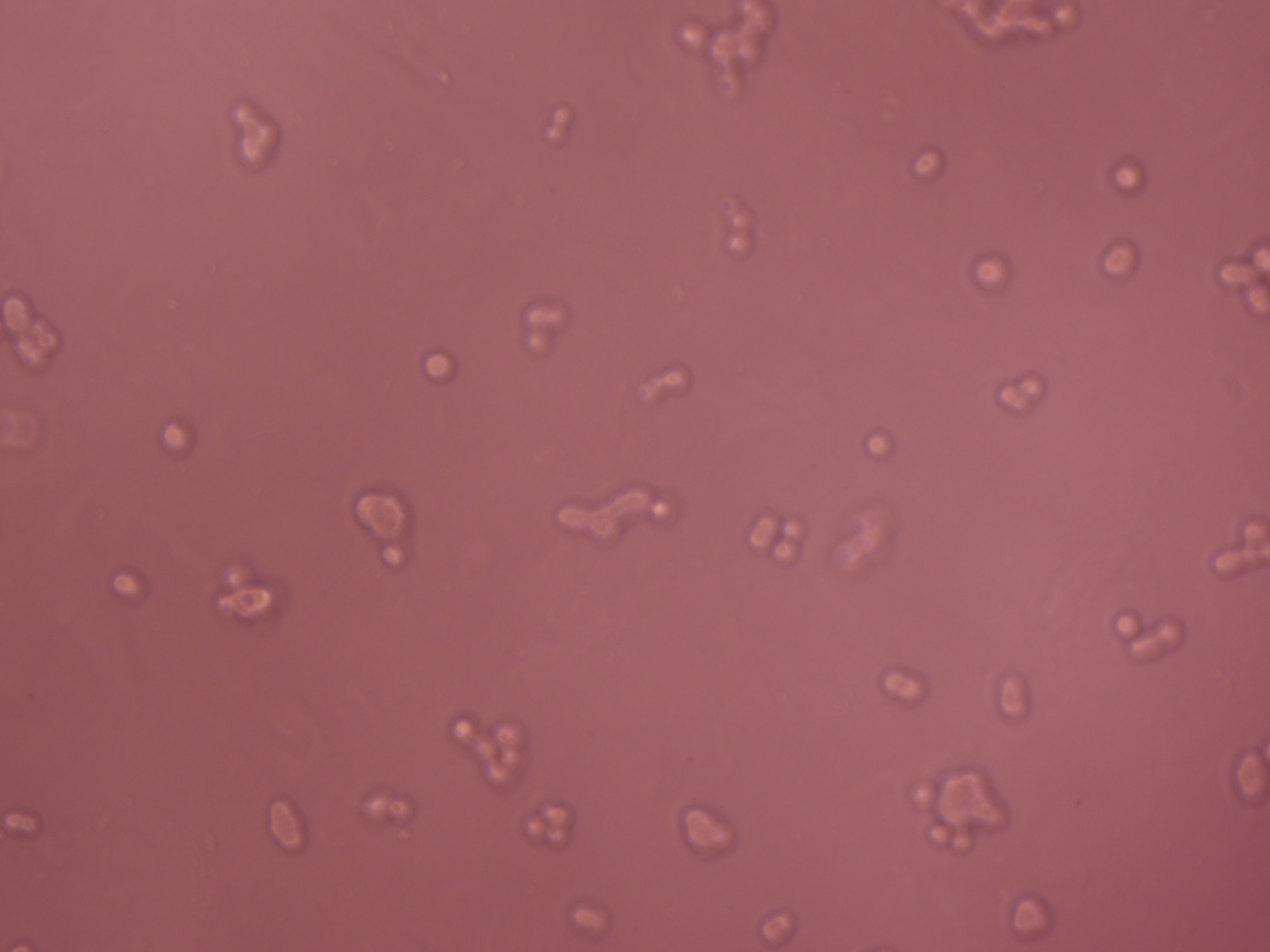

Supplement: Supplementary file 5 — Source data Fig. 4 [file 44319_2024_132_MOESM5_ESM.zip › Figure 4/4B/anoikis A549+P2S.tif]

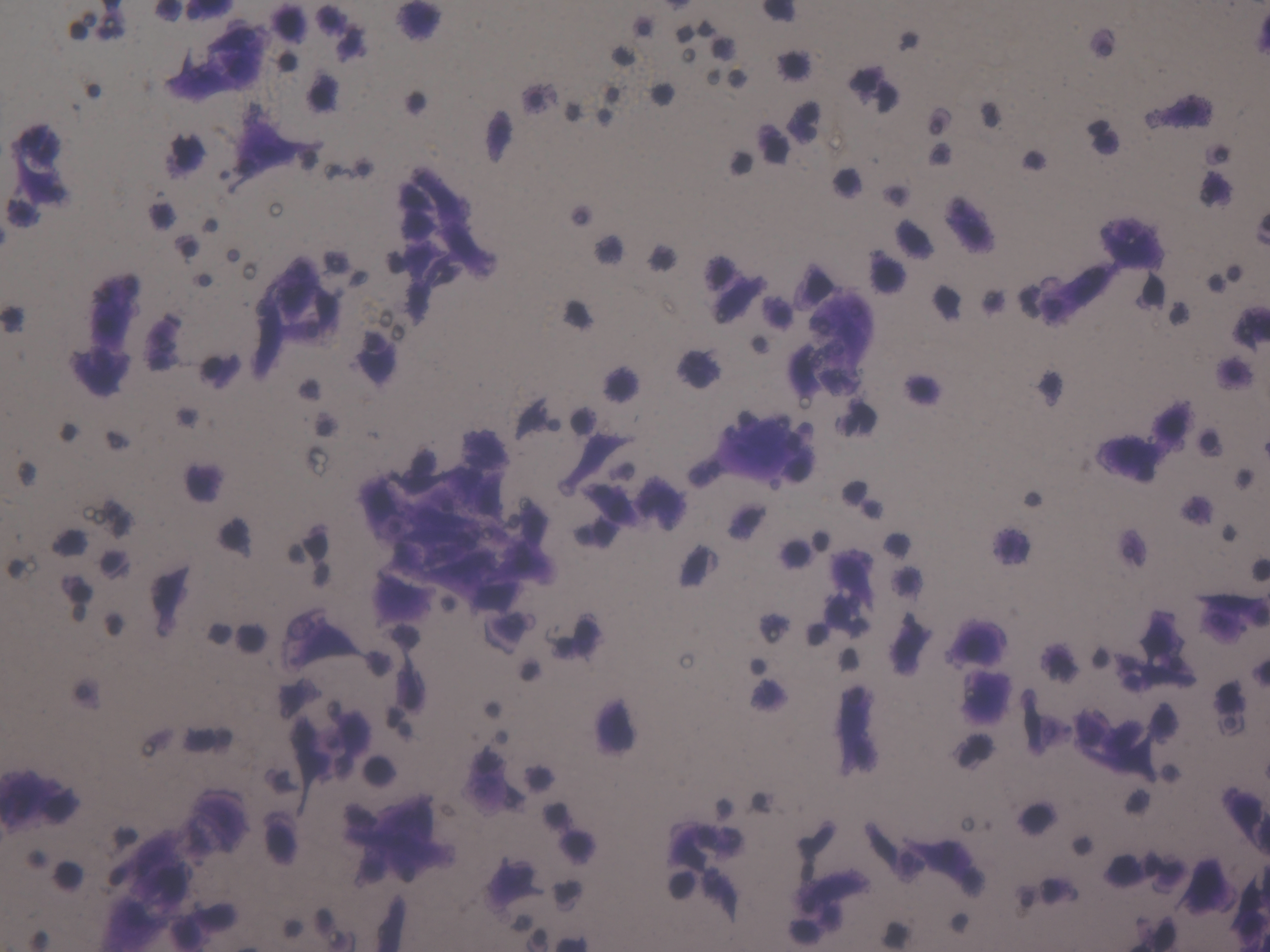

Supplement: Supplementary file 5 — Source data Fig. 4 [file 44319_2024_132_MOESM5_ESM.zip › Figure 4/4E/i/invasaion A549+P2.tif]

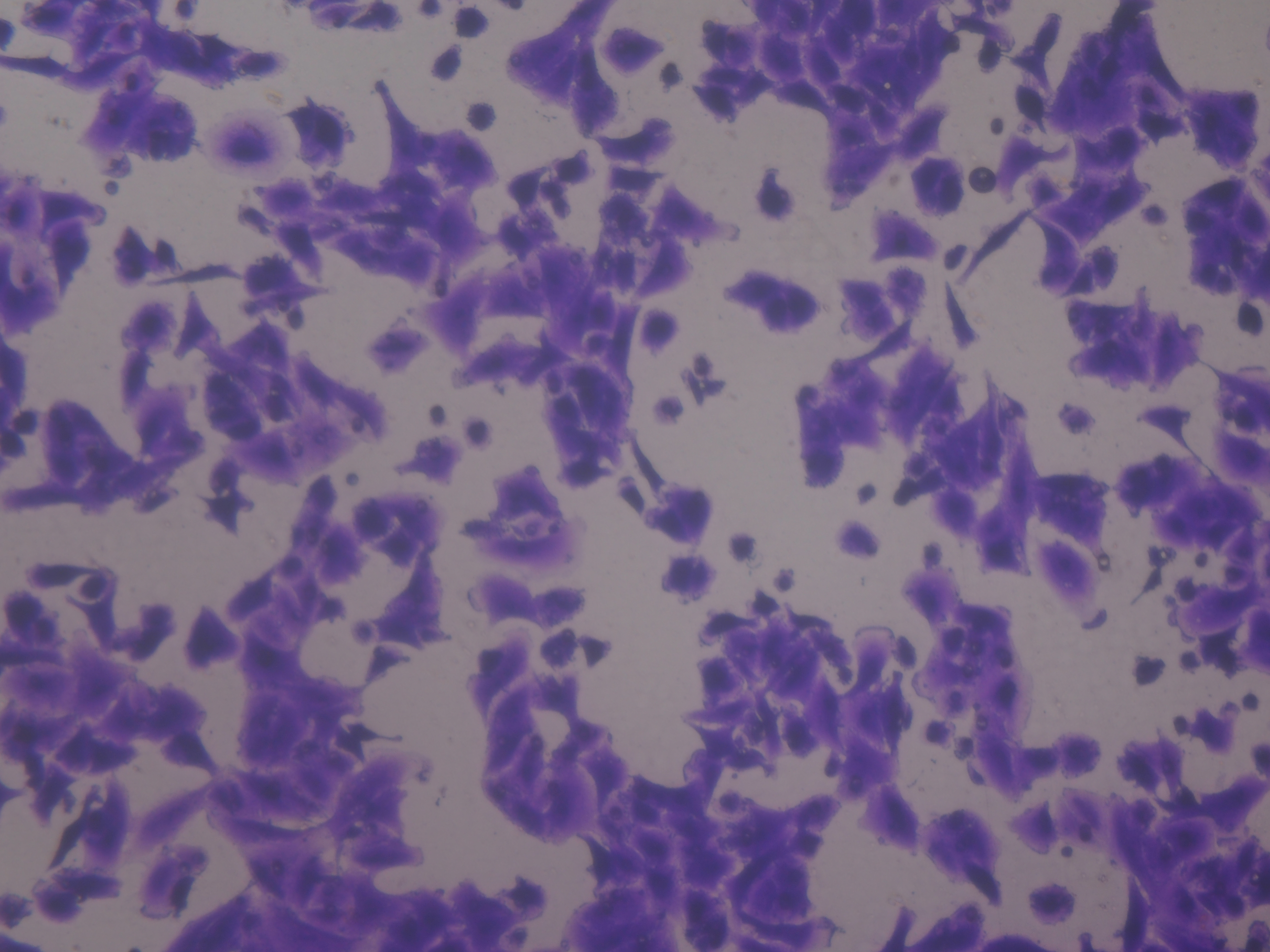

Supplement: Supplementary file 5 — Source data Fig. 4 [file 44319_2024_132_MOESM5_ESM.zip › Figure 4/4E/i/invasaion A549+P2S.tif]

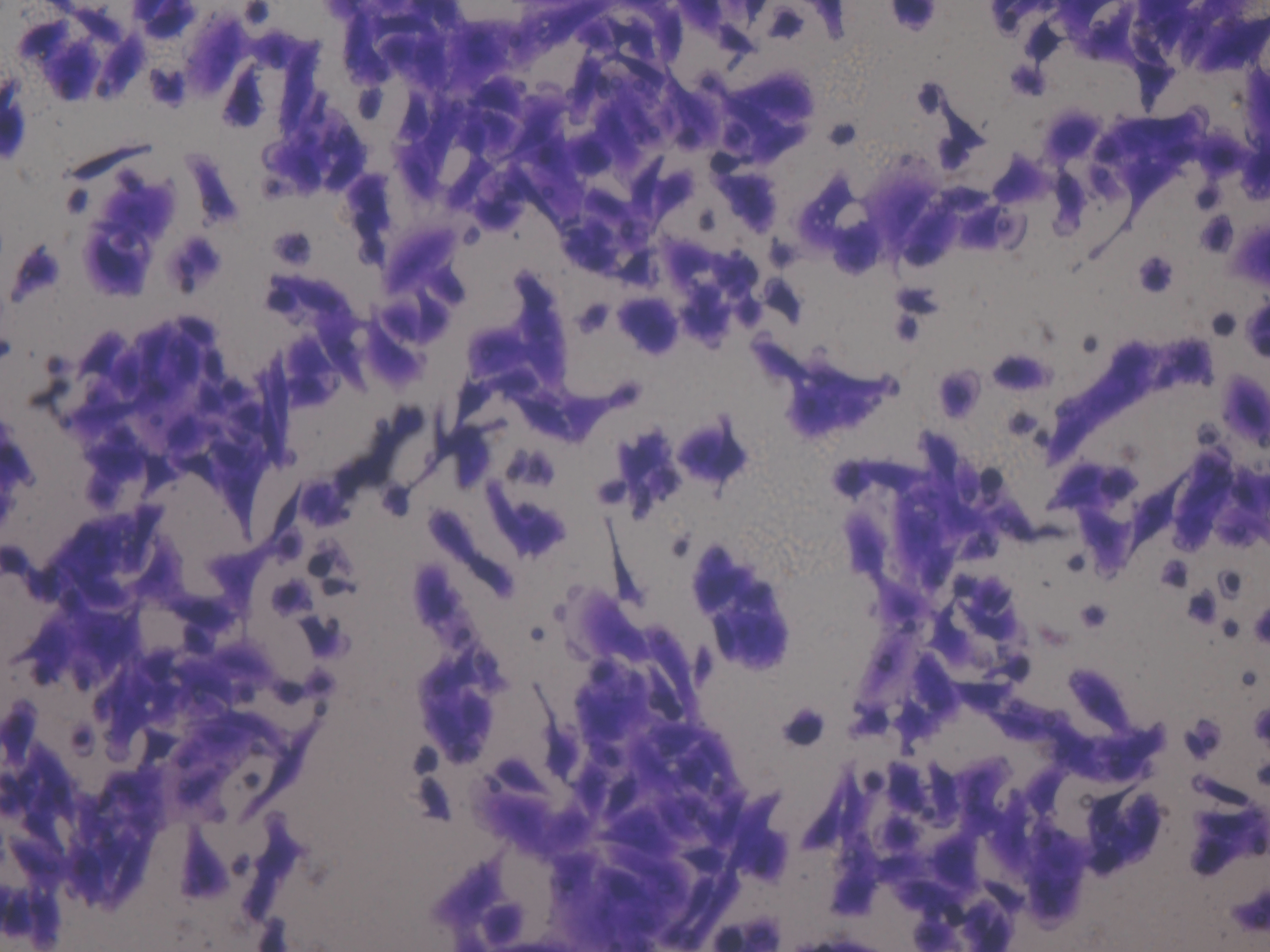

Supplement: Supplementary file 5 — Source data Fig. 4 [file 44319_2024_132_MOESM5_ESM.zip › Figure 4/4E/i/invasaion A549+PBS.tif]

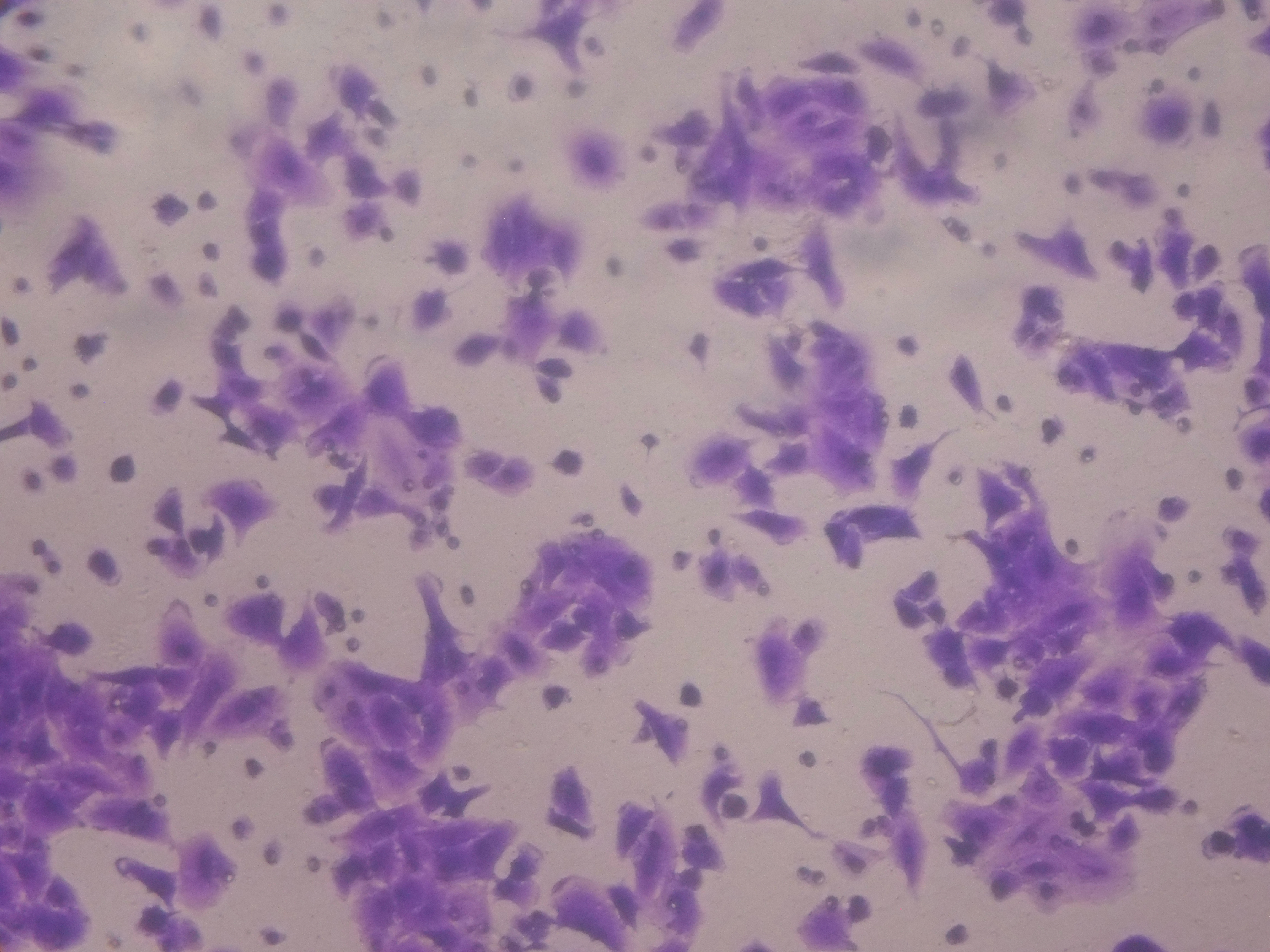

Supplement: Supplementary file 5 — Source data Fig. 4 [file 44319_2024_132_MOESM5_ESM.zip › Figure 4/4E/i/migration A549+P2.tif]

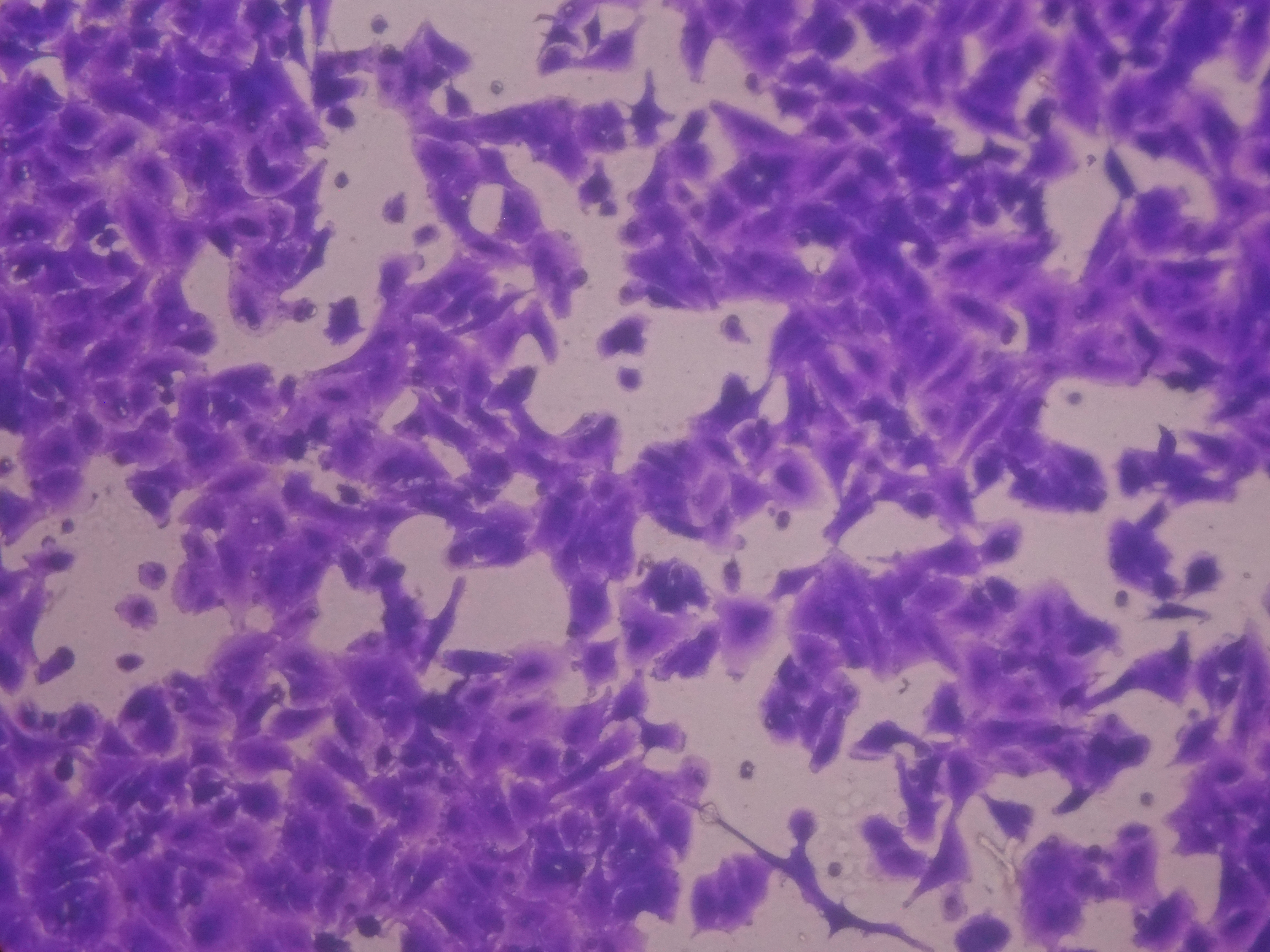

Supplement: Supplementary file 5 — Source data Fig. 4 [file 44319_2024_132_MOESM5_ESM.zip › Figure 4/4E/i/migration A549+P2S.tif]

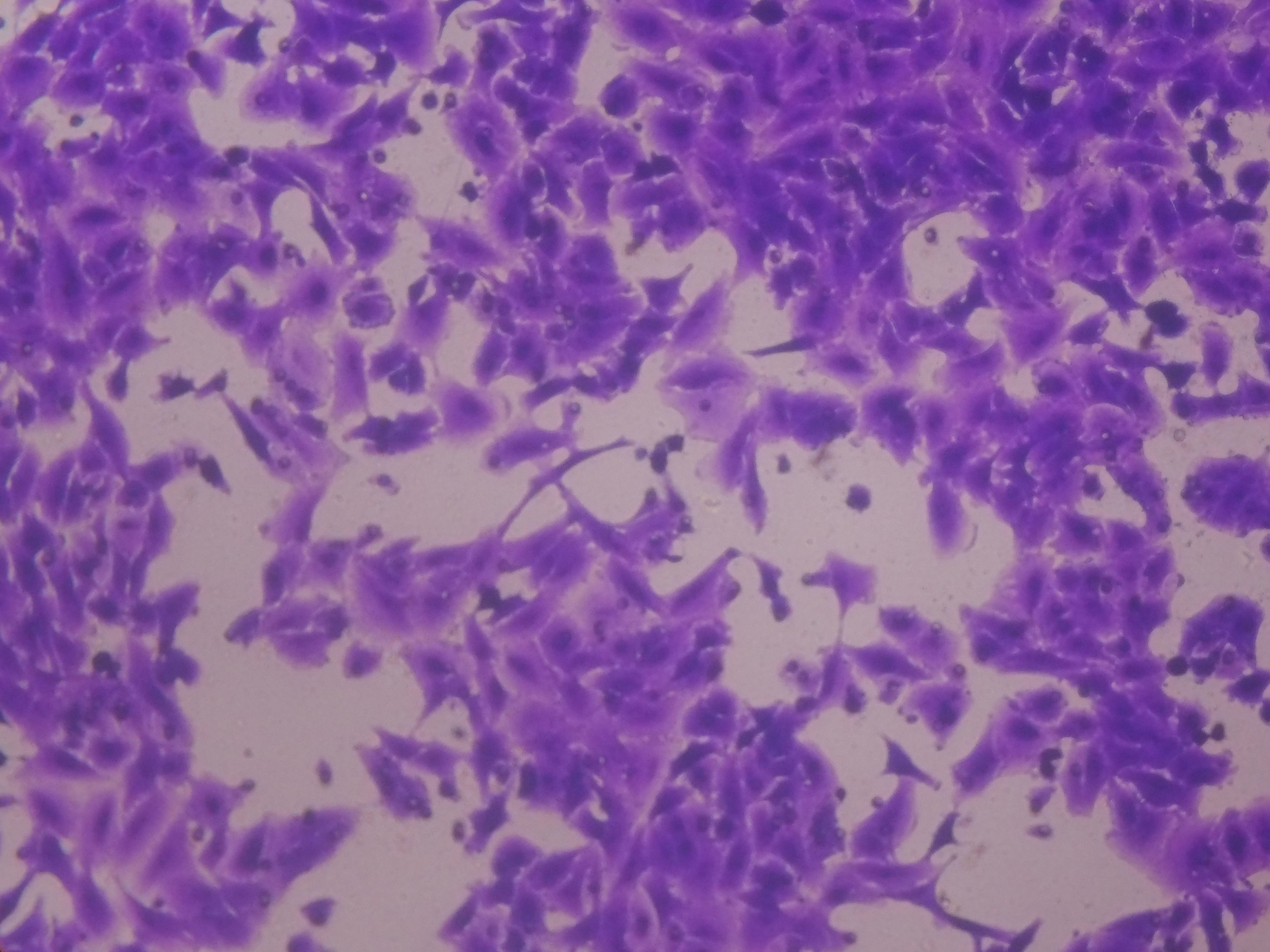

Supplement: Supplementary file 5 — Source data Fig. 4 [file 44319_2024_132_MOESM5_ESM.zip › Figure 4/4E/i/migration A549+PBS.tif]

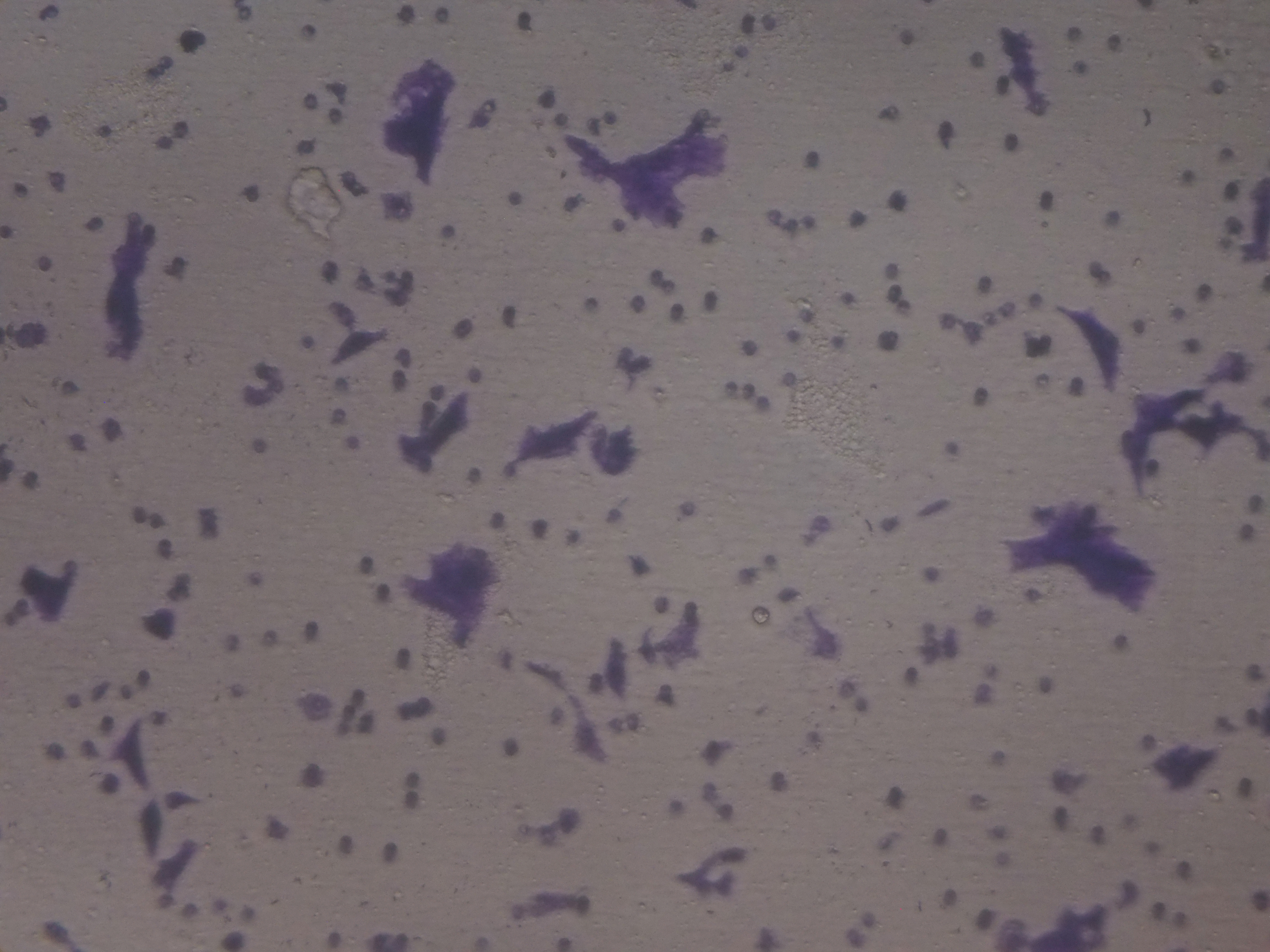

Supplement: Supplementary file 5 — Source data Fig. 4 [file 44319_2024_132_MOESM5_ESM.zip › Figure 4/4E/ii/invasaion HCC1806+P2.tif]

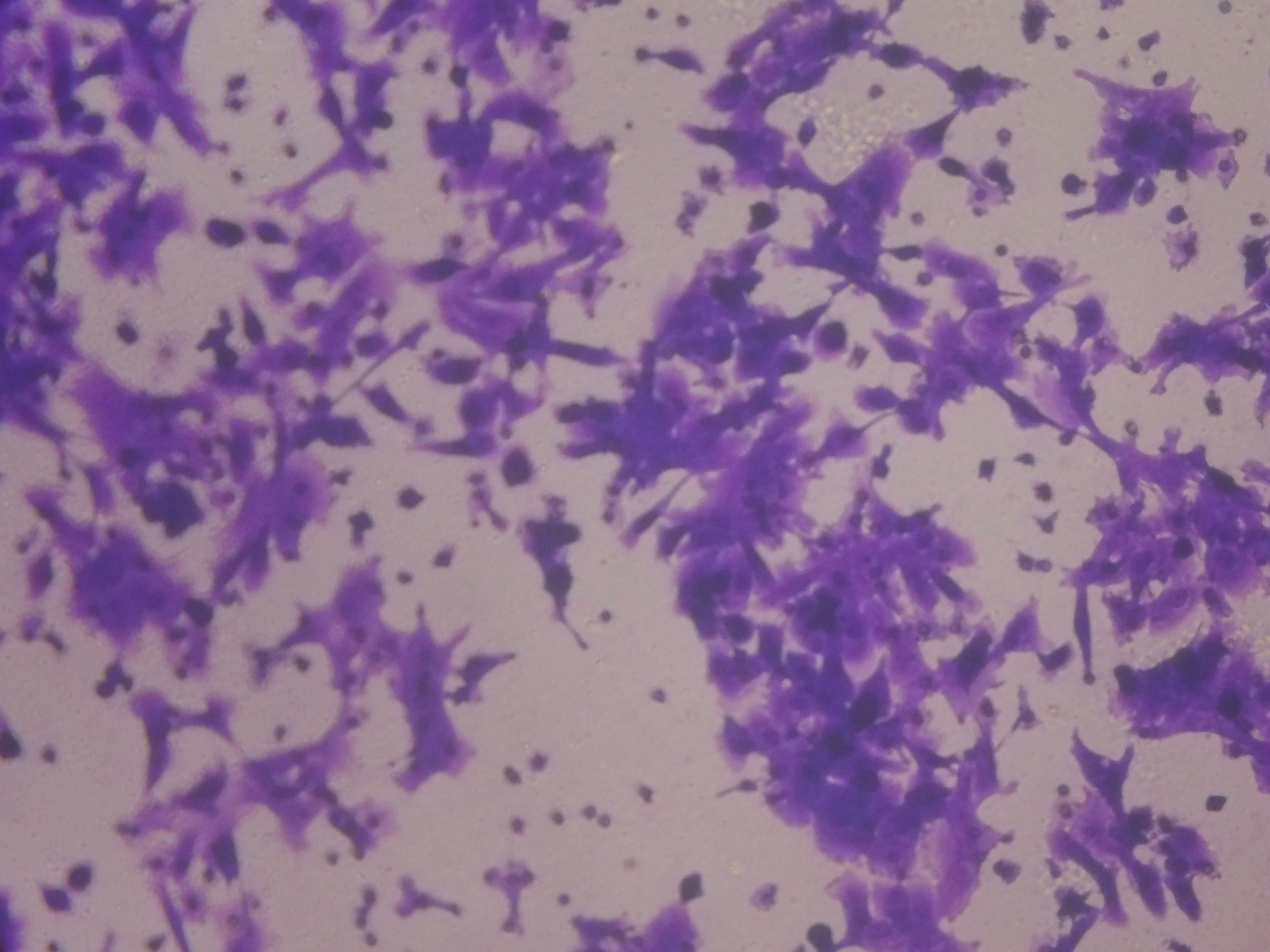

Supplement: Supplementary file 5 — Source data Fig. 4 [file 44319_2024_132_MOESM5_ESM.zip › Figure 4/4E/ii/invasaion HCC1806+P2S.tif]

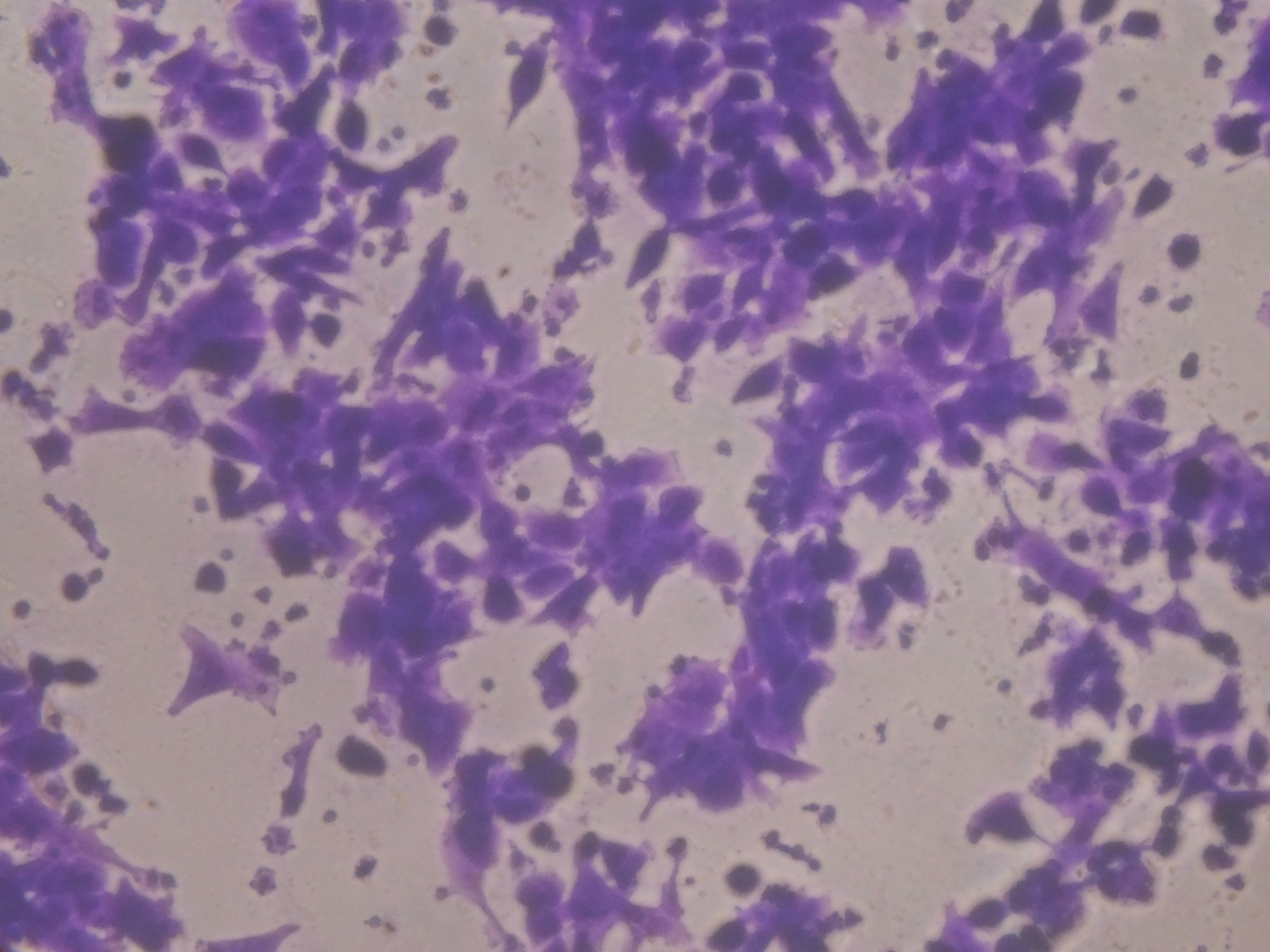

Supplement: Supplementary file 5 — Source data Fig. 4 [file 44319_2024_132_MOESM5_ESM.zip › Figure 4/4E/ii/invasaion HCC1806+PBS.tif]

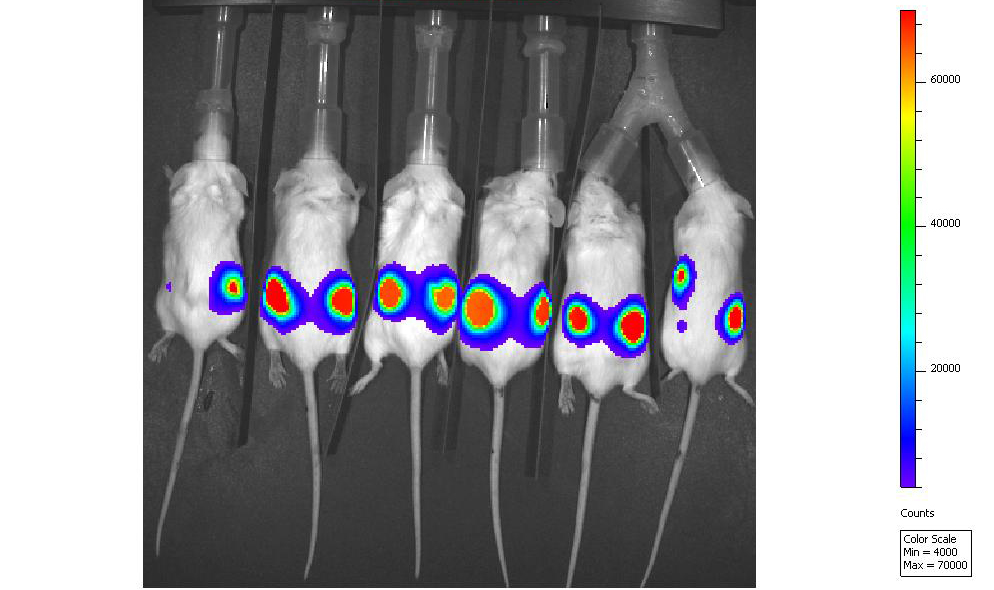

Supplement: Supplementary file 6 — Source data Fig. 5 [file 44319_2024_132_MOESM6_ESM.zip › Figure 5/5B/IAA mice IVIS image.jpg]

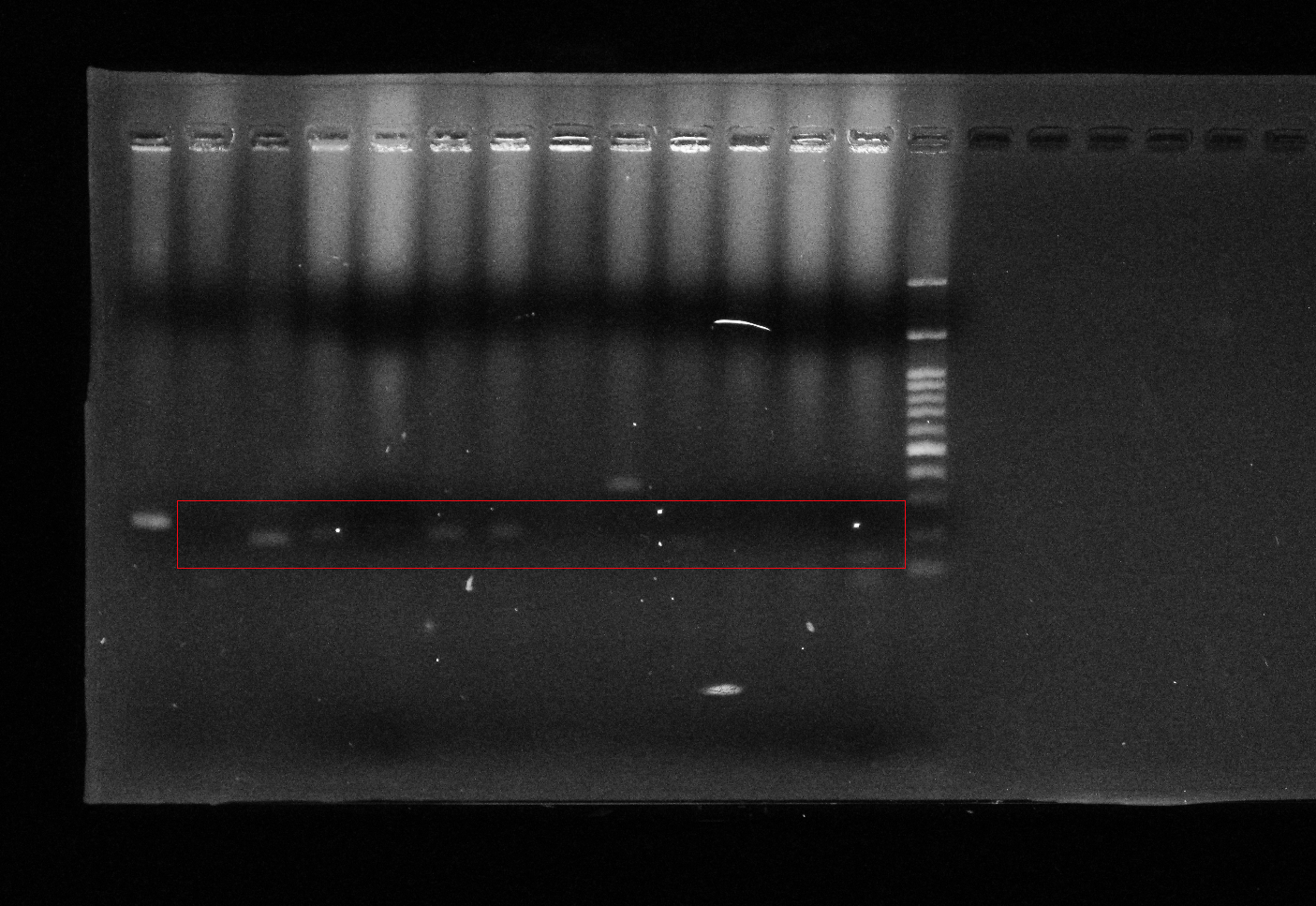

Supplement: Supplementary file 6 — Source data Fig. 5 [file 44319_2024_132_MOESM6_ESM.zip › Figure 5/5B/Lung hGAPDH PCR.tif]

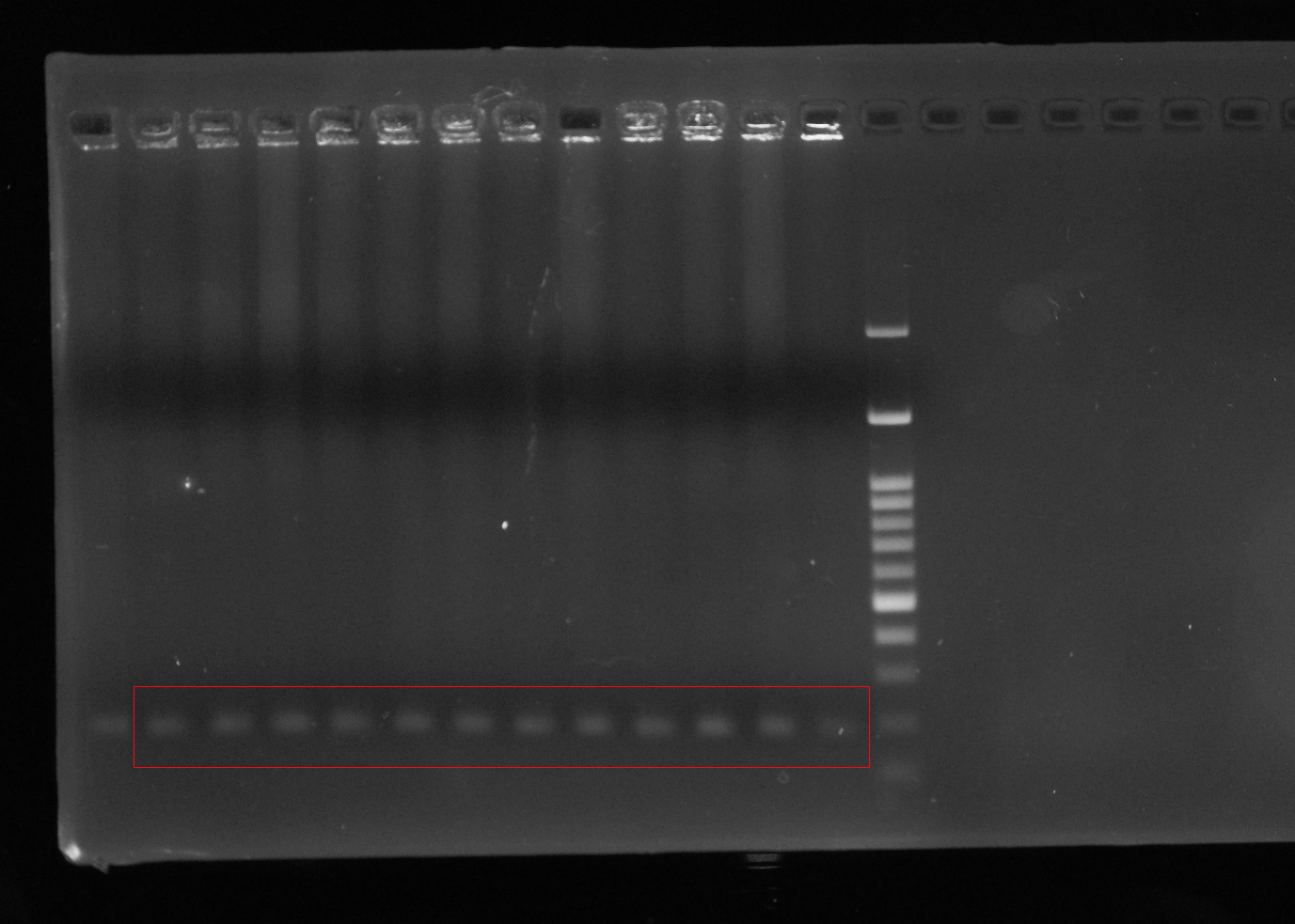

Supplement: Supplementary file 6 — Source data Fig. 5 [file 44319_2024_132_MOESM6_ESM.zip › Figure 5/5B/Lung mGAPDH PCR.tif]

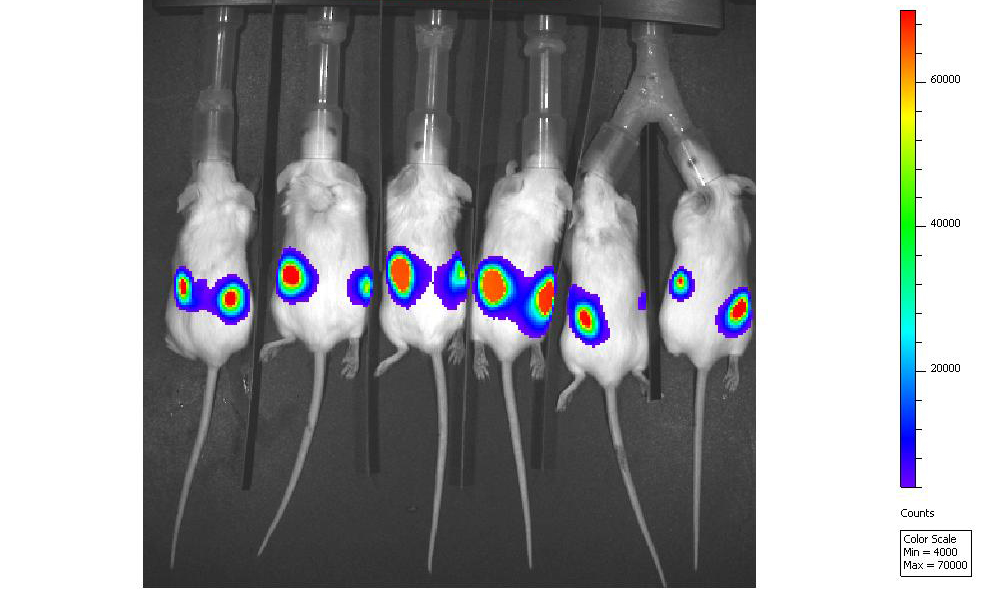

Supplement: Supplementary file 6 — Source data Fig. 5 [file 44319_2024_132_MOESM6_ESM.zip › Figure 5/5B/PBS mice IVIS image.jpg]

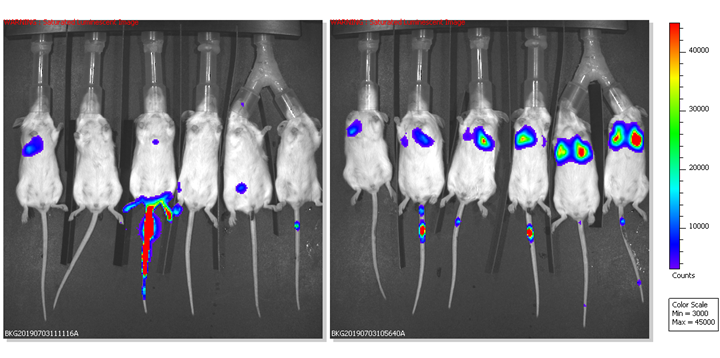

Supplement: Supplementary file 6 — Source data Fig. 5 [file 44319_2024_132_MOESM6_ESM.zip › Figure 5/5E/i/lung tumor IVIS image.tif]

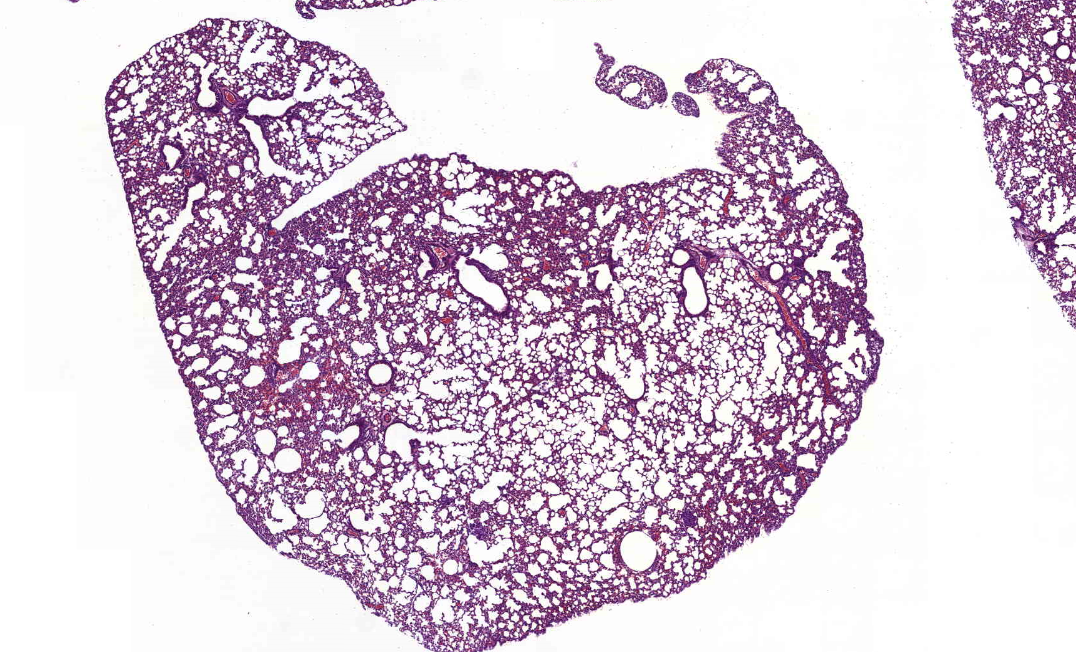

Supplement: Supplementary file 6 — Source data Fig. 5 [file 44319_2024_132_MOESM6_ESM.zip › Figure 5/5E/iii/IAA.bmp]

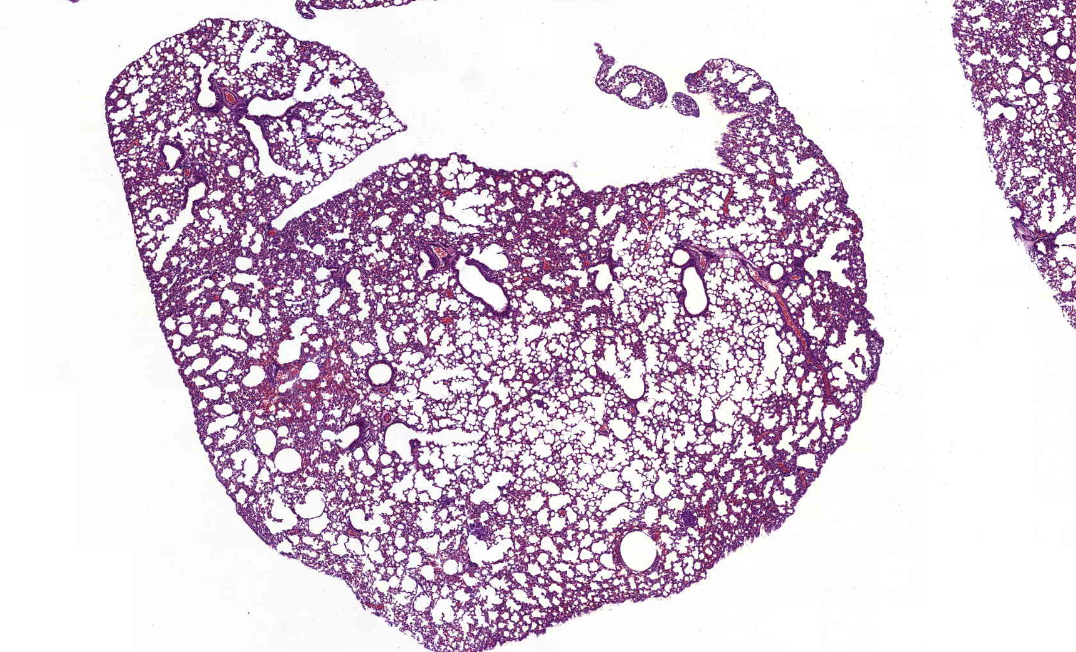

Supplement: Supplementary file 6 — Source data Fig. 5 [file 44319_2024_132_MOESM6_ESM.zip › Figure 5/5E/iii/IAA.tif]

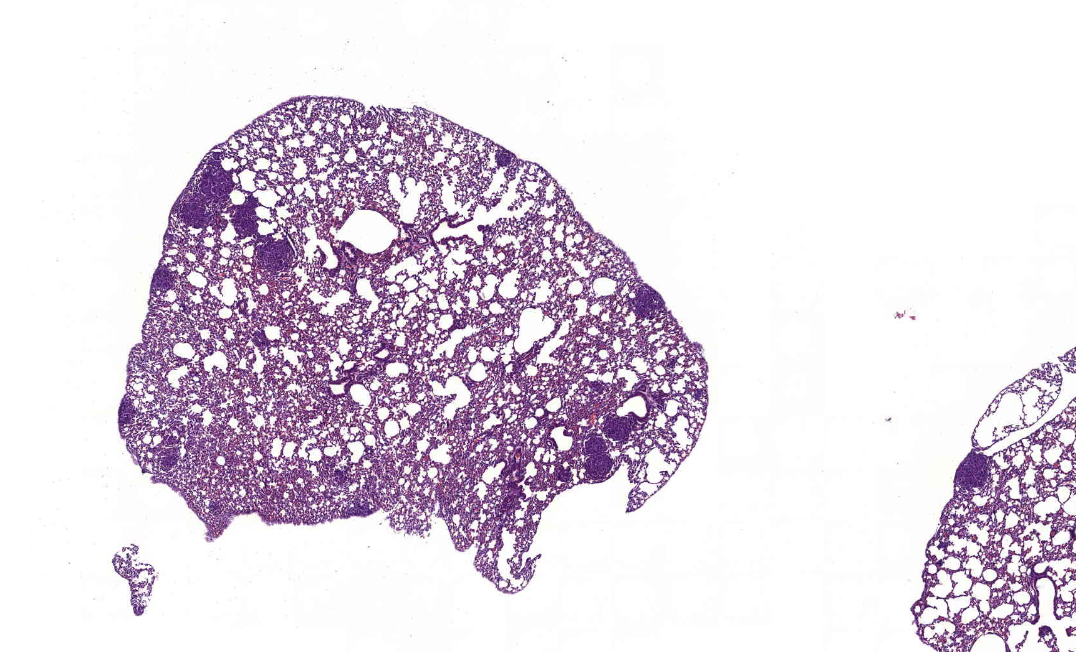

Supplement: Supplementary file 6 — Source data Fig. 5 [file 44319_2024_132_MOESM6_ESM.zip › Figure 5/5E/iii/PBS.bmp]

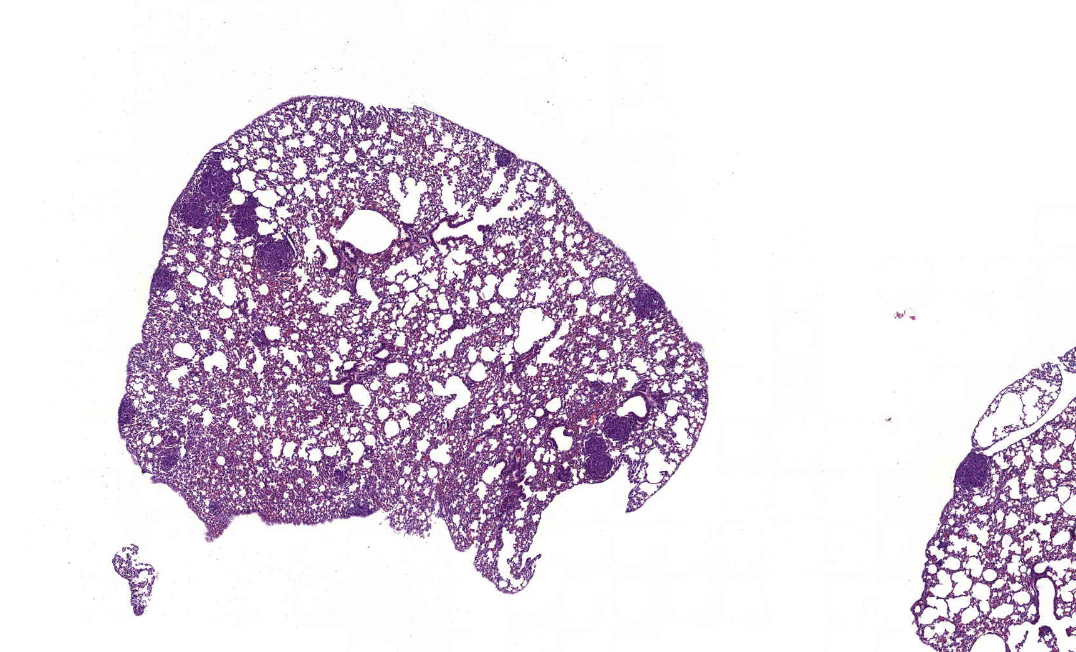

Supplement: Supplementary file 6 — Source data Fig. 5 [file 44319_2024_132_MOESM6_ESM.zip › Figure 5/5E/iii/PBS.tif]

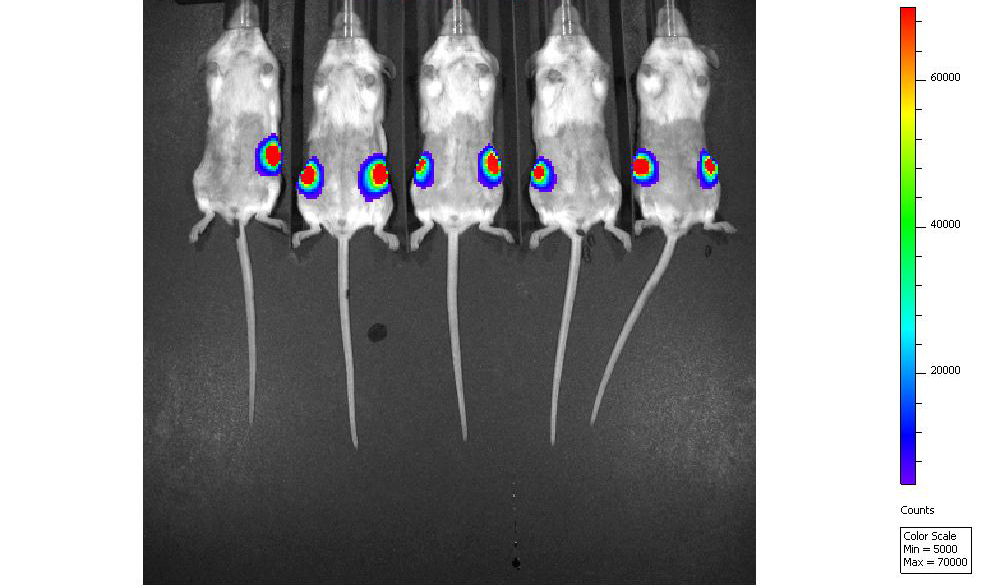

Supplement: Supplementary file 6 — Source data Fig. 5 [file 44319_2024_132_MOESM6_ESM.zip › Figure 5/5F/i/IAA mice IVIS-1.tif]

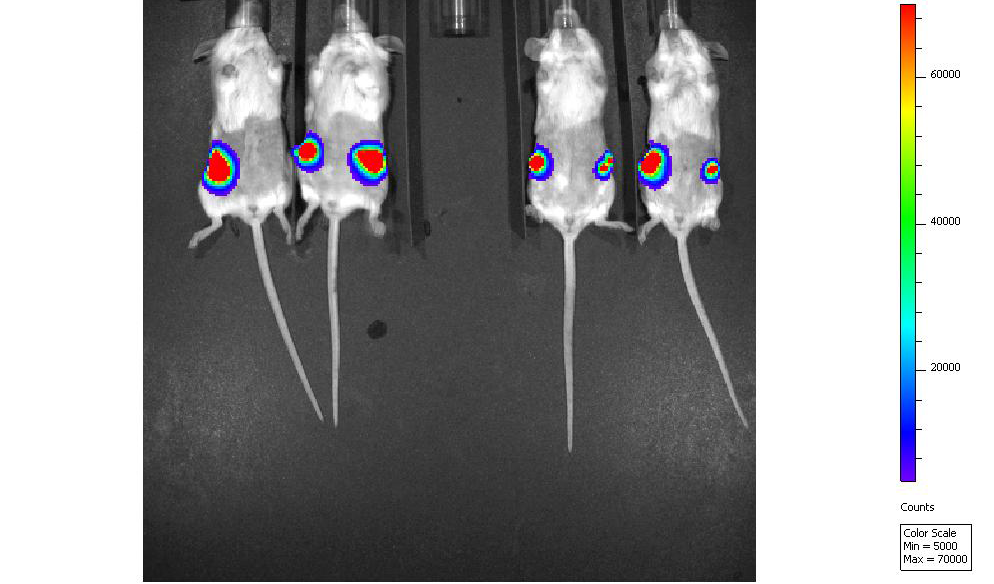

Supplement: Supplementary file 6 — Source data Fig. 5 [file 44319_2024_132_MOESM6_ESM.zip › Figure 5/5F/i/PBS IAA mice IVIS-2.tif]

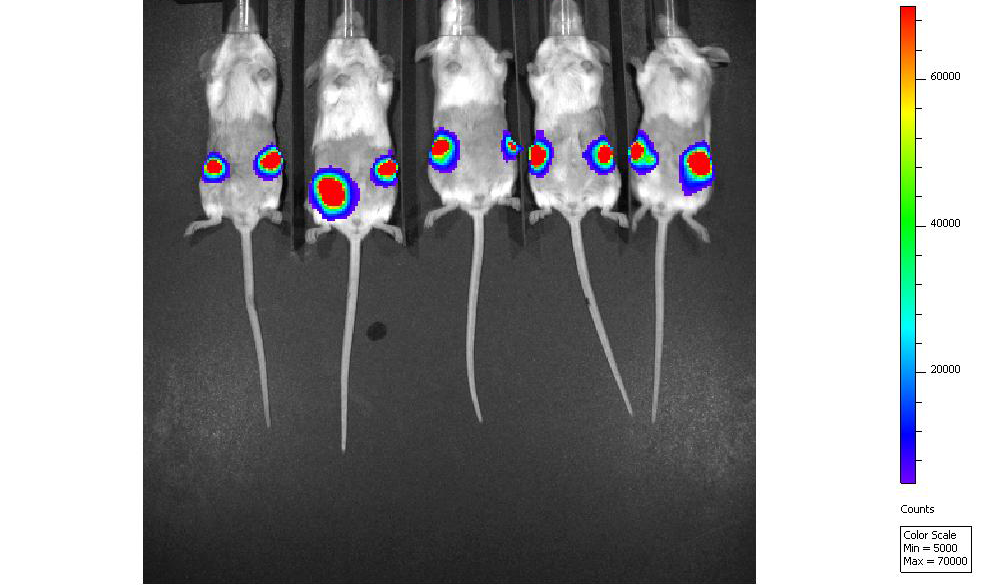

Supplement: Supplementary file 6 — Source data Fig. 5 [file 44319_2024_132_MOESM6_ESM.zip › Figure 5/5F/i/PBS mice IVIS-1.tif]

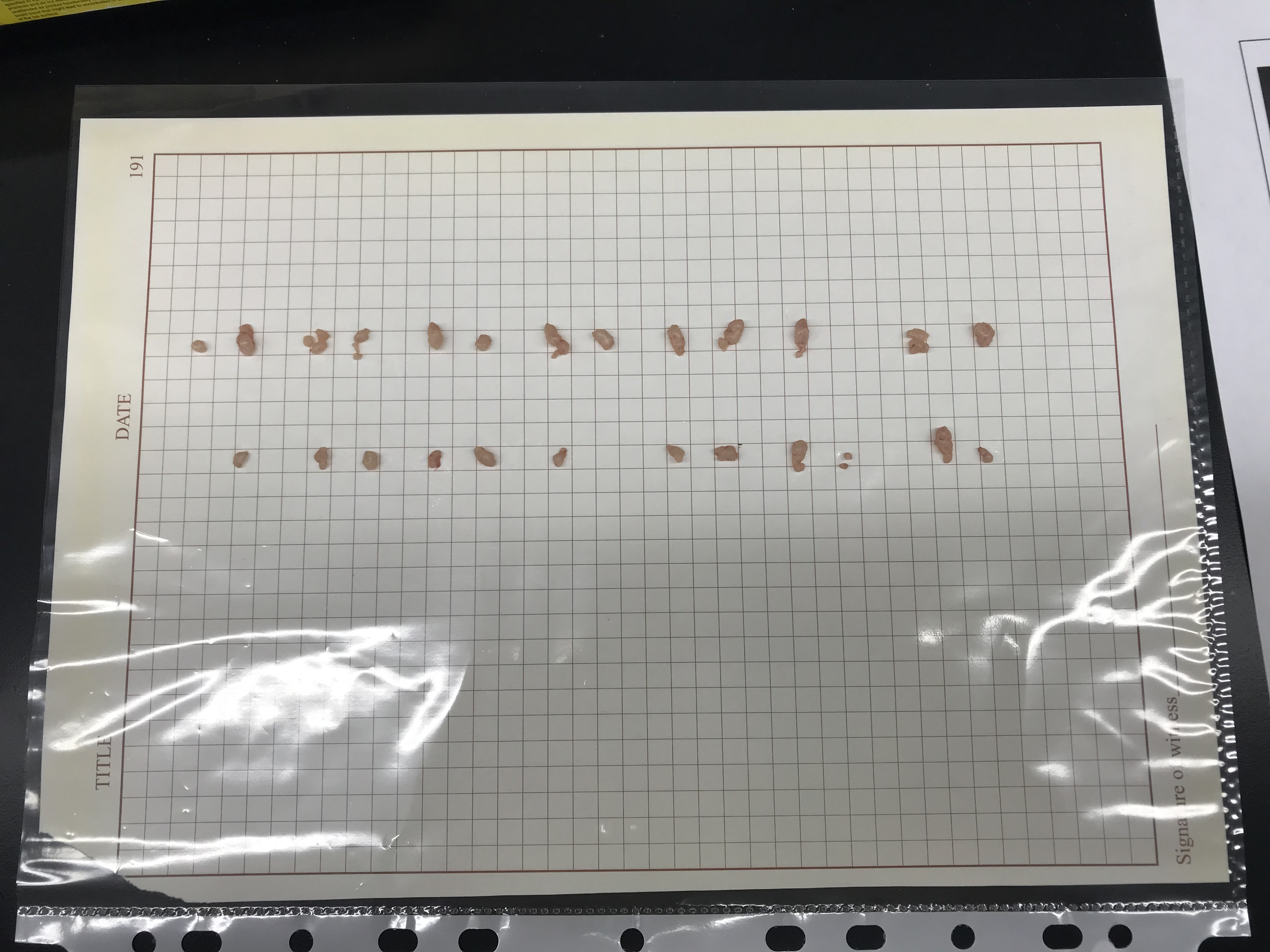

Supplement: Supplementary file 6 — Source data Fig. 5 [file 44319_2024_132_MOESM6_ESM.zip › Figure 5/5F/i/tumor picture.tif]

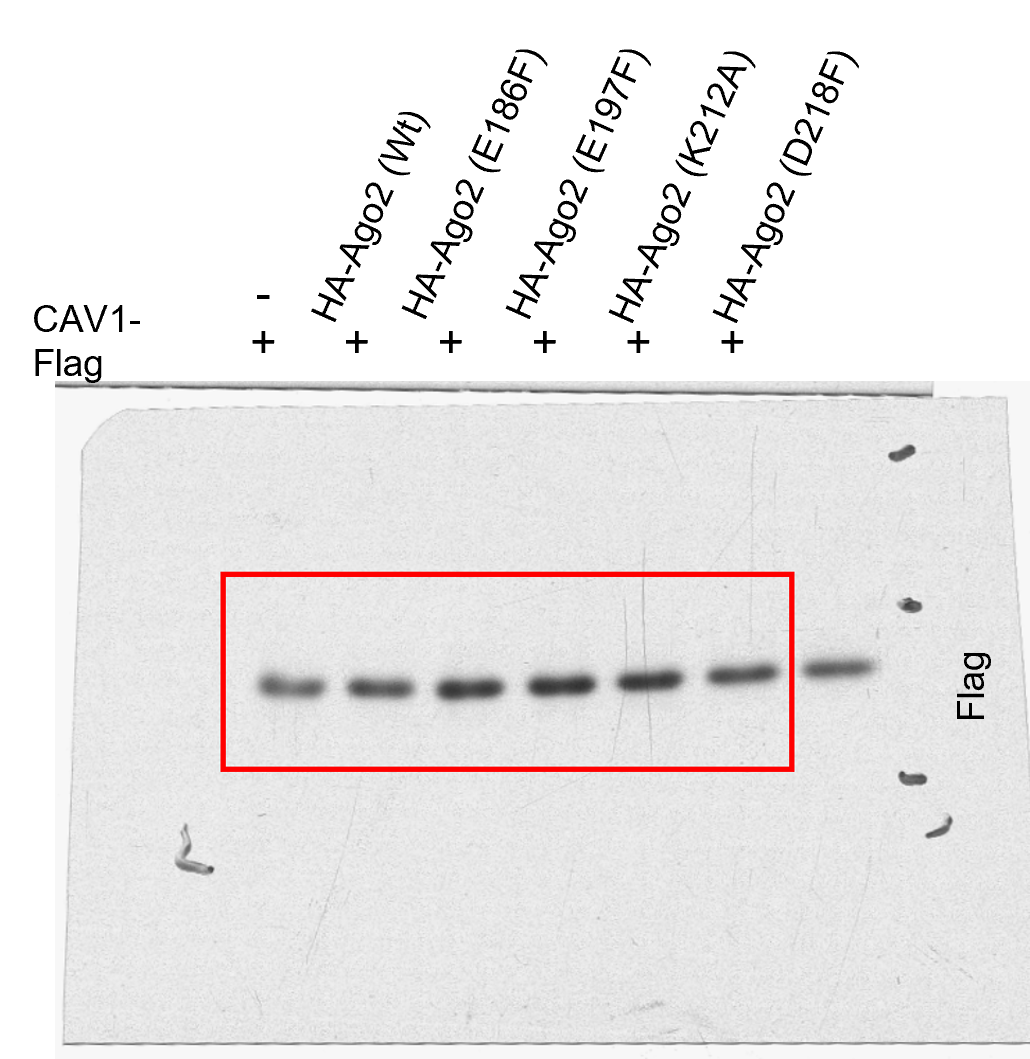

Supplement: Supplementary file 7 — Source data Fig. 6 [file 44319_2024_132_MOESM7_ESM.zip › Figure 6/6A/western input Flag-CAV1.tif]

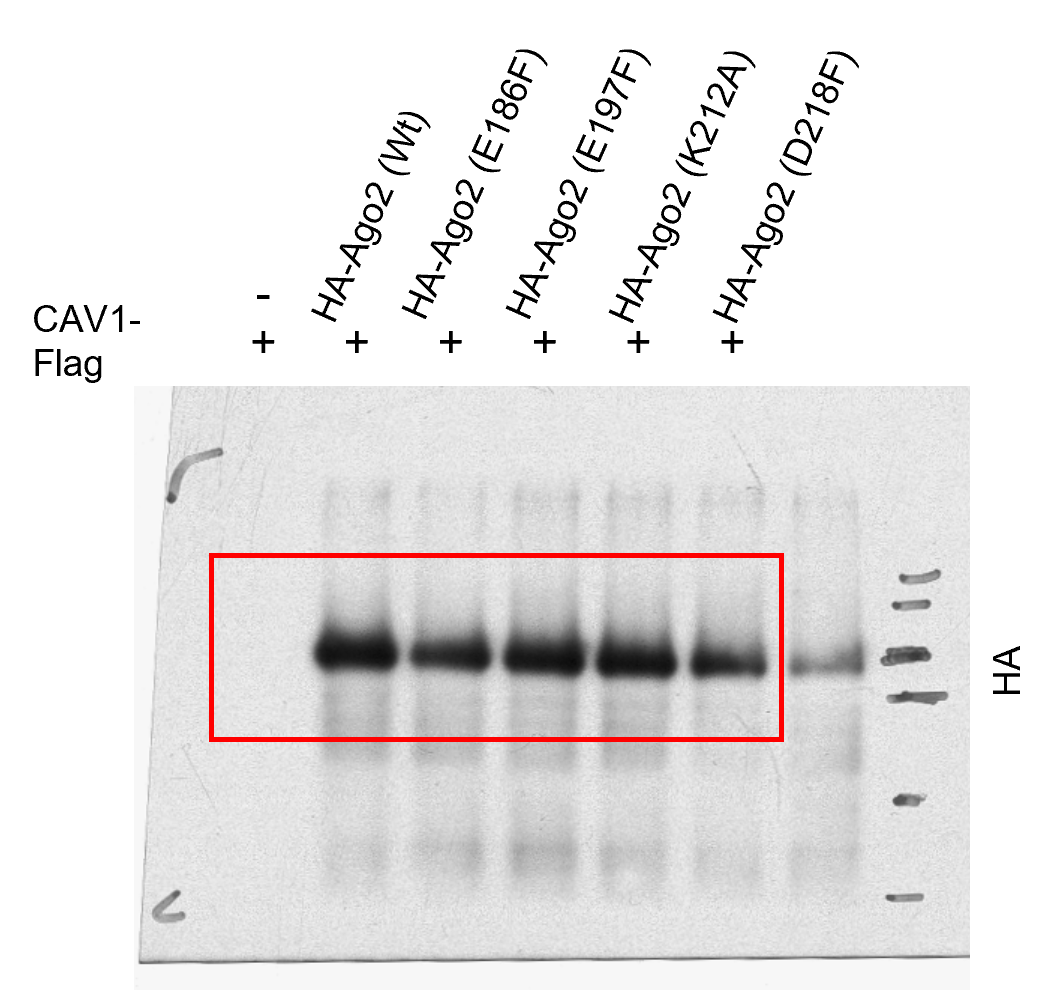

Supplement: Supplementary file 7 — Source data Fig. 6 [file 44319_2024_132_MOESM7_ESM.zip › Figure 6/6A/western input HA-Ago2.tif]

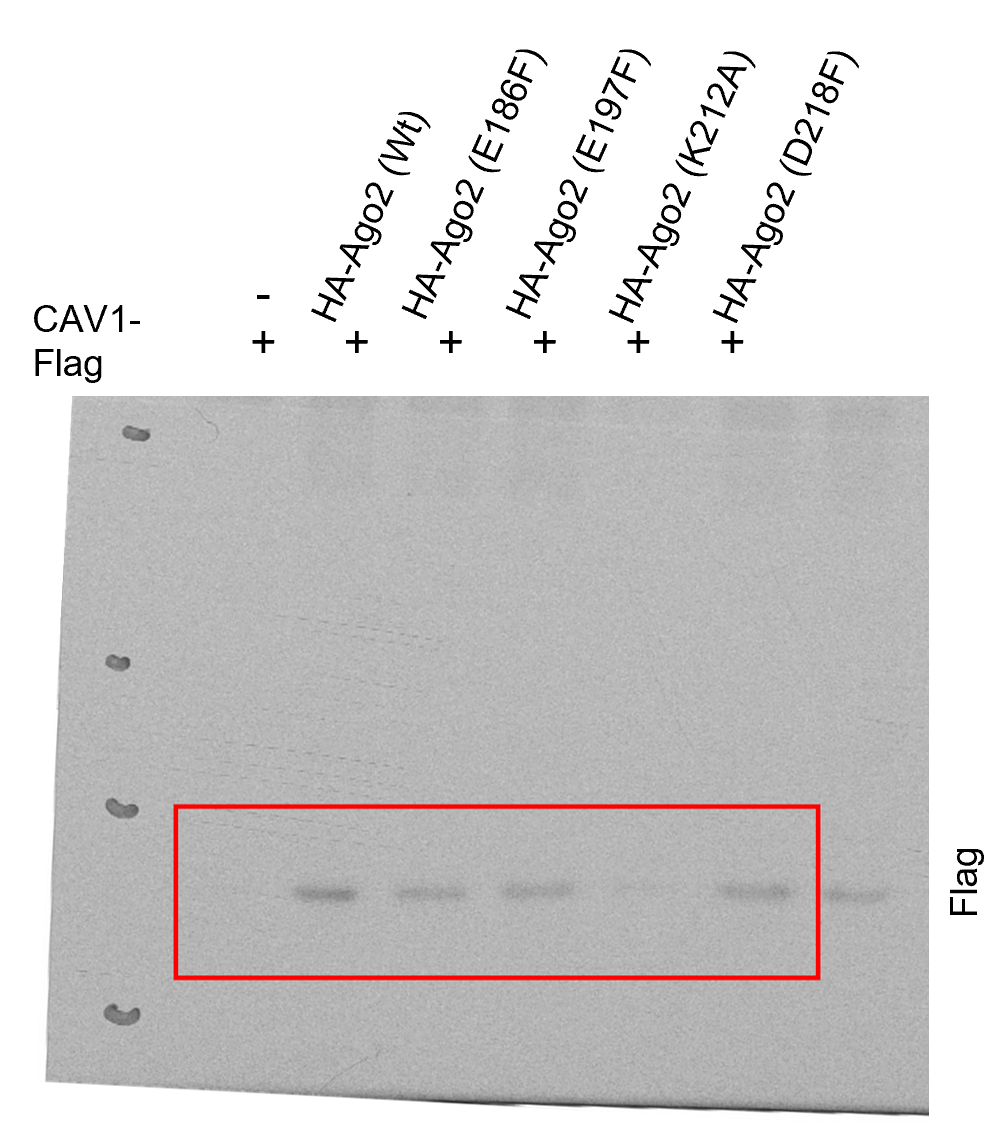

Supplement: Supplementary file 7 — Source data Fig. 6 [file 44319_2024_132_MOESM7_ESM.zip › Figure 6/6A/western IP Flag-CAV1.tif]

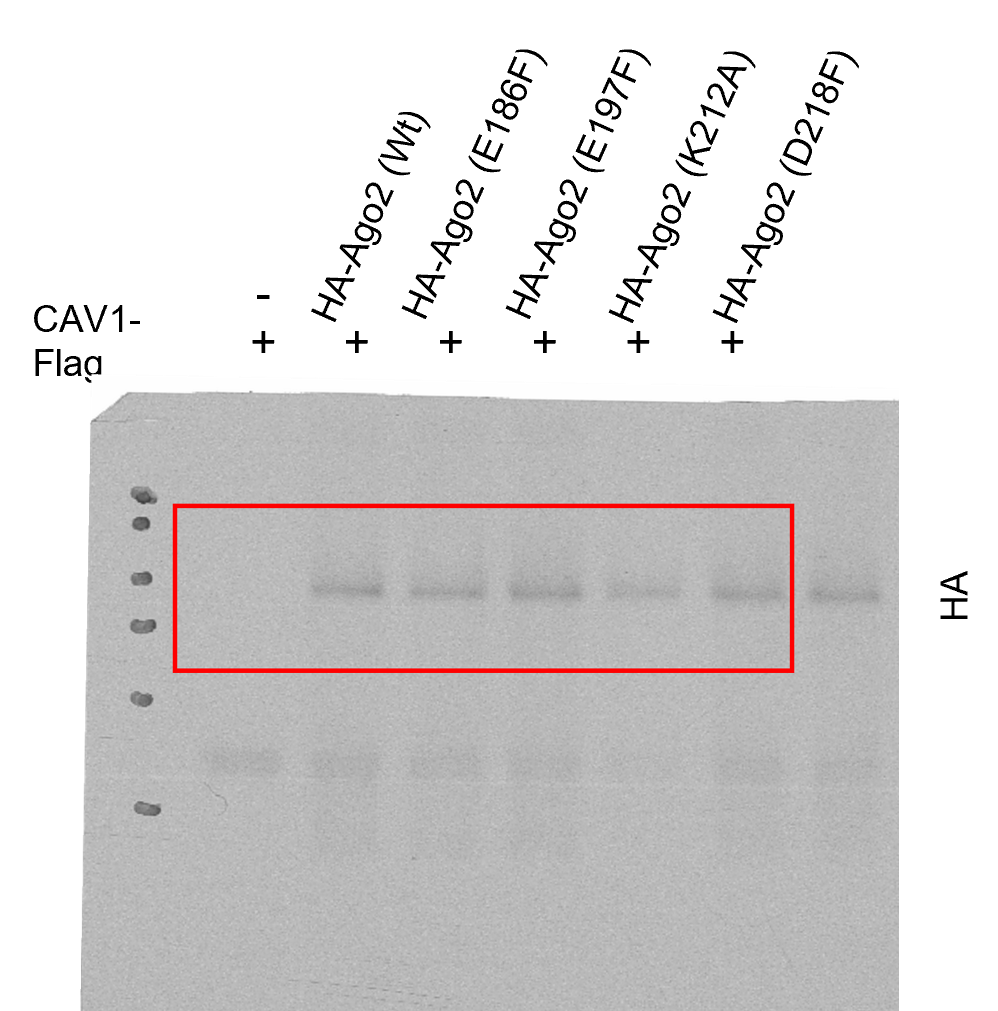

Supplement: Supplementary file 7 — Source data Fig. 6 [file 44319_2024_132_MOESM7_ESM.zip › Figure 6/6A/western IP HA-Ago2.tif]

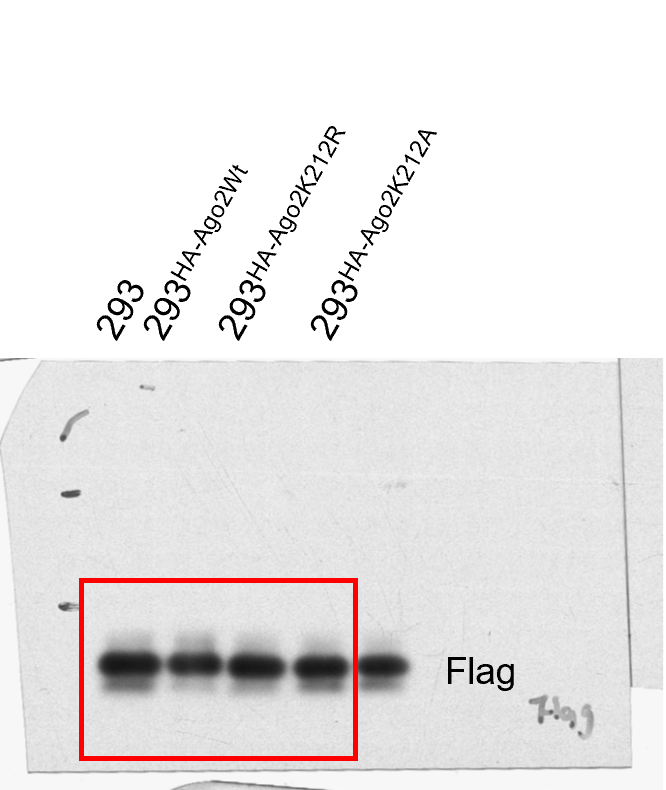

Supplement: Supplementary file 7 — Source data Fig. 6 [file 44319_2024_132_MOESM7_ESM.zip › Figure 6/6B/i/western input Flag-CAV1.tif]

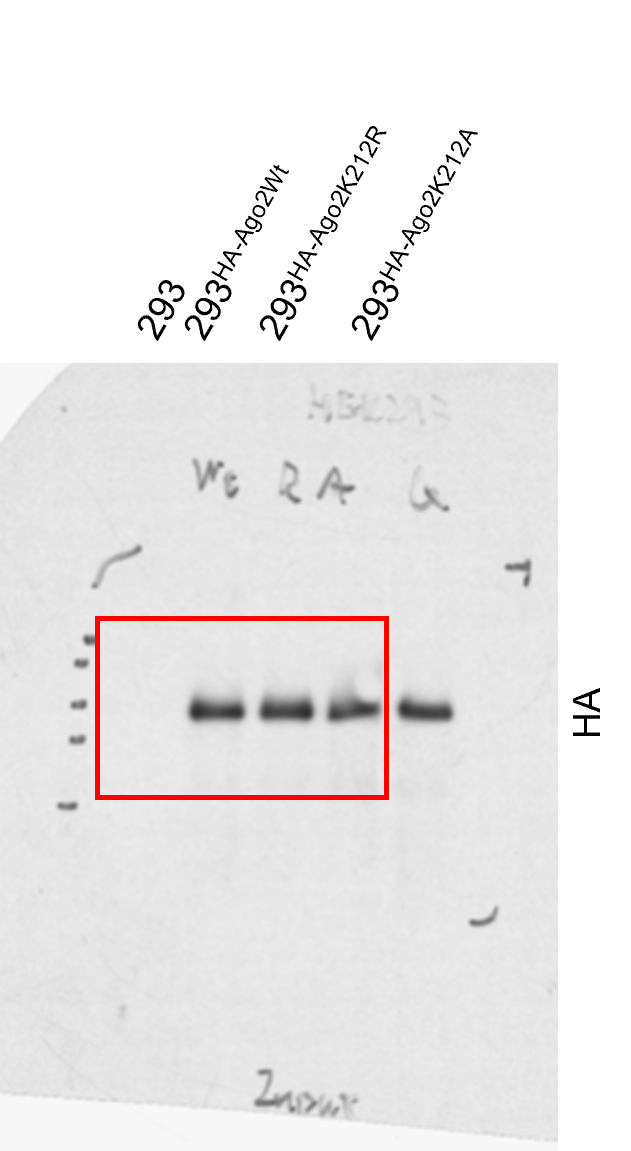

Supplement: Supplementary file 7 — Source data Fig. 6 [file 44319_2024_132_MOESM7_ESM.zip › Figure 6/6B/i/western input HA-Ago2.tif]

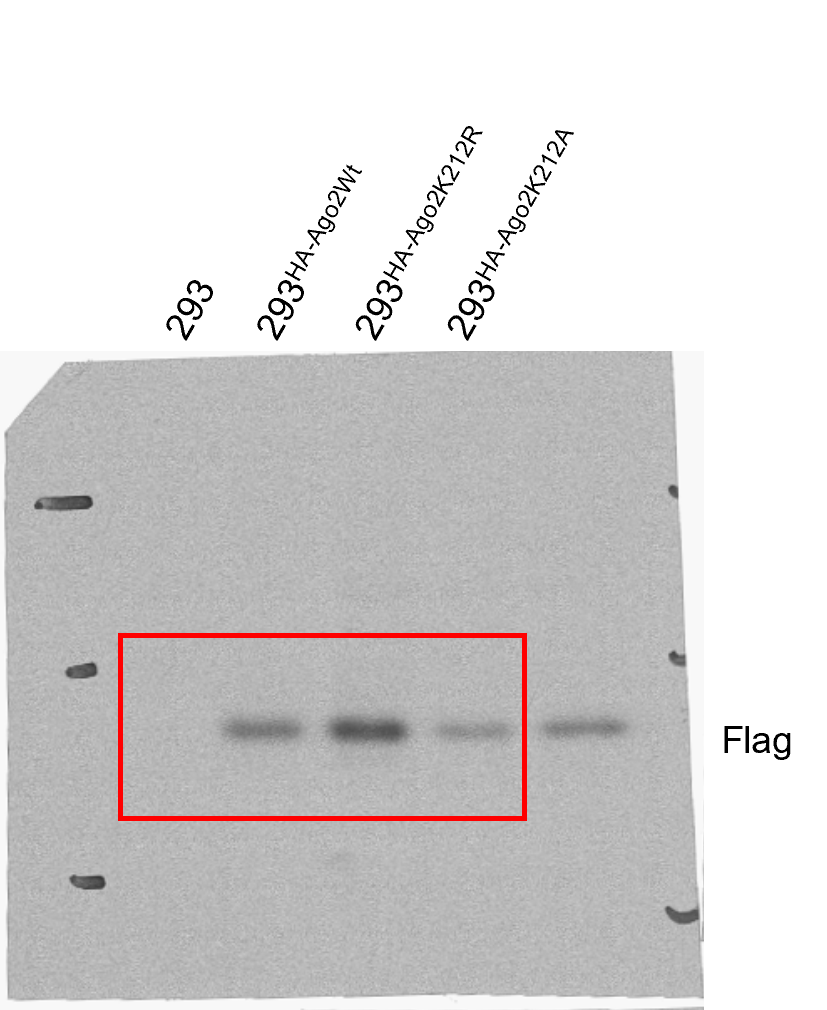

Supplement: Supplementary file 7 — Source data Fig. 6 [file 44319_2024_132_MOESM7_ESM.zip › Figure 6/6B/i/western IP Flag-CAV1.tif]

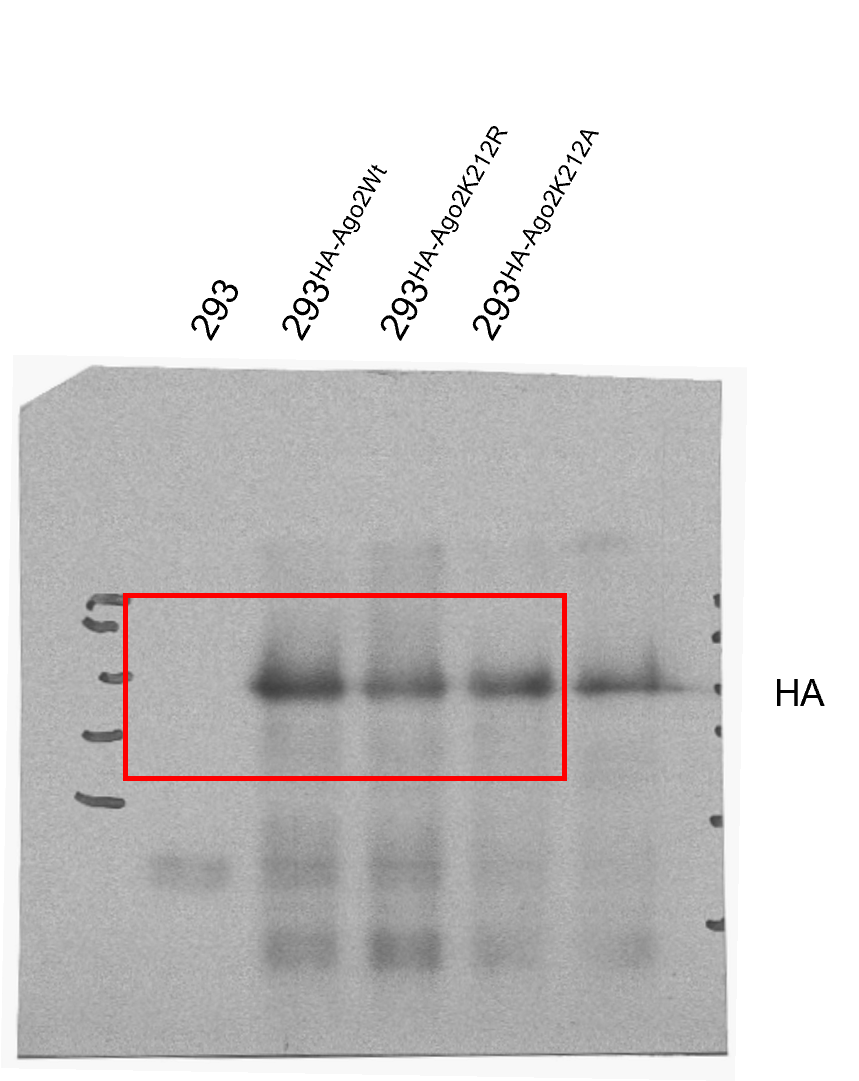

Supplement: Supplementary file 7 — Source data Fig. 6 [file 44319_2024_132_MOESM7_ESM.zip › Figure 6/6B/i/western IP HA-Ago2.tif]

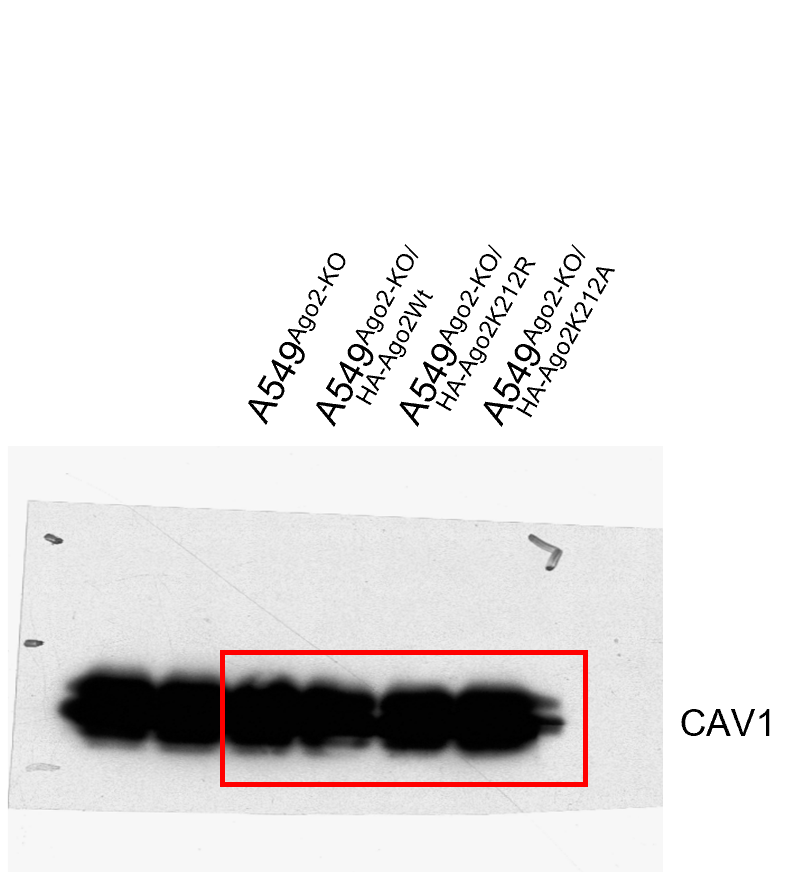

Supplement: Supplementary file 7 — Source data Fig. 6 [file 44319_2024_132_MOESM7_ESM.zip › Figure 6/6B/ii/western input CAV1.tif]

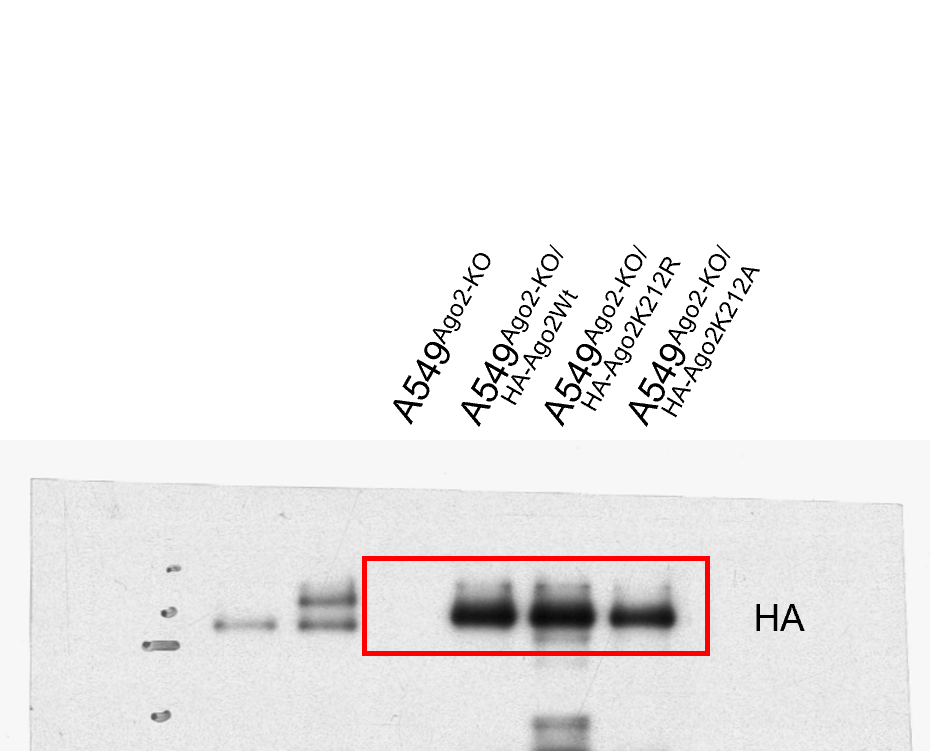

Supplement: Supplementary file 7 — Source data Fig. 6 [file 44319_2024_132_MOESM7_ESM.zip › Figure 6/6B/ii/western input HA-Ago2.tif]

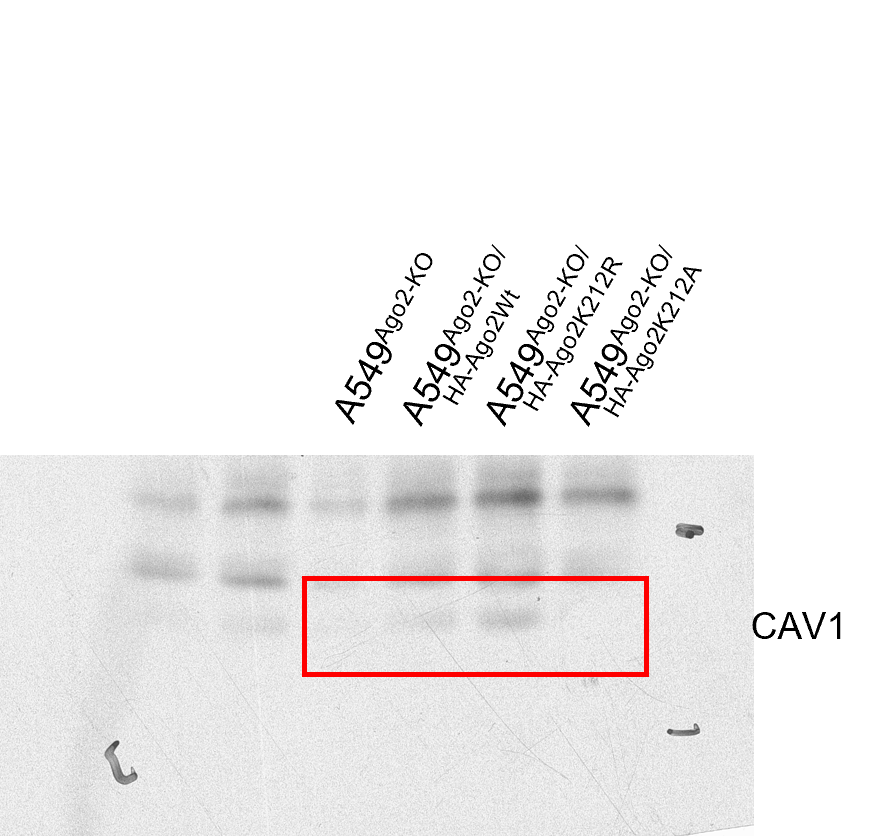

Supplement: Supplementary file 7 — Source data Fig. 6 [file 44319_2024_132_MOESM7_ESM.zip › Figure 6/6B/ii/western IP CAV1.tif]

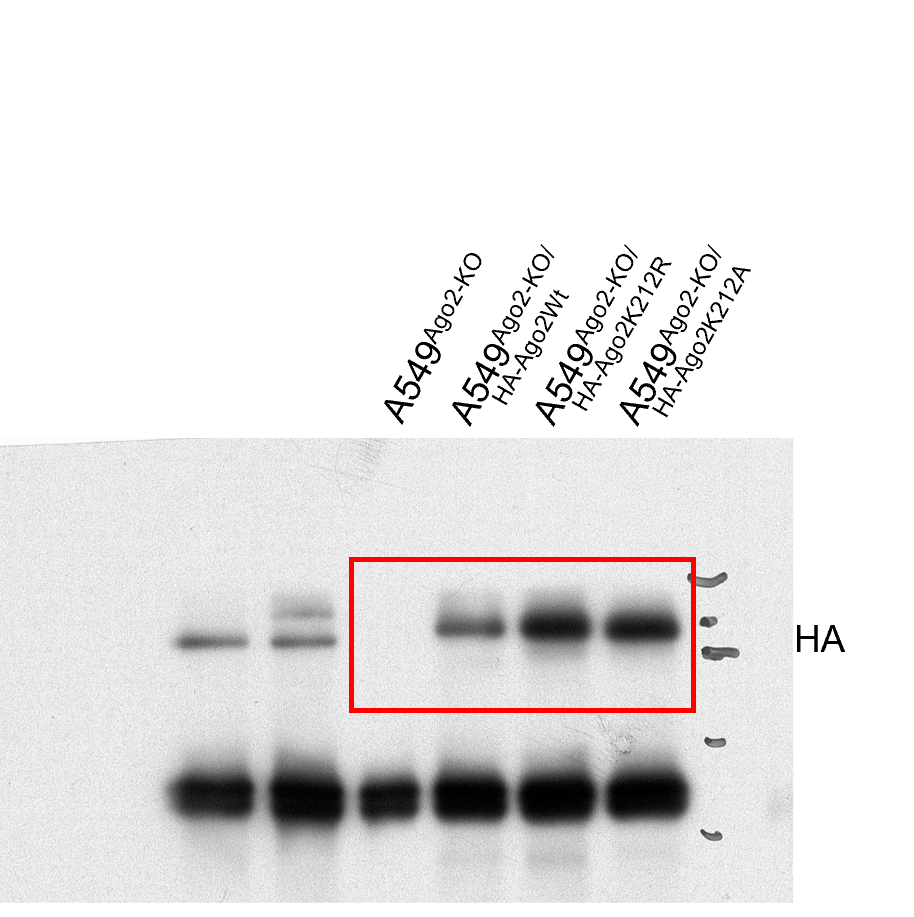

Supplement: Supplementary file 7 — Source data Fig. 6 [file 44319_2024_132_MOESM7_ESM.zip › Figure 6/6B/ii/western IP HA-Ago2.tif]

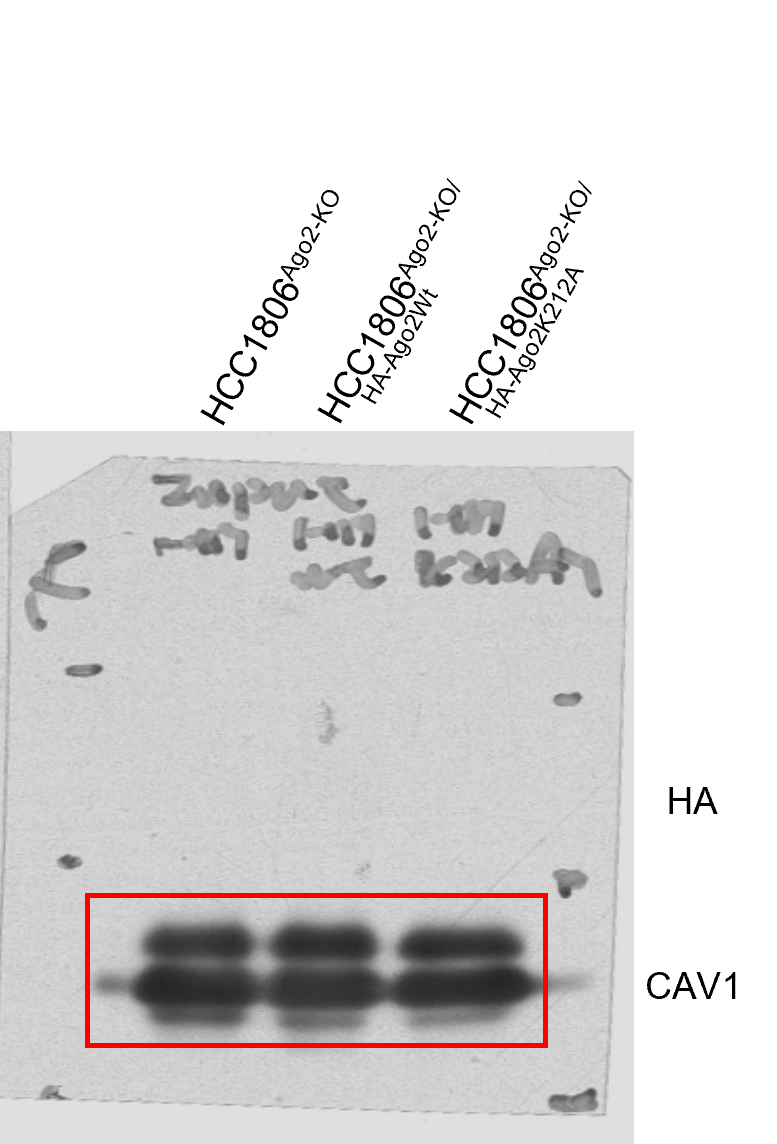

Supplement: Supplementary file 7 — Source data Fig. 6 [file 44319_2024_132_MOESM7_ESM.zip › Figure 6/6B/iii/western input CAV1.tif]

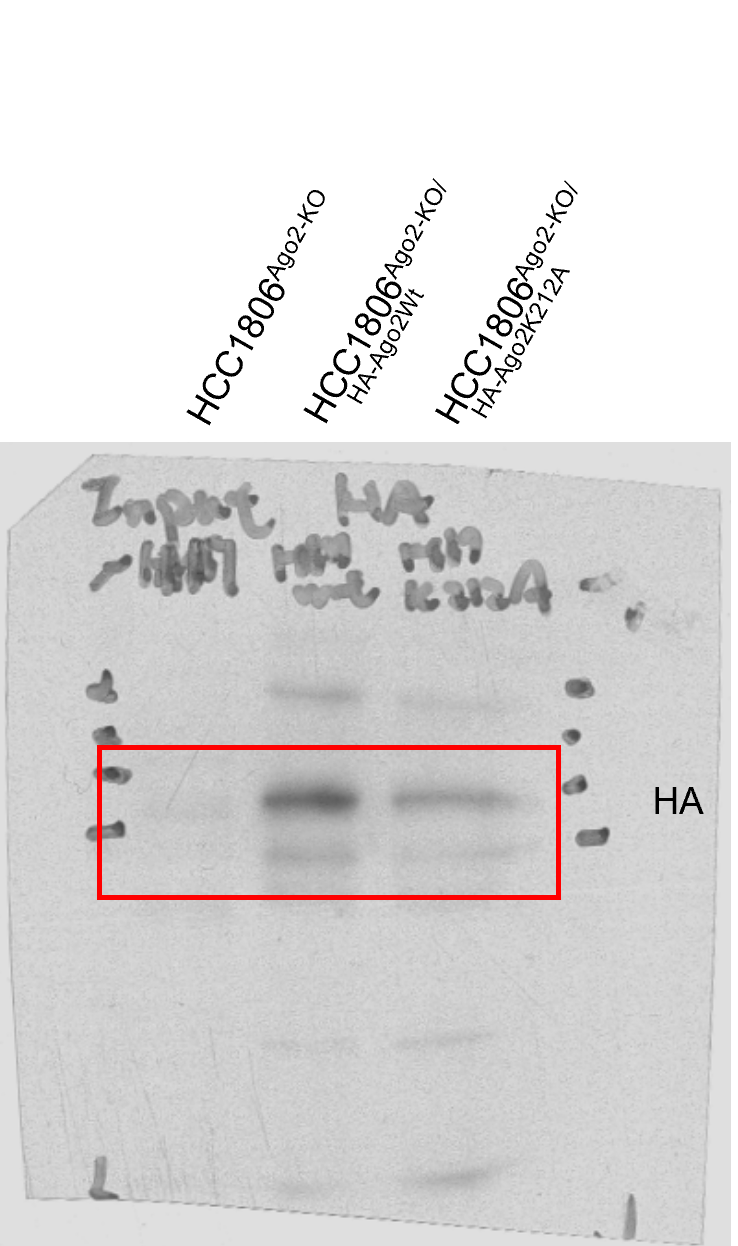

Supplement: Supplementary file 7 — Source data Fig. 6 [file 44319_2024_132_MOESM7_ESM.zip › Figure 6/6B/iii/western input HA-Ago2.tif]

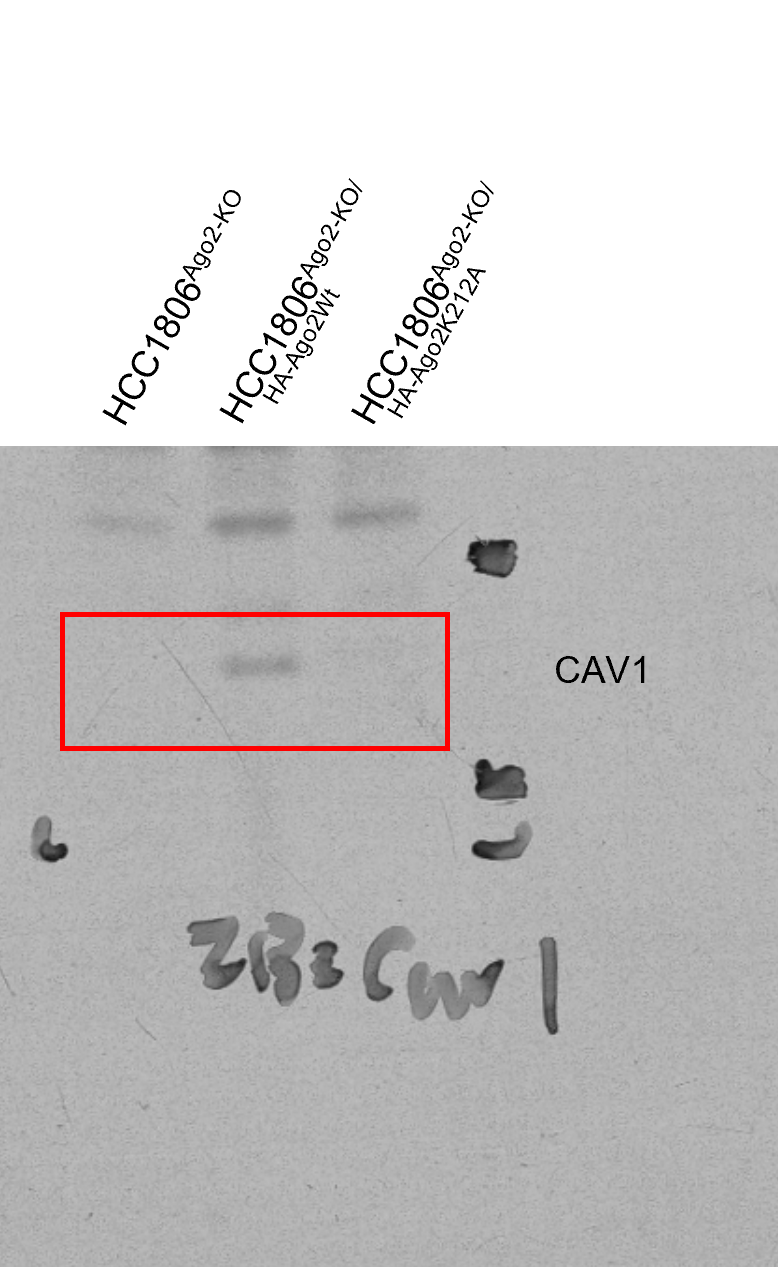

Supplement: Supplementary file 7 — Source data Fig. 6 [file 44319_2024_132_MOESM7_ESM.zip › Figure 6/6B/iii/western IP CAV1.tif]

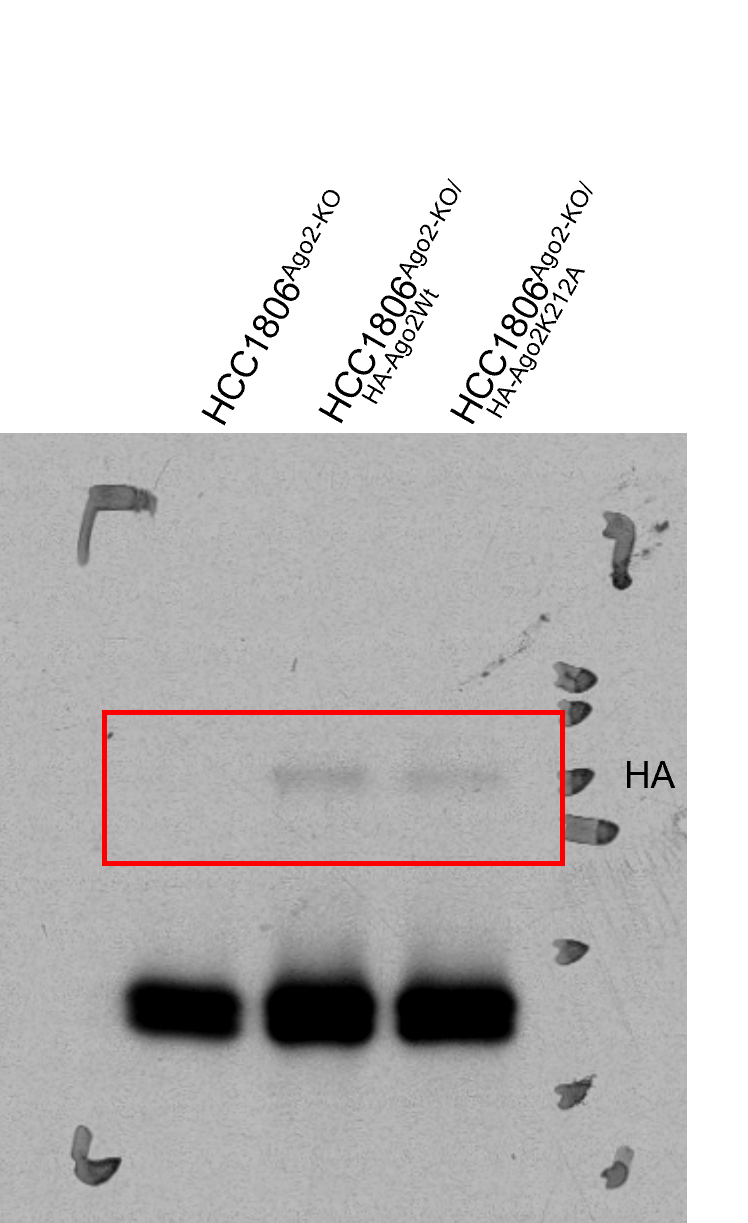

Supplement: Supplementary file 7 — Source data Fig. 6 [file 44319_2024_132_MOESM7_ESM.zip › Figure 6/6B/iii/western IP HA-Ago2.tif]

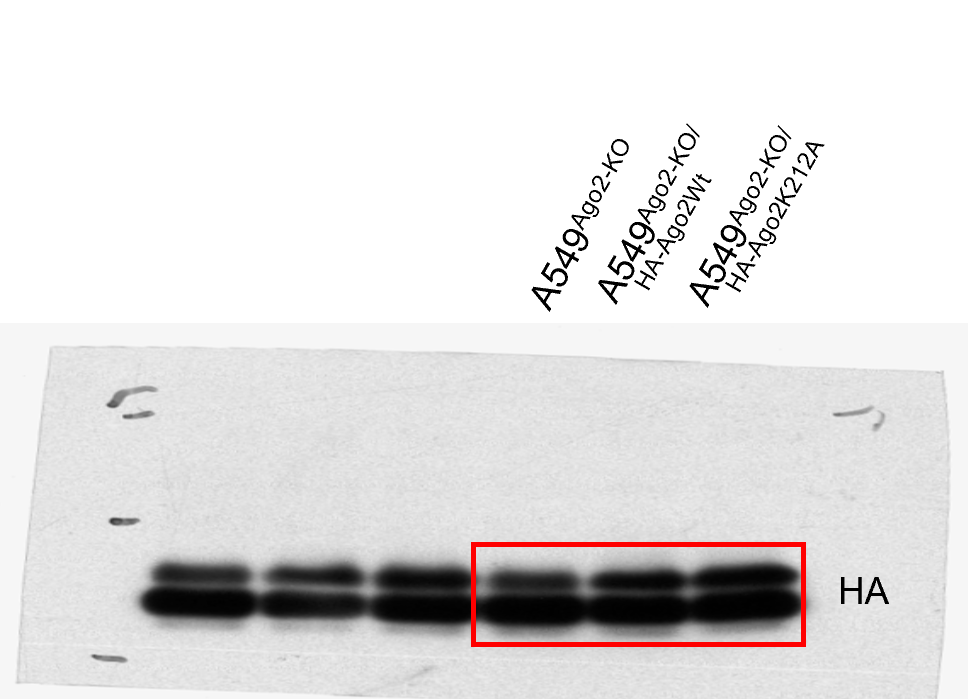

Supplement: Supplementary file 7 — Source data Fig. 6 [file 44319_2024_132_MOESM7_ESM.zip › Figure 6/6C/i/western input CAV1.tif]

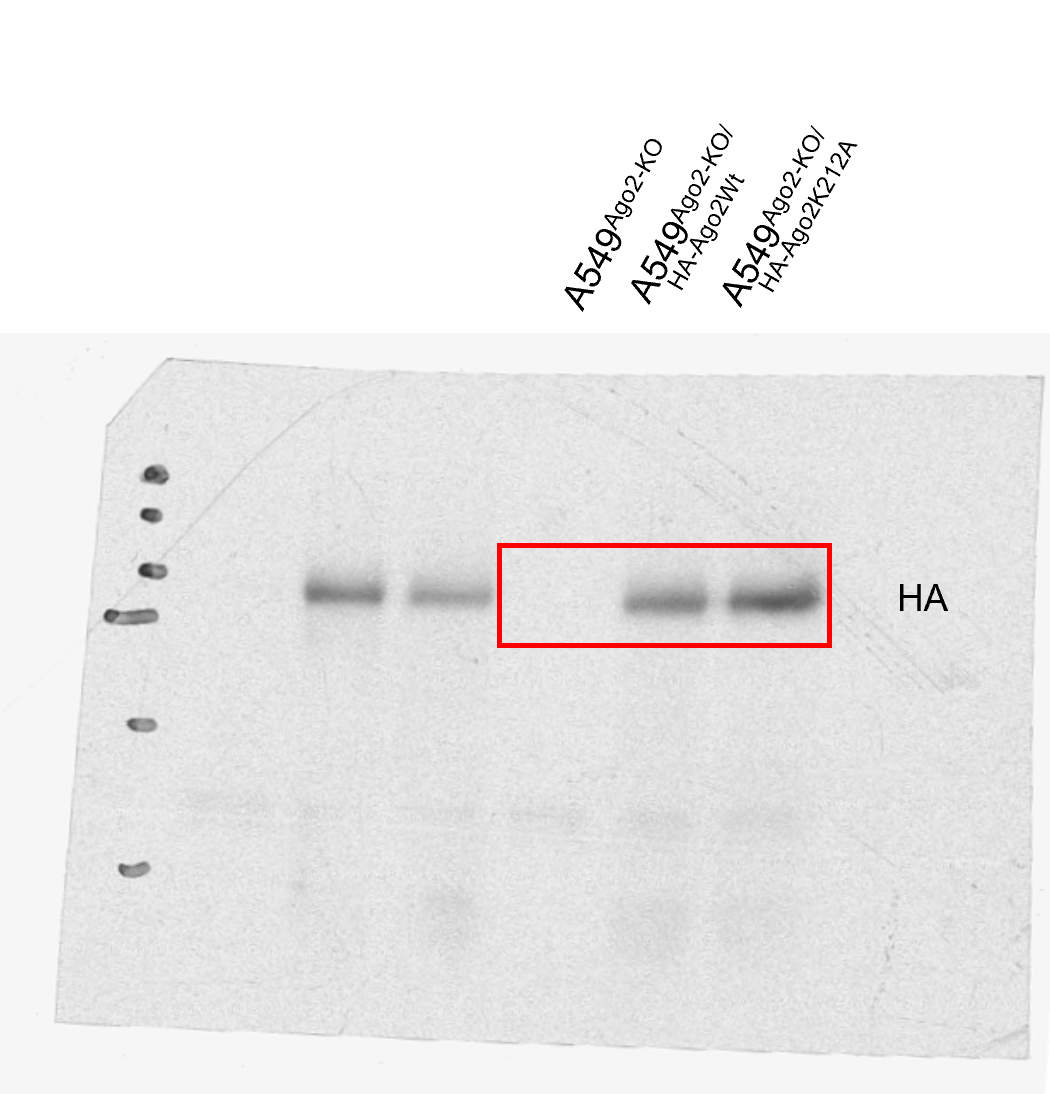

Supplement: Supplementary file 7 — Source data Fig. 6 [file 44319_2024_132_MOESM7_ESM.zip › Figure 6/6C/i/western input HA-Ago2.tif]

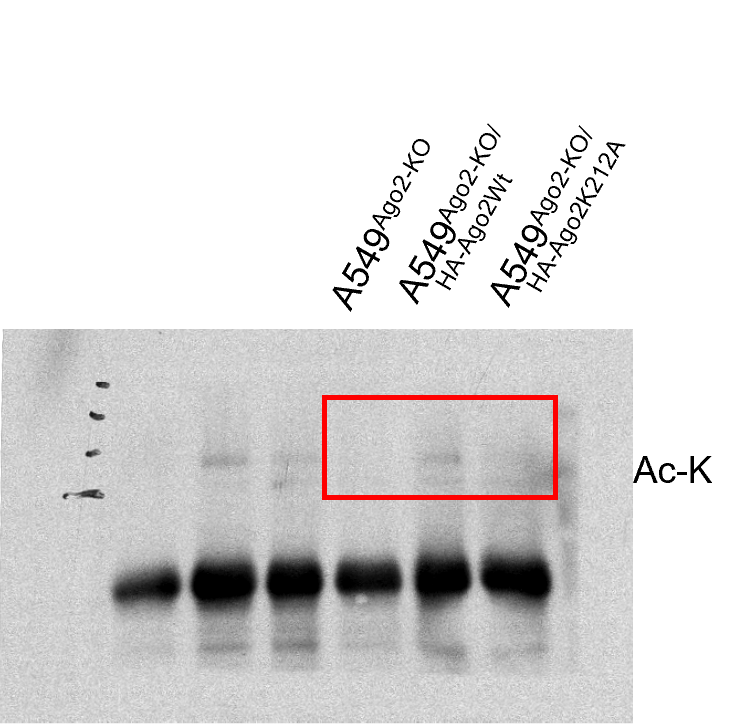

Supplement: Supplementary file 7 — Source data Fig. 6 [file 44319_2024_132_MOESM7_ESM.zip › Figure 6/6C/i/western IP acetylation.tif]

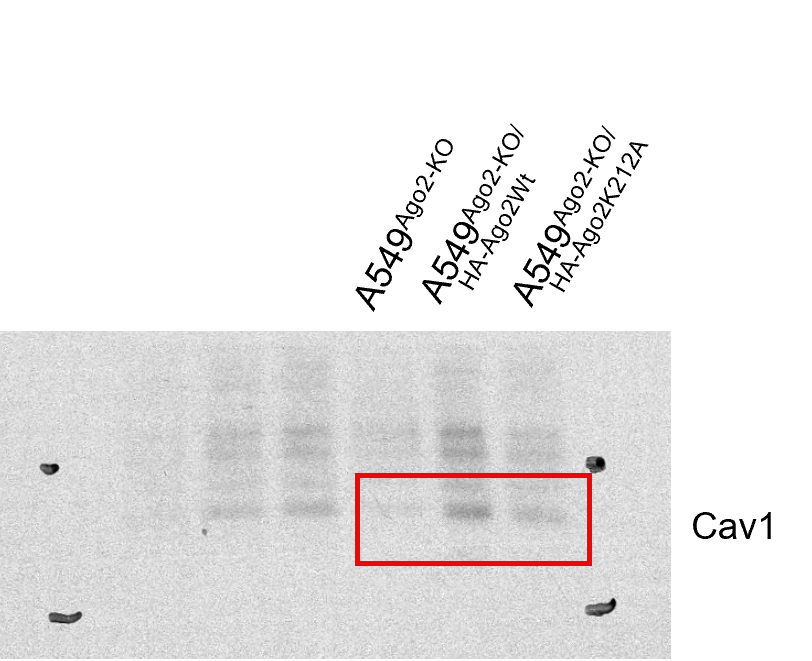

Supplement: Supplementary file 7 — Source data Fig. 6 [file 44319_2024_132_MOESM7_ESM.zip › Figure 6/6C/i/western IP CAV1.tif]

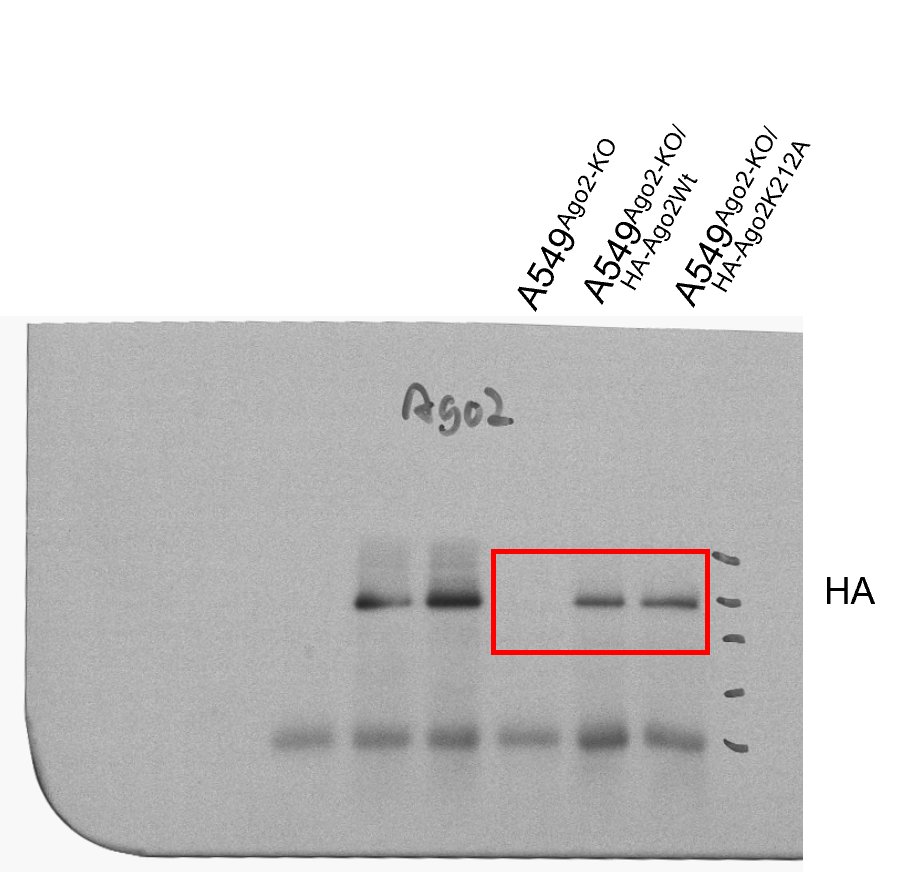

Supplement: Supplementary file 7 — Source data Fig. 6 [file 44319_2024_132_MOESM7_ESM.zip › Figure 6/6C/i/western IP HA-Ago2.tif]

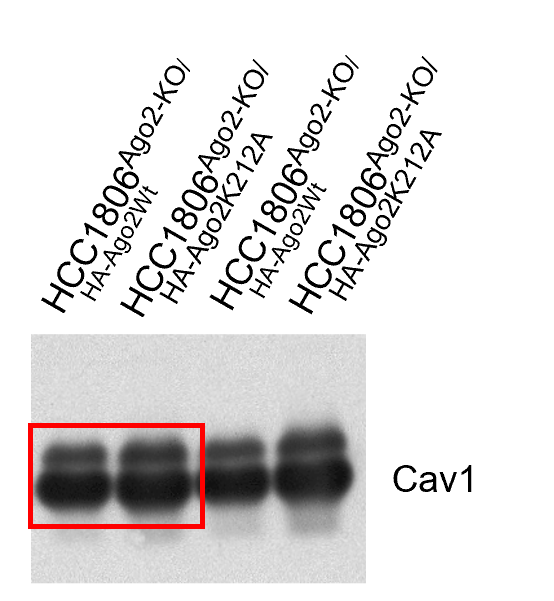

Supplement: Supplementary file 7 — Source data Fig. 6 [file 44319_2024_132_MOESM7_ESM.zip › Figure 6/6C/ii/western input CAV1.tif]

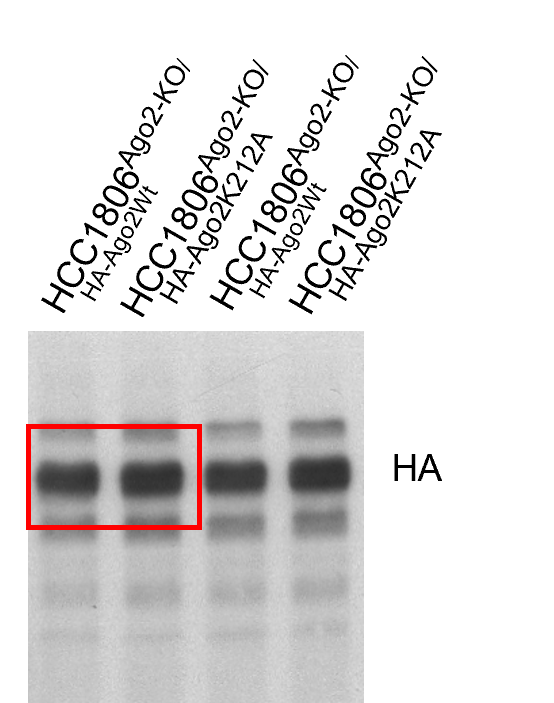

Supplement: Supplementary file 7 — Source data Fig. 6 [file 44319_2024_132_MOESM7_ESM.zip › Figure 6/6C/ii/western input HA-Ago2.tif]

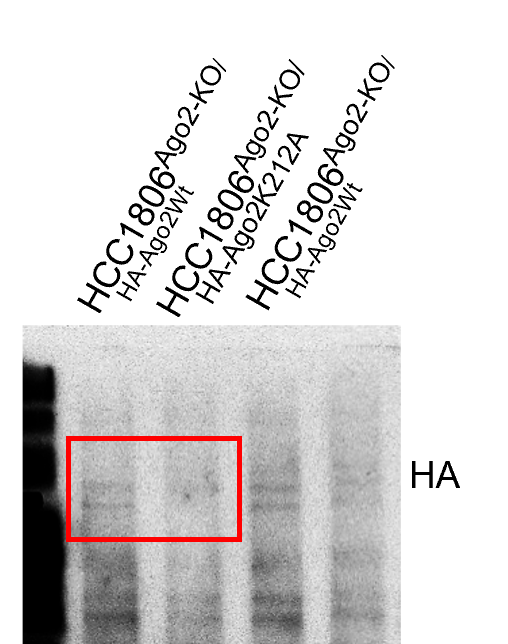

Supplement: Supplementary file 7 — Source data Fig. 6 [file 44319_2024_132_MOESM7_ESM.zip › Figure 6/6C/ii/western IP acetylation.tif]

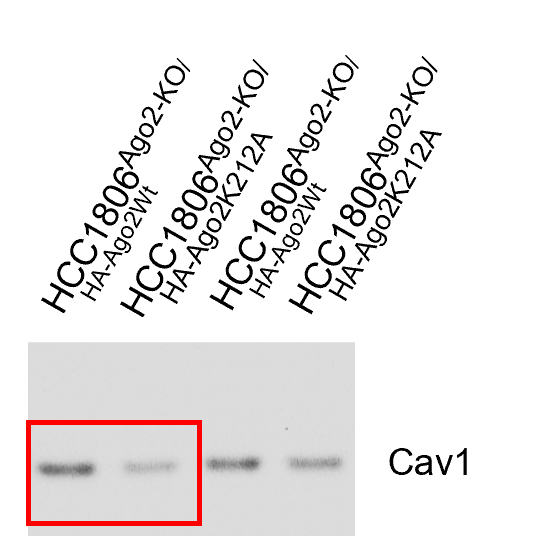

Supplement: Supplementary file 7 — Source data Fig. 6 [file 44319_2024_132_MOESM7_ESM.zip › Figure 6/6C/ii/western IP CAV1.tif]

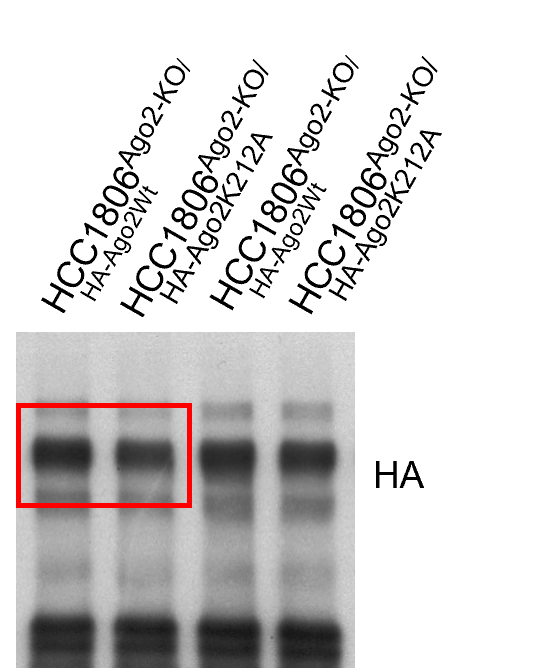

Supplement: Supplementary file 7 — Source data Fig. 6 [file 44319_2024_132_MOESM7_ESM.zip › Figure 6/6C/ii/western IP HA-Ago2.tif]

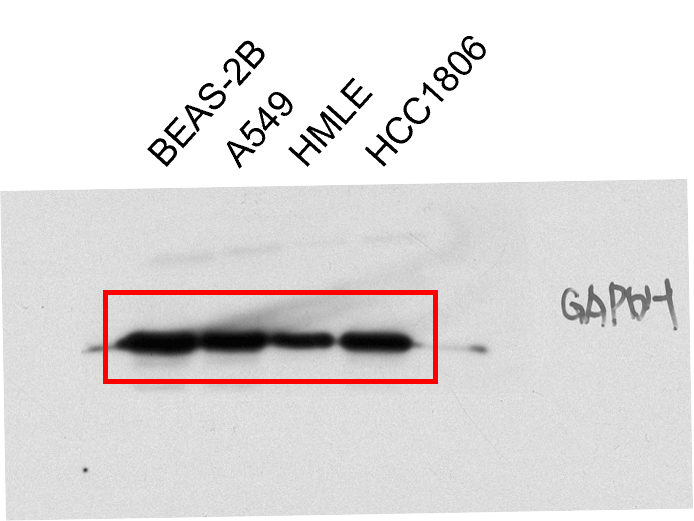

Supplement: Supplementary file 7 — Source data Fig. 6 [file 44319_2024_132_MOESM7_ESM.zip › Figure 6/6D/GAPDH.tif]

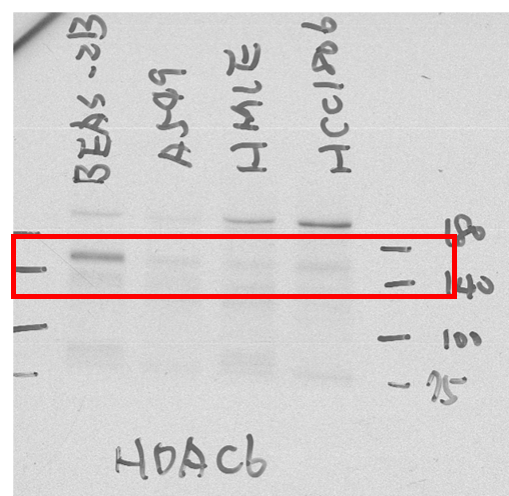

Supplement: Supplementary file 7 — Source data Fig. 6 [file 44319_2024_132_MOESM7_ESM.zip › Figure 6/6D/HDAC6.tif]

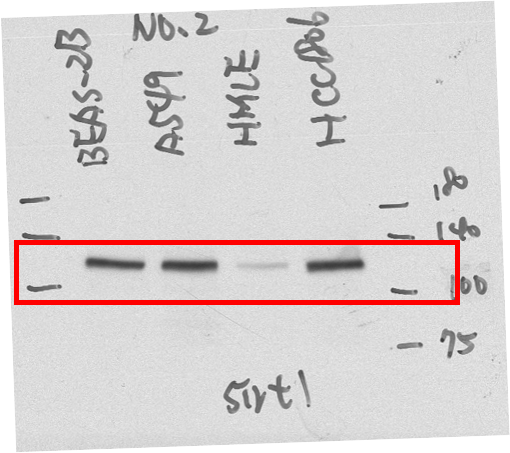

Supplement: Supplementary file 7 — Source data Fig. 6 [file 44319_2024_132_MOESM7_ESM.zip › Figure 6/6D/Sirt1.tif]

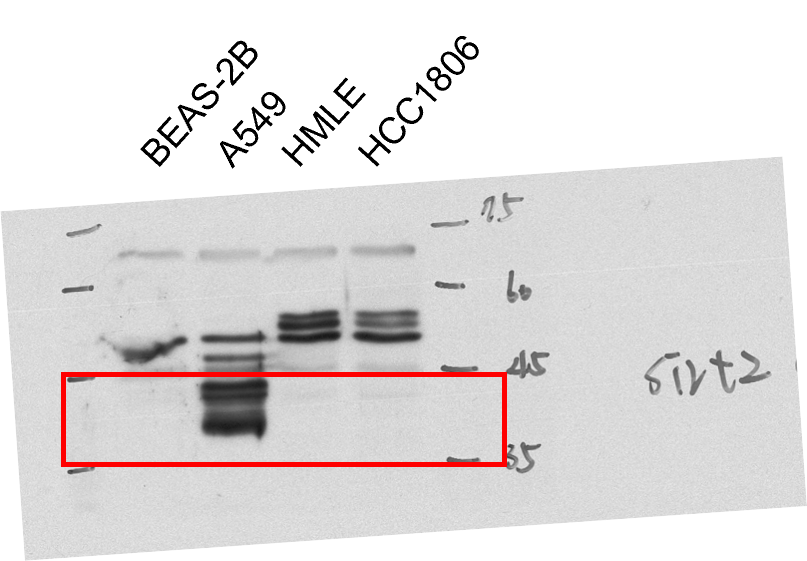

Supplement: Supplementary file 7 — Source data Fig. 6 [file 44319_2024_132_MOESM7_ESM.zip › Figure 6/6D/Sirt2.tif]

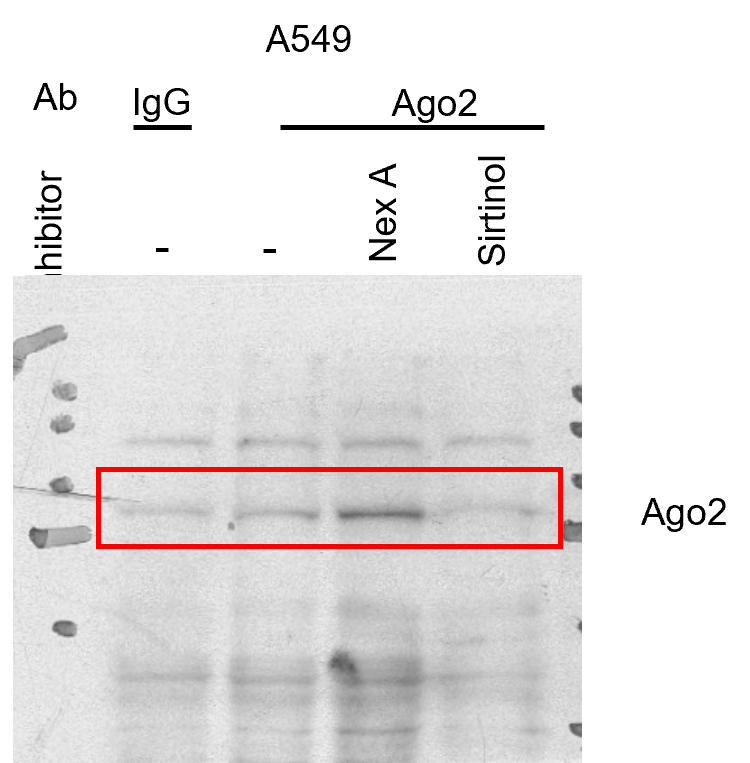

Supplement: Supplementary file 7 — Source data Fig. 6 [file 44319_2024_132_MOESM7_ESM.zip › Figure 6/6E/i/western input Ago2.tif]

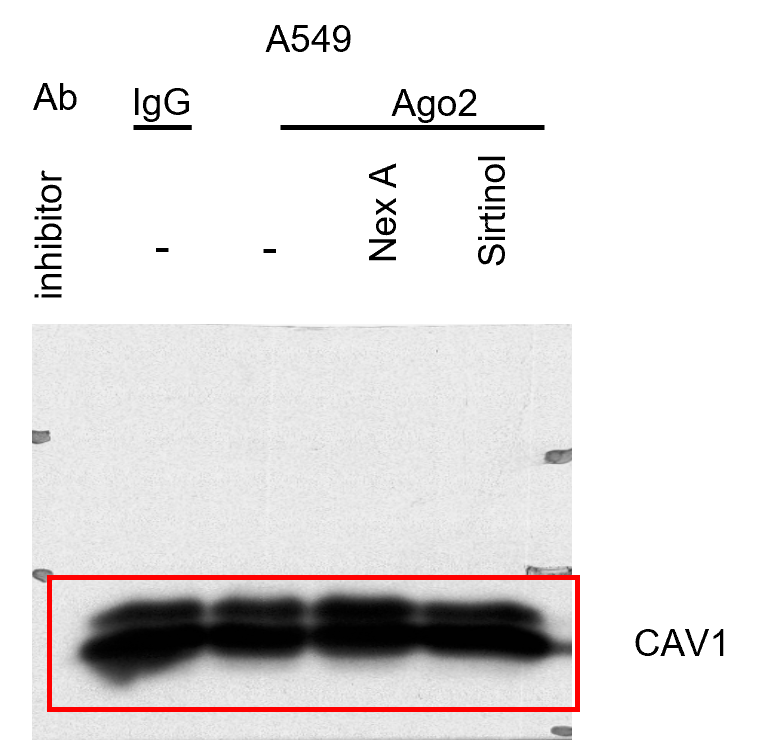

Supplement: Supplementary file 7 — Source data Fig. 6 [file 44319_2024_132_MOESM7_ESM.zip › Figure 6/6E/i/western input CAV1.tif]

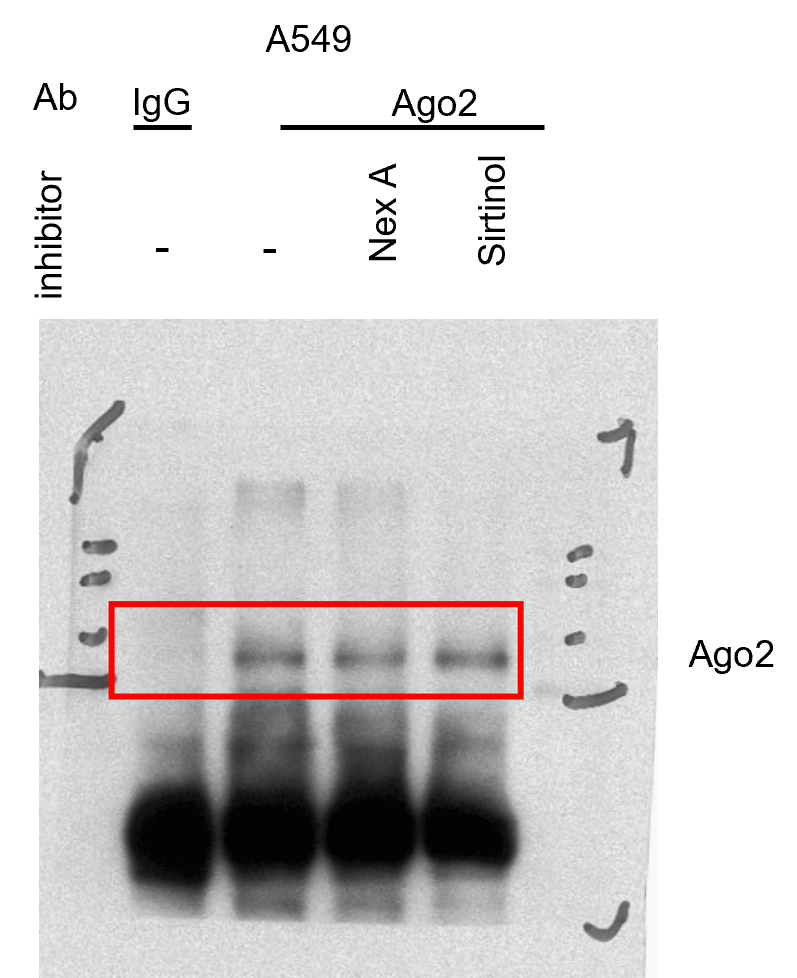

Supplement: Supplementary file 7 — Source data Fig. 6 [file 44319_2024_132_MOESM7_ESM.zip › Figure 6/6E/i/western IP Ago2.tif]

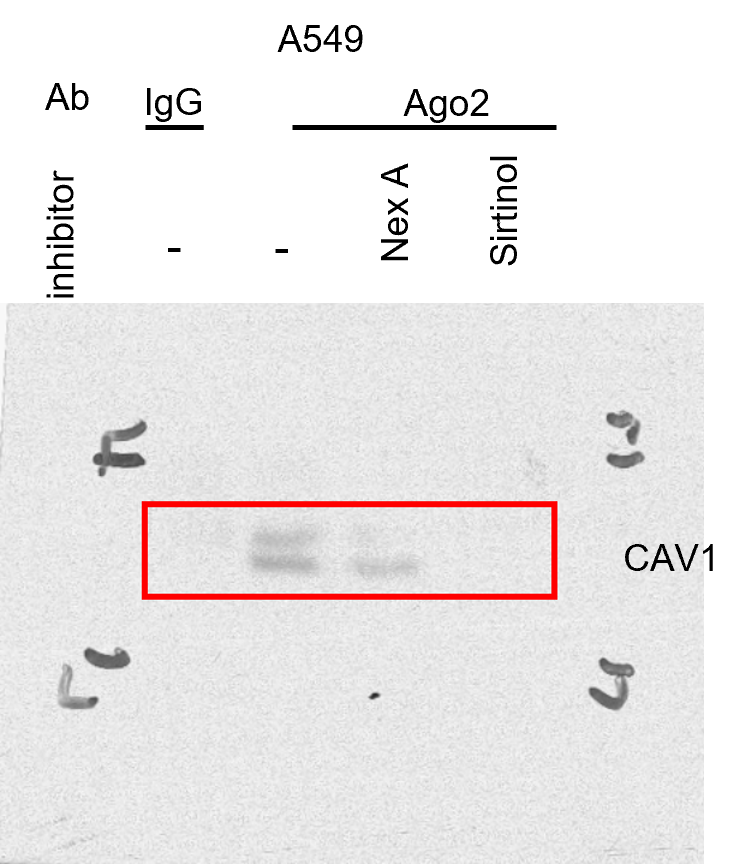

Supplement: Supplementary file 7 — Source data Fig. 6 [file 44319_2024_132_MOESM7_ESM.zip › Figure 6/6E/i/western IP CAV1.tif]

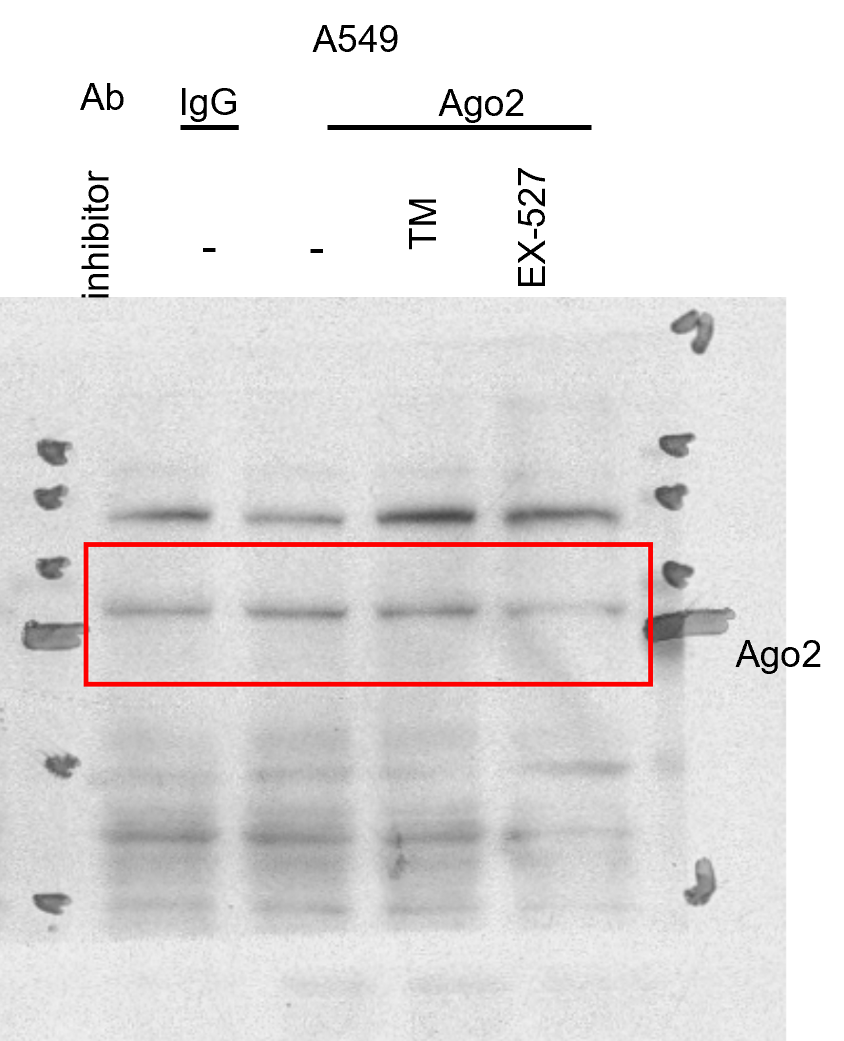

Supplement: Supplementary file 7 — Source data Fig. 6 [file 44319_2024_132_MOESM7_ESM.zip › Figure 6/6E/ii/western input Ago2.tif]

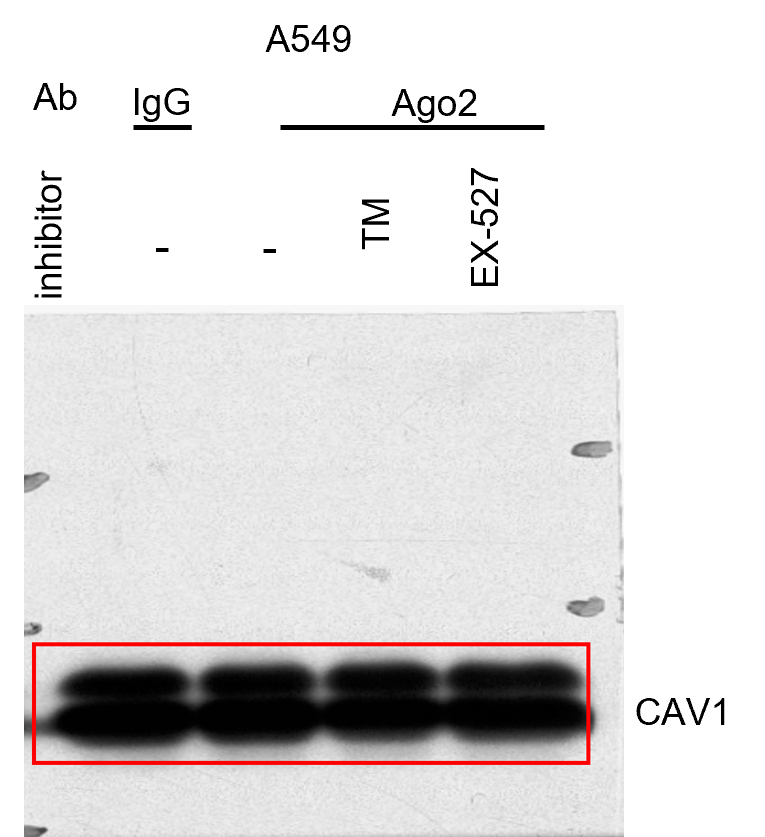

Supplement: Supplementary file 7 — Source data Fig. 6 [file 44319_2024_132_MOESM7_ESM.zip › Figure 6/6E/ii/western input CAV1.tif]

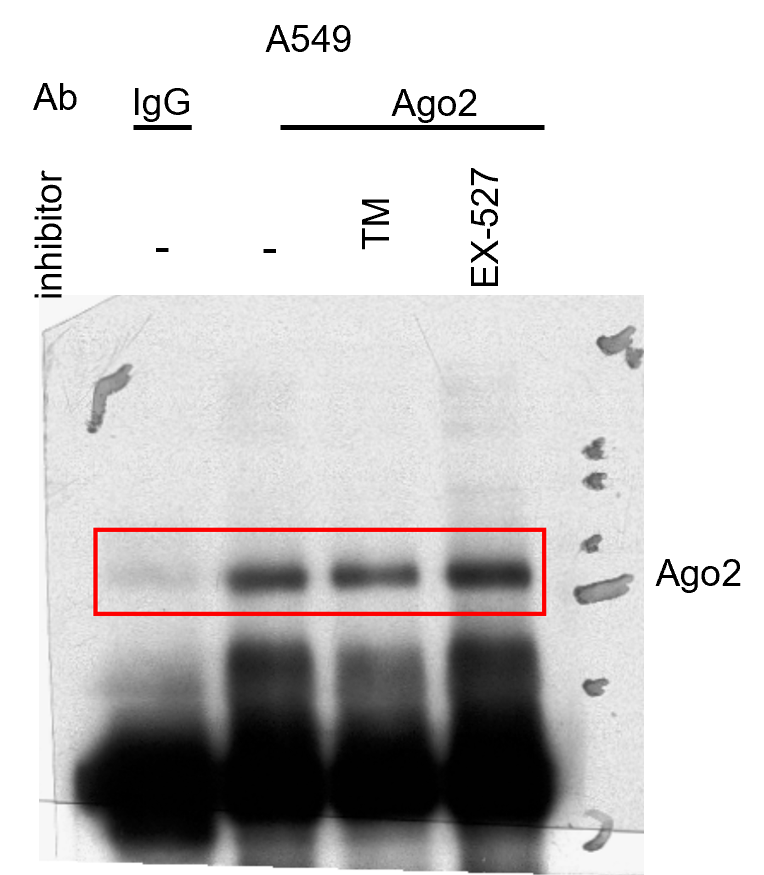

Supplement: Supplementary file 7 — Source data Fig. 6 [file 44319_2024_132_MOESM7_ESM.zip › Figure 6/6E/ii/western IP Ago2.tif]

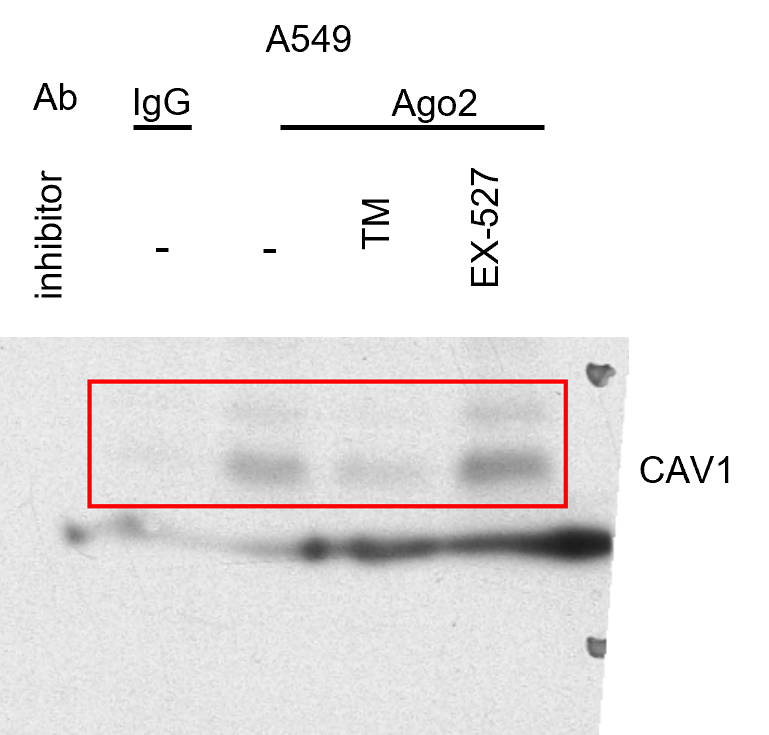

Supplement: Supplementary file 7 — Source data Fig. 6 [file 44319_2024_132_MOESM7_ESM.zip › Figure 6/6E/ii/western IP CAV1.tif]

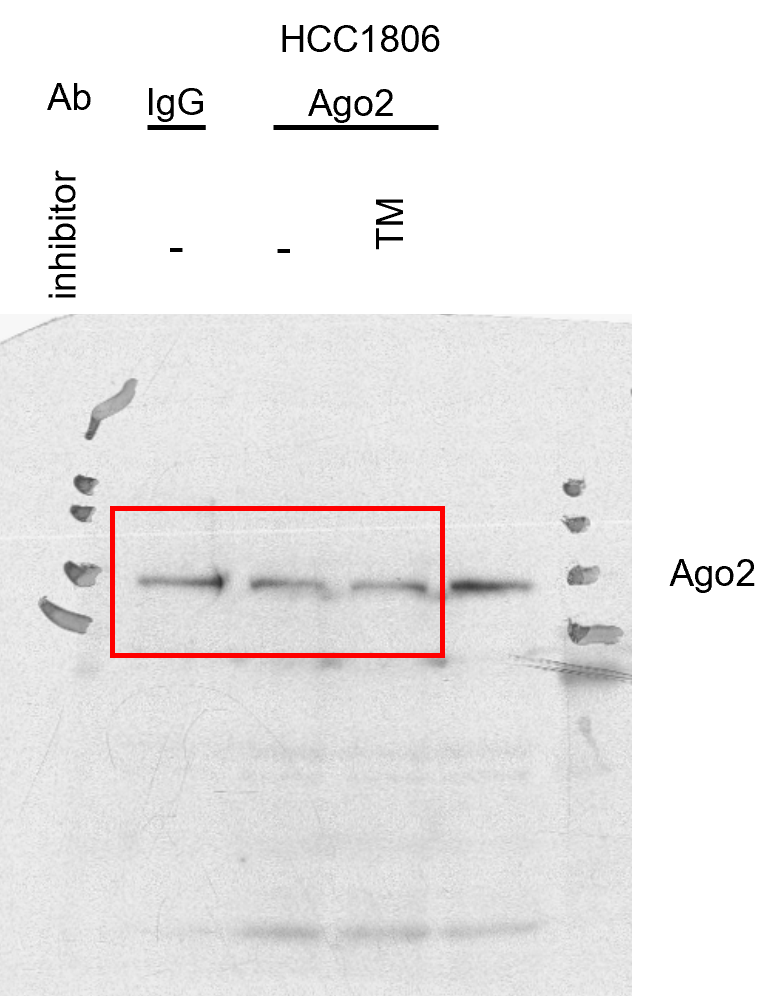

Supplement: Supplementary file 7 — Source data Fig. 6 [file 44319_2024_132_MOESM7_ESM.zip › Figure 6/6E/iii/western input Ago2.tif]

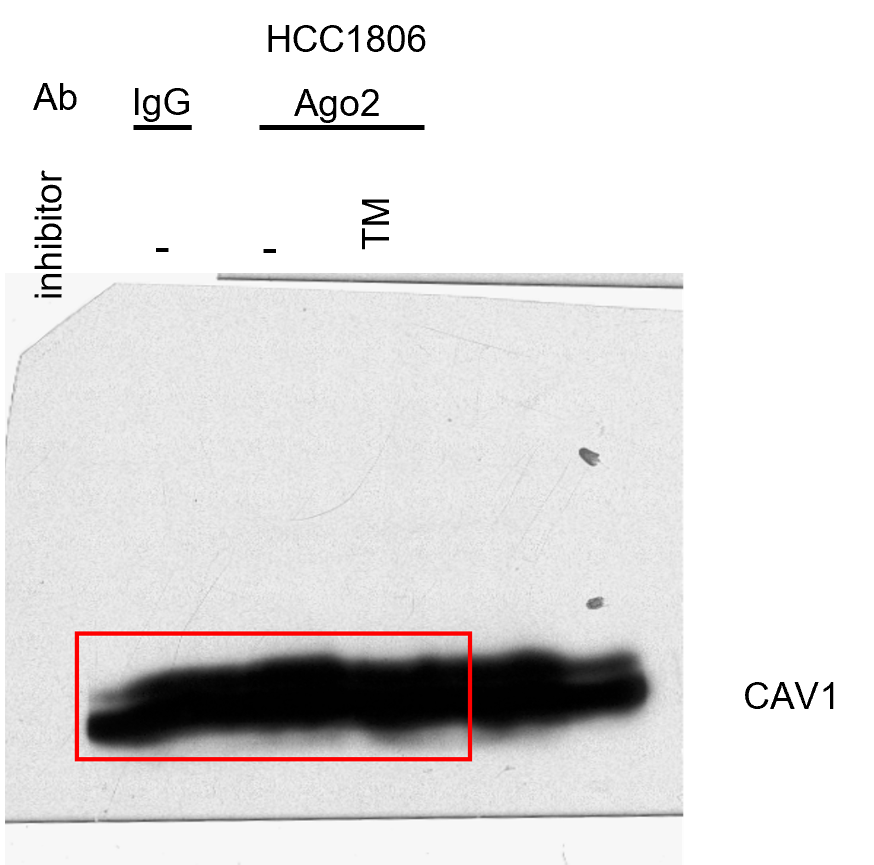

Supplement: Supplementary file 7 — Source data Fig. 6 [file 44319_2024_132_MOESM7_ESM.zip › Figure 6/6E/iii/western input CAV1.tif]

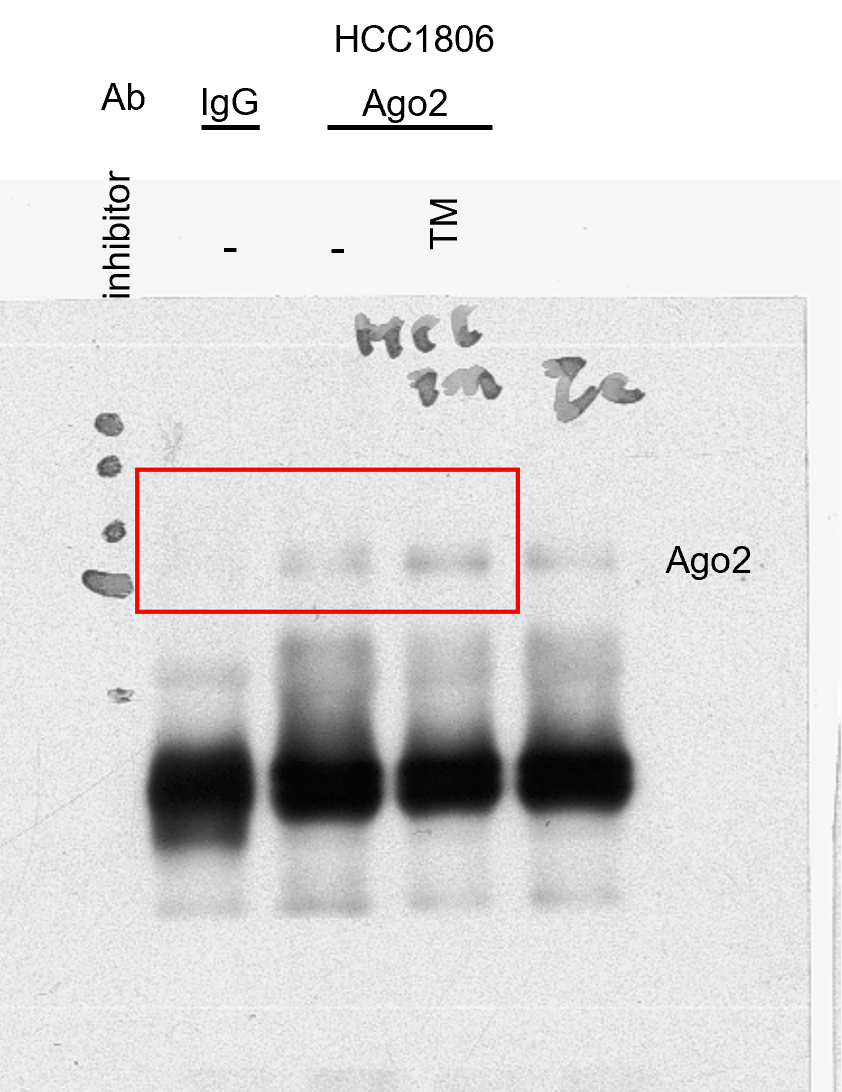

Supplement: Supplementary file 7 — Source data Fig. 6 [file 44319_2024_132_MOESM7_ESM.zip › Figure 6/6E/iii/western IP Ago2.tif]

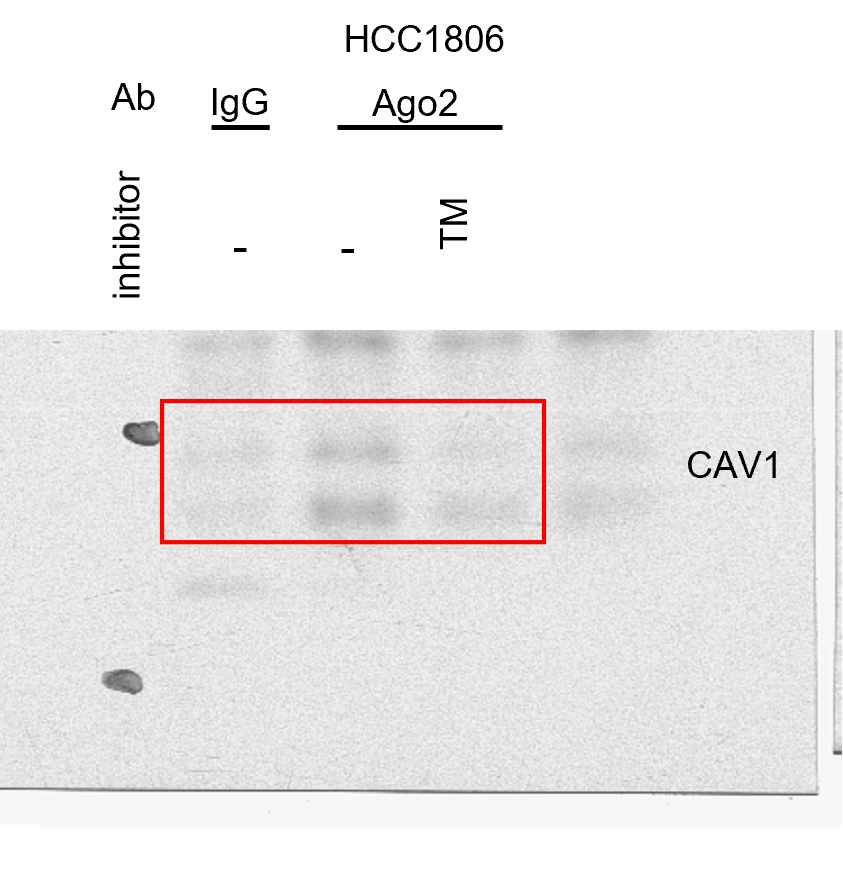

Supplement: Supplementary file 7 — Source data Fig. 6 [file 44319_2024_132_MOESM7_ESM.zip › Figure 6/6E/iii/western IP CAV1.tif]

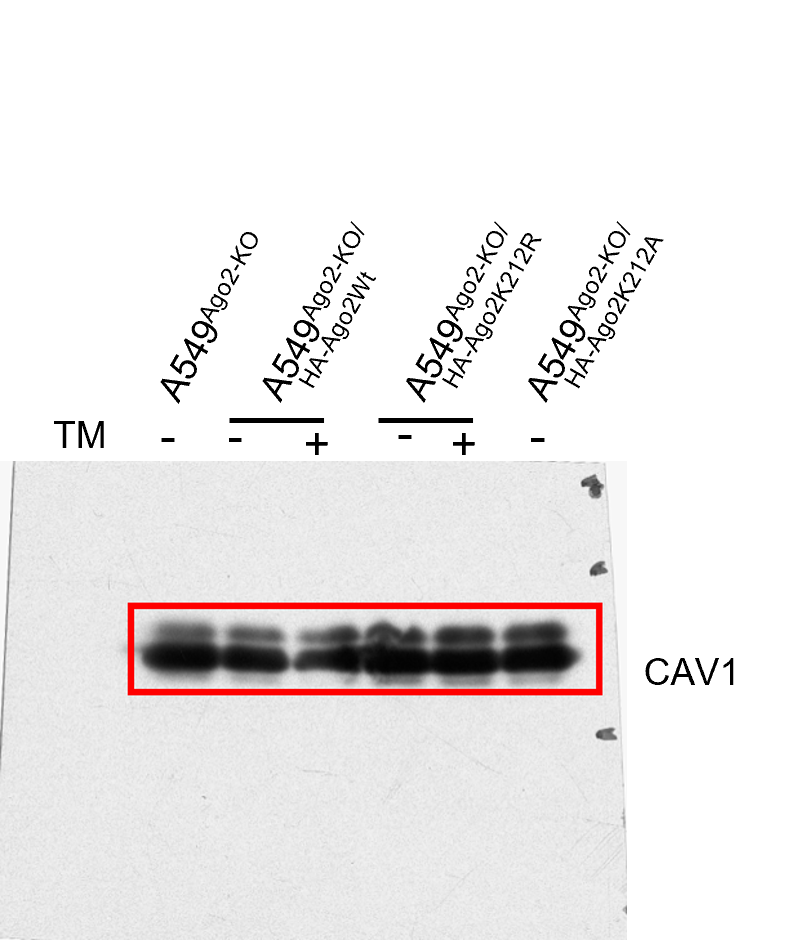

Supplement: Supplementary file 7 — Source data Fig. 6 [file 44319_2024_132_MOESM7_ESM.zip › Figure 6/6F/i/western input CAV1.tif]

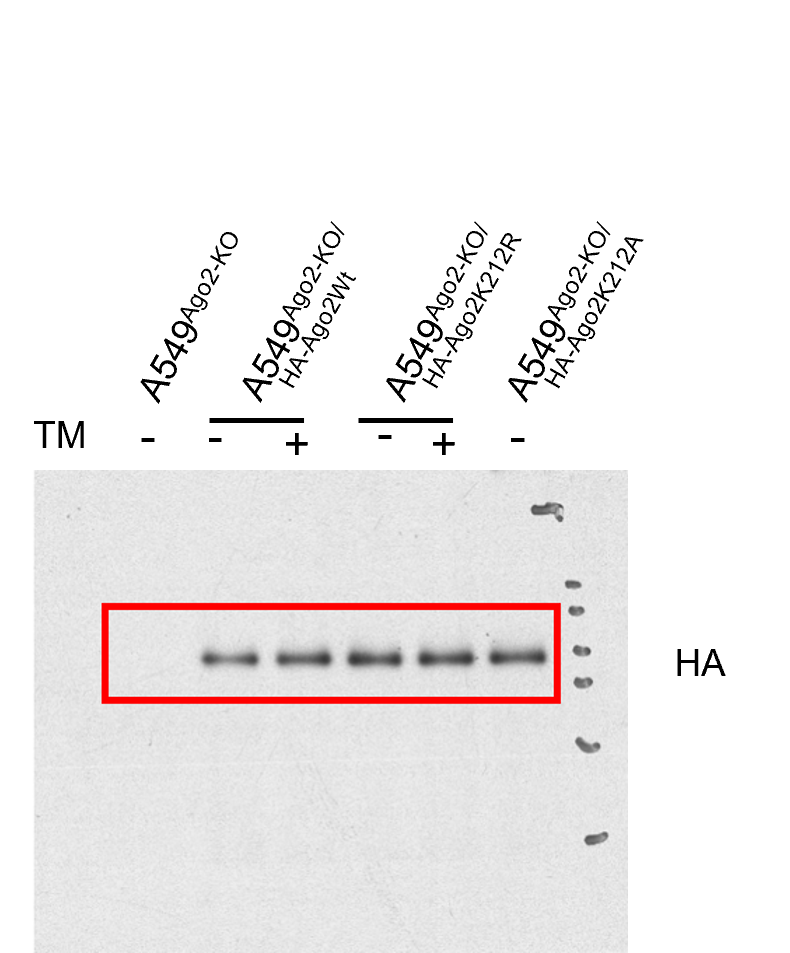

Supplement: Supplementary file 7 — Source data Fig. 6 [file 44319_2024_132_MOESM7_ESM.zip › Figure 6/6F/i/western input HA-Ago2.tif]

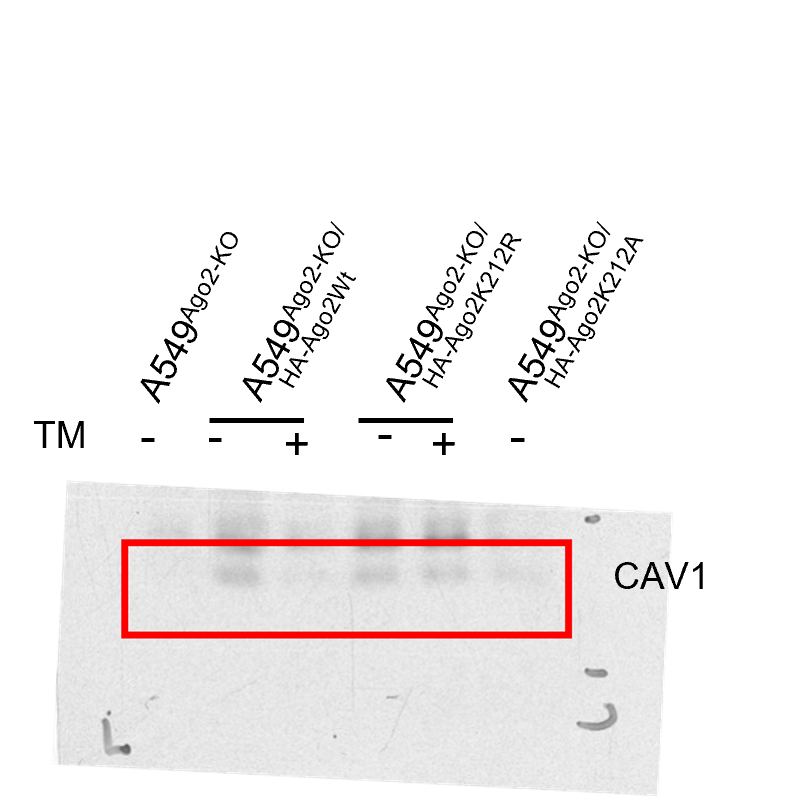

Supplement: Supplementary file 7 — Source data Fig. 6 [file 44319_2024_132_MOESM7_ESM.zip › Figure 6/6F/i/western IP CAV1.tif]

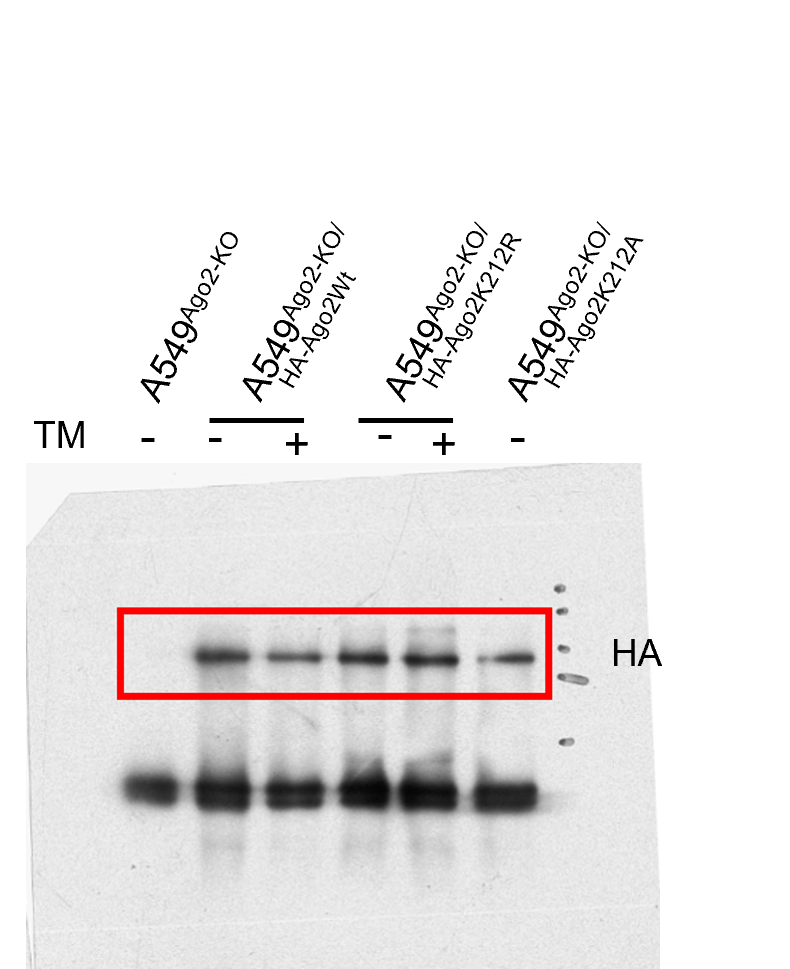

Supplement: Supplementary file 7 — Source data Fig. 6 [file 44319_2024_132_MOESM7_ESM.zip › Figure 6/6F/i/western IP HA-Ago2.tif]

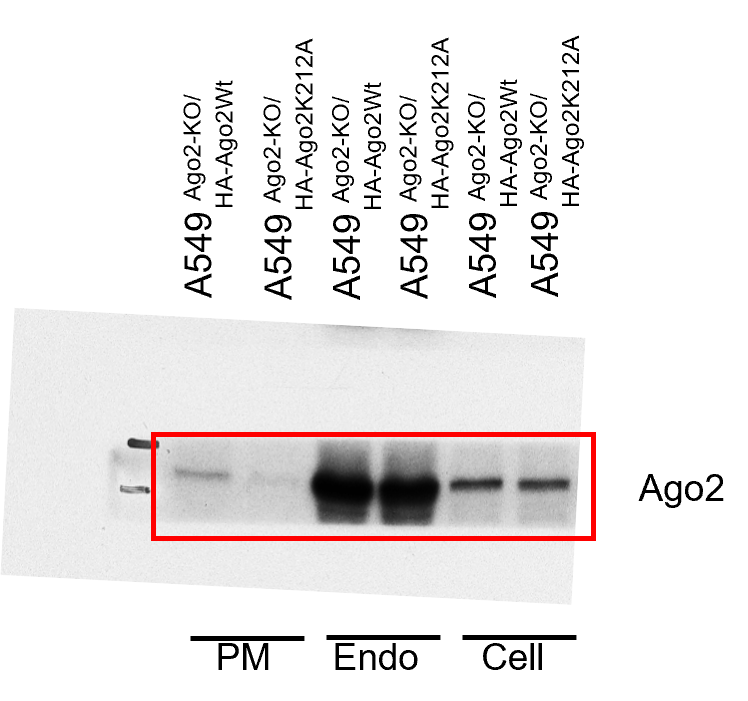

Supplement: Supplementary file 7 — Source data Fig. 6 [file 44319_2024_132_MOESM7_ESM.zip › Figure 6/6G/western Ago2.tif]

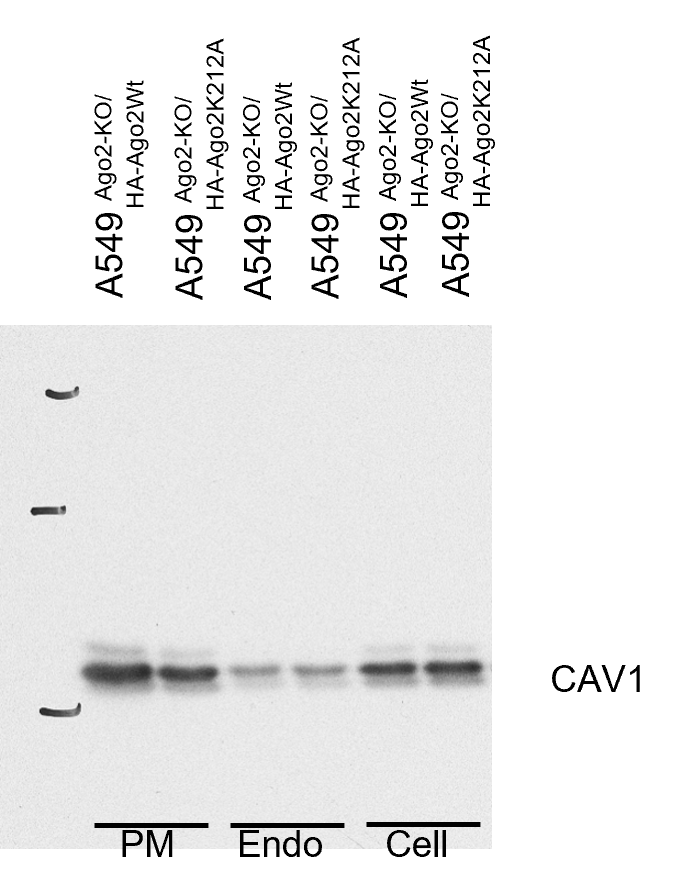

Supplement: Supplementary file 7 — Source data Fig. 6 [file 44319_2024_132_MOESM7_ESM.zip › Figure 6/6G/western CAV1.tif]

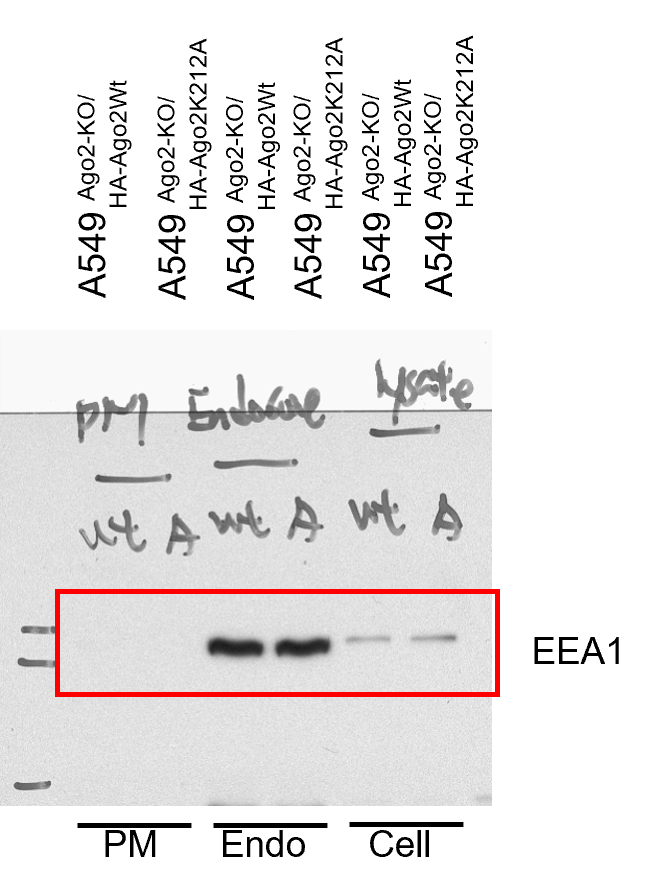

Supplement: Supplementary file 7 — Source data Fig. 6 [file 44319_2024_132_MOESM7_ESM.zip › Figure 6/6G/western EEA1.tif]

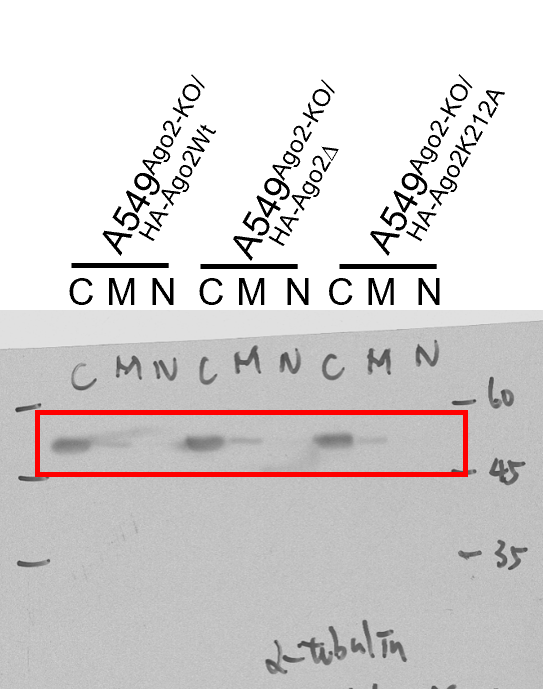

Supplement: Supplementary file 7 — Source data Fig. 6 [file 44319_2024_132_MOESM7_ESM.zip › Figure 6/6J/western alpha-tubulin.tif]

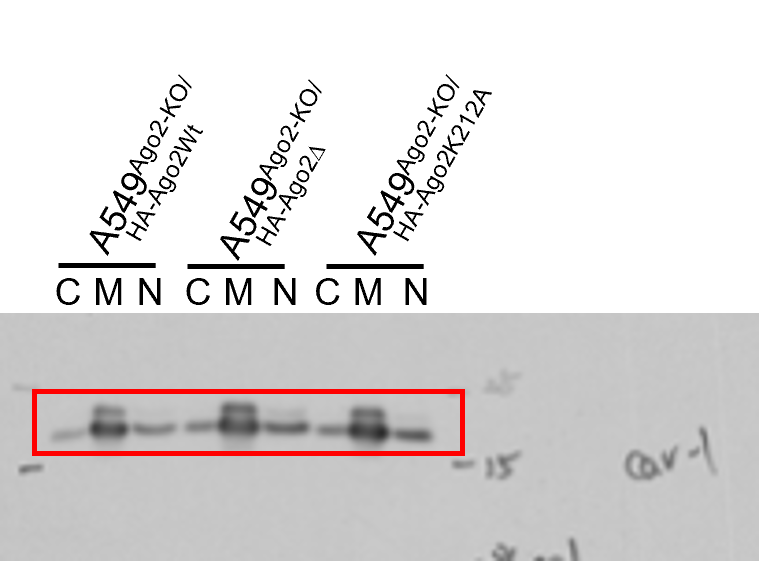

Supplement: Supplementary file 7 — Source data Fig. 6 [file 44319_2024_132_MOESM7_ESM.zip › Figure 6/6J/western CAV1.tif]

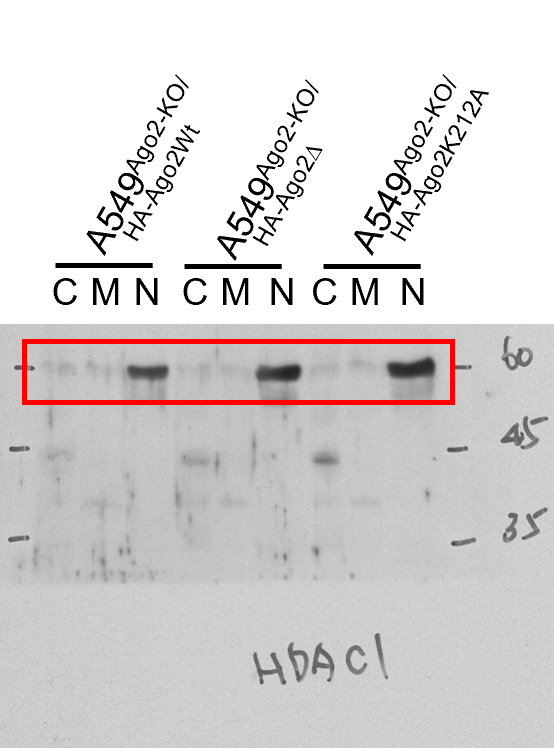

Supplement: Supplementary file 7 — Source data Fig. 6 [file 44319_2024_132_MOESM7_ESM.zip › Figure 6/6J/western HDAC1.tif]

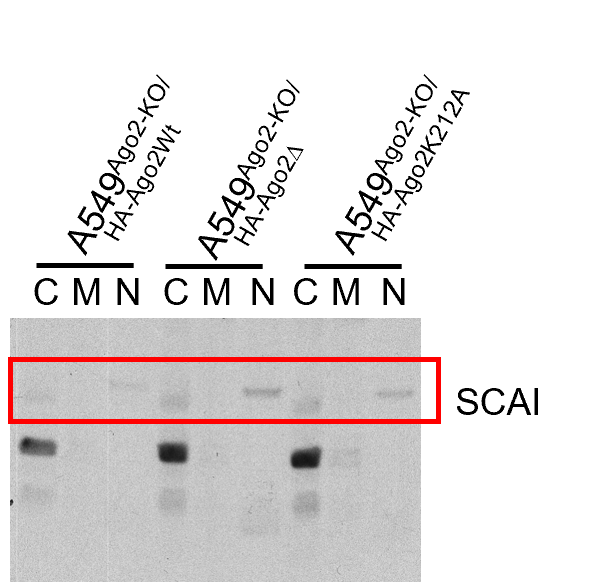

Supplement: Supplementary file 7 — Source data Fig. 6 [file 44319_2024_132_MOESM7_ESM.zip › Figure 6/6J/western SCAI.tif]

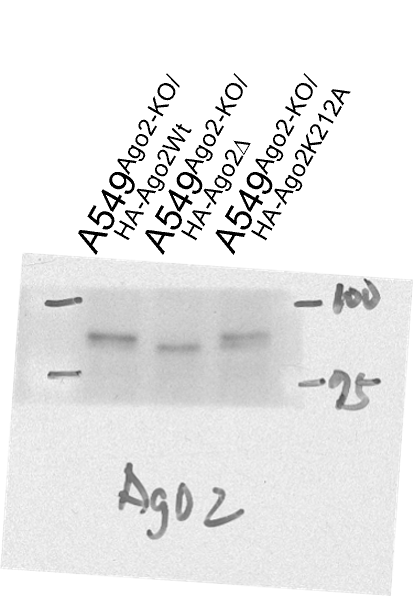

Supplement: Supplementary file 7 — Source data Fig. 6 [file 44319_2024_132_MOESM7_ESM.zip › Figure 6/6K/western Ago2.tif]

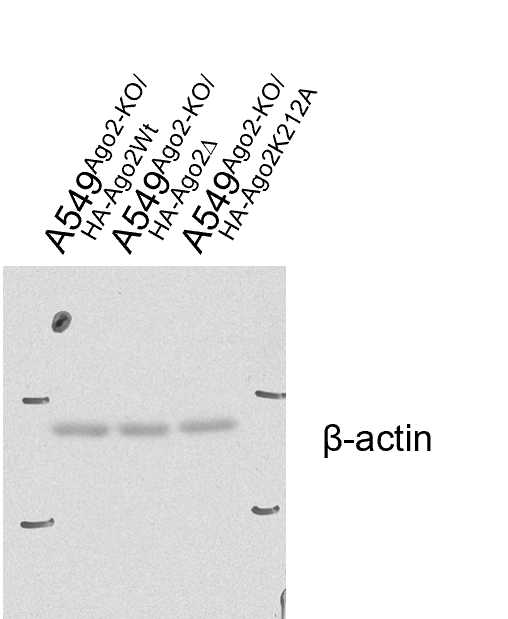

Supplement: Supplementary file 7 — Source data Fig. 6 [file 44319_2024_132_MOESM7_ESM.zip › Figure 6/6K/western beta-actin.tif]

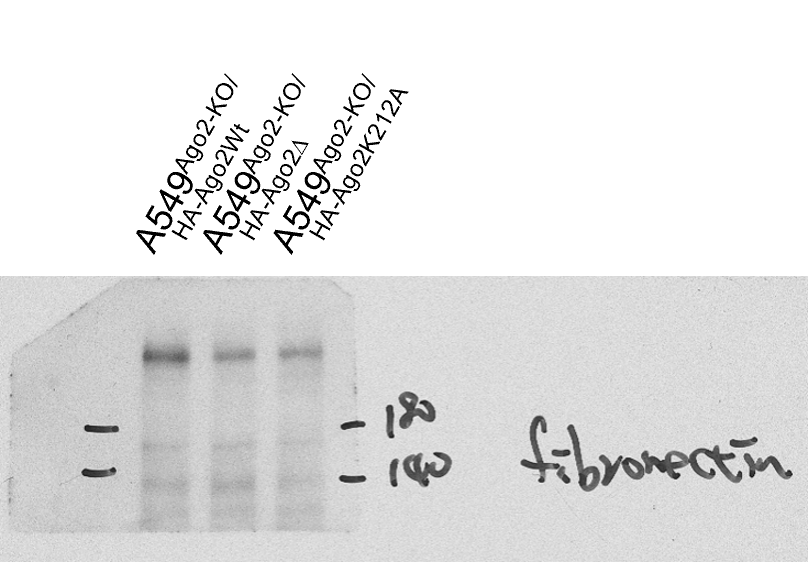

Supplement: Supplementary file 7 — Source data Fig. 6 [file 44319_2024_132_MOESM7_ESM.zip › Figure 6/6K/western fibronectin.tif]

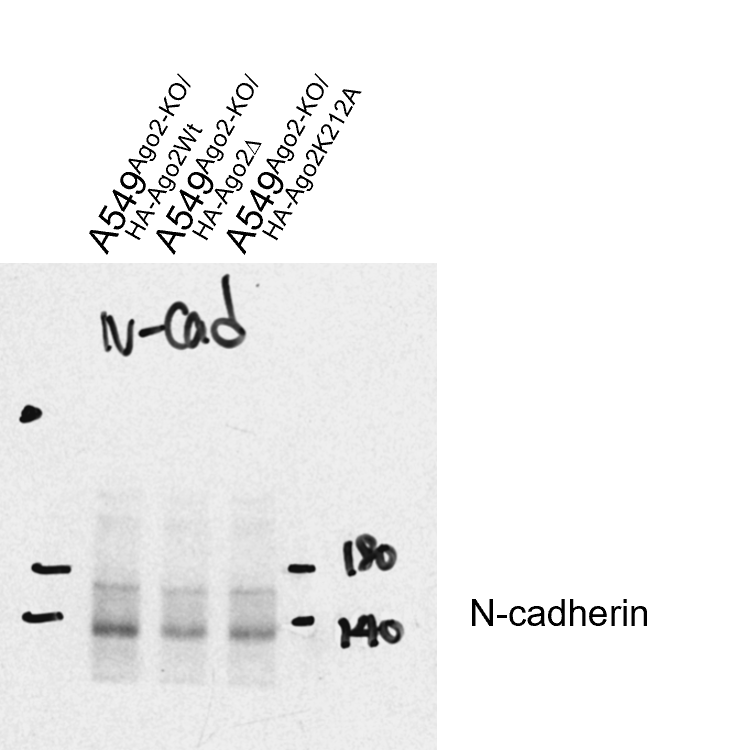

Supplement: Supplementary file 7 — Source data Fig. 6 [file 44319_2024_132_MOESM7_ESM.zip › Figure 6/6K/western N-cadherin.tif]

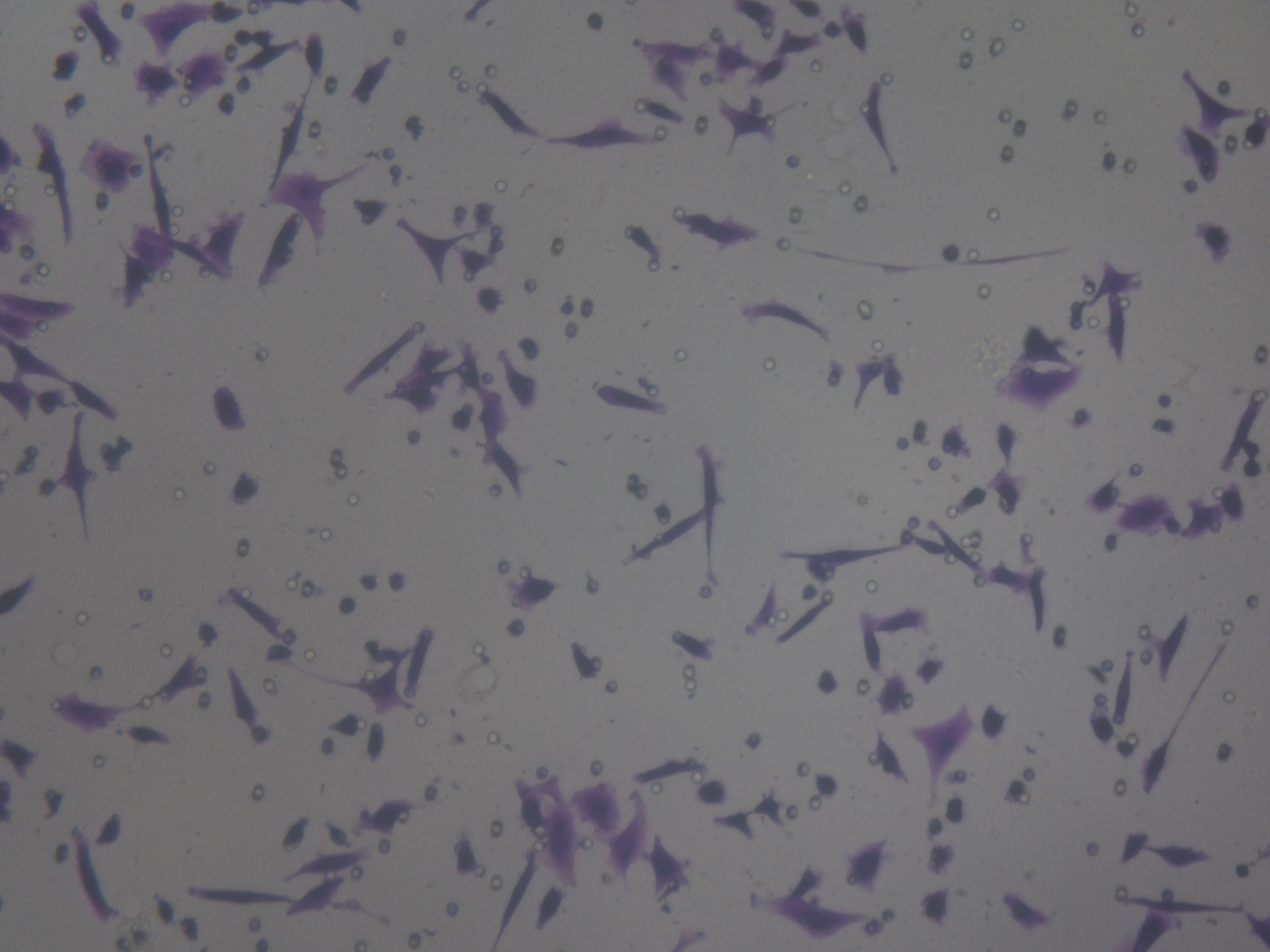

Supplement: Supplementary file 8 — Source data Fig. 7 [file 44319_2024_132_MOESM8_ESM.zip › Figure 7/7A/migration A549-HA-Ago2212A.tif]

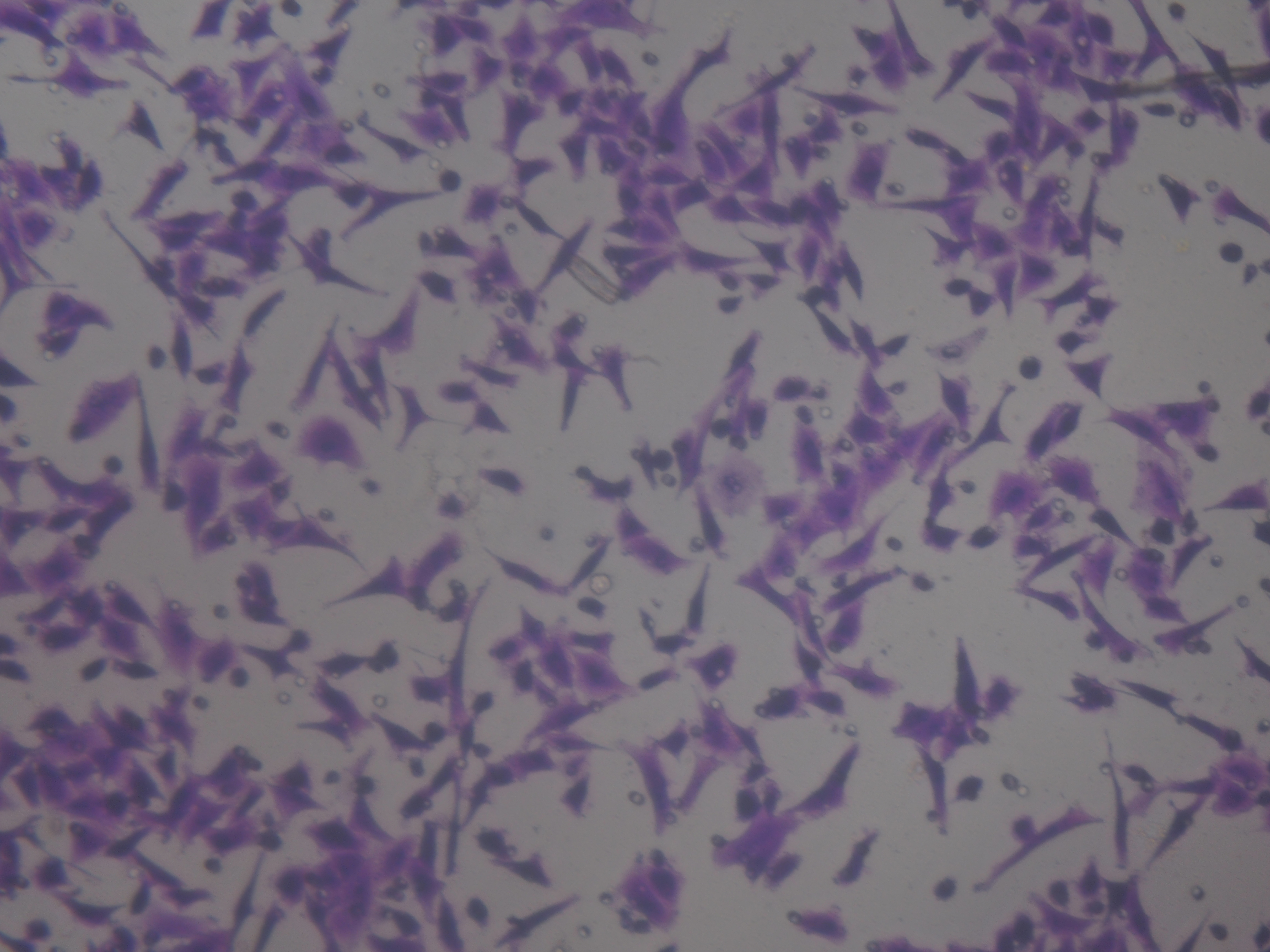

Supplement: Supplementary file 8 — Source data Fig. 7 [file 44319_2024_132_MOESM8_ESM.zip › Figure 7/7A/migration A549-HA-Ago2212R.tif]

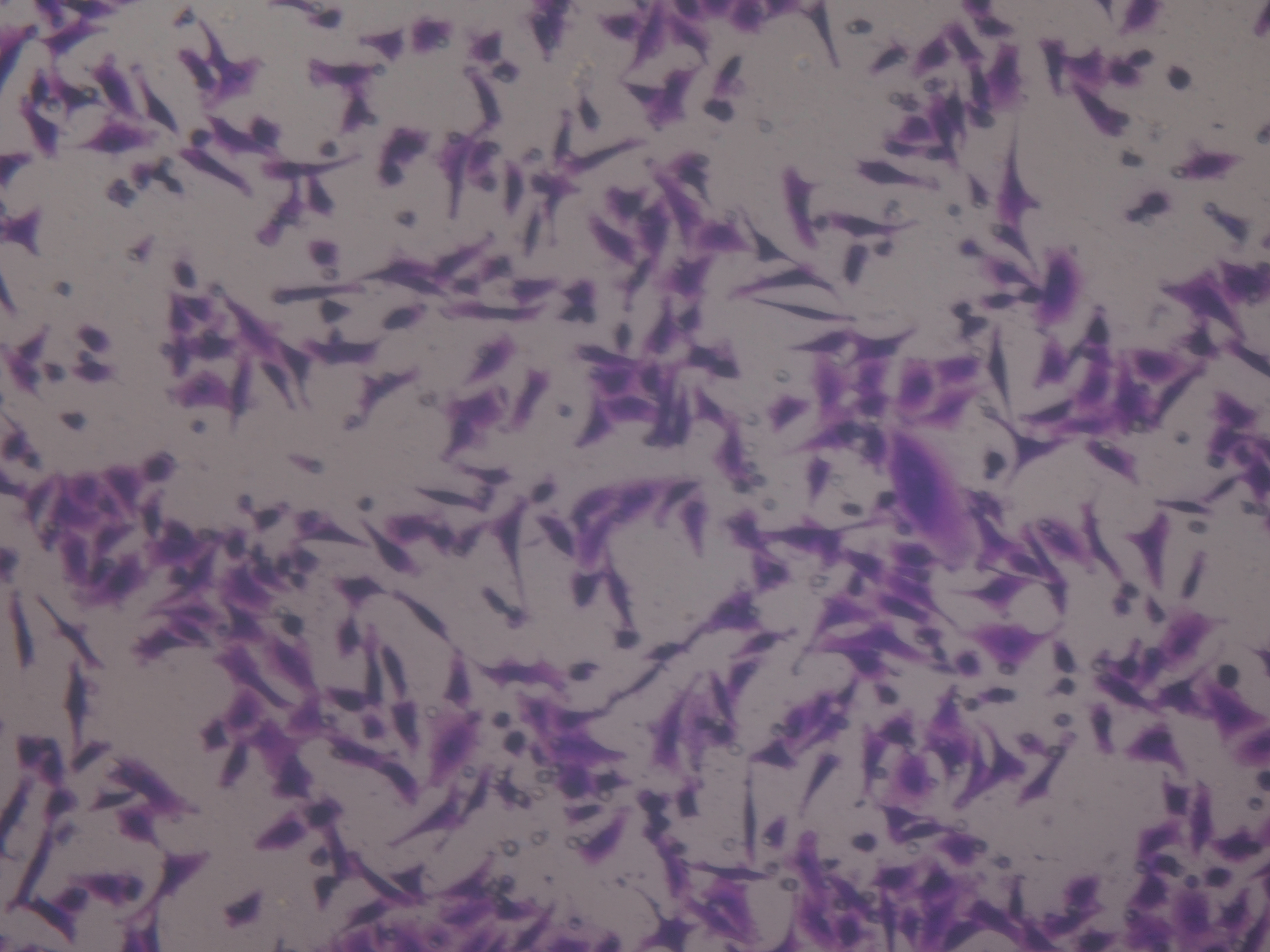

Supplement: Supplementary file 8 — Source data Fig. 7 [file 44319_2024_132_MOESM8_ESM.zip › Figure 7/7A/migration A549-HA-Ago2Wt.tif]

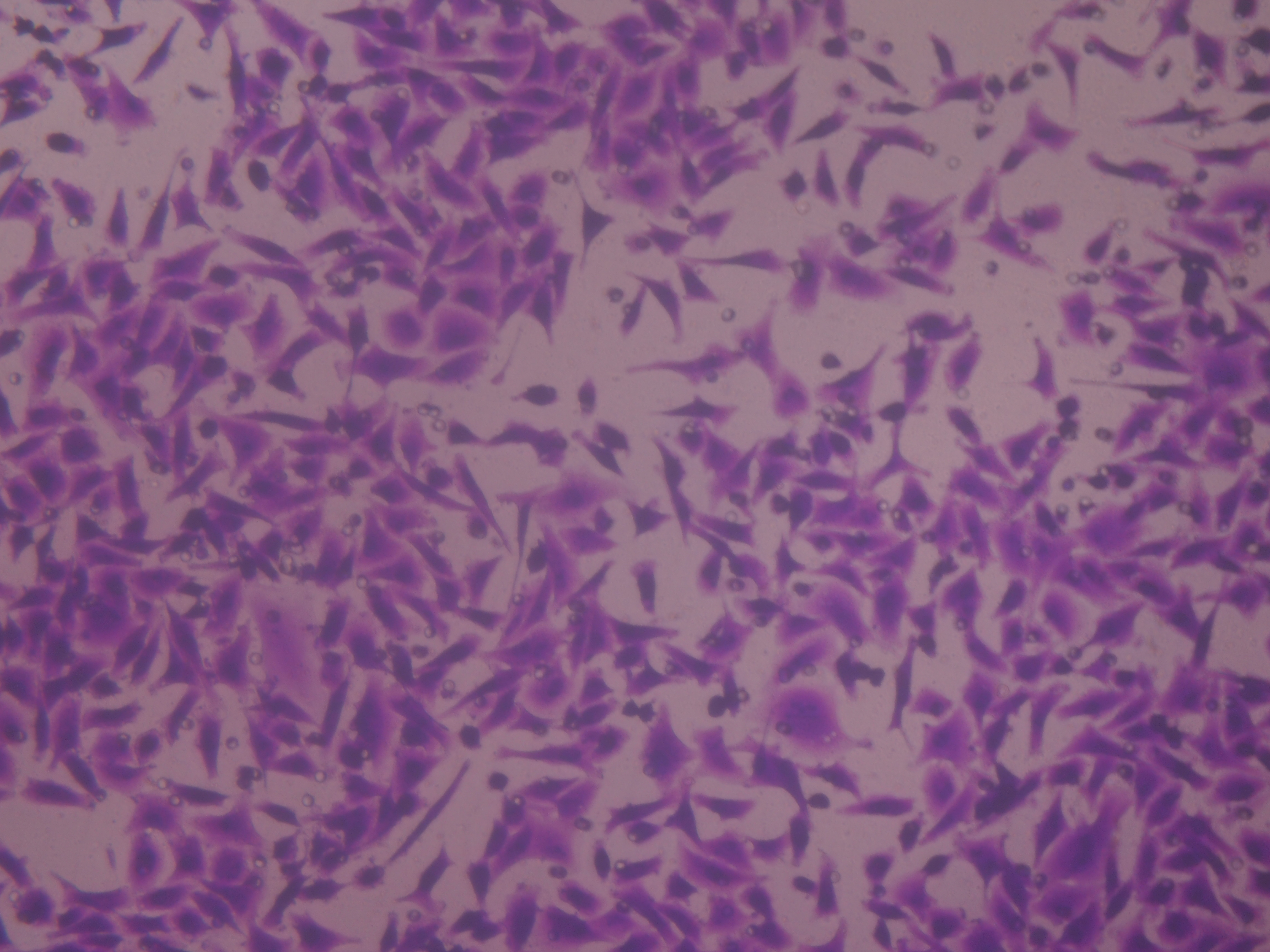

Supplement: Supplementary file 8 — Source data Fig. 7 [file 44319_2024_132_MOESM8_ESM.zip › Figure 7/7B/migration A549-HA-Ago2212R.tif]

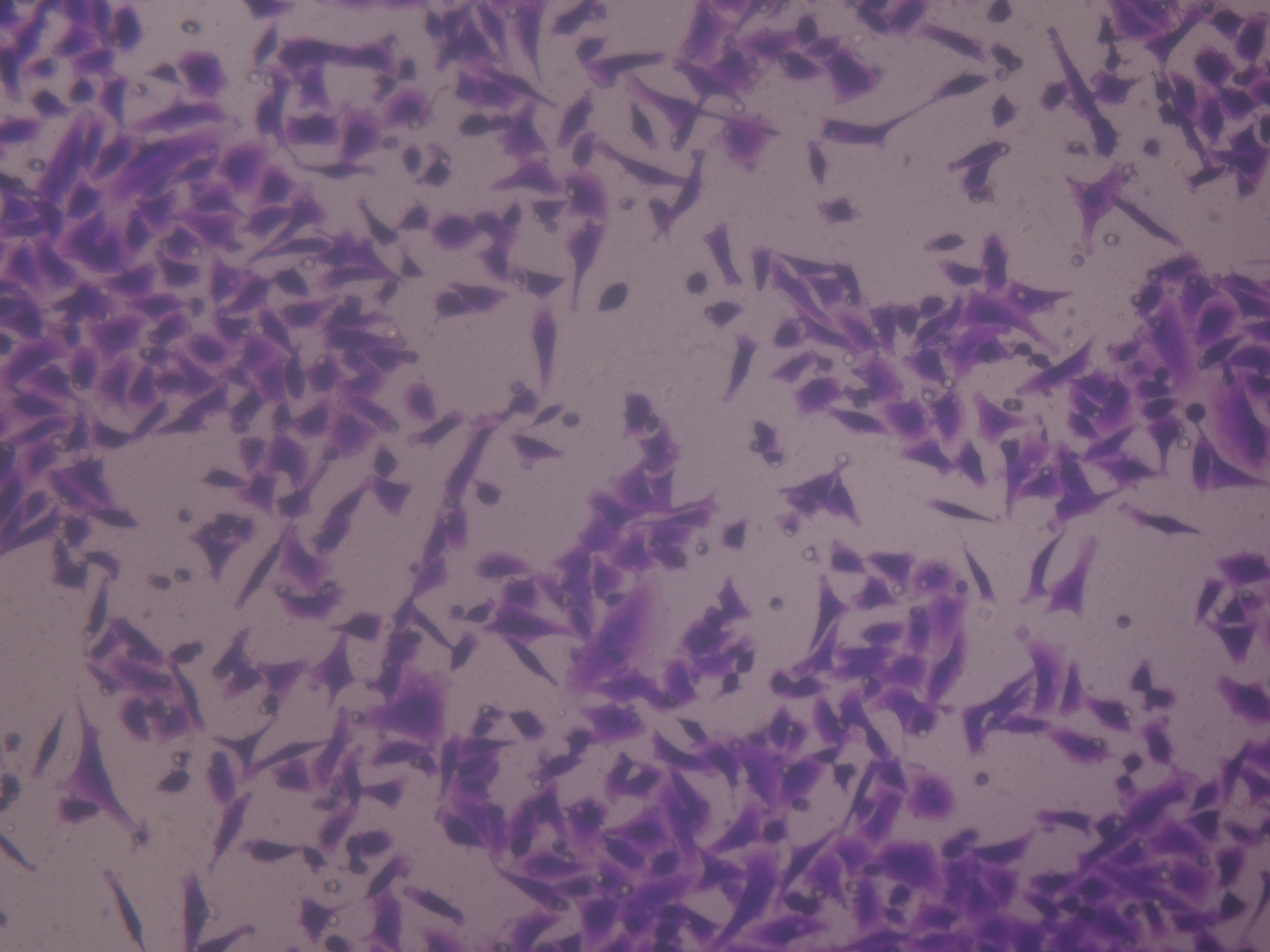

Supplement: Supplementary file 8 — Source data Fig. 7 [file 44319_2024_132_MOESM8_ESM.zip › Figure 7/7B/migration A549-HA-Ago2212R+TM.tif]

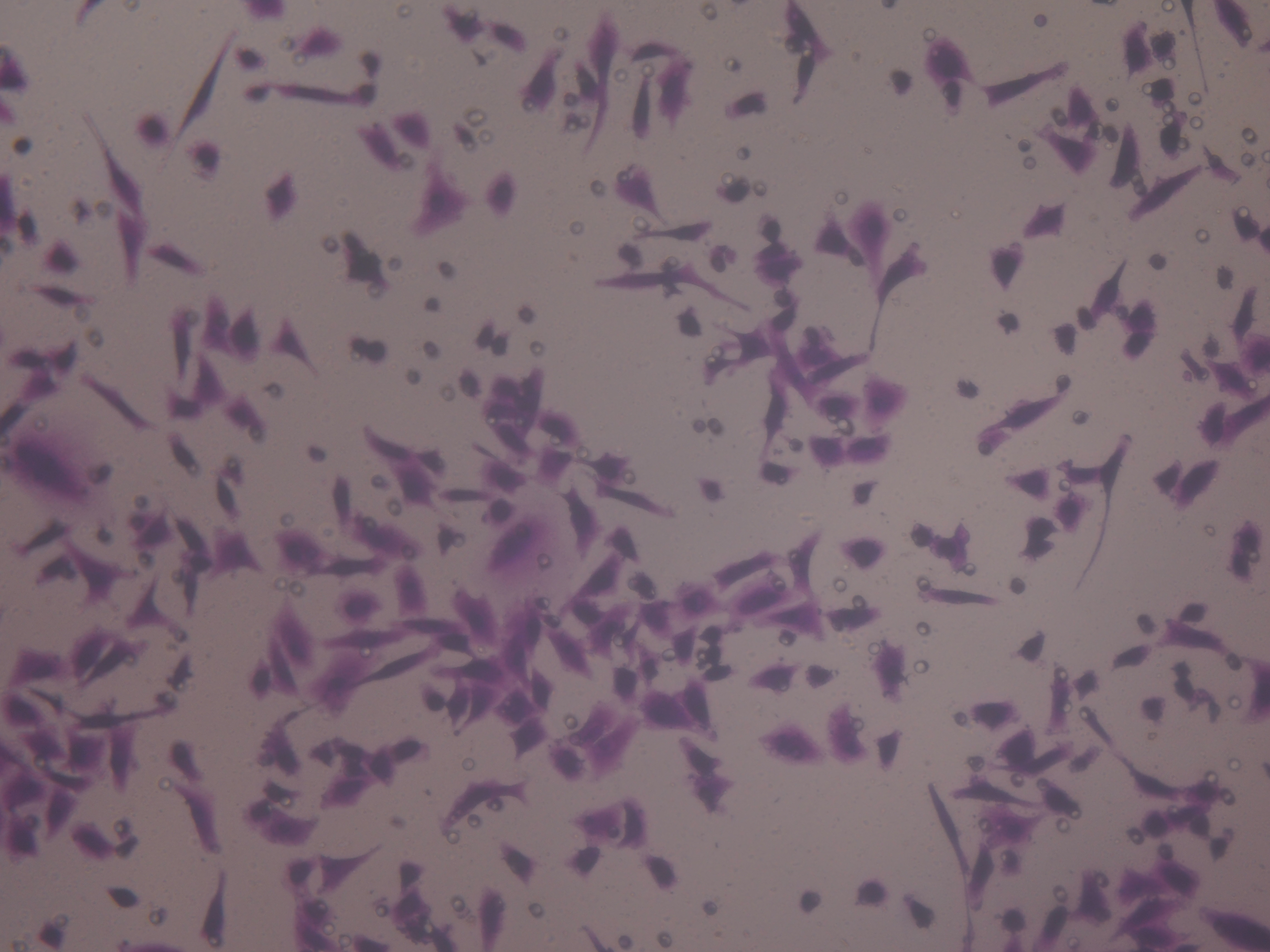

Supplement: Supplementary file 8 — Source data Fig. 7 [file 44319_2024_132_MOESM8_ESM.zip › Figure 7/7B/migration A549-HA-Ago2Wt + TM.tif]

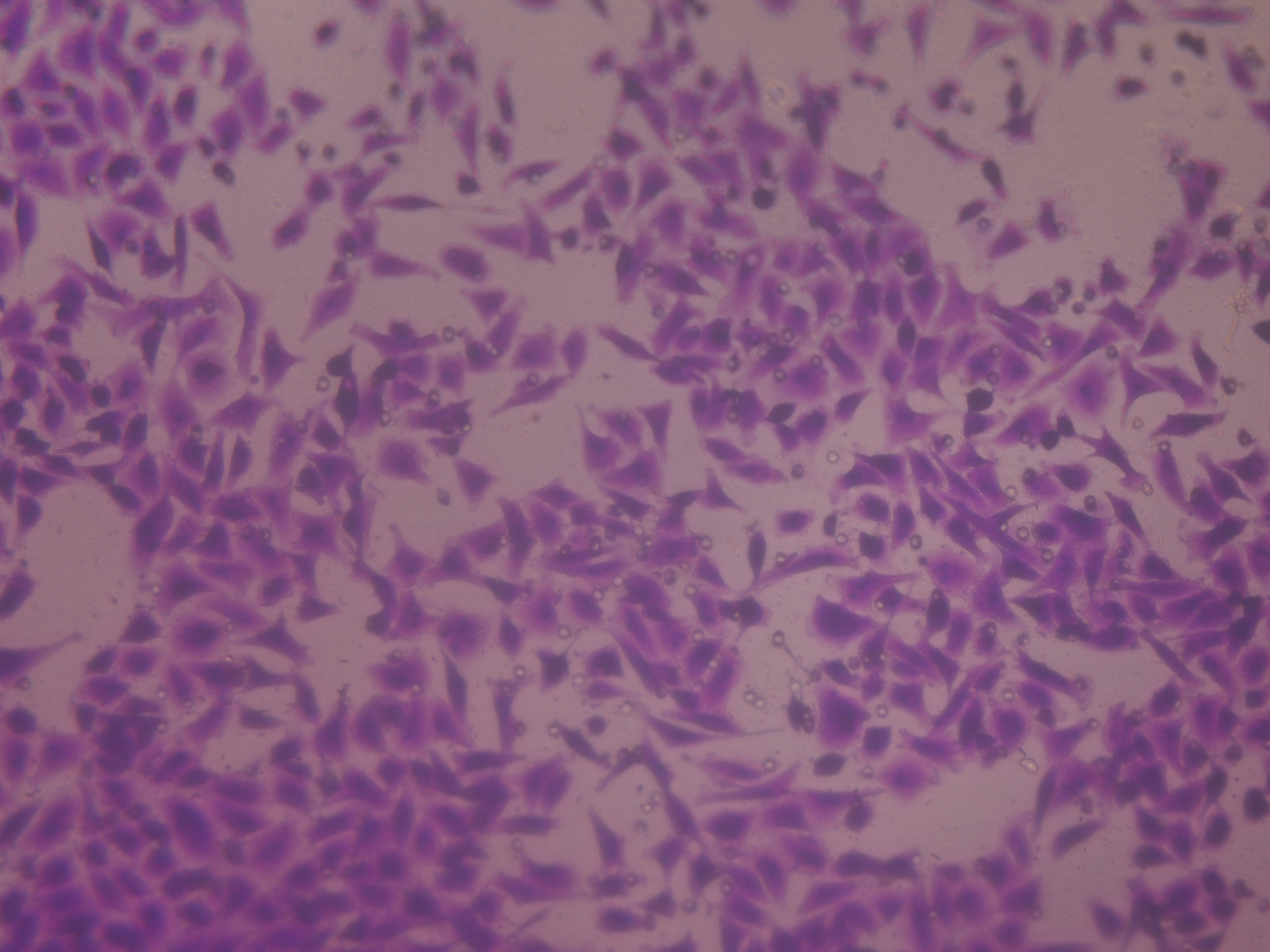

Supplement: Supplementary file 8 — Source data Fig. 7 [file 44319_2024_132_MOESM8_ESM.zip › Figure 7/7B/migration A549-HA-Ago2Wt.tif]

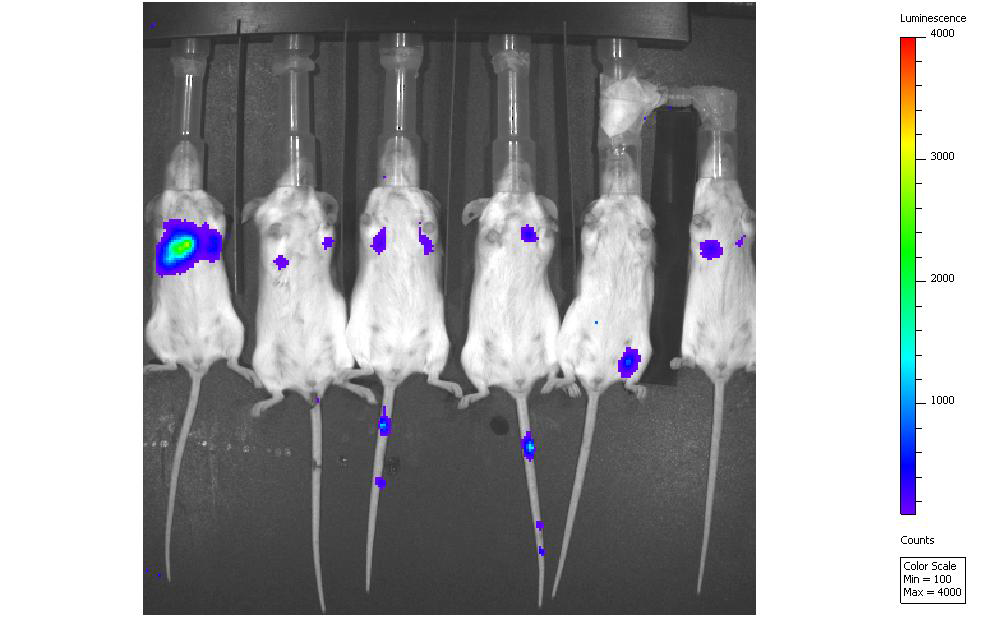

Supplement: Supplementary file 8 — Source data Fig. 7 [file 44319_2024_132_MOESM8_ESM.zip › Figure 7/7E/ii/Ago2-212A IVIS.tif]
