# Supplementary figures and images for: Ago2/CAV1 interaction potentiates metastasis via controlling Ago2 localization and miRNA action (part 3 of 3)
Source: EMBO Rep. 2024 Apr 22;25(5):20. doi: 10.1038/s44319-024-00132-7 (PMC11094075; doi:10.1038/s44319-024-00132-7)

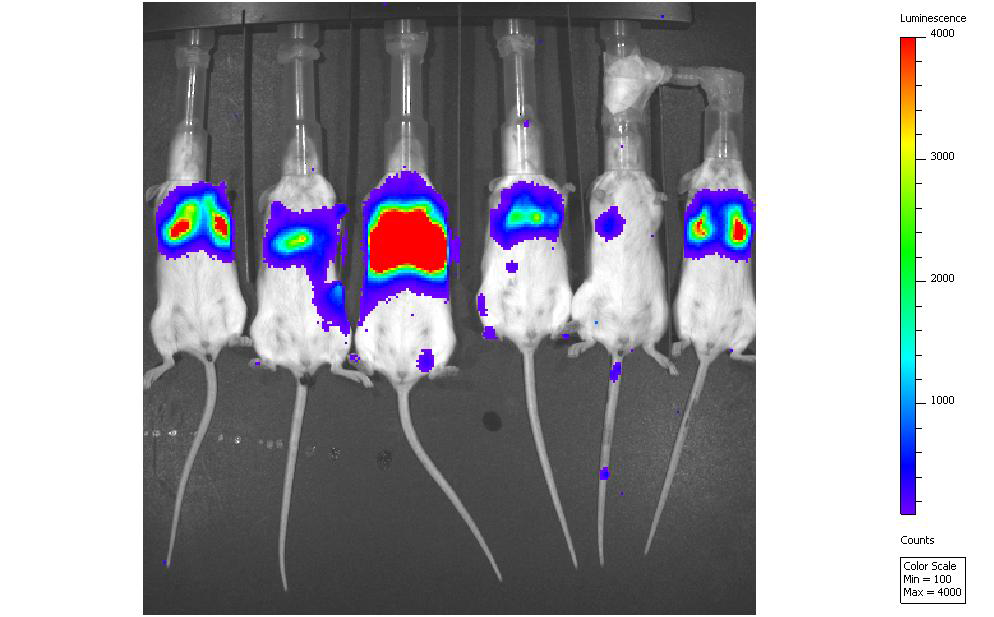

Supplement: Supplementary file 8 — Source data Fig. 7 [file 44319_2024_132_MOESM8_ESM.zip › Figure 7/7E/ii/Ago2-212R IVIS.tif]

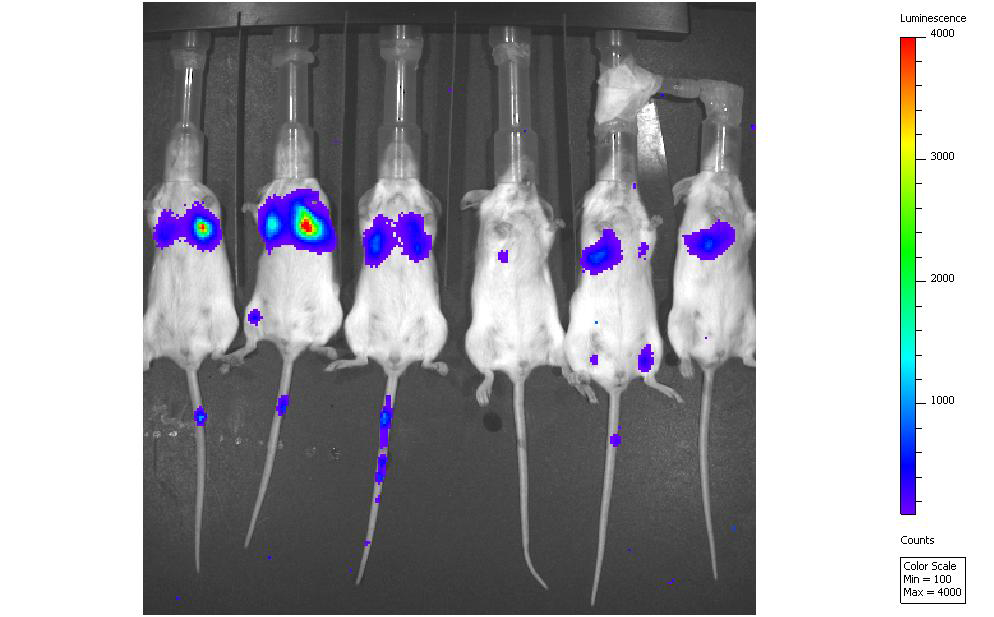

Supplement: Supplementary file 8 — Source data Fig. 7 [file 44319_2024_132_MOESM8_ESM.zip › Figure 7/7E/ii/Ago2-Wt IVIS.tif]

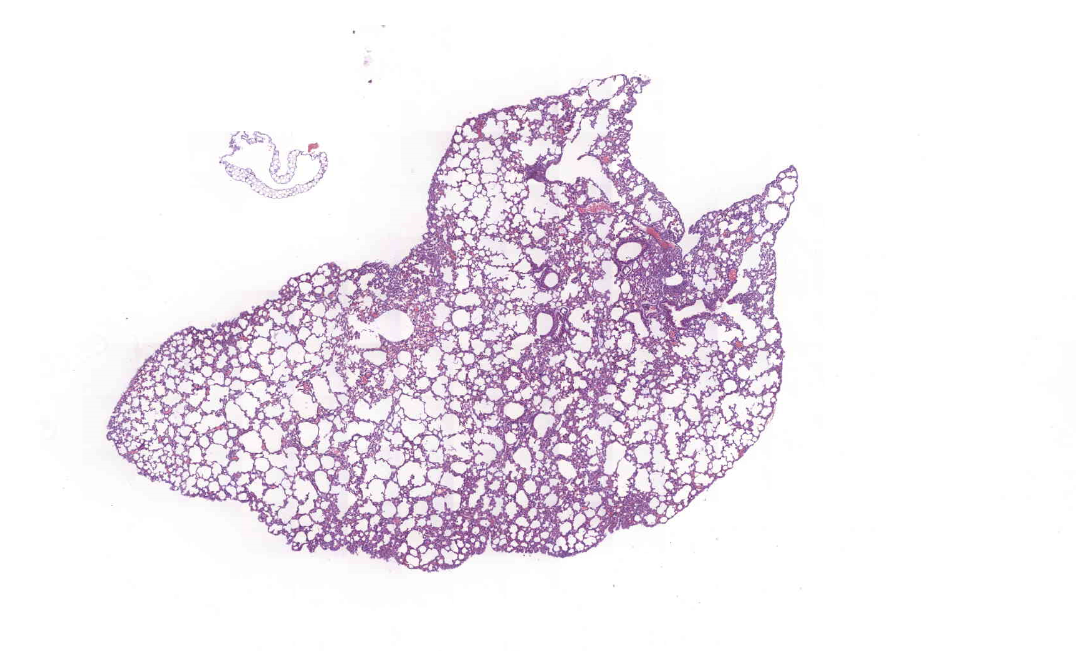

Supplement: Supplementary file 8 — Source data Fig. 7 [file 44319_2024_132_MOESM8_ESM.zip › Figure 7/7E/iv/K212A.tif]

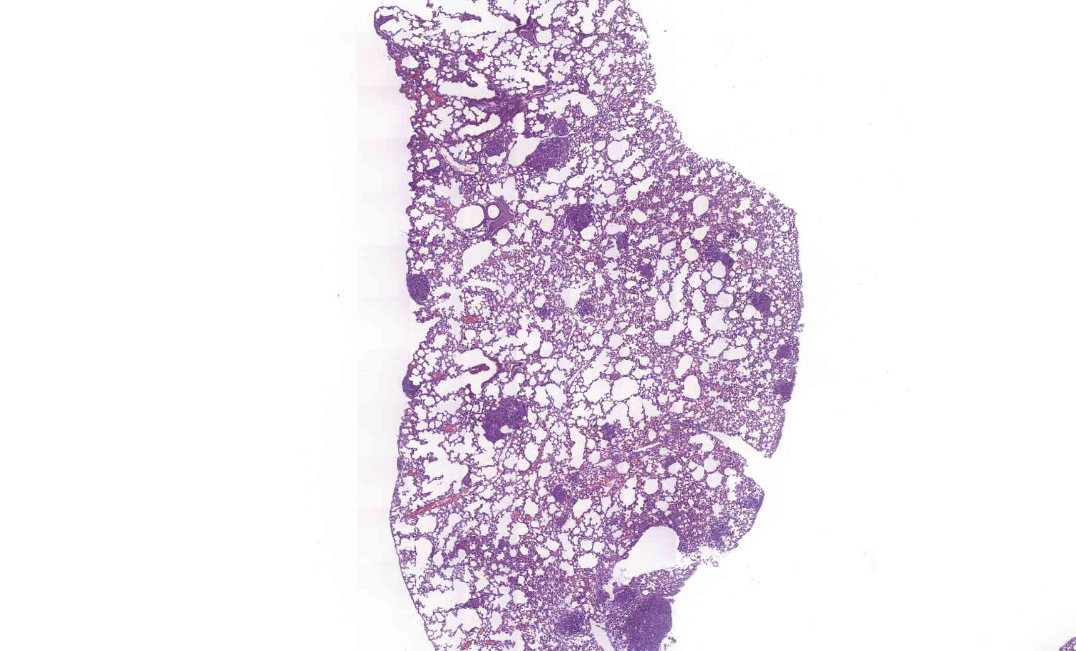

Supplement: Supplementary file 8 — Source data Fig. 7 [file 44319_2024_132_MOESM8_ESM.zip › Figure 7/7E/iv/K212R.tif]

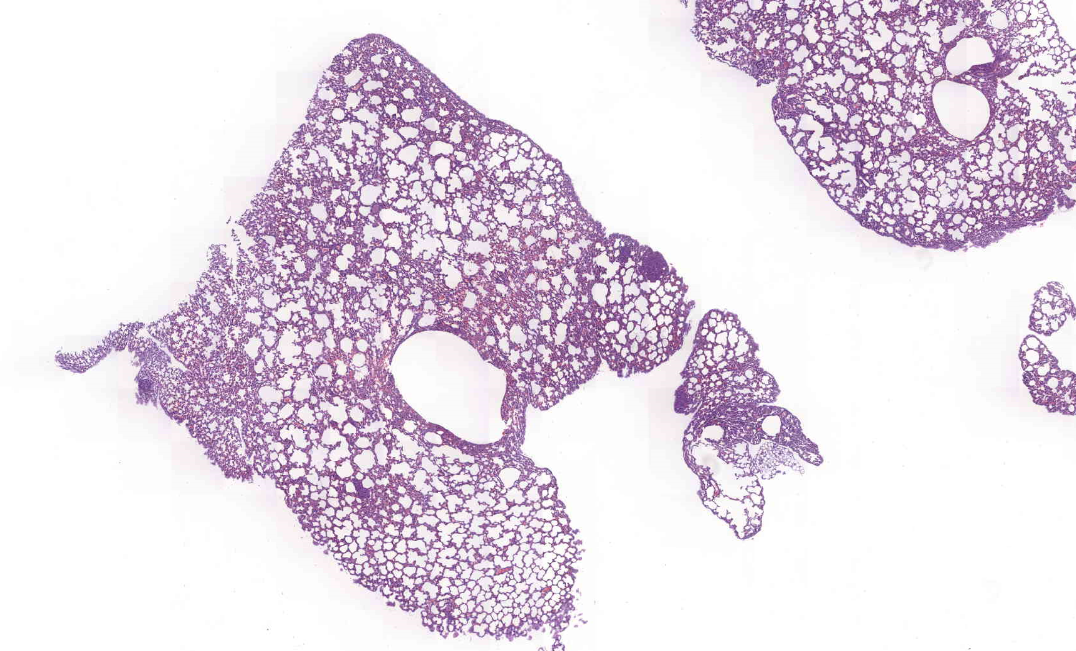

Supplement: Supplementary file 8 — Source data Fig. 7 [file 44319_2024_132_MOESM8_ESM.zip › Figure 7/7E/iv/Wt.tif]

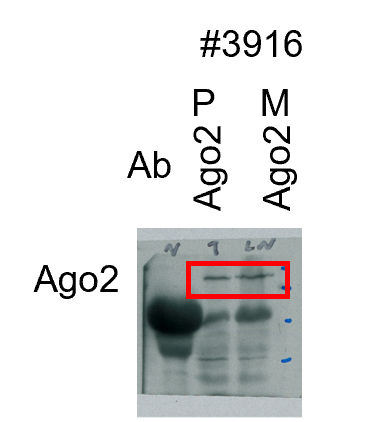

Supplement: Supplementary file 9 — Source data Fig. 8 [file 44319_2024_132_MOESM9_ESM.zip › Figure 8/8A/3916/western input Ago2.tif]

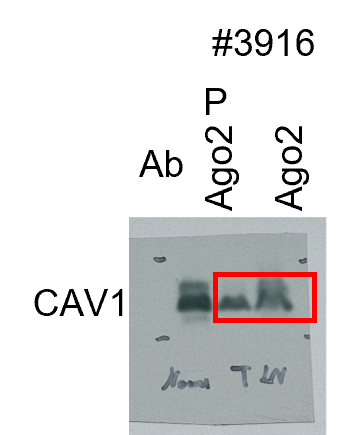

Supplement: Supplementary file 9 — Source data Fig. 8 [file 44319_2024_132_MOESM9_ESM.zip › Figure 8/8A/3916/western input CAV1.tif]

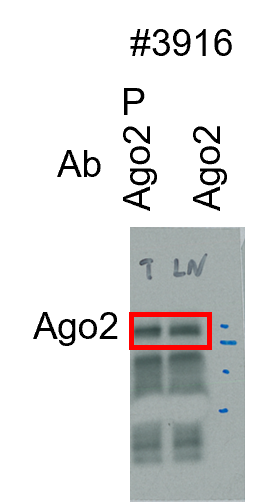

Supplement: Supplementary file 9 — Source data Fig. 8 [file 44319_2024_132_MOESM9_ESM.zip › Figure 8/8A/3916/western IP Ago2.tif]

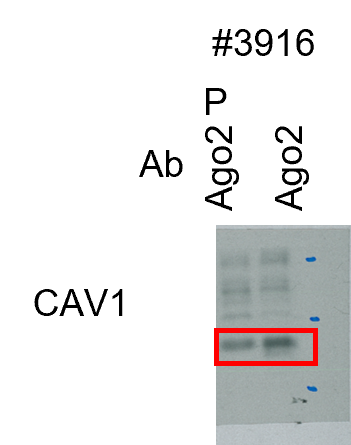

Supplement: Supplementary file 9 — Source data Fig. 8 [file 44319_2024_132_MOESM9_ESM.zip › Figure 8/8A/3916/western IP CAV1.tif]

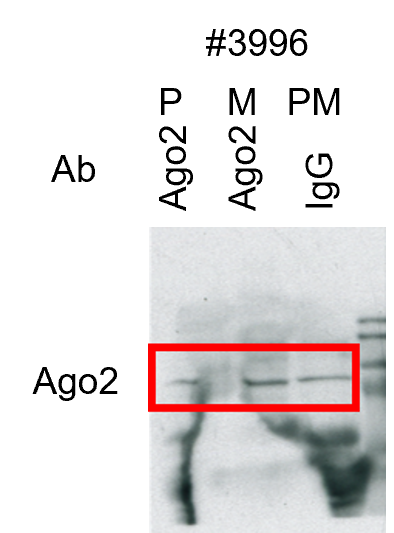

Supplement: Supplementary file 9 — Source data Fig. 8 [file 44319_2024_132_MOESM9_ESM.zip › Figure 8/8A/3996 4014 4137/western input Ago2 3996.tif]

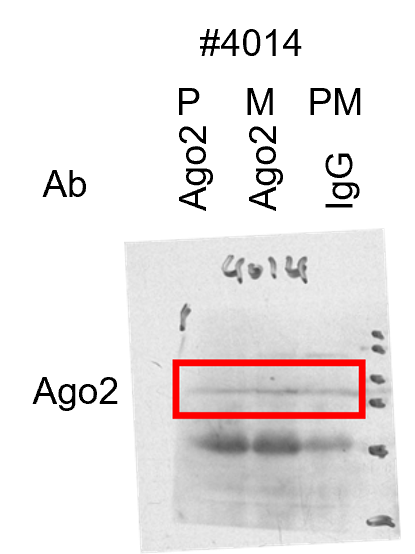

Supplement: Supplementary file 9 — Source data Fig. 8 [file 44319_2024_132_MOESM9_ESM.zip › Figure 8/8A/3996 4014 4137/western input Ago2 4014.tif]

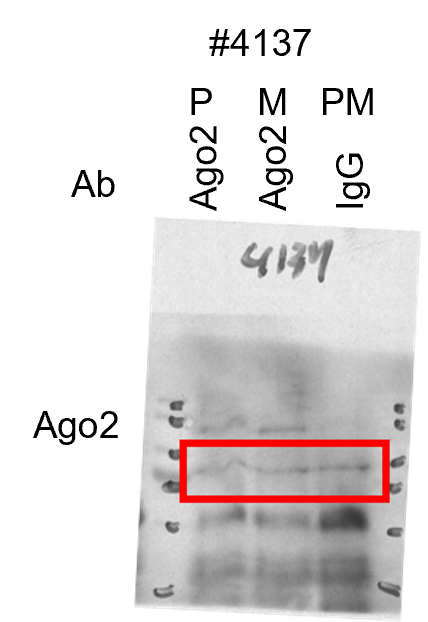

Supplement: Supplementary file 9 — Source data Fig. 8 [file 44319_2024_132_MOESM9_ESM.zip › Figure 8/8A/3996 4014 4137/western input Ago2 4137.tif]

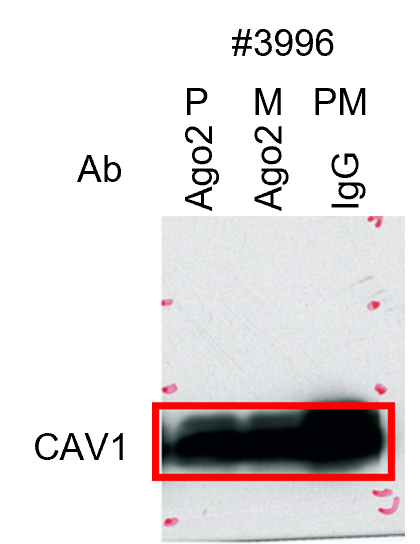

Supplement: Supplementary file 9 — Source data Fig. 8 [file 44319_2024_132_MOESM9_ESM.zip › Figure 8/8A/3996 4014 4137/western input CAV1 3996.tif]

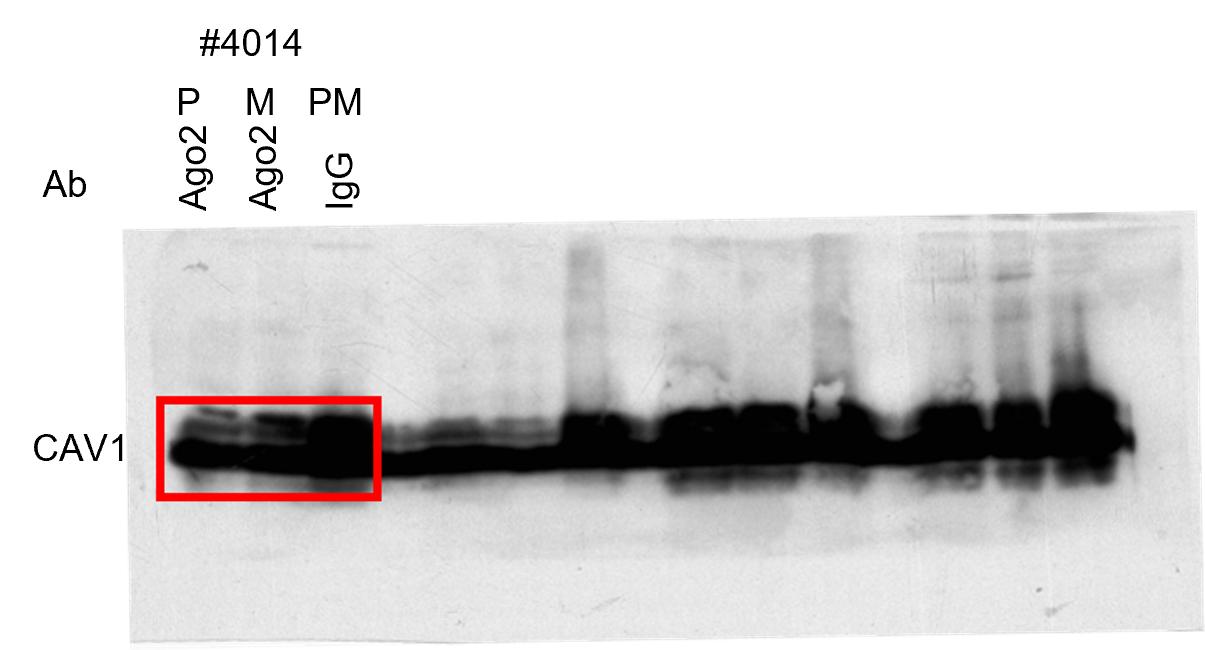

Supplement: Supplementary file 9 — Source data Fig. 8 [file 44319_2024_132_MOESM9_ESM.zip › Figure 8/8A/3996 4014 4137/western input CAV1 4014.tif]

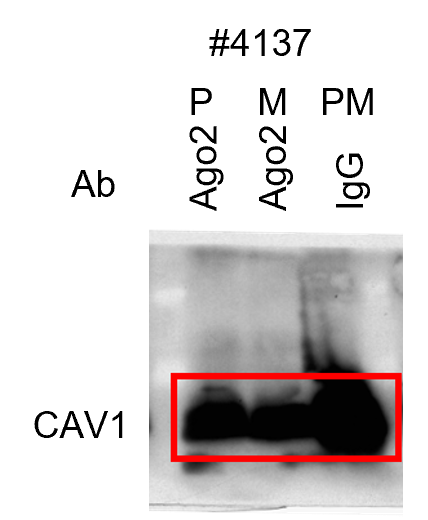

Supplement: Supplementary file 9 — Source data Fig. 8 [file 44319_2024_132_MOESM9_ESM.zip › Figure 8/8A/3996 4014 4137/western input CAV1 4137S.tif]

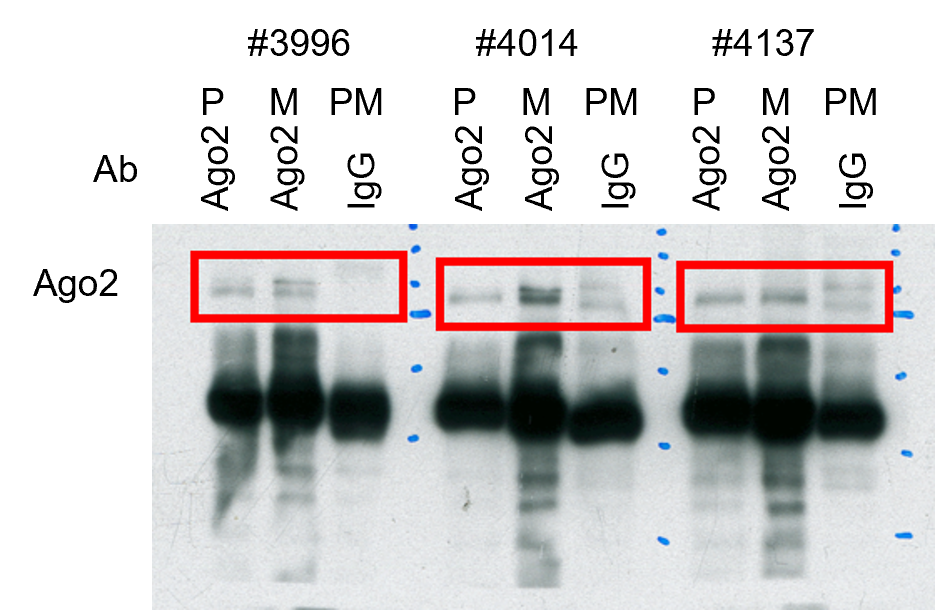

Supplement: Supplementary file 9 — Source data Fig. 8 [file 44319_2024_132_MOESM9_ESM.zip › Figure 8/8A/3996 4014 4137/western IP Ago2.tif]

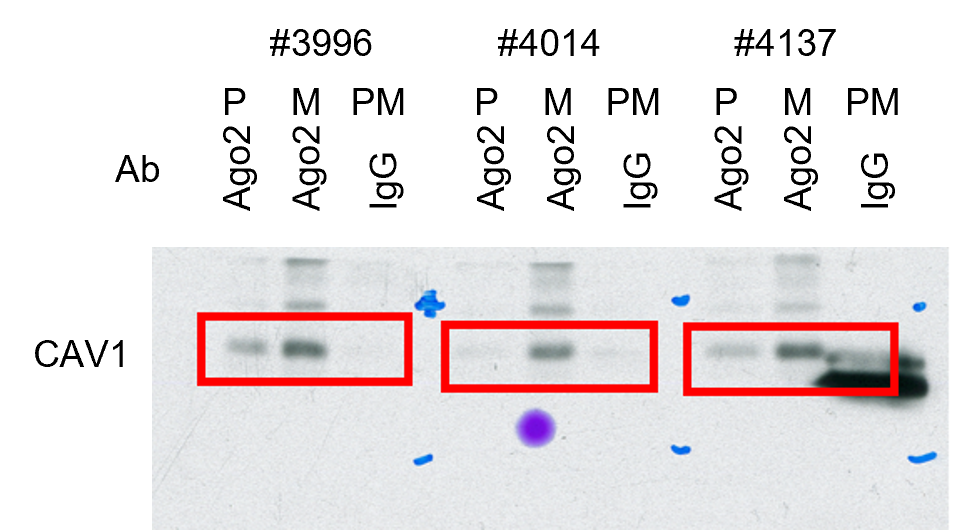

Supplement: Supplementary file 9 — Source data Fig. 8 [file 44319_2024_132_MOESM9_ESM.zip › Figure 8/8A/3996 4014 4137/western IP CAV1.tif]

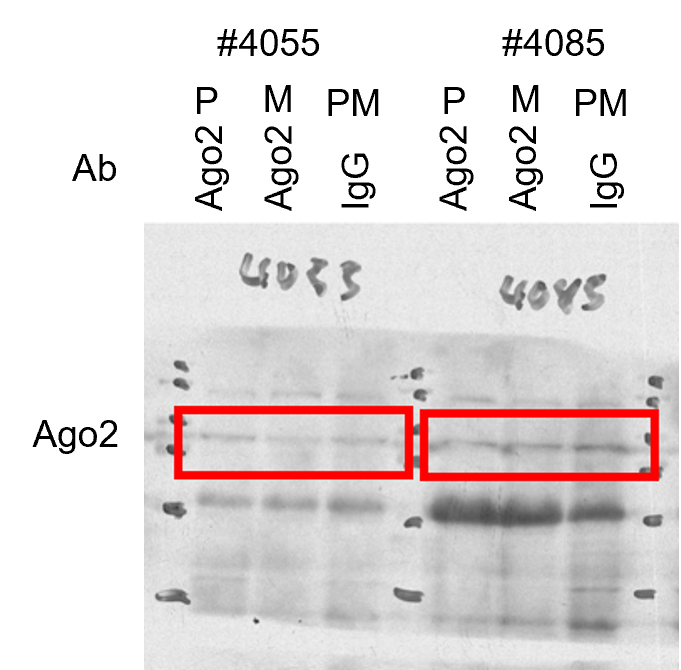

Supplement: Supplementary file 9 — Source data Fig. 8 [file 44319_2024_132_MOESM9_ESM.zip › Figure 8/8A/4055 4085/western input Ago2 4055 4085.tif]

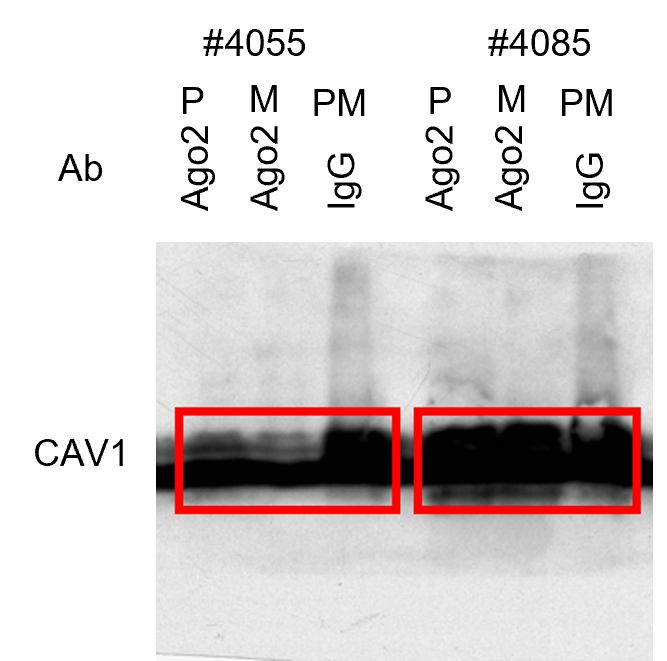

Supplement: Supplementary file 9 — Source data Fig. 8 [file 44319_2024_132_MOESM9_ESM.zip › Figure 8/8A/4055 4085/western input CAV1 4055 4085.tif]

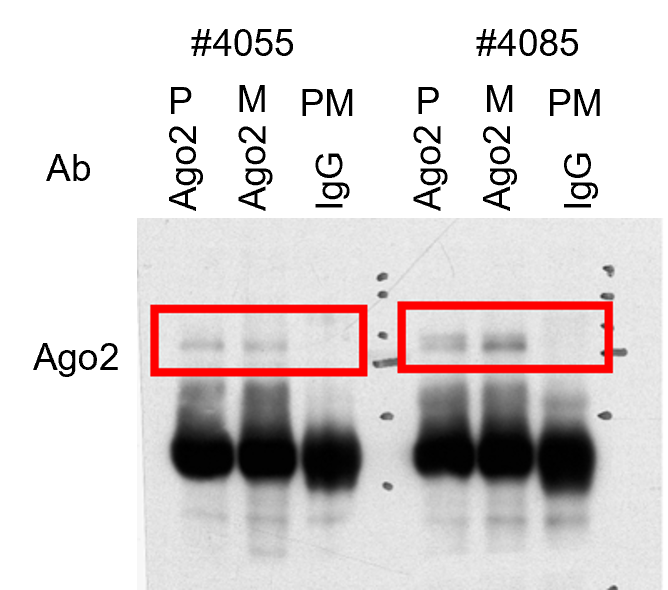

Supplement: Supplementary file 9 — Source data Fig. 8 [file 44319_2024_132_MOESM9_ESM.zip › Figure 8/8A/4055 4085/western IP Ago2 4055 4085.tif]

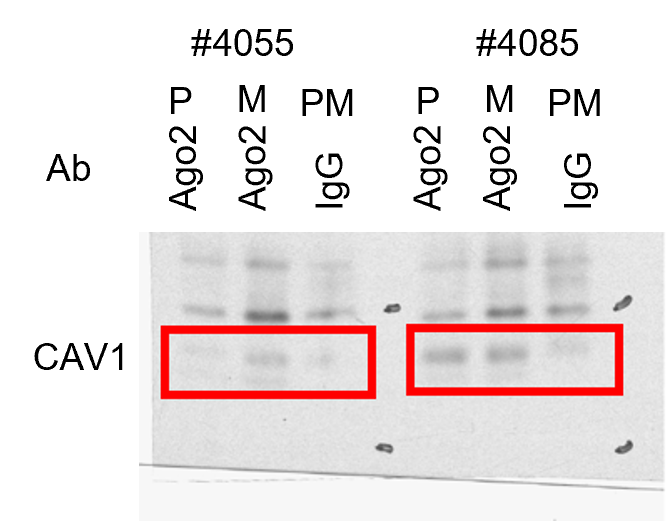

Supplement: Supplementary file 9 — Source data Fig. 8 [file 44319_2024_132_MOESM9_ESM.zip › Figure 8/8A/4055 4085/western IP CAV1 4055 4085.tif]

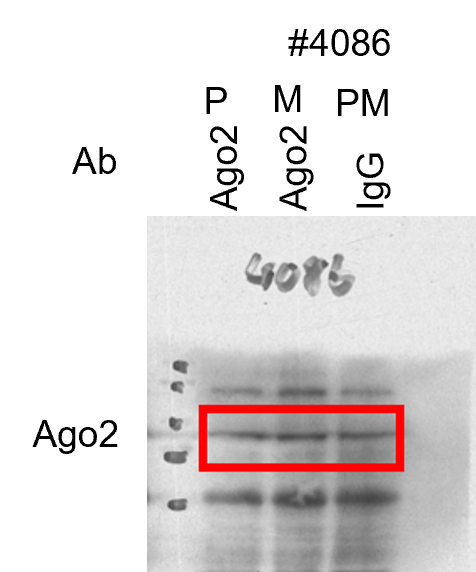

Supplement: Supplementary file 9 — Source data Fig. 8 [file 44319_2024_132_MOESM9_ESM.zip › Figure 8/8A/4086/western input Ago2 4086.tif]

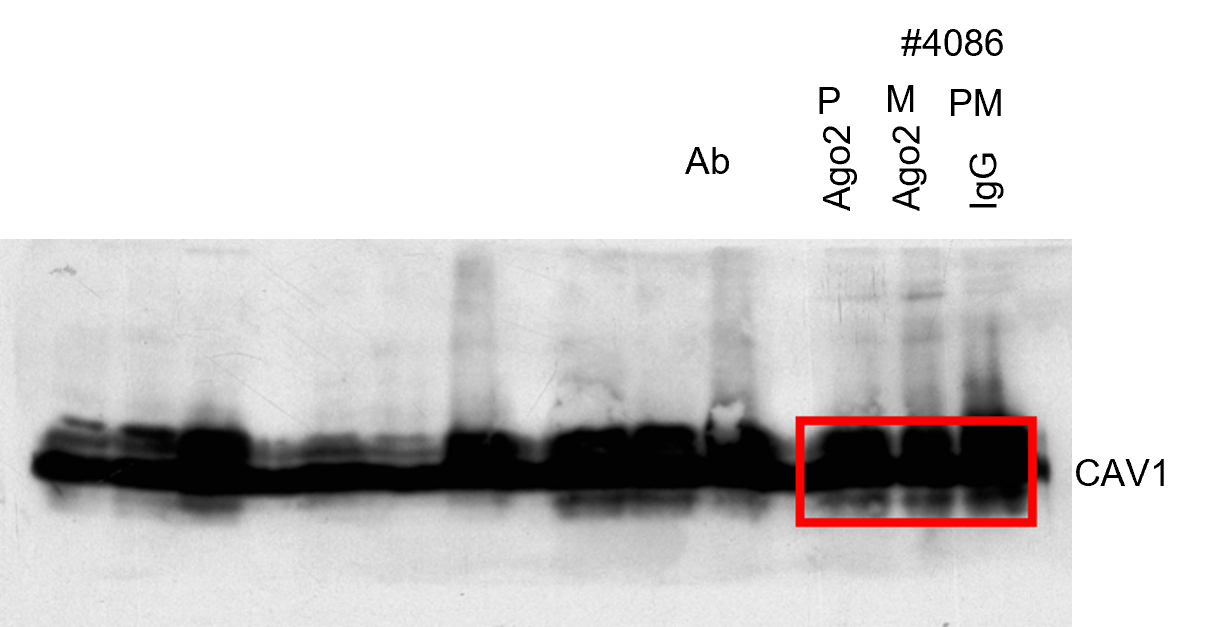

Supplement: Supplementary file 9 — Source data Fig. 8 [file 44319_2024_132_MOESM9_ESM.zip › Figure 8/8A/4086/western input CAV1 4086.tif]

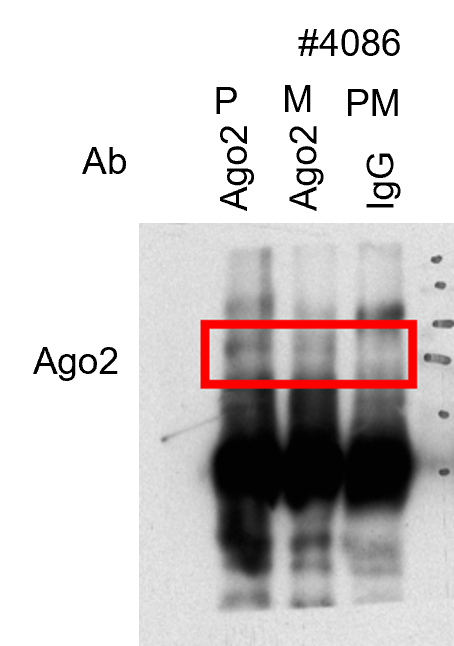

Supplement: Supplementary file 9 — Source data Fig. 8 [file 44319_2024_132_MOESM9_ESM.zip › Figure 8/8A/4086/western IP Ago2 4086.tif]

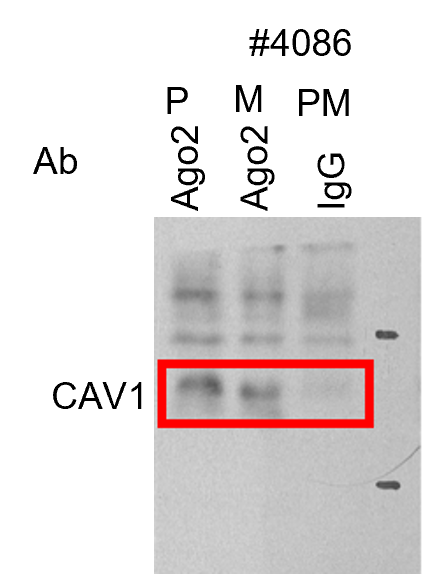

Supplement: Supplementary file 9 — Source data Fig. 8 [file 44319_2024_132_MOESM9_ESM.zip › Figure 8/8A/4086/western IP CAV1 4086.tif]

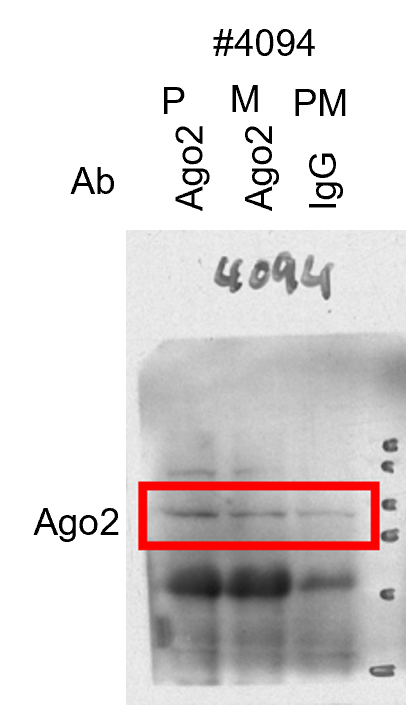

Supplement: Supplementary file 9 — Source data Fig. 8 [file 44319_2024_132_MOESM9_ESM.zip › Figure 8/8A/4094/western input Ago2 4094.tif]

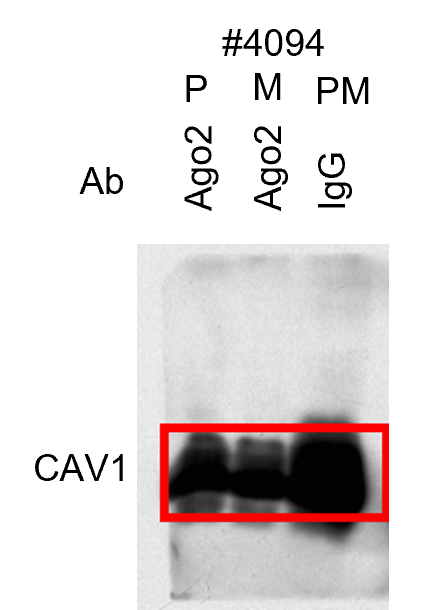

Supplement: Supplementary file 9 — Source data Fig. 8 [file 44319_2024_132_MOESM9_ESM.zip › Figure 8/8A/4094/western input CAV1 4094.tif]

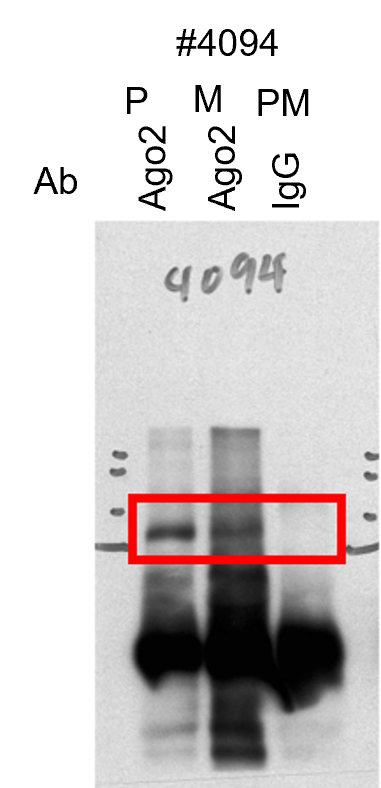

Supplement: Supplementary file 9 — Source data Fig. 8 [file 44319_2024_132_MOESM9_ESM.zip › Figure 8/8A/4094/western IP Ago2 4094.tif]

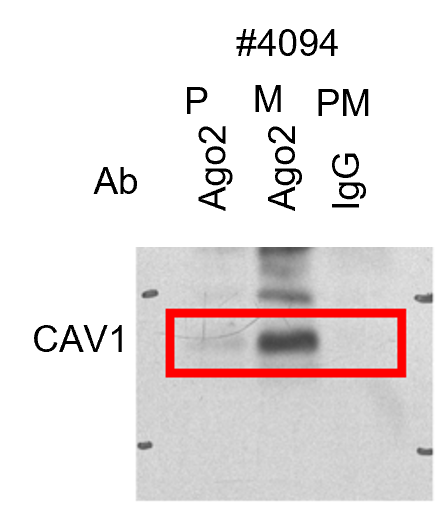

Supplement: Supplementary file 9 — Source data Fig. 8 [file 44319_2024_132_MOESM9_ESM.zip › Figure 8/8A/4094/western IP CAV1 4094.tif]

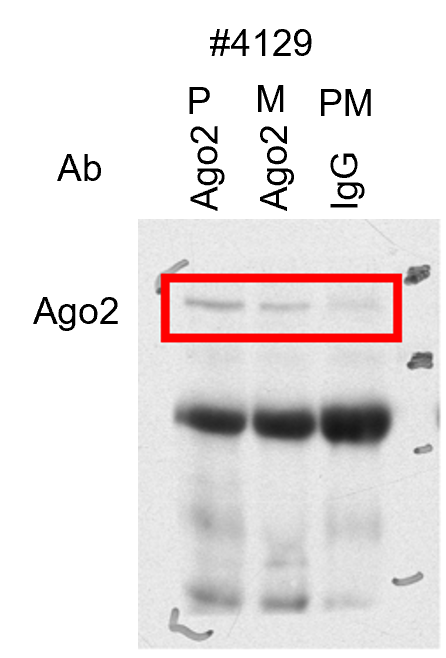

Supplement: Supplementary file 9 — Source data Fig. 8 [file 44319_2024_132_MOESM9_ESM.zip › Figure 8/8A/4129/western input Ago2 4129.tif]

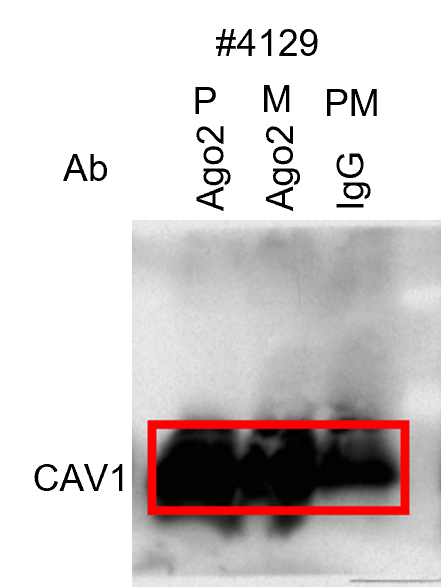

Supplement: Supplementary file 9 — Source data Fig. 8 [file 44319_2024_132_MOESM9_ESM.zip › Figure 8/8A/4129/western input CAV1 4129.tif]

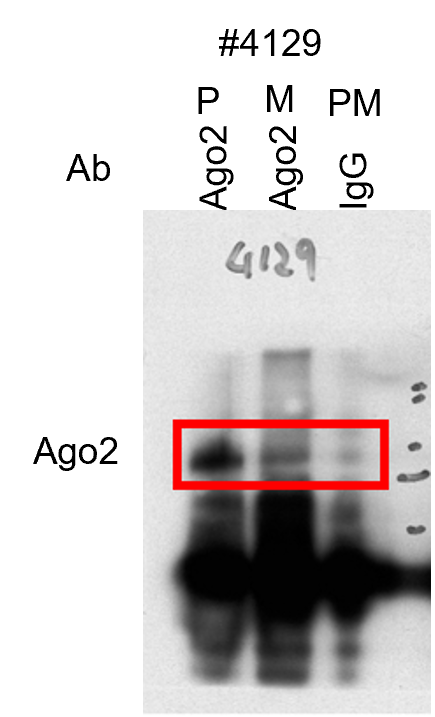

Supplement: Supplementary file 9 — Source data Fig. 8 [file 44319_2024_132_MOESM9_ESM.zip › Figure 8/8A/4129/western IP Ago2 4129.tif]

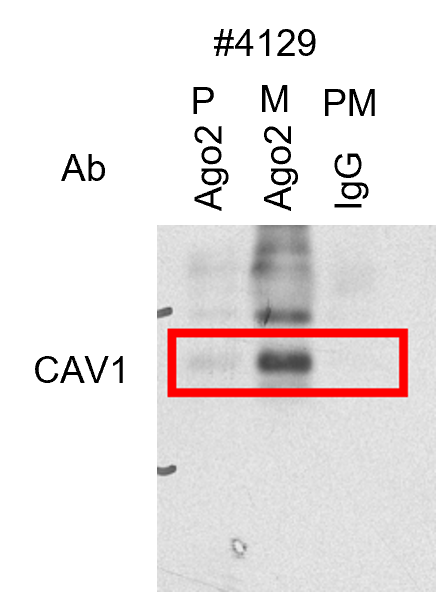

Supplement: Supplementary file 9 — Source data Fig. 8 [file 44319_2024_132_MOESM9_ESM.zip › Figure 8/8A/4129/western IP CAV1 4129.tif]

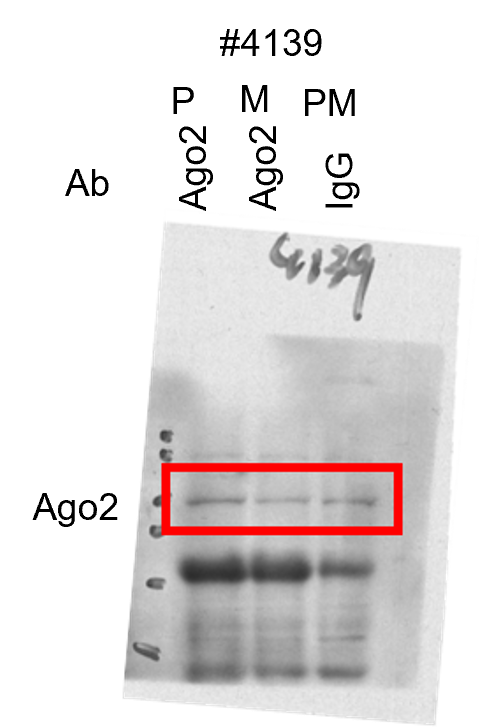

Supplement: Supplementary file 9 — Source data Fig. 8 [file 44319_2024_132_MOESM9_ESM.zip › Figure 8/8A/4139/western input Ago2 4139.tif]

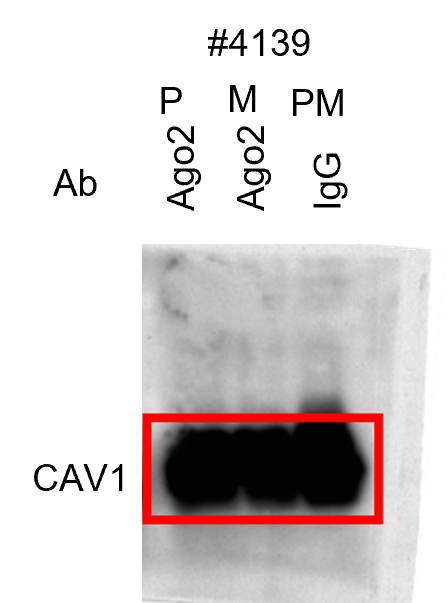

Supplement: Supplementary file 9 — Source data Fig. 8 [file 44319_2024_132_MOESM9_ESM.zip › Figure 8/8A/4139/western input CAV1 4139.tif]

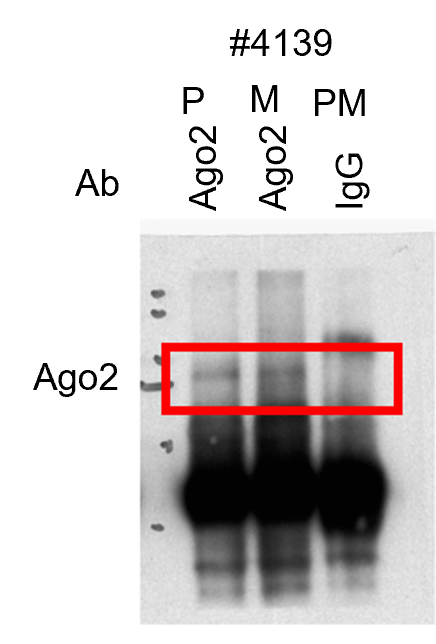

Supplement: Supplementary file 9 — Source data Fig. 8 [file 44319_2024_132_MOESM9_ESM.zip › Figure 8/8A/4139/western IP Ago2 4139.tif]

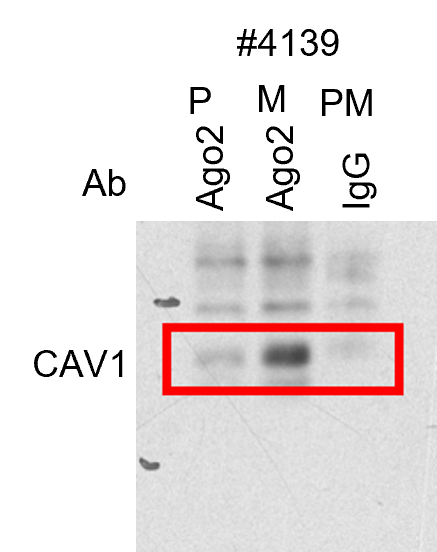

Supplement: Supplementary file 9 — Source data Fig. 8 [file 44319_2024_132_MOESM9_ESM.zip › Figure 8/8A/4139/western IP CAV1 4139.tif]

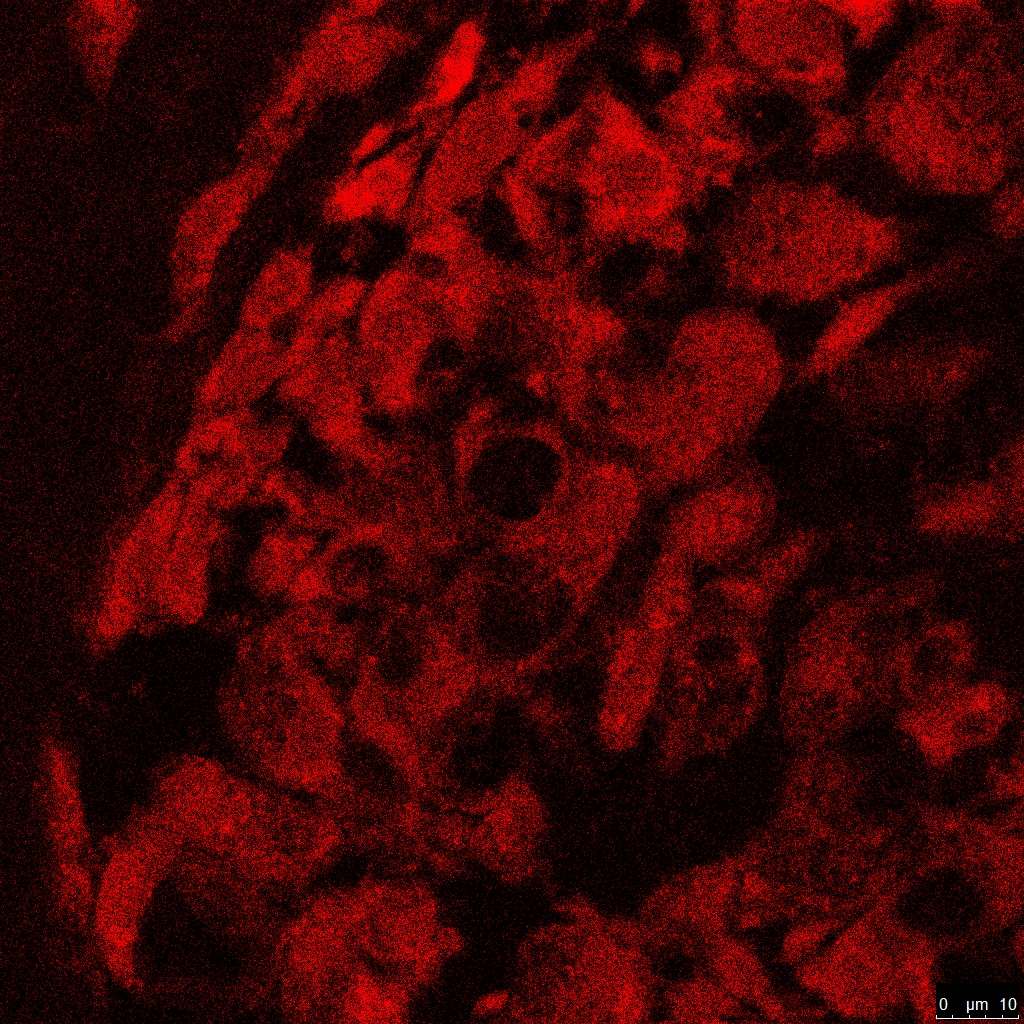

Supplement: Supplementary file 9 — Source data Fig. 8 [file 44319_2024_132_MOESM9_ESM.zip › Figure 8/8C/Dm/Ago2.tif]

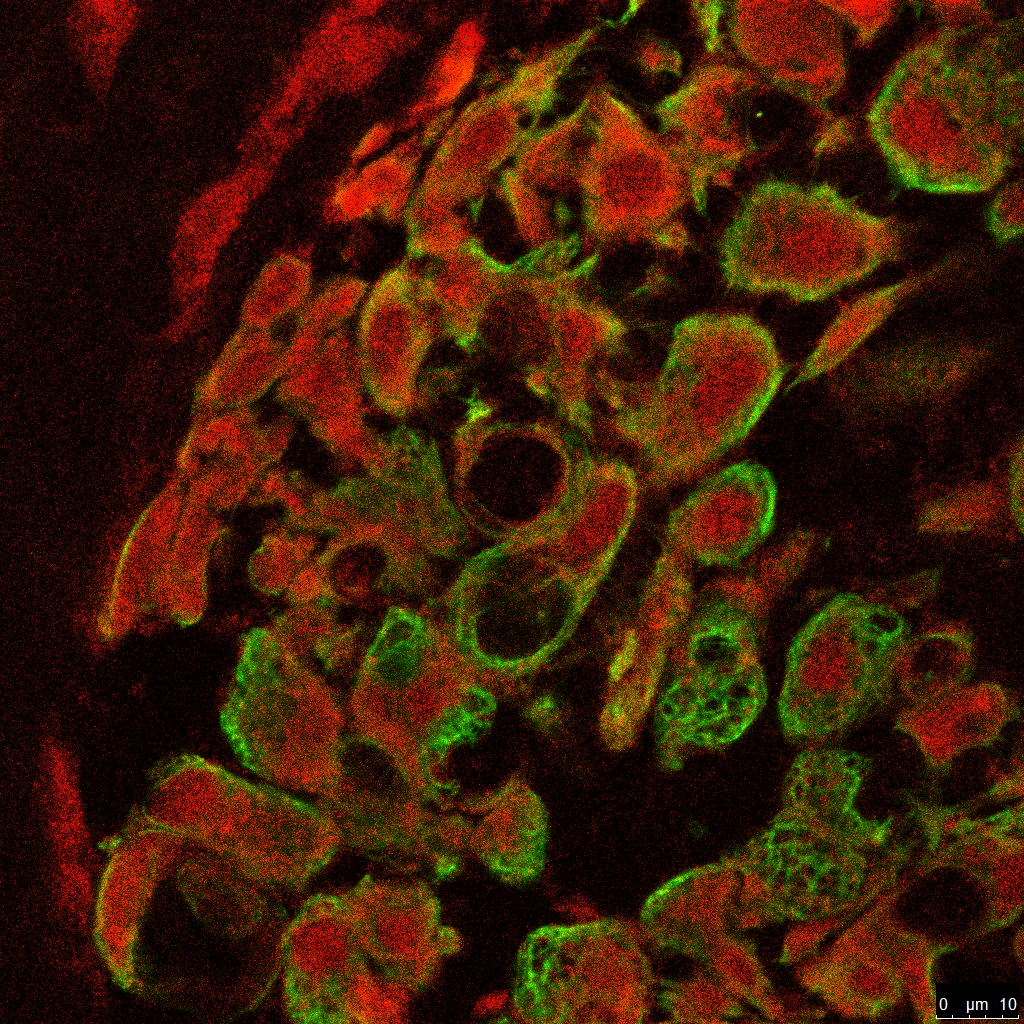

Supplement: Supplementary file 9 — Source data Fig. 8 [file 44319_2024_132_MOESM9_ESM.zip › Figure 8/8C/Dm/Ago2-Cytokeratin.tif]

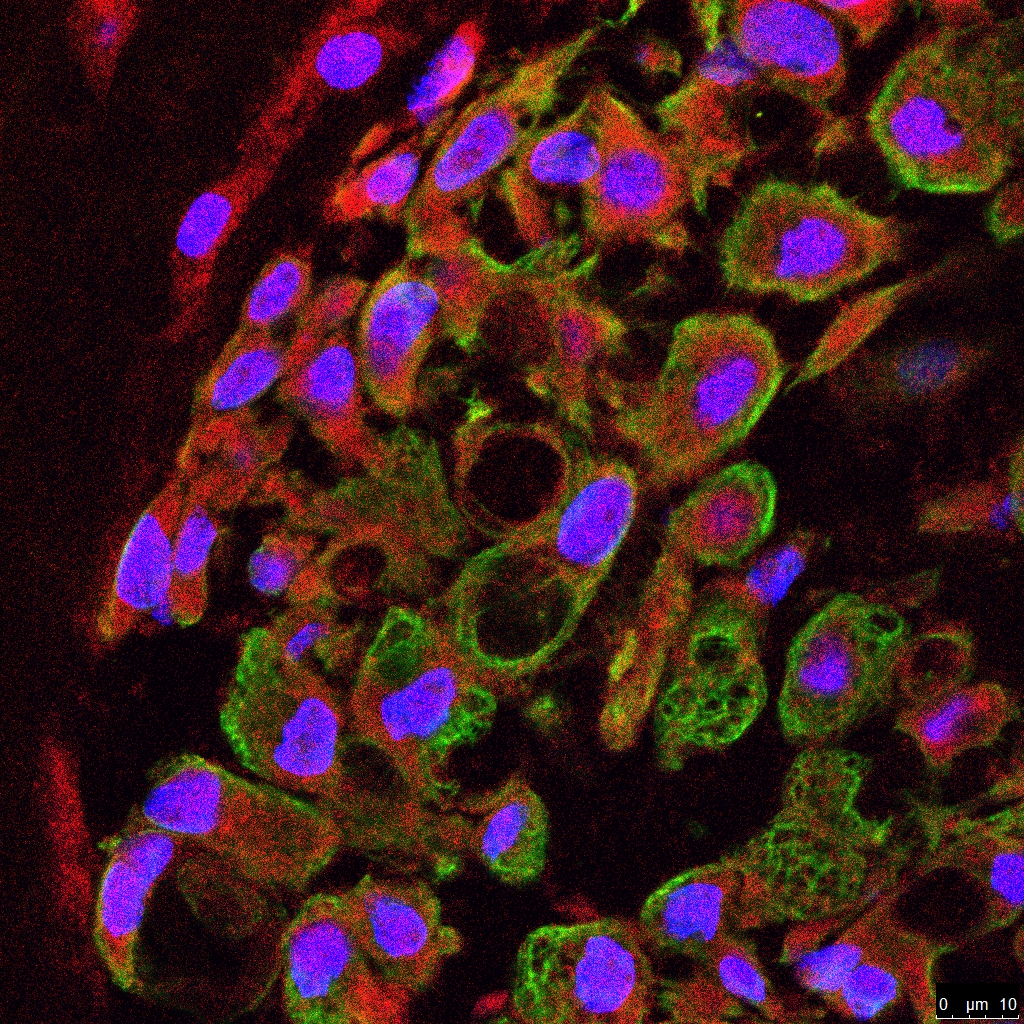

Supplement: Supplementary file 9 — Source data Fig. 8 [file 44319_2024_132_MOESM9_ESM.zip › Figure 8/8C/Dm/Ago2-Cytokeratin-DAPI.tif]

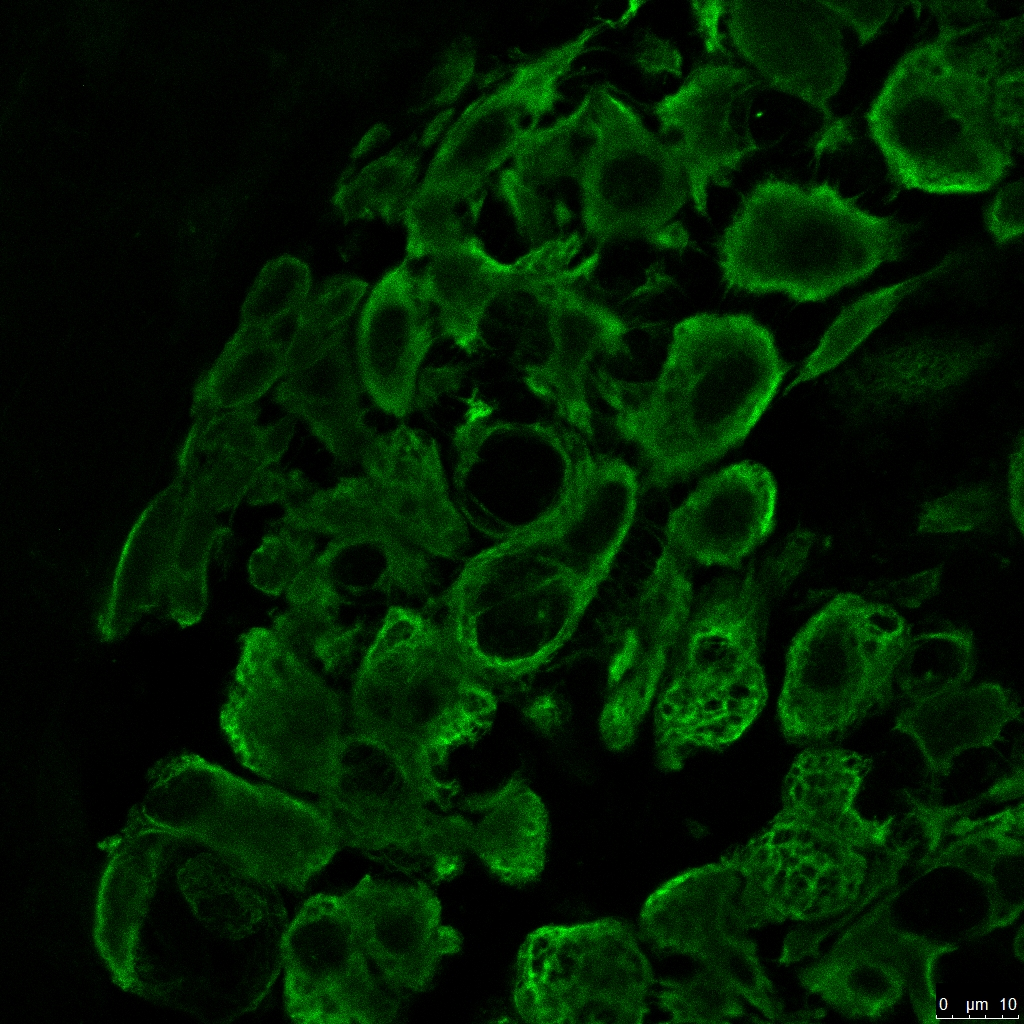

Supplement: Supplementary file 9 — Source data Fig. 8 [file 44319_2024_132_MOESM9_ESM.zip › Figure 8/8C/Dm/Cytokeratin.tif]

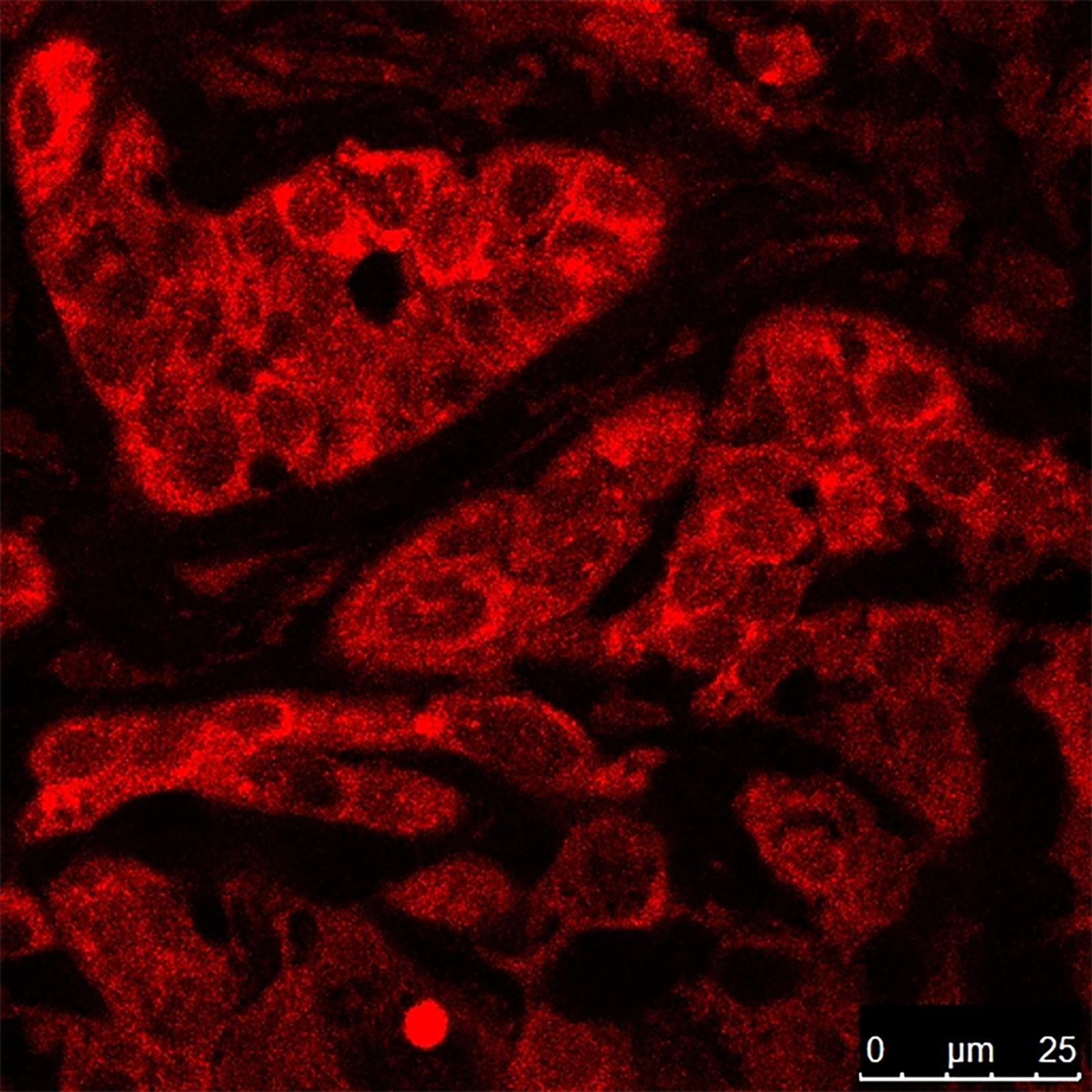

Supplement: Supplementary file 9 — Source data Fig. 8 [file 44319_2024_132_MOESM9_ESM.zip › Figure 8/8C/Wt/Ago2.tif]

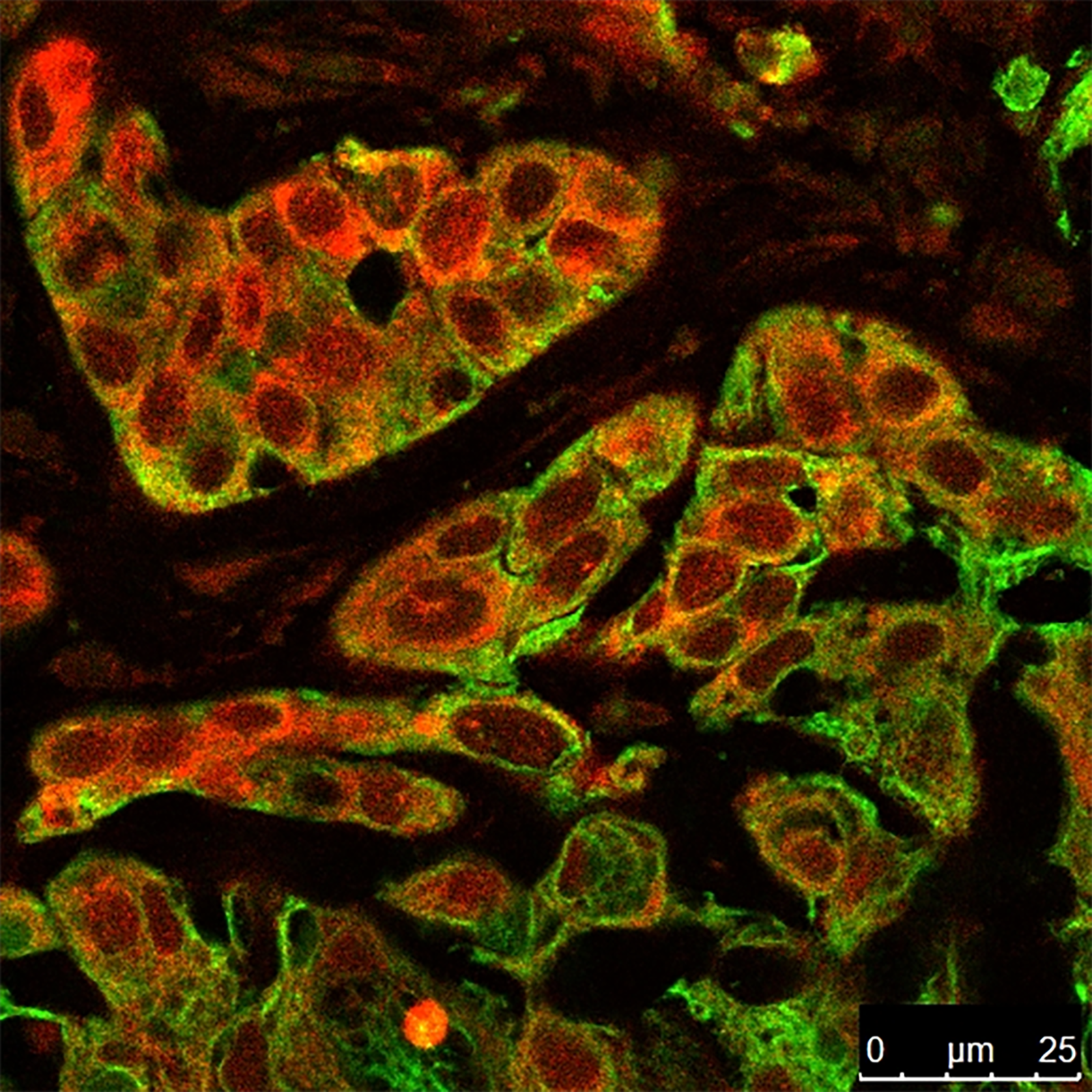

Supplement: Supplementary file 9 — Source data Fig. 8 [file 44319_2024_132_MOESM9_ESM.zip › Figure 8/8C/Wt/Ago2-Cytokeratin.tif]

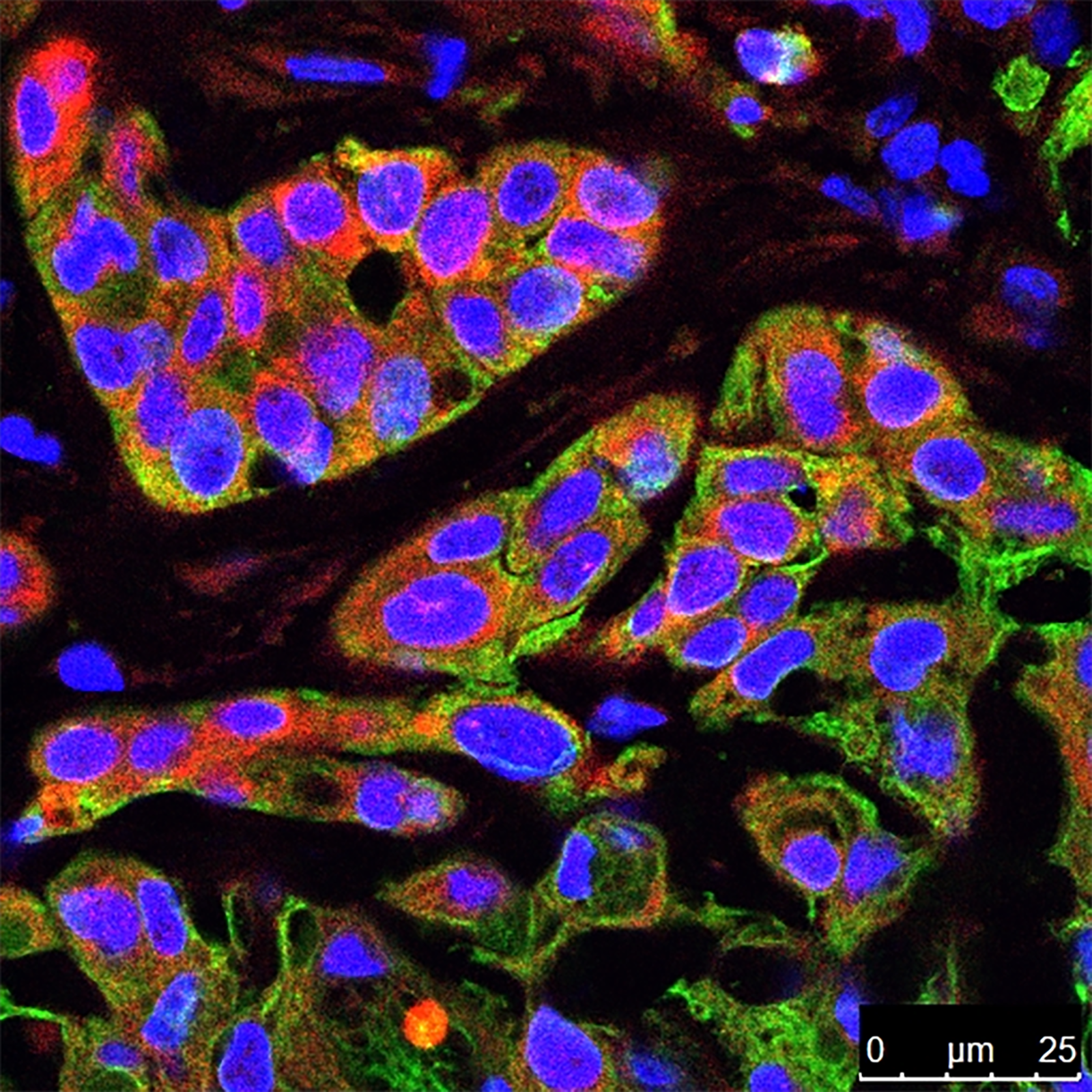

Supplement: Supplementary file 9 — Source data Fig. 8 [file 44319_2024_132_MOESM9_ESM.zip › Figure 8/8C/Wt/Ago2-Cytokeratin-DAPI.tif]

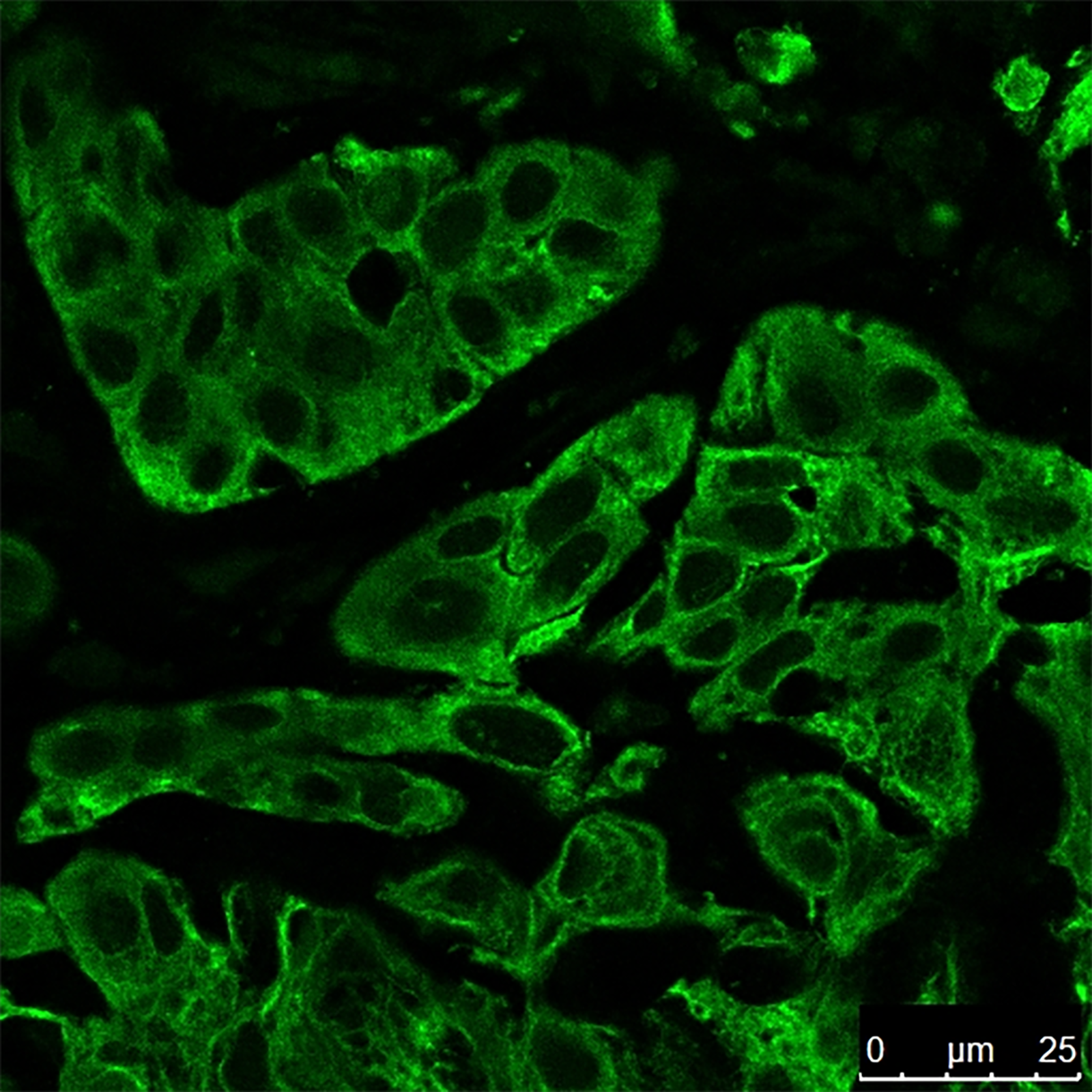

Supplement: Supplementary file 9 — Source data Fig. 8 [file 44319_2024_132_MOESM9_ESM.zip › Figure 8/8C/Wt/Cytokeratin.tif]

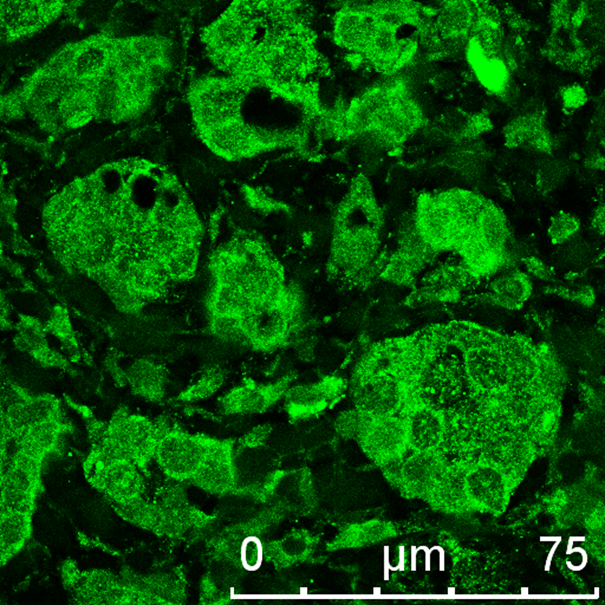

Supplement: Supplementary file 9 — Source data Fig. 8 [file 44319_2024_132_MOESM9_ESM.zip › Figure 8/8D/10482/Metastasis Ago2.tif]

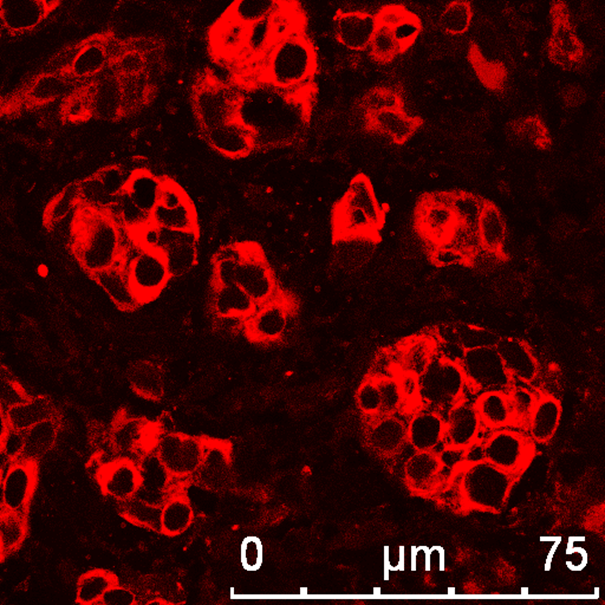

Supplement: Supplementary file 9 — Source data Fig. 8 [file 44319_2024_132_MOESM9_ESM.zip › Figure 8/8D/10482/Metastasis Cytokeratin.tif]

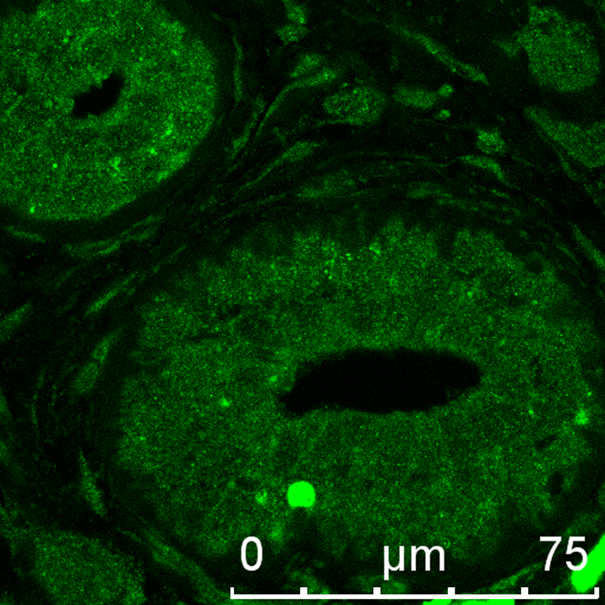

Supplement: Supplementary file 9 — Source data Fig. 8 [file 44319_2024_132_MOESM9_ESM.zip › Figure 8/8D/10482/Primary tumor Ago2.tif]

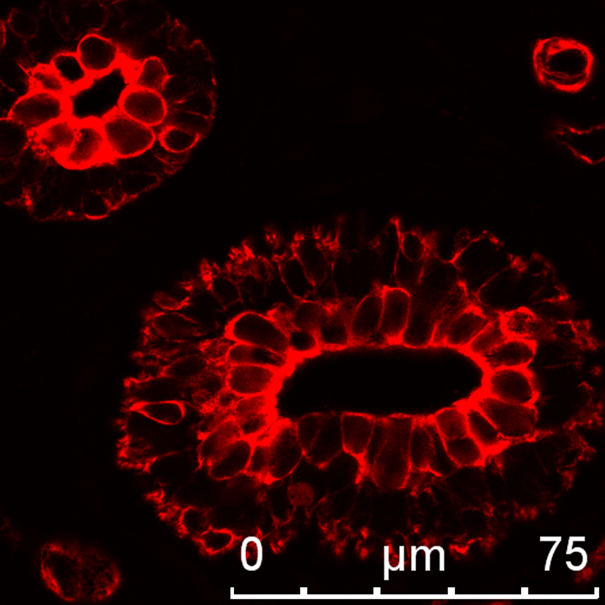

Supplement: Supplementary file 9 — Source data Fig. 8 [file 44319_2024_132_MOESM9_ESM.zip › Figure 8/8D/10482/Primary tumor Cytokeratin.tif]

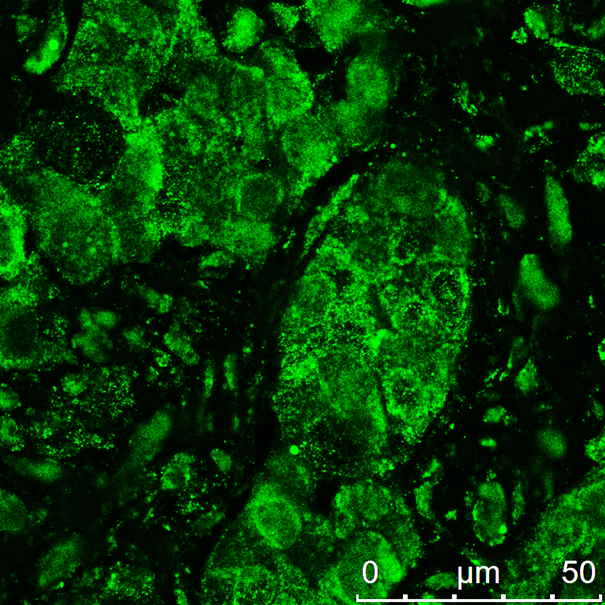

Supplement: Supplementary file 9 — Source data Fig. 8 [file 44319_2024_132_MOESM9_ESM.zip › Figure 8/8D/10549/Metastasis Ago2.tif]

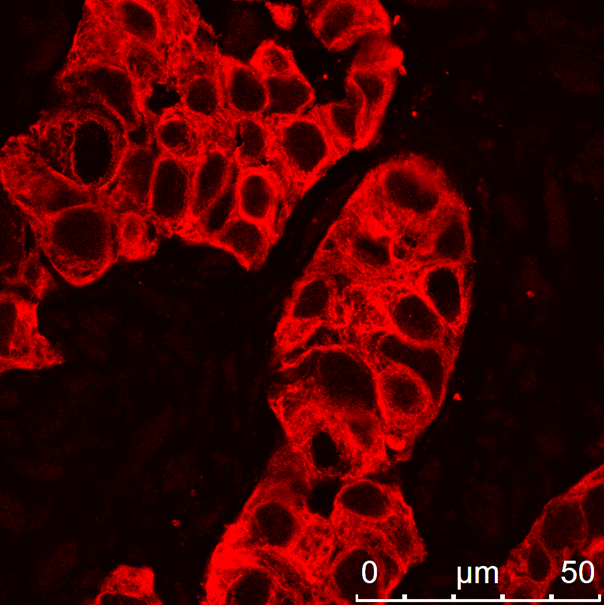

Supplement: Supplementary file 9 — Source data Fig. 8 [file 44319_2024_132_MOESM9_ESM.zip › Figure 8/8D/10549/Metastasis Cytokeratin.tif]

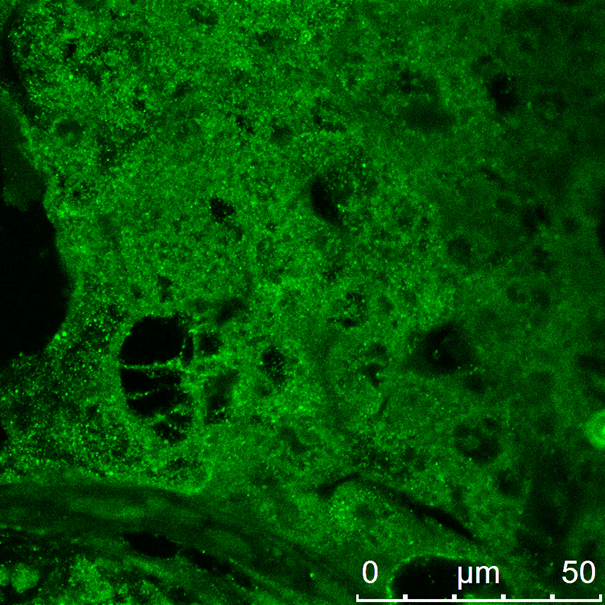

Supplement: Supplementary file 9 — Source data Fig. 8 [file 44319_2024_132_MOESM9_ESM.zip › Figure 8/8D/10549/Primary tumer Ago2.tif]

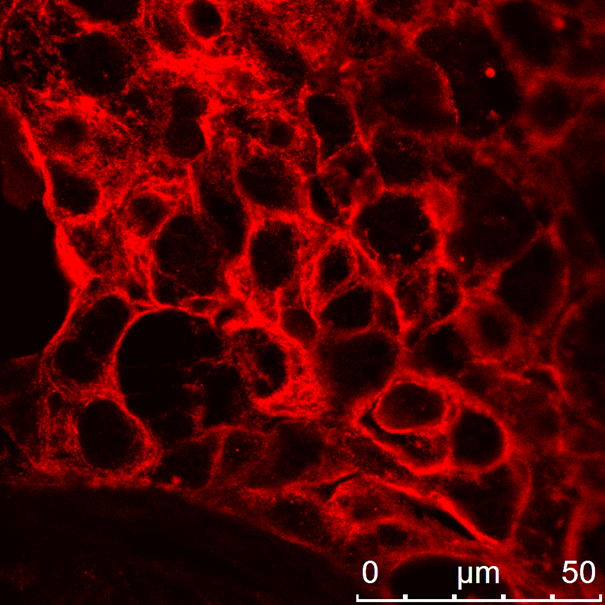

Supplement: Supplementary file 9 — Source data Fig. 8 [file 44319_2024_132_MOESM9_ESM.zip › Figure 8/8D/10549/Primary tumer Cytokeratin.tif]

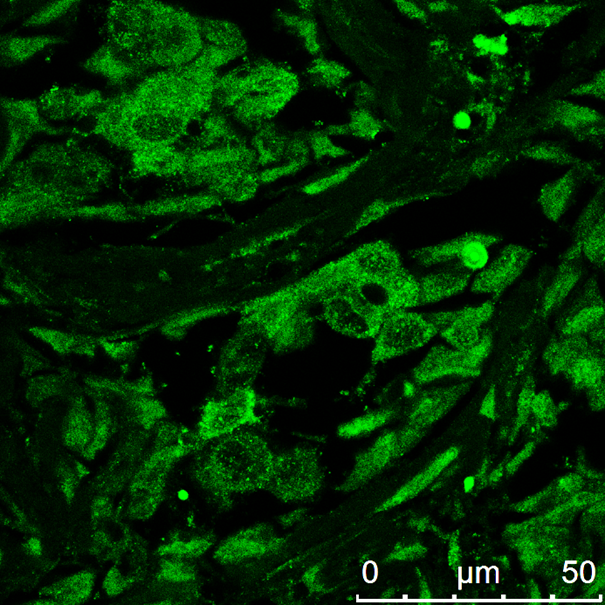

Supplement: Supplementary file 9 — Source data Fig. 8 [file 44319_2024_132_MOESM9_ESM.zip › Figure 8/8D/7194/Metastasis Ago2.tif]

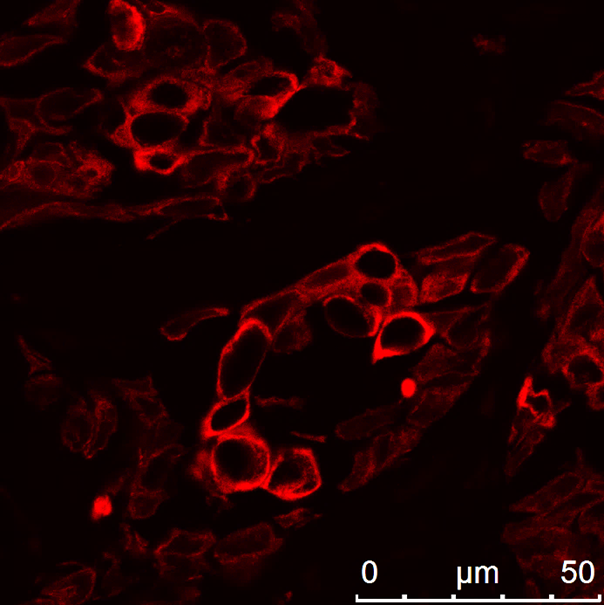

Supplement: Supplementary file 9 — Source data Fig. 8 [file 44319_2024_132_MOESM9_ESM.zip › Figure 8/8D/7194/Metastasis Cytokeratin.tif]

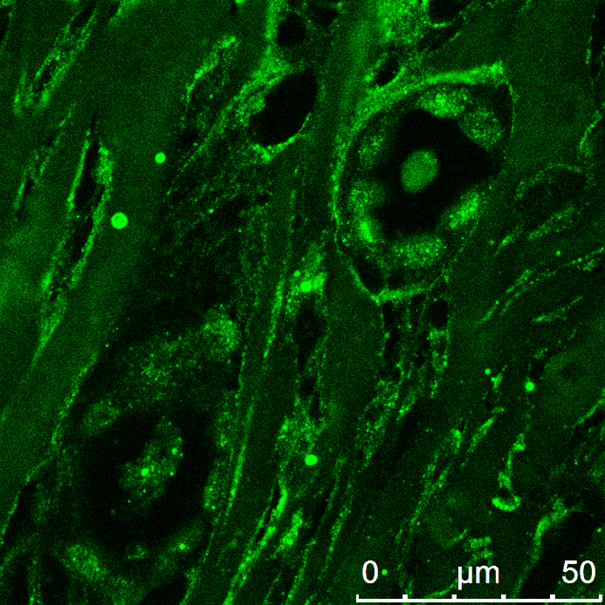

Supplement: Supplementary file 9 — Source data Fig. 8 [file 44319_2024_132_MOESM9_ESM.zip › Figure 8/8D/7194/Primary tumer Ago2.tif]

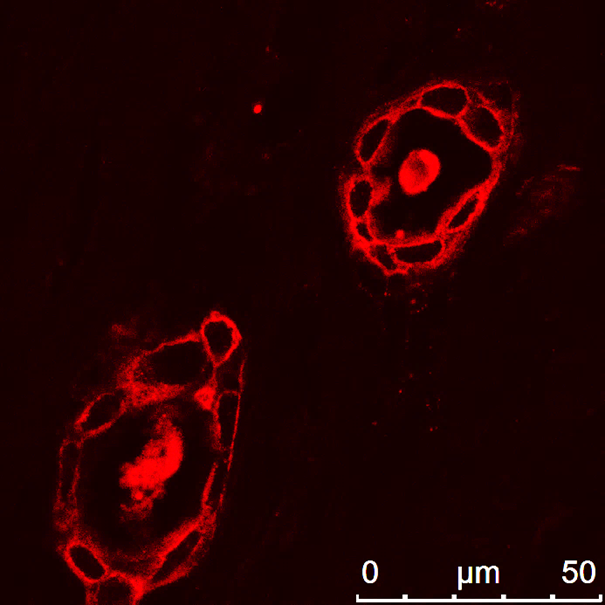

Supplement: Supplementary file 9 — Source data Fig. 8 [file 44319_2024_132_MOESM9_ESM.zip › Figure 8/8D/7194/Primary tumer Cytokeratin.tif]

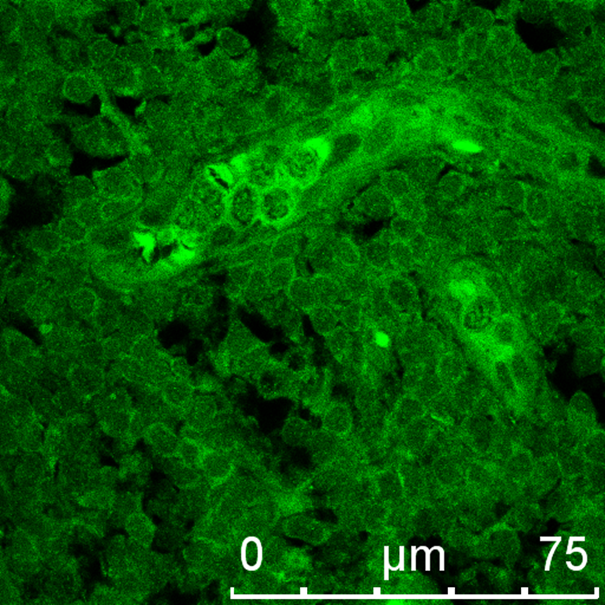

Supplement: Supplementary file 9 — Source data Fig. 8 [file 44319_2024_132_MOESM9_ESM.zip › Figure 8/8D/7512/Metastasis Ago2.tif]

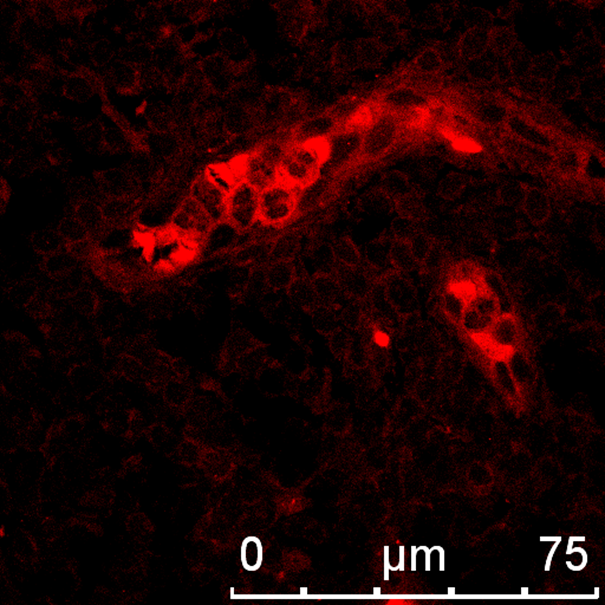

Supplement: Supplementary file 9 — Source data Fig. 8 [file 44319_2024_132_MOESM9_ESM.zip › Figure 8/8D/7512/Metastasis Cytokeratin.tif]

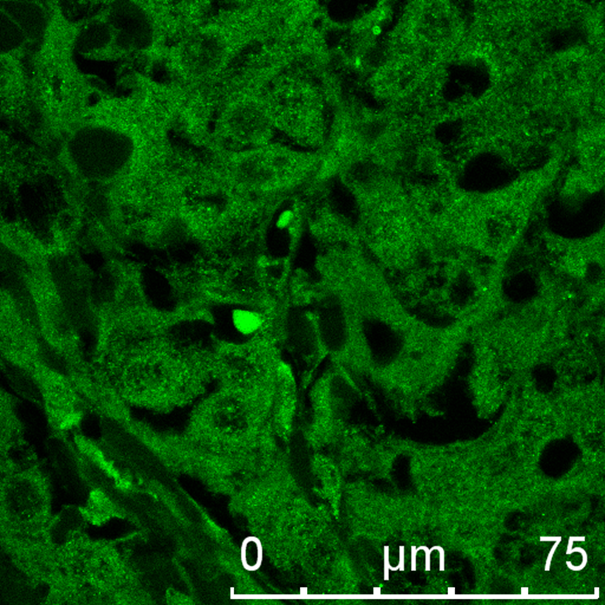

Supplement: Supplementary file 9 — Source data Fig. 8 [file 44319_2024_132_MOESM9_ESM.zip › Figure 8/8D/7512/Primary tumer Ago2.tif]

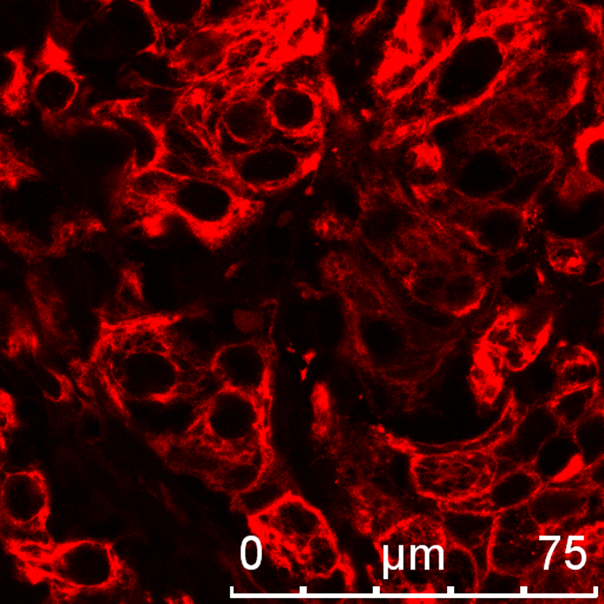

Supplement: Supplementary file 9 — Source data Fig. 8 [file 44319_2024_132_MOESM9_ESM.zip › Figure 8/8D/7512/Primary tumer Cytokeratin.tif]

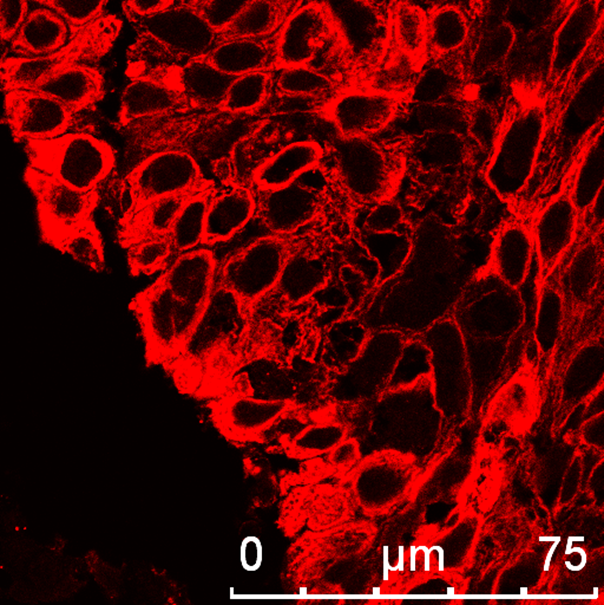

Supplement: Supplementary file 9 — Source data Fig. 8 [file 44319_2024_132_MOESM9_ESM.zip › Figure 8/8D/8364/Metastasis Cytokaretin.tif]

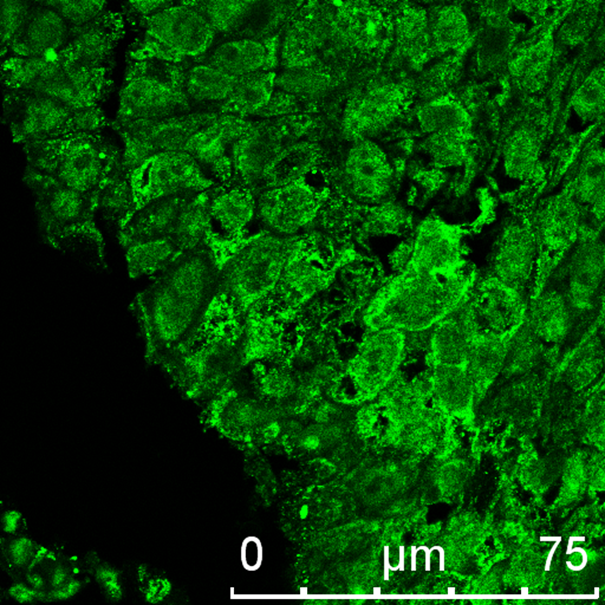

Supplement: Supplementary file 9 — Source data Fig. 8 [file 44319_2024_132_MOESM9_ESM.zip › Figure 8/8D/8364/Metastasis Ago2.tif]

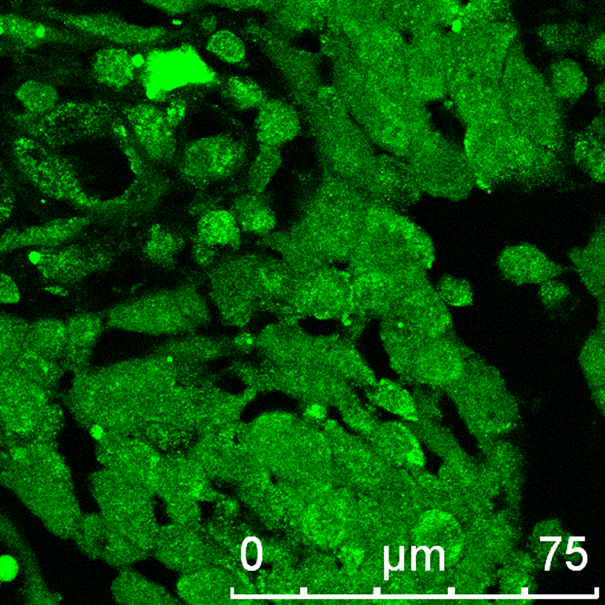

Supplement: Supplementary file 9 — Source data Fig. 8 [file 44319_2024_132_MOESM9_ESM.zip › Figure 8/8D/8364/Primary tumer Ago2.tif]

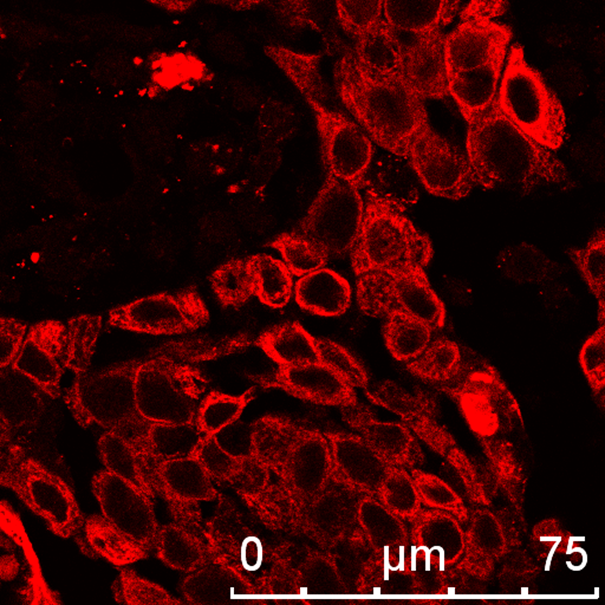

Supplement: Supplementary file 9 — Source data Fig. 8 [file 44319_2024_132_MOESM9_ESM.zip › Figure 8/8D/8364/Primary tumor Cytokaretin.tif]

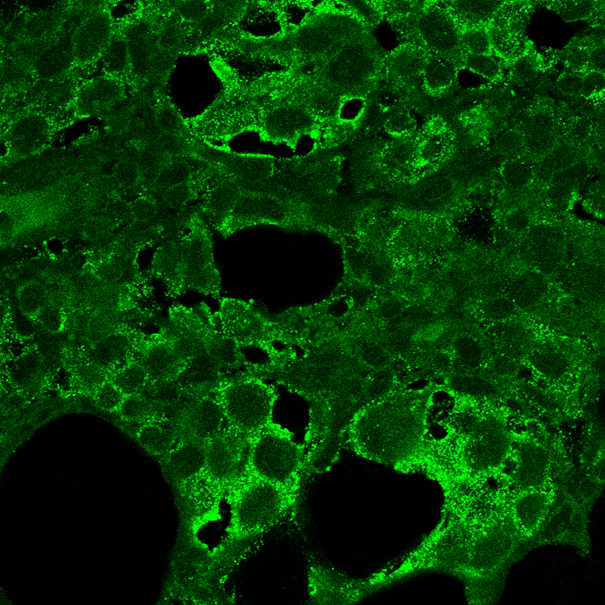

Supplement: Supplementary file 9 — Source data Fig. 8 [file 44319_2024_132_MOESM9_ESM.zip › Figure 8/8D/8549/metastasis Ago2.tif]

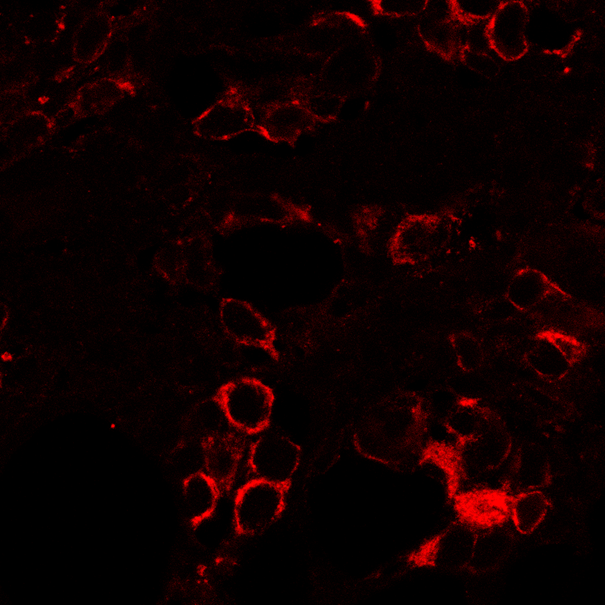

Supplement: Supplementary file 9 — Source data Fig. 8 [file 44319_2024_132_MOESM9_ESM.zip › Figure 8/8D/8549/Metastasis cytokeratin.tif]

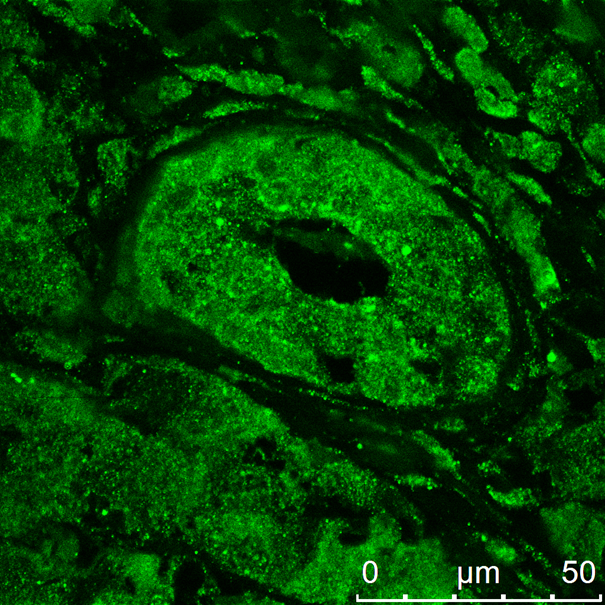

Supplement: Supplementary file 9 — Source data Fig. 8 [file 44319_2024_132_MOESM9_ESM.zip › Figure 8/8D/8549/Primary tumer Ago2.tif]

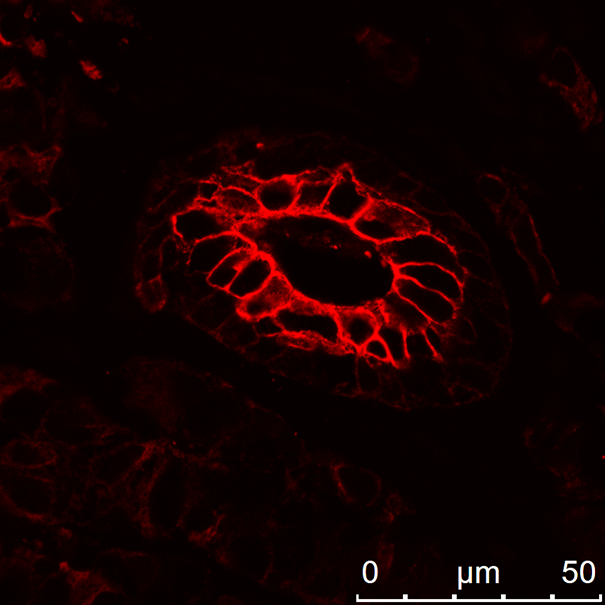

Supplement: Supplementary file 9 — Source data Fig. 8 [file 44319_2024_132_MOESM9_ESM.zip › Figure 8/8D/8549/Primary tumer cytokeratin.tif]

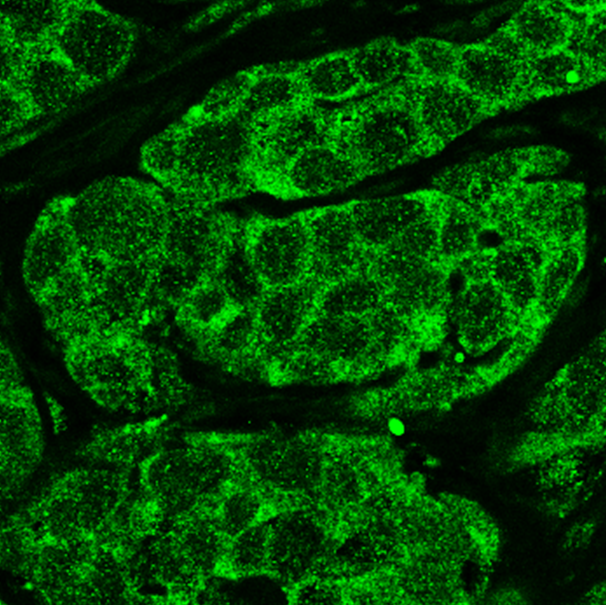

Supplement: Supplementary file 9 — Source data Fig. 8 [file 44319_2024_132_MOESM9_ESM.zip › Figure 8/8D/9455/Metastasis Ago2.tif]

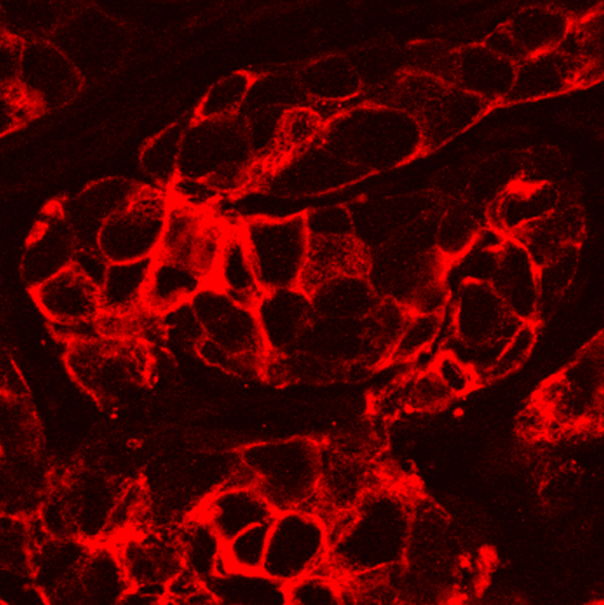

Supplement: Supplementary file 9 — Source data Fig. 8 [file 44319_2024_132_MOESM9_ESM.zip › Figure 8/8D/9455/Metastasis Cytokeratin.tif]

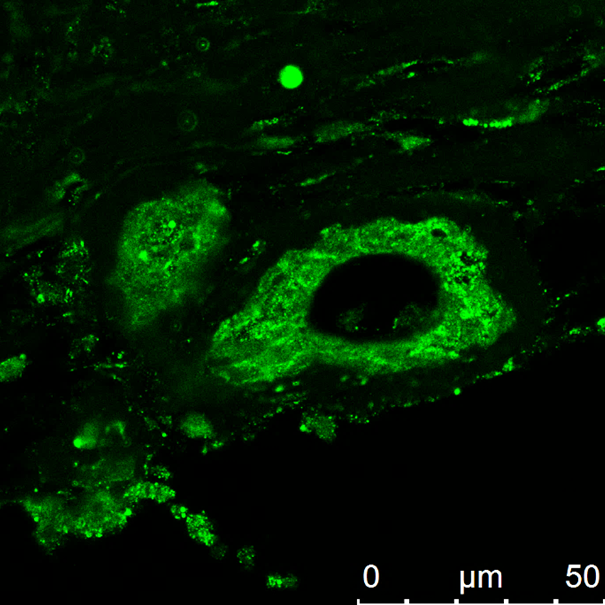

Supplement: Supplementary file 9 — Source data Fig. 8 [file 44319_2024_132_MOESM9_ESM.zip › Figure 8/8D/9455/Primary tumer Ago2.tif]

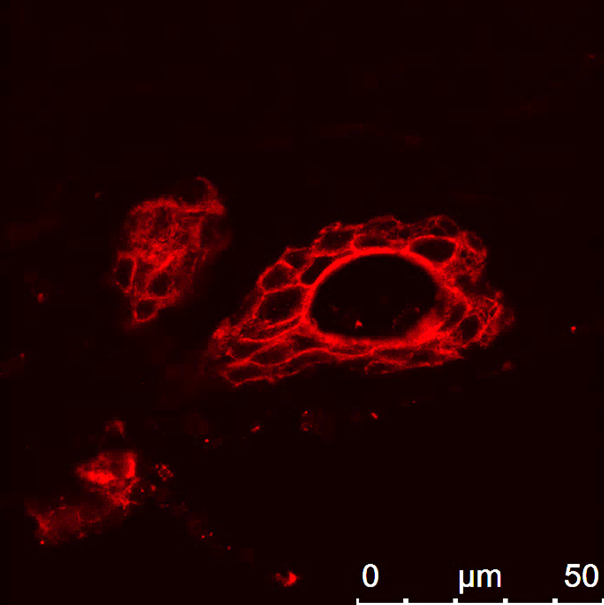

Supplement: Supplementary file 9 — Source data Fig. 8 [file 44319_2024_132_MOESM9_ESM.zip › Figure 8/8D/9455/Primary tumer Cytokeratin.tif]

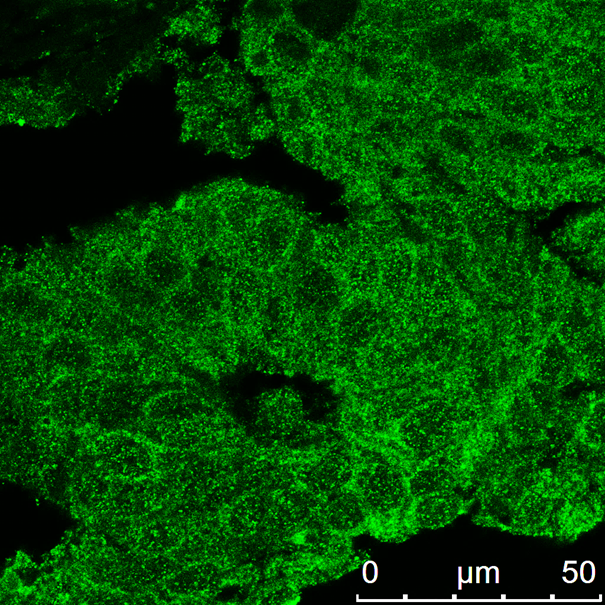

Supplement: Supplementary file 9 — Source data Fig. 8 [file 44319_2024_132_MOESM9_ESM.zip › Figure 8/8D/9456/Metastasis Ago2.tif]

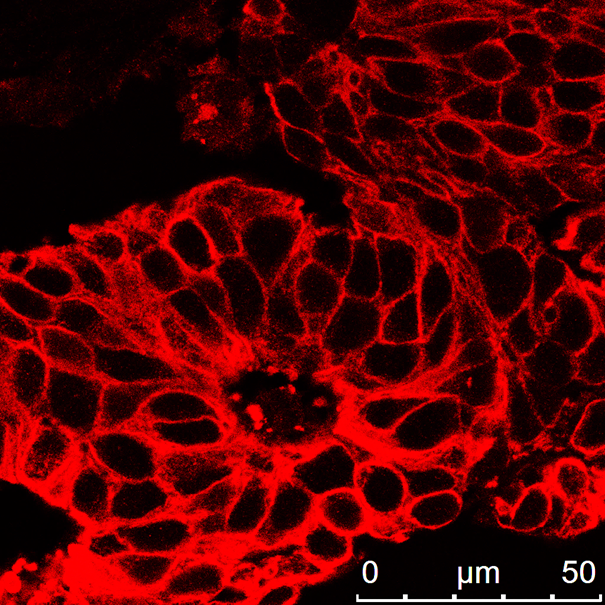

Supplement: Supplementary file 9 — Source data Fig. 8 [file 44319_2024_132_MOESM9_ESM.zip › Figure 8/8D/9456/Metastasis Cytokeratin.tif]

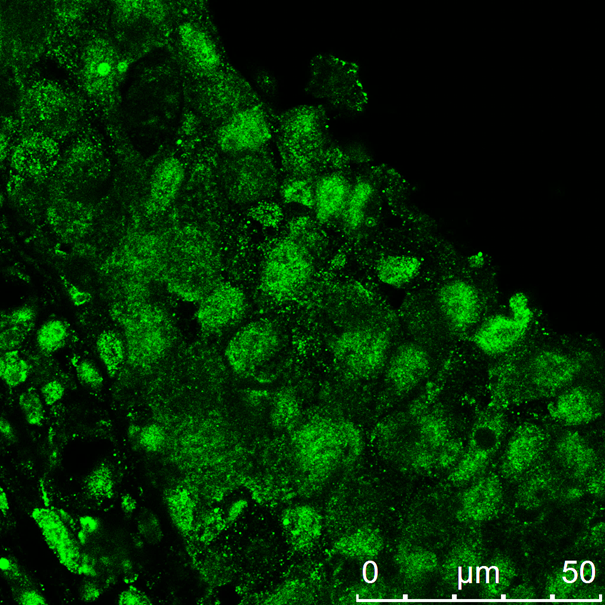

Supplement: Supplementary file 9 — Source data Fig. 8 [file 44319_2024_132_MOESM9_ESM.zip › Figure 8/8D/9456/Primary tumer Ago2.tif]

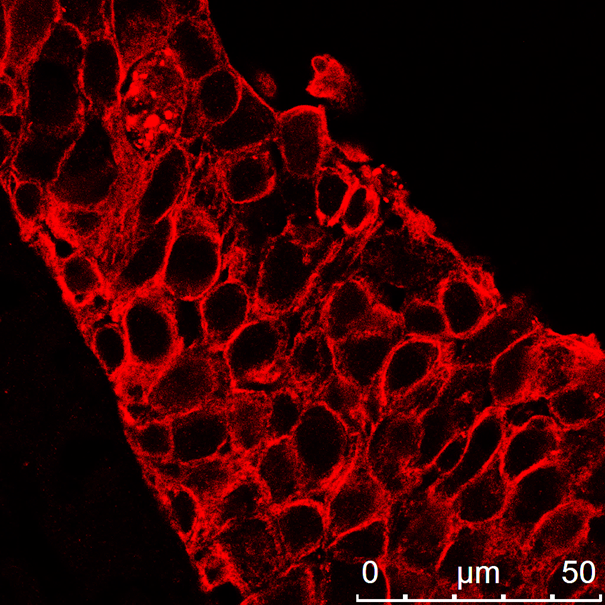

Supplement: Supplementary file 9 — Source data Fig. 8 [file 44319_2024_132_MOESM9_ESM.zip › Figure 8/8D/9456/Primary tumer Cytokeratin.tif]

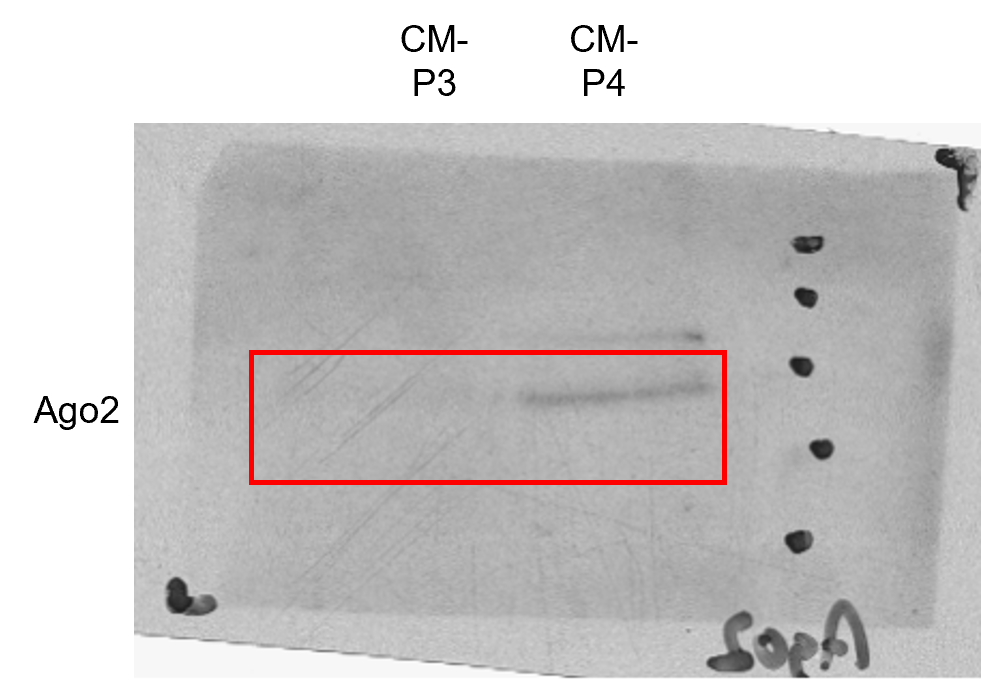

Supplement: Supplementary file 10 — Source data Fig. 9 [file 44319_2024_132_MOESM10_ESM.zip › Figure 9/9A/western Ago2.tif]

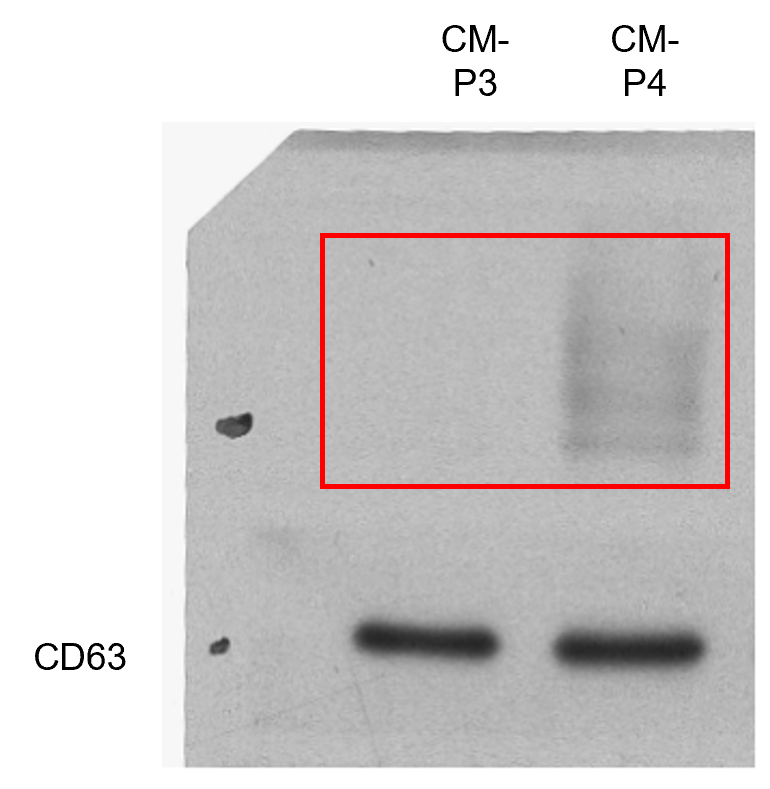

Supplement: Supplementary file 10 — Source data Fig. 9 [file 44319_2024_132_MOESM10_ESM.zip › Figure 9/9A/western CD63.tif]

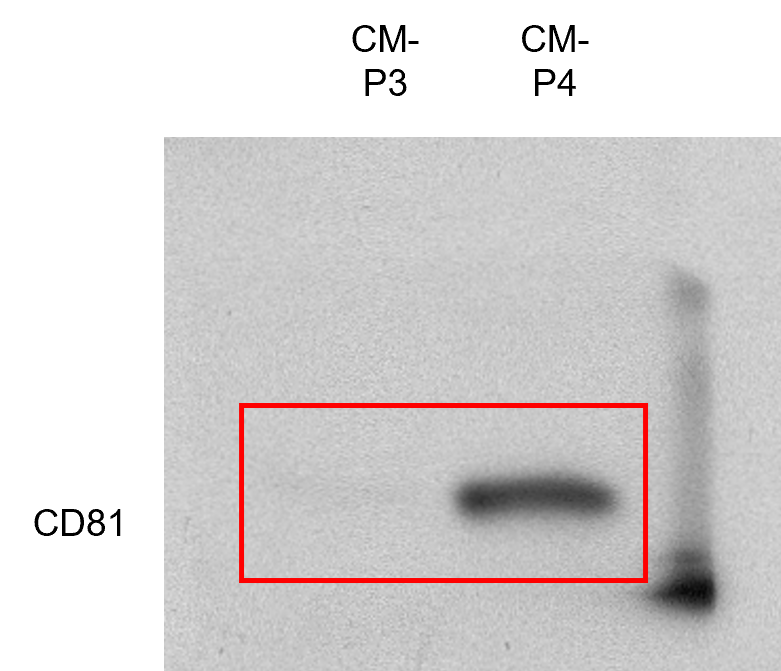

Supplement: Supplementary file 10 — Source data Fig. 9 [file 44319_2024_132_MOESM10_ESM.zip › Figure 9/9A/western CD81.tif]

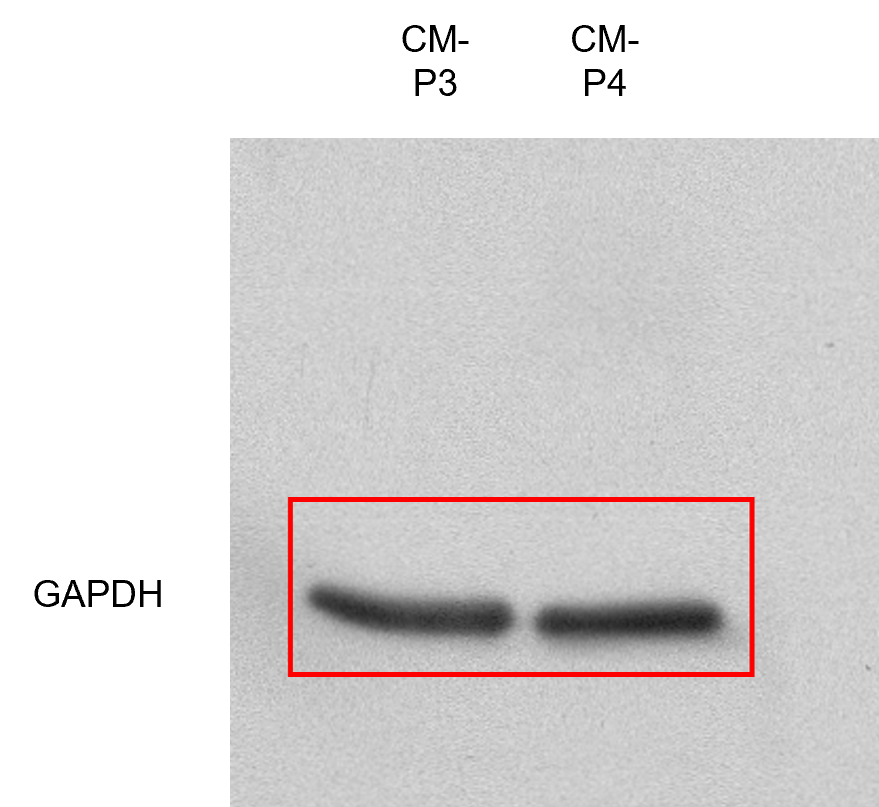

Supplement: Supplementary file 10 — Source data Fig. 9 [file 44319_2024_132_MOESM10_ESM.zip › Figure 9/9A/western GAPDH.tif]

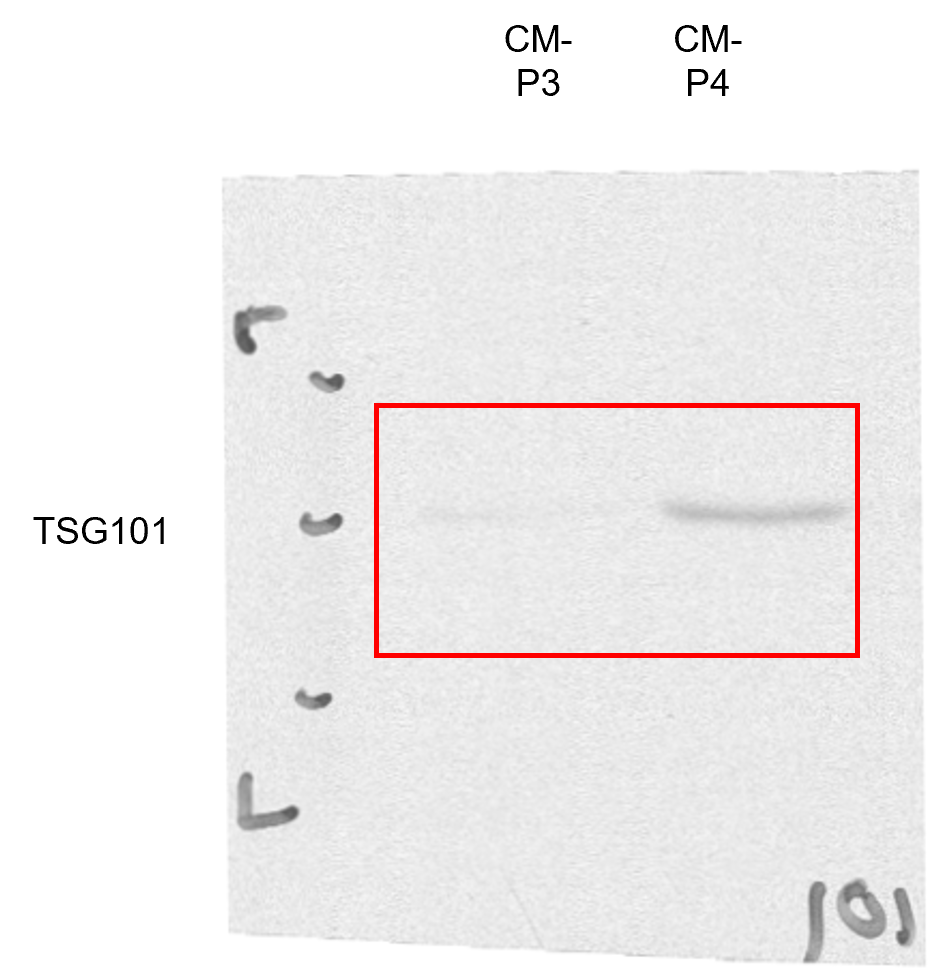

Supplement: Supplementary file 10 — Source data Fig. 9 [file 44319_2024_132_MOESM10_ESM.zip › Figure 9/9A/western TSG101.tif]

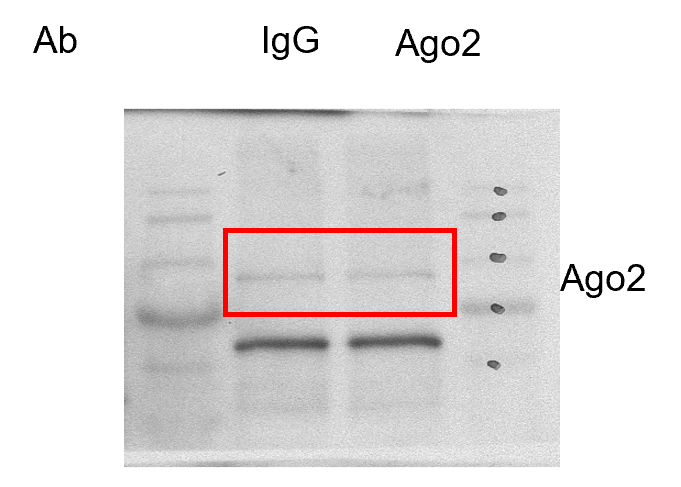

Supplement: Supplementary file 10 — Source data Fig. 9 [file 44319_2024_132_MOESM10_ESM.zip › Figure 9/9B/western input Ago2.tif]

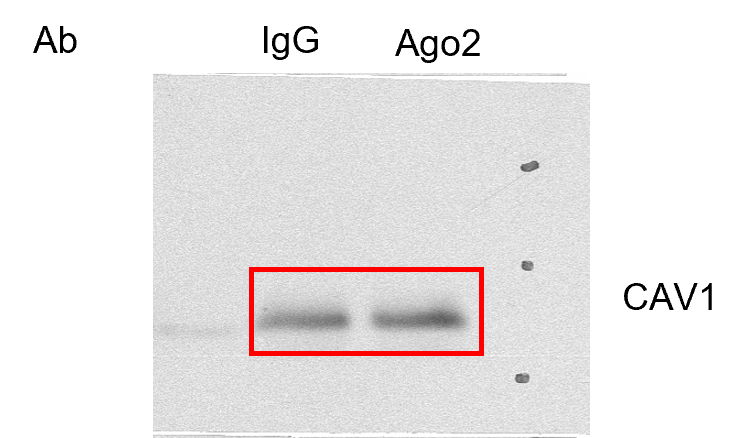

Supplement: Supplementary file 10 — Source data Fig. 9 [file 44319_2024_132_MOESM10_ESM.zip › Figure 9/9B/western input CAV1.tif]

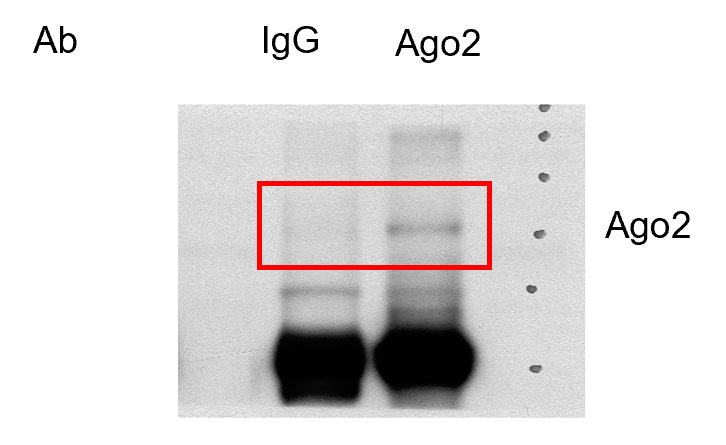

Supplement: Supplementary file 10 — Source data Fig. 9 [file 44319_2024_132_MOESM10_ESM.zip › Figure 9/9B/western IP Ago2.tif]

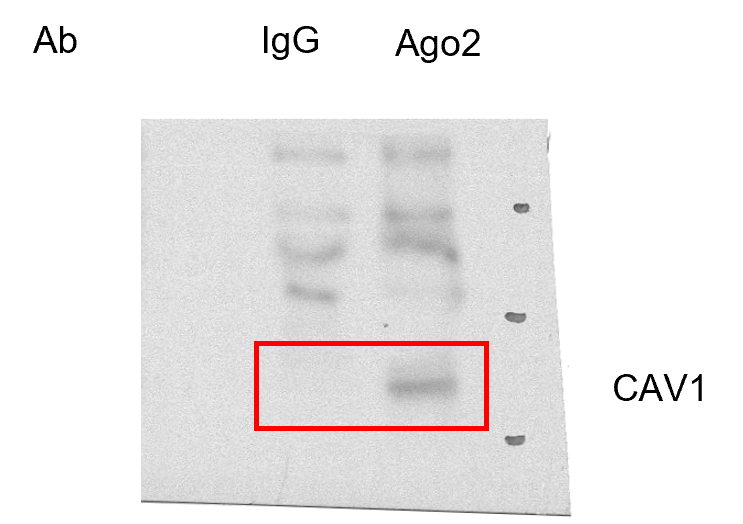

Supplement: Supplementary file 10 — Source data Fig. 9 [file 44319_2024_132_MOESM10_ESM.zip › Figure 9/9B/western IP CAV1.tif]

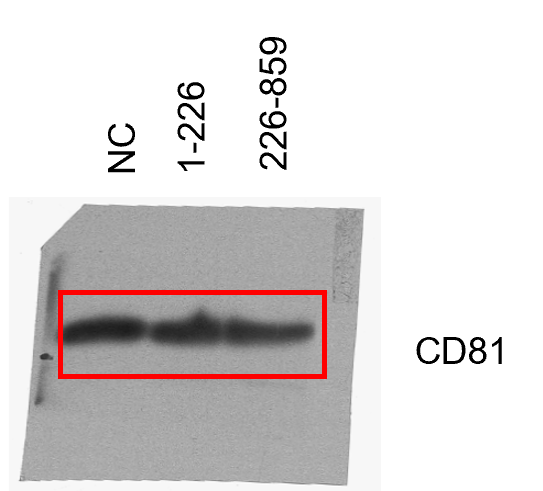

Supplement: Supplementary file 10 — Source data Fig. 9 [file 44319_2024_132_MOESM10_ESM.zip › Figure 9/9C/western exosome CD81.tif]

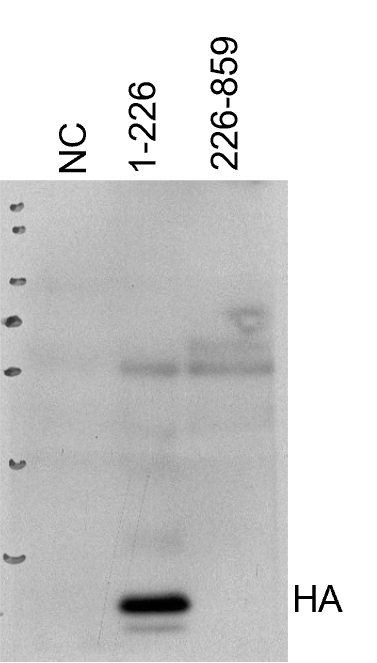

Supplement: Supplementary file 10 — Source data Fig. 9 [file 44319_2024_132_MOESM10_ESM.zip › Figure 9/9C/western exosome HA.tif]

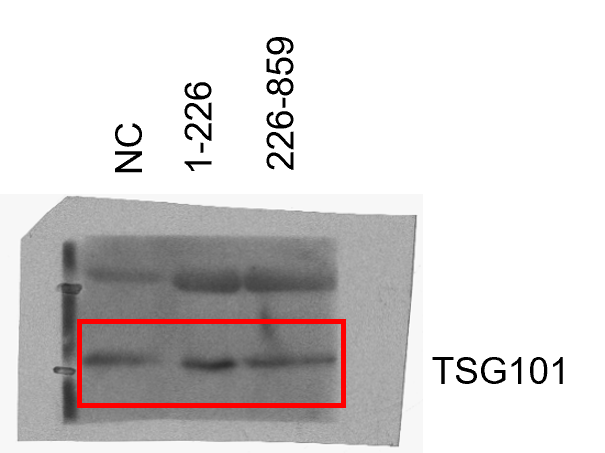

Supplement: Supplementary file 10 — Source data Fig. 9 [file 44319_2024_132_MOESM10_ESM.zip › Figure 9/9C/western exosome TSG101.tif]

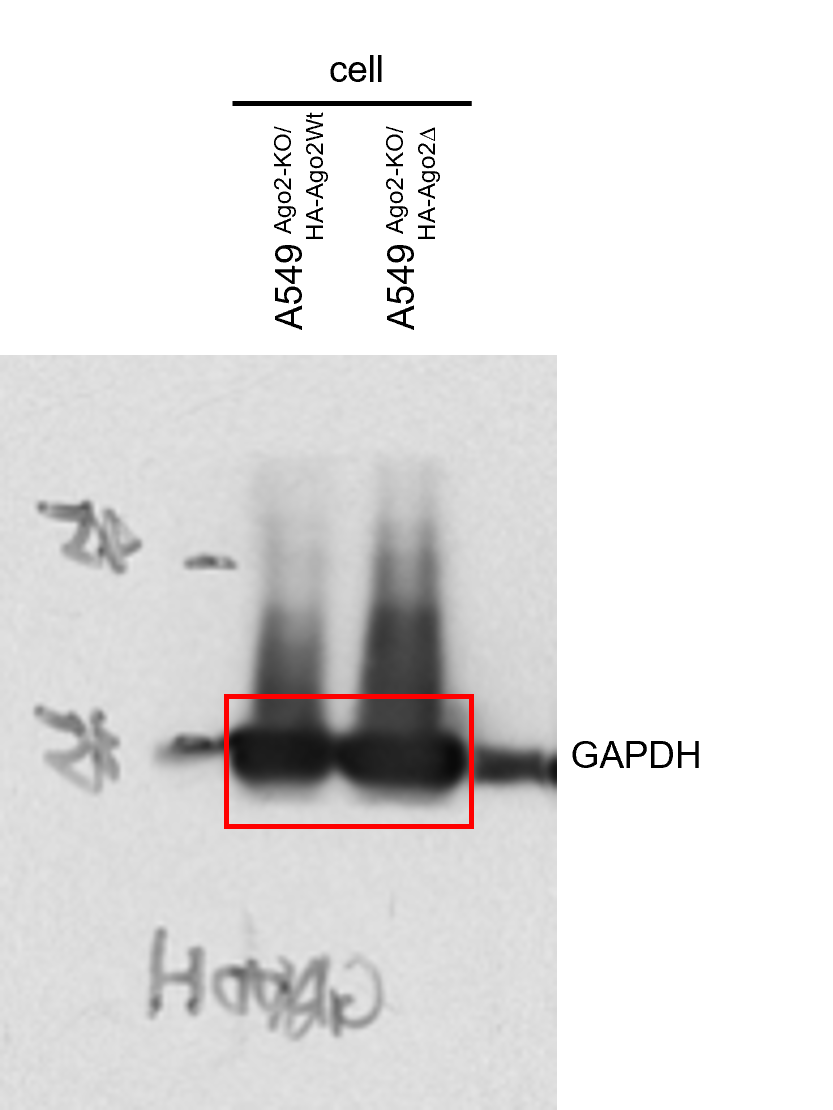

Supplement: Supplementary file 10 — Source data Fig. 9 [file 44319_2024_132_MOESM10_ESM.zip › Figure 9/9D/western cell GAPDH.tif]

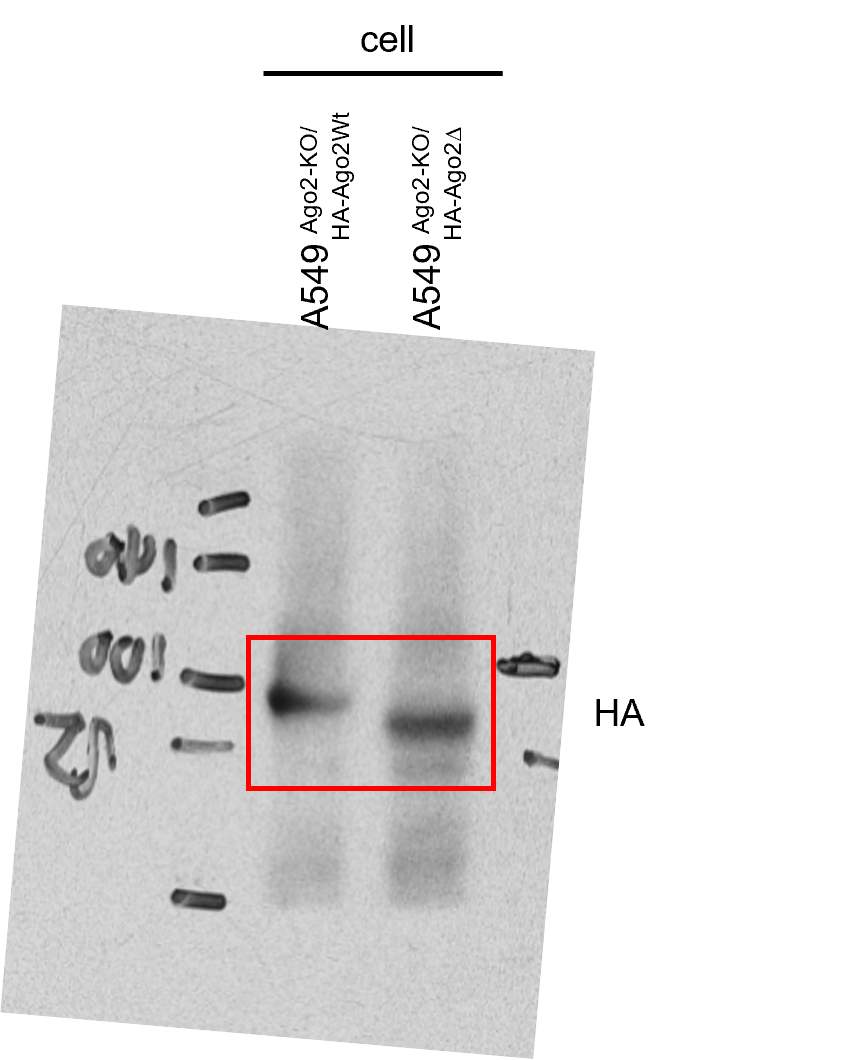

Supplement: Supplementary file 10 — Source data Fig. 9 [file 44319_2024_132_MOESM10_ESM.zip › Figure 9/9D/western cell HA-Ago2.tif]

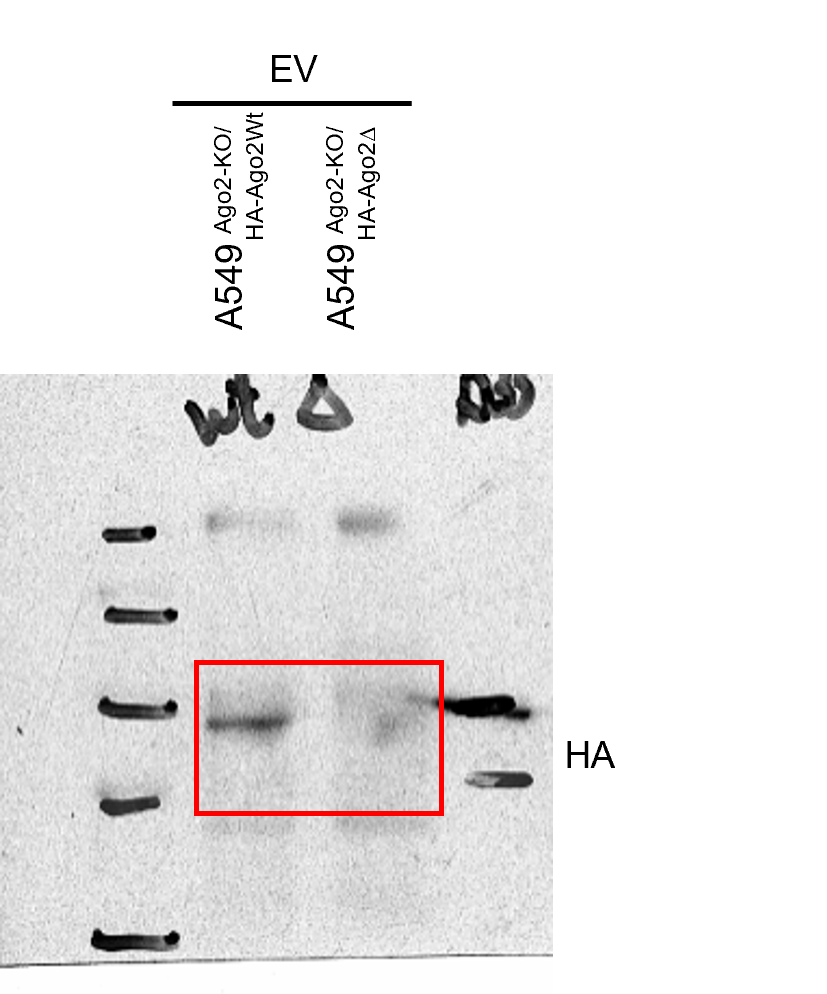

Supplement: Supplementary file 10 — Source data Fig. 9 [file 44319_2024_132_MOESM10_ESM.zip › Figure 9/9D/western EV HA-Ago2.tif]

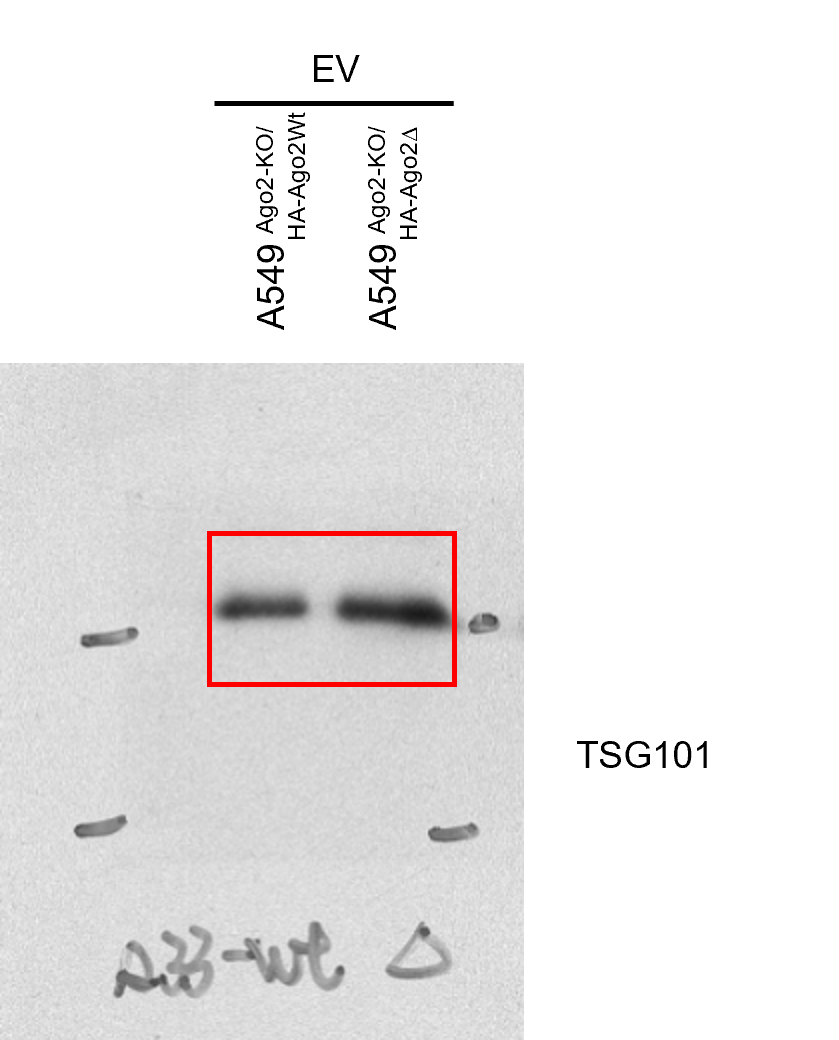

Supplement: Supplementary file 10 — Source data Fig. 9 [file 44319_2024_132_MOESM10_ESM.zip › Figure 9/9D/western EV TSG101.tif]
