# Supplementary material for: Ago2/CAV1 interaction potentiates metastasis via controlling Ago2 localization and miRNA action
Source: EMBO Rep. 2024 Apr 22;25(5):20. doi: 10.1038/s44319-024-00132-7 (PMC11094075; doi:10.1038/s44319-024-00132-7)
Supplement: Supplementary file 12 — Expanded View Figures [file 44319_2024_132_MOESM12_ESM.pdf]

Expanded View Figures

Figure EV1. Mapping the interacting domains of Ago2 and CAV1.

(**A**) The quantitation of Western blots of experiments in Fig. 1A. Each spot describes the ratio of coprecipitated CAV1 (pCAV1) to precipitated Ago2 (pAgo2) in the sample, relative to that of the cancer cell sample in each replicated experiment. Bars are means ± standard error of the mean (SEM, $n = 3$–6, biological replicates). Student's t-test, $^{**}P \leq 0.01$; $^{****}P \leq 0.0001$. (**B**) Direct interaction of Ago2 with CAV1 in vitro. In the mixture of Ago2-His and CAV1-His, Ago2-His was immunoprecipitated with anti-Ago2 antibodies, and coprecipitation of CAV1 was analyzed. The mixtures contain CAV1-His only, or IgG serves as negative controls. (**C**) The quantitation of Western blots of experiments in Fig. 1B. Each spot describes the ratio of coprecipitated CAV1 (pCAV1) to input CAV1 (inCAV1) in the sample, relative to that of the mixture of Ago2-His and CAV1-His (+Ago2-His) in each replicated experiment. Bars are means ± SEM ($n = 3$, biological replicates). Student's t-test, $^{****}P \leq 0.0001$. (**D**) The quantitation of Western blots of experiments in Fig. 1C. Each spot describes the ratio of coprecipitated CAV1 (pCAV1) to precipitated Ago2 (pAgo2), relative to that of the mixture with full-length HA-Ago2 (Full) in each replicated experiment. Bars are means ± SEM ($n = 4$, biological replicates). Student's t-test, $^{**}P \leq 0.01$; $^{***}P \leq 0.001$; $^{****}P \leq 0.0001$. (**E**) The quantitation of Western blots of experiments in Fig. 1D. Each spot describes the ratio of coprecipitated CAV1 (pCAV1) to precipitated Ago2 (pAgo2), relative to that of the mixture with His-CAV1(1-102)-mRuby. $n = 1$. (**F**) The quantitation of Western blots of experiments in Fig. 1E. Each spot describes the ratio of coprecipitated CAV1 (pCAV1) to precipitated Ago2 (pAgo2), relative to that of the mixture with wild-type CAV1-Flag (Wt) in each replicated experiment. Bars are means ± SEM ($n = 3$, biological replicates). Student's t-test, $^{****}P \leq 0.0001$. (**G**) Schematic of human Ago2 deletion constructs. The full-length (Full) construct contains four characteristic domains, namely N-terminal (N), PAZ, MID, and C-terminal PIWI domains, where PAZ is an RNA-binding module and MID and PIWI are catalytic activity domains. (**H**) A series of HA-tagged human Ago2 (HA-Ago2), including C-terminal-deleted, N-terminal-deleted, and serine 387-substituted Ago2, coexpressed with Flag-labeled human CAV1 (CAV1-Flag) in HEK293 cells. Panel i: HA-Ago2 was immunoprecipitated with anti-HA antibodies, and coprecipitation of CAV1-Flag was analyzed using anti-Flag antibodies. Panel ii: the quantitation of Western blots of experiments in panel i. Each spot describes the ratio of coprecipitated CAV1 (pCAV1) to precipitated Ago2 (pAgo2), relative to that of the mixture with full-length HA-Ago2 (Full) in each replicated experiment. Bars are means ± SEM ($n = 2$). (**I**) The quantitation of Western blots of experiments in Fig. 1F. Each spot describes the ratio of coprecipitated CAV1 (pCAV1) to precipitated Ago2 (pAgo2), relative to that of the mixture with full-length HA-Ago2 (Full) in each replicated experiment. Bars are means ± SEM ($n = 3$, biological replicates). Student's t-test, $^{*}P \leq 0.05$. (**J**) The quantitation of Western blots of experiments in Fig. 1G. Each spot describes the ratio of coprecipitated CAV1 (pCAV1) to precipitated Ago2 (pAgo2), relative to that of the mixture with HA-Ago2(1–226) in each replicated experiment. Bars are means ± SEM ($n = 2$). (**K**) The quantitation of Western blots of experiments in Fig. 1H. Each spot describes the ratio of coprecipitated CAV1 (pCAV1) to precipitated Ago2 (pAgo2), relative to that of the mixture with wild-type HA-Ago2 (Wt) in each replicated experiment. Bars are means ± SEM ($n = 6$, biological replicates). Student's t-test, $^{****}P \leq 0.0001$. (**L**) The quantitation of Western blots of experiments in Fig. 1I. Each spot describes the ratio of coprecipitated CAV1 (pCAV1) to precipitated His-tagged eGFP (peGFP), relative to that of the mixture with eGFP-Ago2(175–226)-His in each replicated experiment. Bars are means ± SEM ($n = 3$, biological replicates). Student's t-test, $^{***}P \leq 0.001$. (**M**) Aromatic amino acids in Ago2 are displayed using Pepinfo. The black bars indicate the positions of aromatic residues among a total of 859 residues in Ago2. The red box indicates the CBM of Ago2 amino acids 199–212. (**N**) Changes in the protein thermodynamic stability ($\Delta\Delta G$) of the indicated amino acid mutations, estimated using the PoPMuSiC algorithm. Aromatic amino acids are marked in red, and lysine is marked in blue. SER: surface exposed ratio. SERs exceeding 45% are indicated by a yellow background, suggesting an exposed amino acid. SERs below 20% are indicated by a gray background, suggesting a buried amino acid. Mutation-inducing protein thermodynamic instability is indicated by a red background. (**O**) Effects of Ago2 CBM mutation on Ago2/CAV1 interaction. In HEK293 cells, wild-type (Wt) or mutated (WF199/200AA, F202A, W211A) HA-Ago2 was coexpressed with CAV1-Flag. Panel i: in HEK293 cells, HA-Ago2 was immunoprecipitated with anti-HA antibodies, and coprecipitation of CAV1-Flag was analyzed using anti-Flag antibodies. The mixture contains HA-Ago2 serves as a negative control. Panel ii: the quantitation of Western blots of experiments in panel i. Each spot describes the ratio of coprecipitated CAV1 (pCAV1) to precipitated Ago2 (pAgo2), relative to that of the mixture with wild-type HA-Ago2 (Wt) in each replicated experiment. Bars are means ± SEM ($n = 3$, biological replicates). Student's t-test, $^{*}P \leq 0.05$; $^{**}P \leq 0.01$. (**P**) The quantitation of Western blots of experiments in Fig. 1K. Each spot describes the ratio of coprecipitated CAV1 (pCAV1) to input CAV1 (inCAV1) in the sample, relative to that of the mixture with wild-type HA-Ago2 (Wt) in each replicated experiment. Bars are means ± SEM ($n = 3$, biological replicates). Student's t-test, $^{****}P \leq 0.0001$.

 © The Author(s)

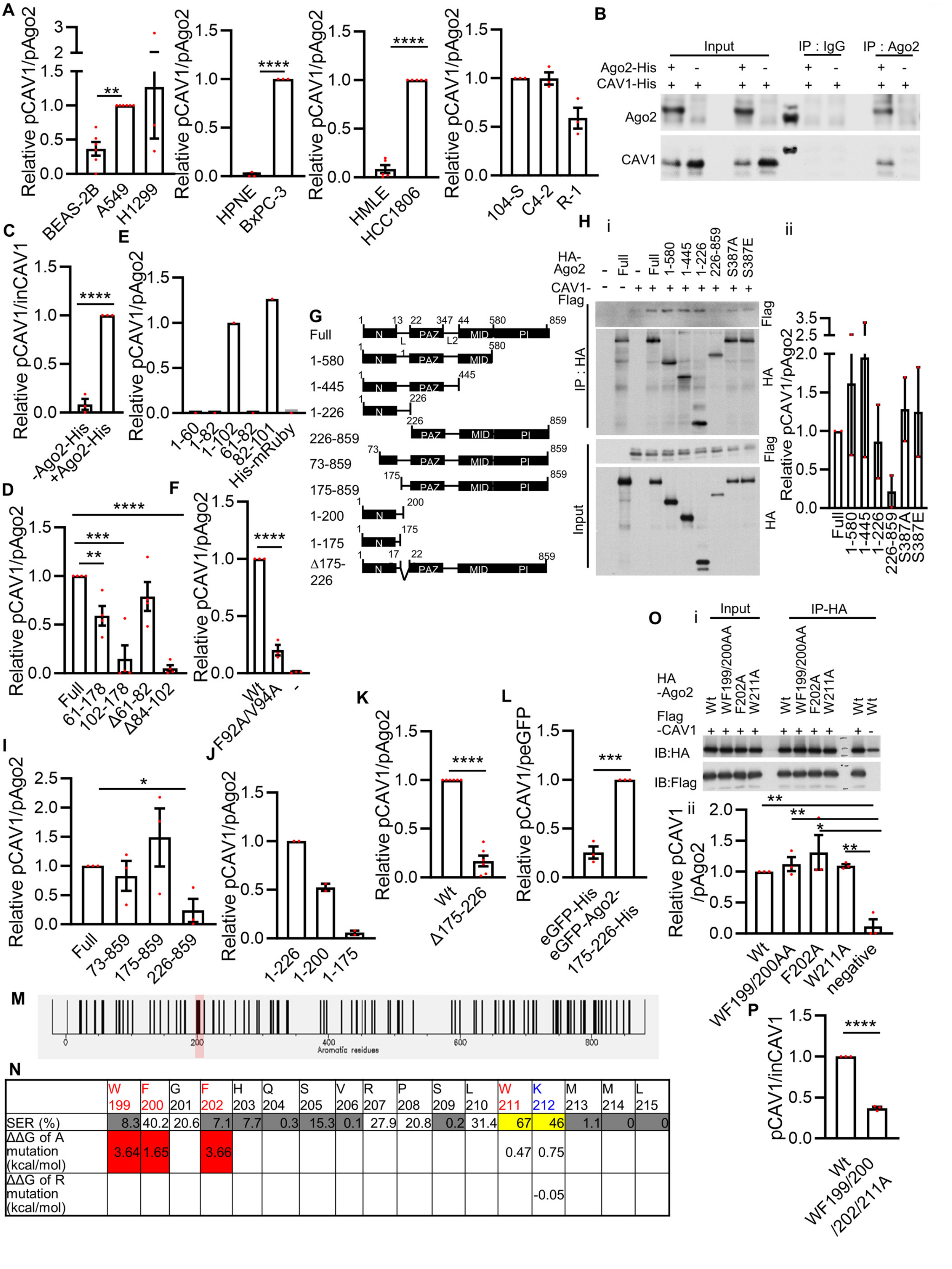

	W 199	F 200	G 201	F 202	H 203	Q 204	S 205	V 206	R 207	P 208	S 209	L 210	W 211	K 212	M 213	M 214	L 215
SER (%)	8.3	40.2	20.6	7.1	7.7	0.3	15.3	0.1	27.9	20.8	0.2	31.4	67	46	1.1	0	0
ΔΔG of A mutation (kcal/mol)	3.64	1.65		3.66									0.47	0.75			
ΔΔG of R mutation (kcal/mol)														-0.05			

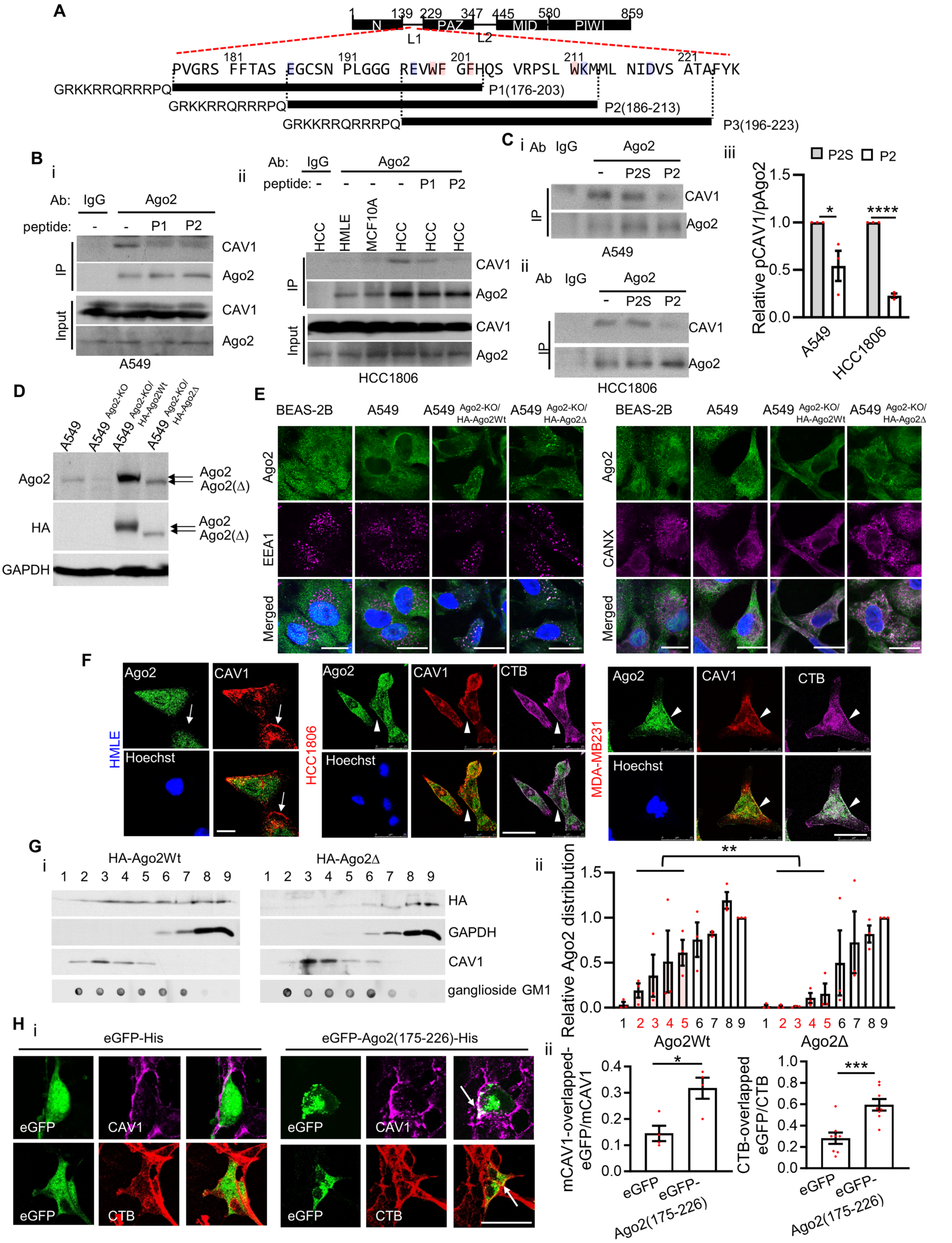
A
1 139 229 347 445 580 859
N PAZ MID PIWI
L1 L2
181 191 201 211 221
PVGRS FFTAS EGCSN PLGGG REVWF GFHQS VRPSL WKMML NIDVS ATAFYK
GRKKRRQRRRPQ P1(176-203)
GRKKRRQRRRPQ P2(186-213)
GRKKRRQRRRPQ P3(196-223)
B i
Ab: IgG Ago2
peptide: - - P1 P2
IP CAV1 Ago2
Input CAV1 Ago2
A549
ii
Ab: IgG Ago2
peptide: - - - - P1 P2
HCC HMLE MCF10A HCC HCC HCC
IP CAV1 Ago2
Input CAV1 Ago2
HCC1806
C i Ab IgG Ago2
- P2S P2
IP CAV1 Ago2
A549
ii Ab IgG Ago2
- P2S P2
IP CAV1 Ago2
HCC1806
iii P2S P2
Relative pCAV1/pAgo2
A549 HCC1806
D
A549 A549 Ago2-KO A549 Ago2-KO/HA-Ago2Wt A549 Ago2-KO/HA-Ago2Δ
Ago2 Ago2 Ago2(Δ)
HA Ago2 Ago2(Δ)
GAPDH
E
BEAS-2B A549 A549 Ago2-KO/HA-Ago2Wt A549 Ago2-KO/HA-Ago2Δ
Ago2 EEA1 Merged
Ago2 CANX Merged
F
HMLE Ago2 CAV1 Hoechst
HCC1806 Ago2 CAV1 CTB Hoechst
MDA-MB231 Ago2 CAV1 CTB Hoechst
G i
HA-Ago2Wt 1 2 3 4 5 6 7 8 9
HA-Ago2Δ 1 2 3 4 5 6 7 8 9
HA GAPDH CAV1 ganglioside GM1
ii
Relative Ago2 distribution
Ago2Wt Ago2Δ
H i
eGFP-His eGFP-Ago2(175-226)-His
eGFP CAV1 eGFP CTB
ii
mCAV1-overlapped-eGFP/mCAV1
CTB-overlapped eGFP/CTB
eGFP eGFP-Ago2(175-226)

Figure EV2. The CBM of Ago2 affects plasma membrane-associated distribution of Ago2.

(**A**) Amino acid sequences of blocking peptides P1, P2, and P3, which are designed in accordance with the amino acid sequence 175–226 of Ago2. Cell-penetrating sequence GRKKRRQRRRPQ was added to the blocking peptides. The red box indicates an aromatic amino acid, and the blue box indicates a charged amino acid.
(**B**) Disruption of Ago2/CAV1 interaction with blocking peptides P1 and P2 was analyzed using co-immunoprecipitation. In A549 (panel i) and HCC1806 (panel ii) cancer cells treated with P1 and P2 peptides, respectively, Ago2 was immunoprecipitated with anti-Ago2 antibodies, and coprecipitation of CAV1 was analyzed. Normal IgG was used as a negative control for precipitation, and HMLE and MCF10A cells were used as negative controls for the interaction. (**C**) Disruption of Ago2/CAV1 interaction by peptides. In A549 (panel i) and HCC1806 (panel ii) cancer cells treated with P2 and P2S peptides, respectively, Ago2 was immunoprecipitated with anti-Ago2 antibodies, and coprecipitation of CAV1 was analyzed. Panel iii: The quantitation of Western blots of experiments in panel i and ii. Each spot describes the ratio of coprecipitated CAV1 (pCAV1) to precipitated Ago2 (pAgo2), relative to that of the sample treated with P2S in each replicated experiment. Bars are means ± SEM ($n = 3$, biological replicates). Student's *t*-test, $^{*}P \le 0.05$; $^{****}P \le 0.0001$. (**D**) Expression of Ago2 in CRISPER/Cas9 gene-edited A549 cancer cells. $A549^{Ago2\text{-}KO}$: A549 cells with Ago2 knocked out by CRISPER/Cas9 gene editing. $A549^{Ago2\text{-}KO/HA\text{-}Ago2Wt}$: Ago2-knockout A549 cells expressing HA-tagged wild-type Ago2. $A549^{Ago2\text{-}KO/HA\text{-}Ago2\Delta}$: Ago2-knockout A549 cells expressing HA-tagged CBM-deleted (amino acids 175–226) Ago2. The expression of Ago2 was analyzed using anti-Ago2 and anti-HA antibodies, with GAPDH used as a loading control. (**E**) Distribution of Ago2 (green), endosome marker EEA1 (purple, left panel), and ER marker CANX (purple, right panel) analyzed using immunofluorescence in normal epithelial cells BEAS-2B and cancer cells A549, $A549^{Ago2\text{-}KO/HA\text{-}Ago2Wt}$ and $A549^{Ago2\text{-}KO/HA\text{-}Ago2\Delta}$. Cell nuclei were stained with Hoechst (blue). Scale bar = 20 μm. (**F**) Distribution of Ago2 (green) and CAV1 (red) was analyzed using immunofluorescence in normal epithelial cells and cancer cells. Lipid rafts were stained with CTB (purple), and cell nuclei were stained with Hoechst (blue). The arrowheads indicate the colocalization of Ago2 and CAV1, and the arrows indicate CAV1 without colocalization with Ago2. Scale bar = 25 μm. (**G**) Western blot analysis of HA-Ago2(Wt), HA-Ago2(Δ175–226), CAV1, and GAPDH in cell membrane fractions enriched in lipid rafts (LRF, fractions 2–5) and non-lipid rafts (non-LRF, fractions 7–9). HA-Ago2(Wt) and HA-Ago2(Δ175–226) were expressed in HEK293 cells. Panel i: proteins were detected in these membrane fractions through Western blotting, with ganglioside-GM1 (CTB dot blotting) and CAV1 being markers of lipid rafts and GAPDH being a marker of non-lipid rafts. Panel ii: The quantitation of Western blots of experiments in panel i. Each spot describes the level of Ago2 in the fraction, relative to that of the 9th fraction in each replicated experiment. Bars are means ± SEM ($n = 3$, biological replicates). Student's *t*-test, $^{**}P \le 0.01$. (**H**) Distribution of eGFP-His, eGFP-Ago2(175–226)-His (green), and CAV1 (purple) in HEK293 cells analyzed using immunofluorescence in panel i. Lipid rafts were stained with CTB (red). The arrows indicate protein colocalization. Scale bar = 25 μm. Panel ii: the quantification of eGFP-His/eGFP-Ago2(175–226)-His association with CAV1 (left) and CTB (right) on the plasma membrane of normal epithelial cells and cancer cells in panel i. Each spot indicates the level of Ago2 overlapping with cell surface CAV1/CTB, normalized with total mCAV1/CTB, respectively. Each condition was quantified from 2 and 4 fields per replicate, 2 replicates per experiment. Bars are means ± SEM ($n = 4$ and 8). Student's *t*-test, $^{*}P \le 0.05$, $^{***}P \le 0.001$.

© The Author(s)

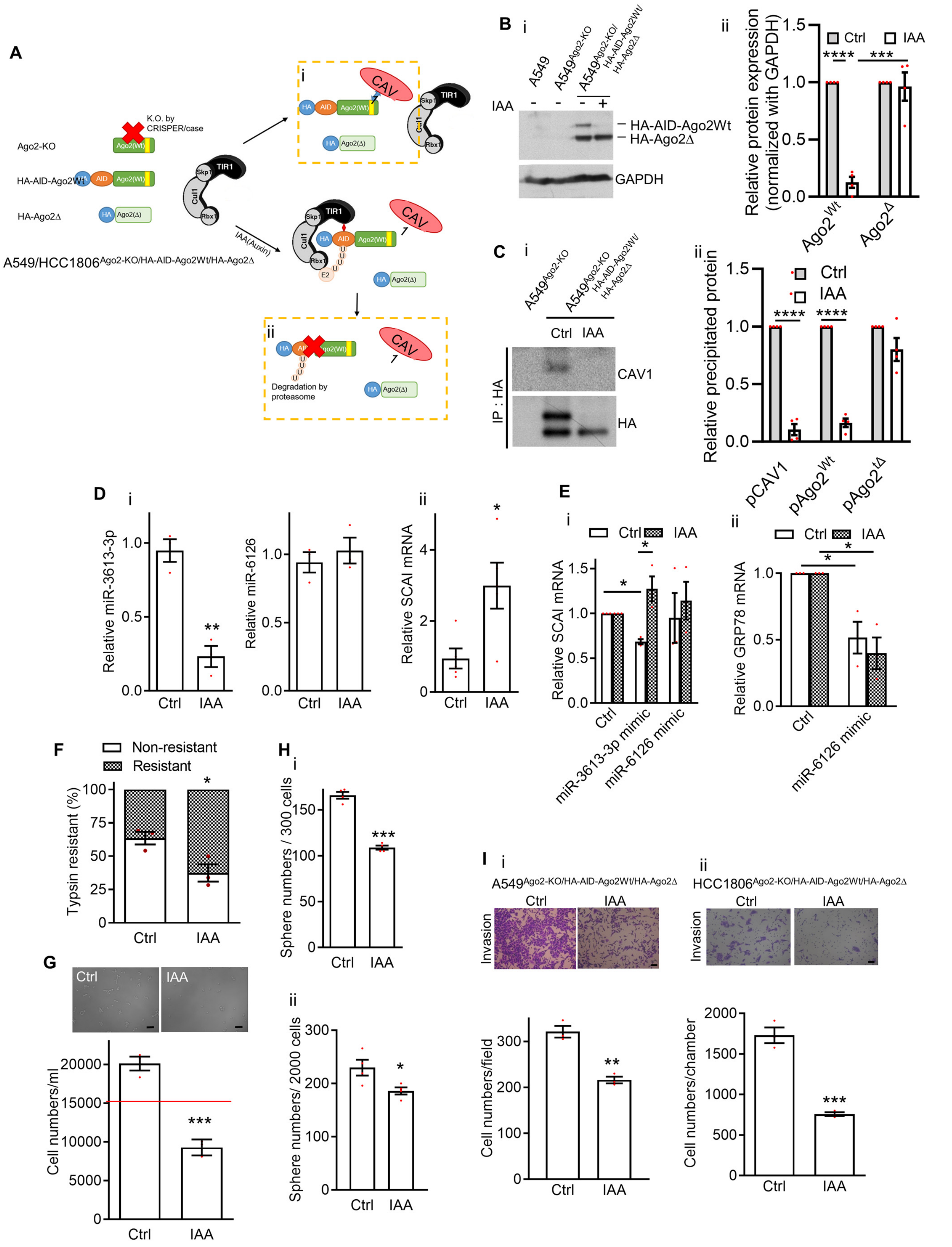
A
K.O. by CRISPER/case
Ago2-KO
HA-AID-Ago2Wt
HA-Ago2Δ
A549/HCC1806Ago2-KO/HA-AID-Ago2Wt/HA-Ago2Δ
IAA(Auxin)
Degradation by proteasome
B i ii
IAA
HA-AID-Ago2Wt
HA-Ago2Δ
GAPDH
Relative protein expression (normalized with GAPDH)
Ctrl IAA
C i ii
Ctrl IAA
IP : HA
CAV1
HA
Relative precipitated protein
pCAV1
D i ii
Relative miR-3613-3p
Relative miR-6126
Relative SCAI mRNA
E i ii
Relative SCAI mRNA
Relative GRP78 mRNA
miR-3613-3p mimic
miR-6126 mimic
F
Non-resistant
Resistant
Typsin resistant (%)
G
Cell numbers/ml
H i ii
Sphere numbers / 300 cells
Sphere numbers/ 2000 cells
I i ii
A549Ago2-KO/HA-AID-Ago2Wt/HA-Ago2Δ
HCC1806Ago2-KO/HA-AID-Ago2Wt/HA-Ago2Δ
Invasion
Cell numbers/field
Cell numbers/chamber
Ctrl
IAA

 © The Author(s)

◀ **Figure EV3. Cancer cells with inducible disruption of Ago2/CAV1 interaction.**

(**A**) Blockage of Ago2/CAV1 interaction with AID (see "Materials and Methods"). In A549$^{Ago2\text{-}KO/HA\text{-}AID\text{-}Ago2Wt/HA\text{-}Ago2\Delta}$ and HCC1806$^{Ago2\text{-}KO/HA\text{-}AID\text{-}Ago2Wt/HA\text{-}Ago2\Delta}$ cells, OsTIR1 E3 ligase increased the ubiquitylation of HA-AID-Ago2Wt in the presence of IAA, which in turn resulted in protein degradation. IAA-treated cells expressed only HA-Ago2Δ, which does not bind to CAV1. Ago2/CAV1 interaction was blocked in IAA-treated cancer cells with an auxin-inducible degron (AID) system. (**B**) Expression of HA-Ago2 and HA-Ago2Δ of A549$^{Ago2\text{-}KO/HA\text{-}AID\text{-}Ago2Wt/HA\text{-}Ago2\Delta}$ under auxin-inducible degradation. A549$^{Ago2\text{-}KO}$: A549 cells with Ago2 knocked out by CRISPER/Cas9 gene editing. A549$^{Ago2\text{-}KO/HA\text{-}AID\text{-}Ago2Wt/HA\text{-}Ago2\Delta}$: Ago2-knockout A549 cells expressing HA-AID-tagged wild-type Ago2 and HA-tagged CBM-deleted (amino acids 175–226) Ago2. Panel i: The expression of Ago2 was analyzed using anti-HA antibodies, with GAPDH used as a loading control. Panel ii: The quantitation of Western blots of experiments in panel i. Each spot describes the level of Ago2 in the sample, relative to that of vehicle (Ctrl)-treated A549$^{Ago2\text{-}KO/HA\text{-}AID\text{-}Ago2Wt/HA\text{-}Ago2\Delta}$ cells in each replicated experiment. Bars are means ± SEM ($n = 4$, biological replicates). Student's *t*-test, $^{***}P \le 0.001$, $^{****}P \le 0.0001$ (**C**) Panel: In vehicle (Ctrl)- and IAA-treated A549$^{Ago2\text{-}KO/HA\text{-}AID\text{-}Ago2Wt/HA\text{-}Ago2\Delta}$ cells, HA-AID-Ago2Wt and HA-Ago2Δ proteins were immunoprecipitated with anti-HA antibodies, and coprecipitation of CAV1 was analyzed. A549$^{Ago2\text{-}KO}$ was used as a negative control. Panel ii: The quantitation of Western blots of experiments in panel i. Each spot describes the level of the precipitated protein, relative to that of vehicle (Ctrl)-treated A549$^{Ago2\text{-}KO/HA\text{-}AID\text{-}Ago2Wt/HA\text{-}Ago2\Delta}$ cells in each replicated experiment. Bars are means ± SEM ($n = 4$, biological replicates). Student's *t*-test, $^{****}P \le 0.0001$. (**D**) Expression of miR-3613-3p, miR-6126 (panel i), and SCAI mRNAs (panel ii) in PBS- and IAA-treated A549$^{Ago2\text{-}KO/HA\text{-}AID\text{-}Ago2Wt/HA\text{-}Ago2\Delta}$ cells. Each spot describes the relative miRNA or mRNA expression level of the sample to that of the PBS-treated sample. miRNA data normalized to U6 snRNA and mRNA data normalized to GAPDH mRNA. Bars are means ± SEM ($n = 3$ and 4, biological replicates). Student's *t*-test, $^{*}P \le 0.05$; $^{**}P \le 0.01$. (**E**) Expression of SCAI (left panel) and GRP78 (right panel) mRNAs in PBS- and IAA-treated A549$^{Ago2\text{-}KO/HA\text{-}AID\text{-}Ago2Wt/HA\text{-}Ago2\Delta}$ cells with miR-3613-3p or miR-6126 mimics. Each spot describes the relative mRNA expression level of the sample to that of the A549 cells without miRNA mimics (Ctrl). Bars are means ± SEM ($n = 3$, biological replicates). Student's *t*-test, $^{*}P \le 0.05$. (**F**) Blockage of Ago2/CAV1 interaction increased the resistance of cancer cells to trypsinization. A549$^{Ago2\text{-}KO/HA\text{-}AID\text{-}Ago2Wt/HA\text{-}Ago2\Delta}$ cells were treated with vehicle or IAA. Treated cells were evaluated in terms of resistance to 0.05% trypsin. Bars are means ± SEM ($n = 3$, biological replicates). Student's *t*-test, $^{*}P \le 0.05$. (**G**) Blockage of Ago2/CAV1 interaction decreased the resistance of cancer cells to anoikis. The numbers of IAA/PBS-treated A549$^{Ago2\text{-}KO/HA\text{-}AID\text{-}Ago2Wt/HA\text{-}Ago2\Delta}$ cells in suspension were evaluated in terms of resistance to anoikis. The red line indicates the initial cell number on day 0. Bars are means ± SEM ($n = 3$, biological replicates). Student's *t*-test, $^{***}P \le 0.001$. Scale bar = 50 μm. (**H**) Blockage of Ago2/CAV1 interaction decreased tumorsphere formation. A549$^{Ago2\text{-}KO/HA\text{-}AID\text{-}Ago2Wt/HA\text{-}Ago2\Delta}$ (panel i) and HCC1806$^{Ago2\text{-}KO/HA\text{-}AID\text{-}Ago2Wt/HA\text{-}Ago2\Delta}$ (panel ii) cells were treated with vehicle or IAA. The cells were subjected to tumorsphere assays, and treatment was suspended during the assays. Bars are means ± SEM ($n = 4$, biological replicates). Student's *t*-test, $^{*}P \le 0.05$; $^{***}P \le 0.001$. (**I**) Blockage of Ago2/CAV1 interaction decreased the invasion and migration of cancer cells. A549$^{Ago2\text{-}KO/HA\text{-}AID\text{-}Ago2Wt/HA\text{-}Ago2\Delta}$ (panel i) and HCC1806$^{Ago2\text{-}KO/HA\text{-}AID\text{-}Ago2Wt/HA\text{-}Ago2\Delta}$ (panel ii) cells were treated with vehicle or IAA. The cells were subjected to invasion assays, and treatment was suspended during the assays. Bars are means ± SEM ($n = 3$, biological replicates). Student's *t*-test, $^{**}P \le 0.01$, $^{***}P \le 0.001$. Scale bar = 100 μm.

© The Author(s)

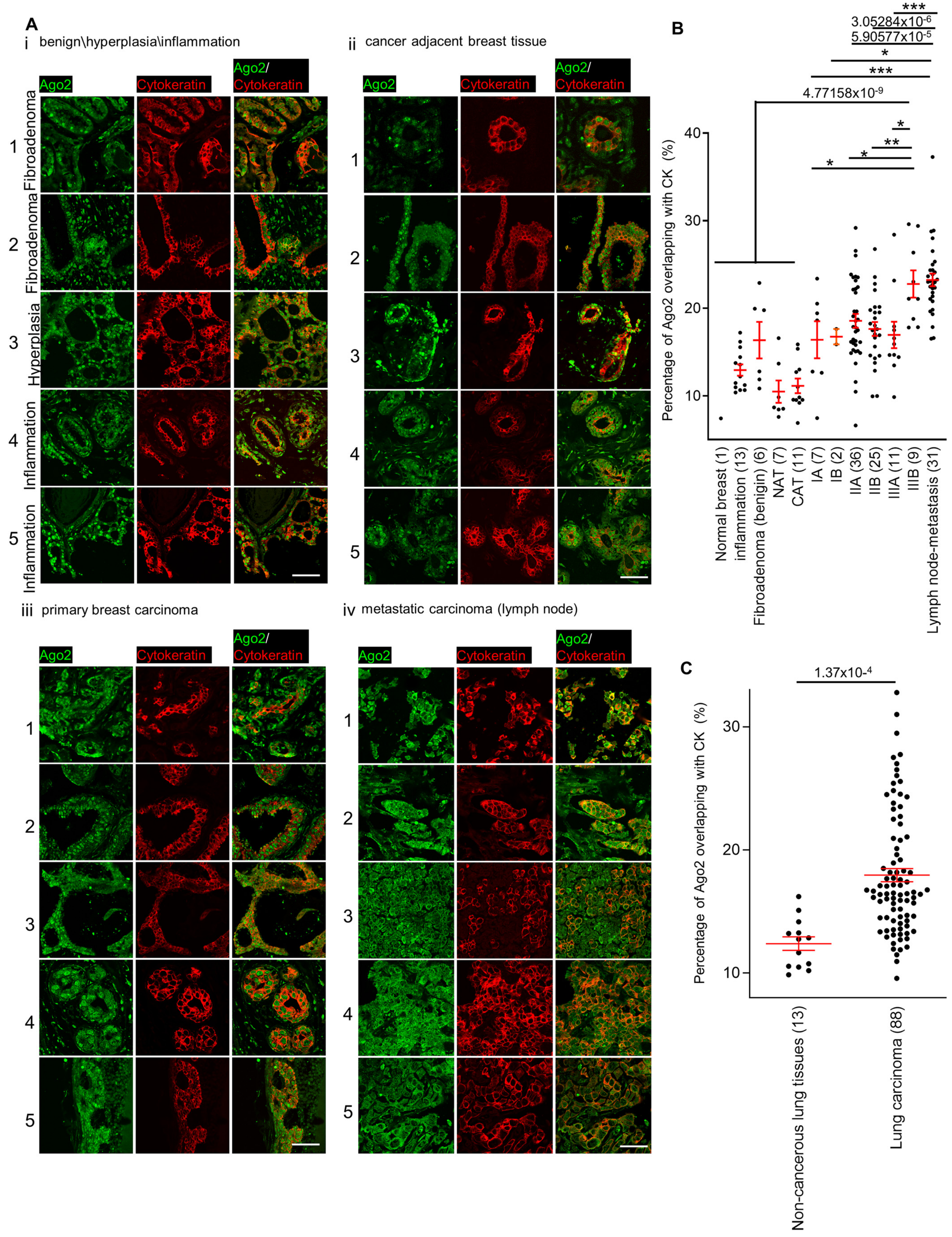
A
i benign\hyperplasia\inflammation
Ago2
Cytokeratin
Ago2/ Cytokeratin
1 Fibroadenoma
2 Fibroadenoma
3 Hyperplasia
4 Inflammation
5 Inflammation
ii cancer adjacent breast tissue
iii primary breast carcinoma
iv metastatic carcinoma (lymph node)
B
Percentage of Ago2 overlapping with CK (%)
Normal breast (1)
inflammation (13)
Fibroadenoma (benigin) (6)
NAT (7)
CAT (11)
IA (7)
IB (2)
IIA (36)
IIB (25)
IIIA (11)
IIIB (9)
Lymph node-metastasis (31)
4.77158x10-9
5.90577x10-5
3.05284x10-6
***
*
**
C
1.37x10-4
Percentage of Ago2 overlapping with CK (%)
Non-cancerous lung tissues (13)
Lung carcinoma (88)

 © The Author(s)

Figure EV4. Ago2 distribution in breast and lung disease.

(**A**) Ago2 distribution (red) in tissues in the breast disease spectrum array, analyzed using immunofluorescence. Cytokeratin was recognized using anti-cytokeratin 8/18 (green). Images of representative samples are shown. Scale bar = 50 μm. Representative immunofluorescence images of tissues in the breast disease spectrum array are depicted. Scale bar = 50 μm. (**B**) Percentage of Ago2 overlaps with cytokeratin 8/18 in images of tissues in the breast disease spectrum array. Data were identical to those shown in Fig. 8F and are re-sorted by the cancer stage along the *x*-axis. Each spot describes the percentage of Ago2 overlaps with cytokeratin 8/18 in a tissue sample. Bars are means ± SEM. The sample size (*n*, patient number) of each category is depicted on the *x*-axis. Student's *t*-test, $^{*}P \le 0.05$; $^{**}P \le 0.01$; $^{***}P \le 0.001$. (**C**) Ago2 distribution in lung tissues and lung carcinomas in the lung disease spectrum array. Each spot describes the percentage of Ago2 overlaps with cytokeratin 8/18 in a tissue sample. Bars are means ± SEM ($n = 13$ and 88, patient number). Student's *t*-test, *P* values are indicated on each plot.

© The Author(s)

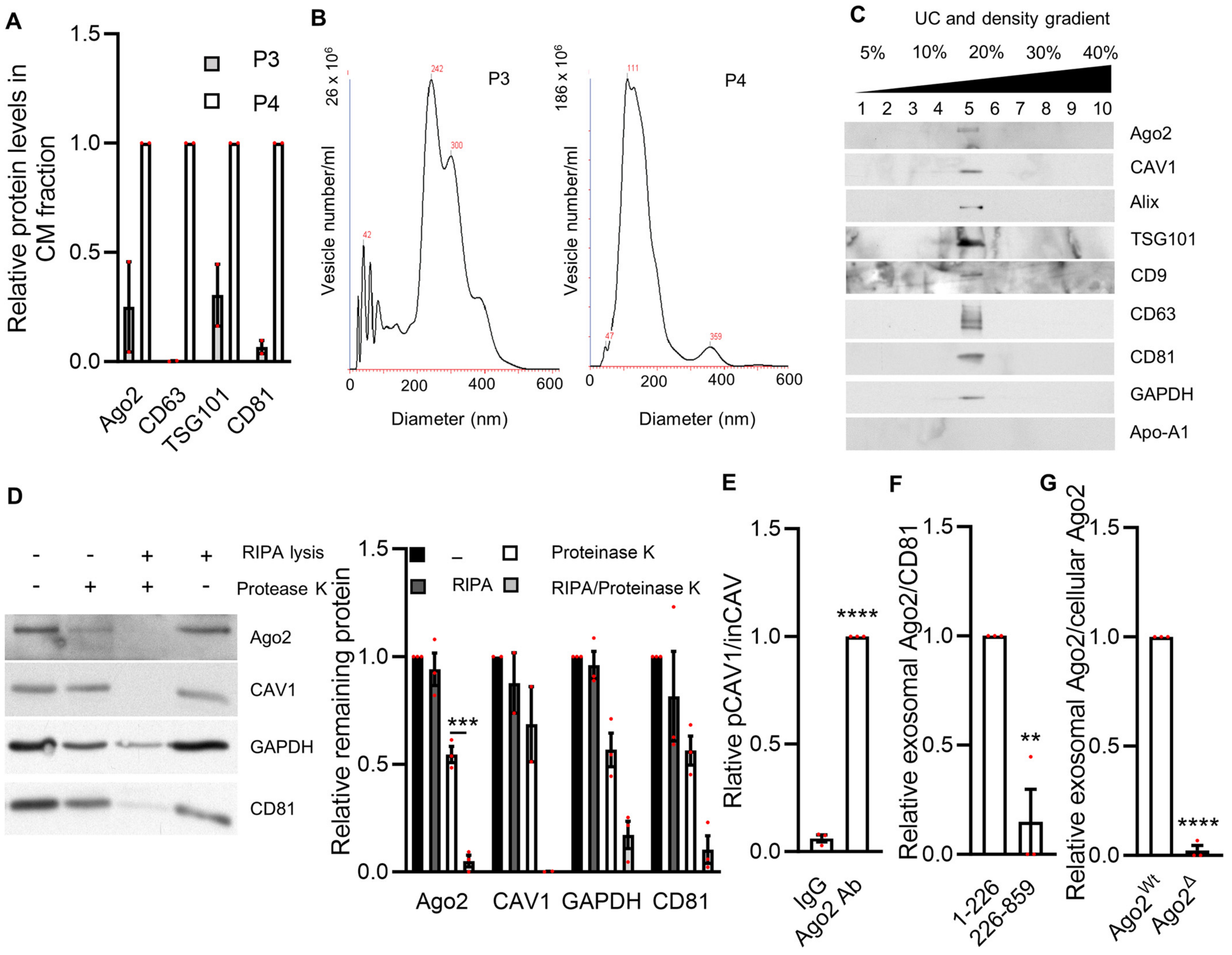

Figure EV5. Both Ago2 and CAV1 exist in cancer cell-derived EVs.

(**A**) The quantitation of Western blots of experiments in Fig. 9A. Each spot describes the protein level of the sample normalized by GAPDH, relative to that of the fourth fraction sample in each replicated experiment. Bars are means ± SEM ($n = 2$). (**B**) P3 and P4 fractions of A549 cell-conditioned media were collected and subjected to NanoSight nanoparticle tracking analysis, revealing their particle size and concentration. (**C**) Ago2 and small EVs in the same fraction of A549 cell-conditioned media separated with a density gradient. Ago2, CAV1, CD63, TSG101, CD9, and CD81 were measured using Western blotting in each fraction of the density gradient. (**D**) Proteinase K protection assay revealing Ago2 in EVs. A549 cell-derived EVs were treated using RIPA lysis buffer or protease K as indicated. Ago2, CAV1, and CD81 were measured using Western blotting in the treated EVs. Right panel: the quantitation of Western blots. Each spot describes the protein level, relative to that of PBS(-)-treated sample in each replicated experiment. Bars are means ± SEM ($n = 3$, biological replicates). Student's *t*-test, $^{***}P \leq 0.001$. (**E**) The quantitation of Western blots of experiments in Fig. 9B. Each spot describes the ratio of precipitated CAV1 (pCAV1) to input CAV1 (inCAV1) in the sample, relative to that of the sample precipitated by anti-Ago2 antibodies in each replicated experiment. Bars are means ± SEM ($n = 3$, biological replicates). Student's *t*-test, $^{****}P \leq 0.0001$. (**F**) The quantitation of Western blots of experiments in Fig. 9C. Each spot describes the exosomal Ago2 protein level of the sample normalized by exosome marker CD81, relative to that of the exosome sample of HEK293 cells expressing HA-Ago2(1–226) in each replicated experiment. Bars are means ± SEM ($n = 3$, biological replicates). Student's *t*-test, $^{**}P \leq 0.01$. (**G**) The quantitation of Western blots of experiments in Fig. 9D. Each spot describes the ratio of exosomal Ago2 to cellular Ago2 in the sample, relative to that of A549$^{Ago2KO/HA\text{-}Ago2Wt}$ (Ago2^{Wt}) sample in each replicated experiment. Bars are means ± SEM ($n = 3$, biological replicates). Student's *t*-test, $^{****}P \leq 0.0001$.

 © The Author(s)
